# Mechanistic Understanding from Molecular Dynamics in Pharmaceutical Research 2: Lipid Membrane in Drug Design

**DOI:** 10.3390/ph14101062

**Published:** 2021-10-19

**Authors:** Tomasz Róg, Mykhailo Girych, Alex Bunker

**Affiliations:** 1 Department of Physics, University of Helsinki, 00014 Helsinki, Finland; mykhailo.girych@helsinki.fi; 2 Drug Research Program, Division of Pharmaceutical Biosciences, Faculty of Pharmacy, University of Helsinki, 00014 Helsinki, Finland; alex.bunker@helsinki.fi

**Keywords:** molecular modeling, permeability, membrane disruption, membrane proteins, drugs, antimicrobial peptides

## Abstract

We review the use of molecular dynamics (MD) simulation as a drug design tool in the context of the role that the lipid membrane can play in drug action, i.e., the interaction between candidate drug molecules and lipid membranes. In the standard “lock and key” paradigm, only the interaction between the drug and a specific active site of a specific protein is considered; the environment in which the drug acts is, from a biophysical perspective, far more complex than this. The possible mechanisms though which a drug can be designed to tinker with physiological processes are significantly broader than merely fitting to a single active site of a single protein. In this paper, we focus on the role of the lipid membrane, arguably the most important element outside the proteins themselves, as a case study. We discuss work that has been carried out, using MD simulation, concerning the transfection of drugs through membranes that act as biological barriers in the path of the drugs, the behavior of drug molecules within membranes, how their collective behavior can affect the structure and properties of the membrane and, finally, the role lipid membranes, to which the vast majority of drug target proteins are associated, can play in mediating the interaction between drug and target protein. This review paper is the second in a two-part series covering MD simulation as a tool in pharmaceutical research; both are designed as pedagogical review papers aimed at both pharmaceutical scientists interested in exploring how the tool of MD simulation can be applied to their research and computational scientists interested in exploring the possibility of a pharmaceutical context for their research.

## 1. Introduction

In the most breathtaking giant leap forward in life science since the determination of the double helix structure of DNA, the general structure from the sequence problem has now been solved for the case of an individual protein domain [1]. Additionally, in 2020, the Human Proteome project, after ten years of work, reported that their complete high-stringency blueprint of the human proteome is 90.4% complete [2]; thus, an accurate sequence of every human protein and the most common variants is in sight. Together, these developments mean that we can now foresee being in possession of accurate structures for the active sites of all human proteins and most common variants; with predicted advances in computational power and the development of better algorithms, it is no longer fantasizing to speculate that, in the near future, it could be possible to obtain a sufficiently accurate estimate of the binding free energy for any given drug candidate molecule for every single possible human protein active site it may encounter. This may represent a Holy Grail of drug design; however, it does not mean the problem of computational drug design will have become a solved problem—it leaves out the rest of the biophysical landscape within which drug action takes place. This review paper explores the role that computational modeling, using the toolkit of molecular dynamics (MD) simulation, can play regarding drug design outside of this “lock and key” paradigm, focusing on one fundamentally important aspect: the lipid membrane.

In our previous review paper [3], part 1 of this series, we presented many examples of how MD simulation has been used as a tool to provide mechanistic insight relevant to drug delivery and how this insight has been used, in concrete practical terms; we now continue the discussion of the role MD simulation has and will continue to play in pharmaceutical research, focusing, in this review paper, on its role in drug design, specifically covering its ability to elucidate a central element left out of the conventional drug design paradigm: lipid membranes. As with part 1, this is a pedagogical review, with two separate target audiences, pharmaceutical researchers interested in understanding the increasing role MD simulation can play in their research, and computational researchers interested in the possibility of developing a pharmaceutical context for their research.

In part 1, we reviewed what MD simulation is and the limitations of both the aforementioned conventional “lock and key” approach to drug design [4,5] and the absorption, distribution, metabolism, and excretion (ADME) approach to drug delivery [6,7,8] that has led to the diminishing returns known as “Eroom’s law” [9]. We showed how MD simulation can provide mechanistic insight needed for the development of advanced drug delivery mechanisms. We argued that research has been hamstrung by the limited paradigm used and that MD simulation has the power to bring into consideration the broader biophysical context within which drug delivery occurs; we now proceed to argue the same for the case of drug design. As discussed in part 1, drug design has been carried out within a limited paradigm that only considered a compromise between the drug interaction with the active site of a specific target protein and its solubility; increasingly sophisticated techniques are being used to approximate the relative binding free energies of different drugs, in each case a balance being reached between maximizing accuracy and minimizing the computational resources used [10]. Although techniques have recently been developed to consider the importance of binding kinetics [10,11,12,13,14], in addition to thermodynamics, this still does not take the broader biophysical context in which the drug interaction with the protein occurs into consideration, i.e., the environment beyond the immediate drug protein interaction; additionally, it is possible for drugs to have an effect that does not even involve interaction with a protein, i.e., a mode of action entirely external to the “lock and key” paradigm.

The elucidation of all aspects of the biophysical context of drug action is currently beyond reach and even all aspects that MD simulation is capable of elucidating are beyond the scope of a single review paper; we thus focus on an important element involved in drug action left out of the “lock and key” paradigm: lipid membranes. Biomembranes, complex fluid structures formed primarily from bilayers of phospholipids, are one of the primary building blocks of life [15,16,17,18]. In all cases, drug access to its target and action involves, in some fashion, interaction with biomembranes. Drug molecules must traverse membranes that form biological barriers. The majority of proteins that are drug targets are associated with biomembranes and the membrane plays a role in the interaction with substrates, thus potential drug molecules; interactions of both target proteins and drugs, with the membrane to which the protein is associated, play a role in the drug–target protein interaction. Finally, there are cases where the mode of action of the drug does not even involve interaction with a protein; the drug acts directly on a specific biomembrane rather than with a protein. In all of these cases, MD simulation, a mature tool for the study of biomembranes [19,20,21,22,23,24,25,26,27], can provide a window on the role the lipid membrane plays in drug action; this review paper describes how MD provides mechanistic insight and includes many examples where it has been successfully applied. We will first discuss the behavior of drug molecules in the membrane, then how drugs transfect through membranes that form biological barriers and how drugs can, collectively, at sufficient concentration, affect the properties of the membrane. Finally, we will discuss the role the membrane plays in the selection of substrates, thus potential drug molecules, for membrane associated proteins. In all these areas, MD simulation has acted as a unique window capable of adding mechanistic insight that can be applied in drug design; we describe many case studies where this has been used effectively. 

While previous review papers have shown how MD simulation has helped elucidate the role the lipid membrane plays in substrate and thus drug selection for membrane proteins [28,29,30,31,32], drug membrane interactions [33,34,35,36,37,38,39,40,41], drug delivery [3,42,43,44,45], antimicrobial peptides [46,47,48,49,50,51,52,53,54], and methodologies [28,55,56,57,58,59,60], this is the first review paper, that we are aware of, focusing on the entirety of the use of MD simulation to incorporate the role played by interactions with lipid membranes in drug design. This can be seen, in turn, as a case study of the potential for MD simulation to expand the paradigm of drug design to all aspects of the broader biophysical environment within which drug action occurs. 

**Figure 1:**
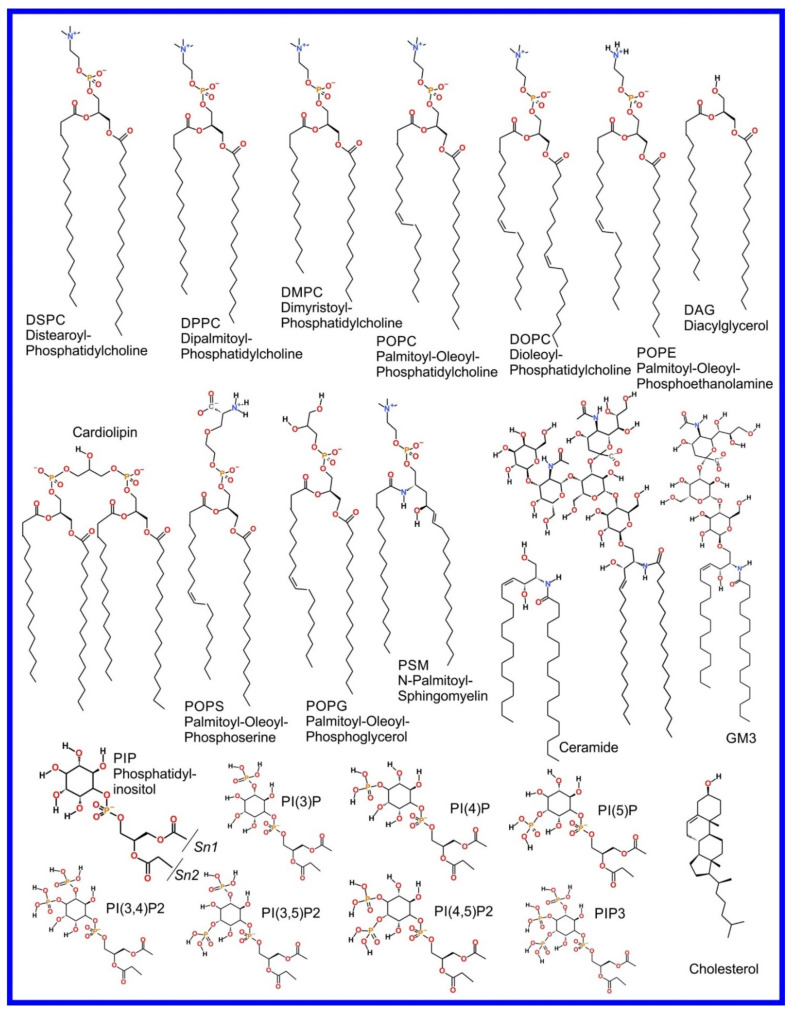
Chemical structure of lipids mentioned in this paper.

We now refer the reader to our discussion of the basics of the MD simulation method in our previous review paper, i.e., “part 1” [3], or an equivalent discussion found in Braun et al. [61], where its ability to provide an effective visualization of the system studied with all atom resolution (also reviewed in [62,63,64,65]) and how coarse grained (CG) potential sets can be used to effectively zoom out to study larger length and time scales (also reviewed in [66,67,68,69,70]) are covered. However, the application of MD simulation to the study of the more complex systems involved in drug action rather than drug delivery, that we cover in this review, requires advanced MD simulation related methodologies not discussed in the aforementioned references; these will now be described and examples of their use will be encountered later, throughout the rest of the paper. As an aside, it is appropriate that we now draw attention to the fact that the chemical structures of the lipids discussed in this manuscript are shown in Figure 1.

## 2. Advanced Simulation Methods

In its pure form, MD simulation provides a window into the system, with all atom resolutions, at a timescale of up to 1–2 μs and a box size of up to 15–20 nm, significantly greater with CG models [3]. In our previous review paper [3], we discussed many examples where this has been used in issues related to drug delivery, and how it is a valuable tool when used its in pure form; however, significant limitations are encountered when one addresses issues related to drug design rather than delivery.

When expanding what we consider in drug design the oversimplified “lock and key” paradigm to the broader context of all the relevant biophysics, there are many challenges that result from the added complexity. Within the context of biophysics, drug design can be studied through the paradigm of the interaction of four different basic varieties of entities, each of which present their own challenges: (1) small drug molecules, which, while simple in structure, are often chemically/structurally novel as they are often synthetically created molecules that do not naturally exist, thus in many cases new parameter sets must be developed and extra challenges are encountered in attempting the development of CG models; (2) specifically structured large biomolecules, e.g., proteins and nucleic acids, that have a highly specific structure defined by a very rigid, steep, and complex energy landscape that molecules that interact with them must negotiate; (3) large unstructured molecules, e.g., polymers that, while without specific structure but with an uncomplicated energy landscape governing their interactions, exhibit complex behavior that is difficult to model due to topological effects that arise spontaneously due to their size and flexibility; (4) lipid membranes, that are flexible, complex, and diverse entities with very complex interactions with other elements. Clearly, one must adopt more advanced methodologies rooted in MD simulation to explore this complex landscape and these will now be introduced. Discussing this will, however, require the presentation of some mathematical and physical (thermodynamic) constructs. 

Let us consider our window into a given physical system that is provided by MD simulation: a set of molecules with periodic boundary conditions being simulated at temperature T, e.g., a section of lipid membrane, protein, or other macromolecule or molecular complex in solvent. Technically, this can be seen as a set N particles governed by a potential that is a function of the position of all particles: $U({\vec{r}}^{N})$; ${\vec{r}}^{N}$ is the position of all particles, which can in turn be seen as a point in a 3N dimensional space with $\left\{{\vec{r}}^{N}\right\}$ representing the entirety of this space, i.e., the set of all possible particle positions, known as the *conformation space*, i.e., the system of N particles governed by potential $U({\vec{r}}^{N})$ can be said to inhabit the space $\left\{{\vec{r}}^{N}\right\}$. If we begin the simulation at a certain point in this space ${\vec{r}}_{0}^{N}$ and simulate, with a thermostat holding the system at constant temperature T, using the MD simulation method described in our previous review paper “part 1” [3], then the system will move around in this space following what can be called a *trajectory*. In any real simulation there will be enough particles and $U({\vec{r}}^{N})$ will be sufficiently non-trivial so that we can assume what is known as the ergodic hypothesis: given infinite time, the system will eventually pass through all possible points in $\left\{{\vec{r}}^{N}\right\}$. The relative amount of time spent within a given 3N dimensional differential element $d{\vec{r}}^{N}$ will be equivalent to the probability of the system being within that element that we will signify as $P({\vec{r}}^{N},T)d{\vec{r}}^{N}$. For a system at constant temperature, i.e., a *canonical ensemble* [71],

(1)  P(r→N,T)=e−γZu(T)    γ=U(r→N)T

where $Z_{u}(T)$ is a normalization factor known as the *partition function* that is, for any but the most trivial systems, impossible to determine analytically; in this equation, we have assumed temperature to be in units that match $U({\vec{r}}^{N})$, thus have not included the Boltzmann constant normally seen in this equation. A clever way around the impossible to determine partition function makes use of the principle of detailed balance: given two points in the conformation space ${\vec{r}}_{1}^{N}$ and ${\vec{r}}_{2}^{N}$, the ratio of the transition probabilities between these two points, $W({\vec{r}}_{1}^{N}\to {\vec{r}}_{2}^{N},T)$ and $W({\vec{r}}_{2}^{N}\to {\vec{r}}_{1}^{N},T)$ is equal to the ratio of the probabilities of being at the two points:

(2) $$\frac{W({\vec{r}}_{2}^{N}\to {\vec{r}}_{1}^{N},T)\;}{W({\vec{r}}_{1}^{N}\to {\vec{r}}_{2}^{N},T)}=\frac{P({\vec{r}}_{1}^{N},T)}{P({\vec{r}}_{2}^{N},T)\;\;\;}$$

Given

(3) Δγ=U(r→2N)T−U(r→1N)T ,

an algorithm that satisfies all preceding relations, is: for a system at ${\vec{r}}_{1}^{N}$ considering a transition to ${\vec{r}}_{2}^{N}$, if Δγ≤0 the system moves to ${\vec{r}}_{2}^{N}$ otherwise the transition occurs with probability $e^{-\Delta \gamma }$. This can be continued indefinitely, following what is known as a *Markov chain*, moving through conformation space sampling a set of states that will have a distribution of $P({\vec{r}}^{N},T)$ in the limit of infinite steps. The result of carrying this out for a finite number of steps is thus an approximation of $P({\vec{r}}^{N},T)$, that we will refer to as $\bar{P({\vec{r}}^{N},T)}$; as the number of steps in the Markov chain increases, the longer the trajectory, the greater the accuracy of our approximation. The simulation of a system in this fashion is known as a *Monte Carlo (MC) simulation* [72].

If one is able to concisely calculate $P({\vec{r}}^{N},T)$ over all $\{{\vec{r}}^{N}\}\;$for any system, then one will know everything that is to be known about that system and any property of that system can be calculated; this is, however, impossible for all but the most trivial systems. Yet, both MC and MD simulation are able to provide an incomplete estimate of this: the above mentioned $\bar{P({\vec{r}}^{N},T)}$; the longer the trajectory the more information, for the case of MD as well as MC simulation. Depending on how the algorithms are constructed, each have their relative strengths and weaknesses and can be hybridized, particularly since MD simulation has been shown to be superior at relaxing the local degrees of freedom, while MC simulation, if designed properly, can more effectively relax the more global degrees of freedom [73,74,75,76]. While MD simulation, if carried out on its own, can also provide information related to dynamics, this information is usually of secondary importance; we will only consider MC and MD simulation as means to provide a result for $\bar{P({\vec{r}}^{N},T)}$ here.

**Figure 2:**
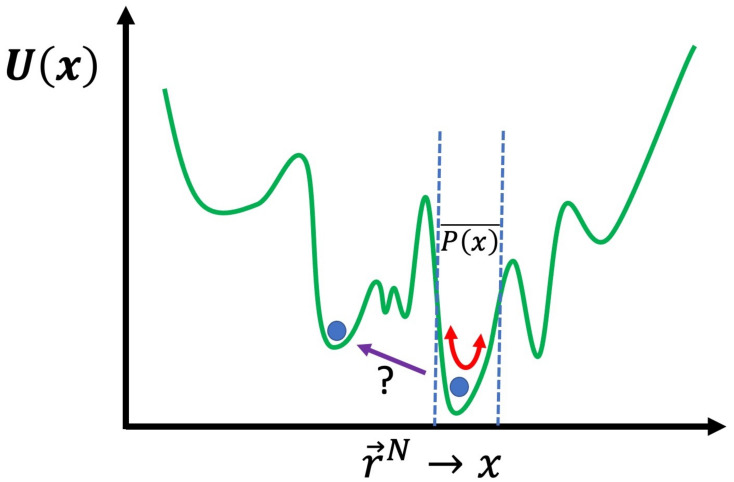
Schematic of the energy landscape of a system; with basic unbiased MD or MC simulation, if performed below a certain temperature, only the region of the local minimum will be explored, thus a result for $\bar{P({\vec{r}}^{N},T)}$ will only be obtained here. Finding the free energy change to another region of conformation space, where the energy barrier must be crossed and $\bar{P({\vec{r}}^{N},T)}$ determined along the path, is not practically possible without the advanced biasing schemes we discuss.

The problem with both MC and MD simulation, or even the two hybridized, in their basic forms, is that the approximation of $P({\vec{r}}^{N},T)$ ($\bar{P({\vec{r}}^{N},T)}$) will provide the most information in the regions of the conformation space of the system where $P({\vec{r}}^{N},T)$ is greatest. For many cases, for example, the aforementioned systems related to drug delivery [3], this may be sufficient, i.e., the answer to the question “what does the system normally do?” may be all we seek. There are many cases where this will not be sufficient, for example, when calculating the free energy to cross certain barriers from one region of conformation space to another. A useful schematic is to express ${\vec{r}}^{N}$ as a single dimension x rather than 3N dimensions and plot $U({\vec{r}}^{N})$ as U(x), as shown in Figure 2. The energy landscape is very rough with many peaks and valleys; with either MC or MD, in their basic form, crossing a very high energy barrier will be an extremely unlikely event. If you have managed to determine the global energy minimum, then you will only obtain results for $\bar{P({\vec{r}}^{N},T)}$ in the local valley of the global minimum. Even worse, usually, you cannot even be certain you have found the global minimum and thus will only be exploring the vicinity of a local minimum. In general, the primary concern is the region of $\left\{{\vec{r}}^{N}\right\}$ where you need to obtain information regarding $P({\vec{r}}^{N},T)$. If you wish to calculate the free energy difference, between two different local minima, then you will need to obtain a result for $\bar{P({\vec{r}}^{N},T)}$ in the region of conformation space along the path that connects them. For example, for the affinity of a drug for a protein, we need to determine the free energy difference between the drug in solution and the drug bound to the specific binding site of the protein that is being targeted. Moreover, in many cases, what is of interest in the simulation is what is known as a “rare event”; this corresponds to a transition through a region of conformation space with low $P({\vec{r}}^{N},T)$. We will now discuss how approximate values of such free energies can be calculated and rare events can be sampled more effectively, using advanced simulation methods.

The most direct way to increase the region of space where $\bar{P({\vec{r}}^{N},T)}$ is obtained, and to ensure the rare events that we wish to observe are sampled, is to globally accelerate the sampling. The simplest way to achieve this is to just artificially raise the concentration of the molecules that would trigger the event. This is known as a *flooding simulation* [77]. Another simple method is to perform an MD simulation at an artificially raised temperature, to move the system through conformation space more quickly and through greater thermal excitation push the system through energy barriers that would otherwise not be crossed. Obviously, just simply raising the temperature will often be problematic as the alterations to the system, e.g., passing through phase transitions, that happen in the real system with elevated temperature, will often occur in the model, producing a false result. A way around this is to anticipate the unphysical changes to the system the temperature rise will invoke and add a bias potential to $U({\vec{r}}^{N})$ to block this from occurring while raising the temperature; this method is known as temperature accelerated molecular dynamics (TAMD) [78]. While TAMD has been used effectively in many cases, it suffers from the problem that the bias potential may not completely alleviate the undesired temperature effects. A safer but more complex method to achieve the same acceleration, but with a guarantee of obtaining an unbiased result, is Replica Exchange.

**Figure 3:**
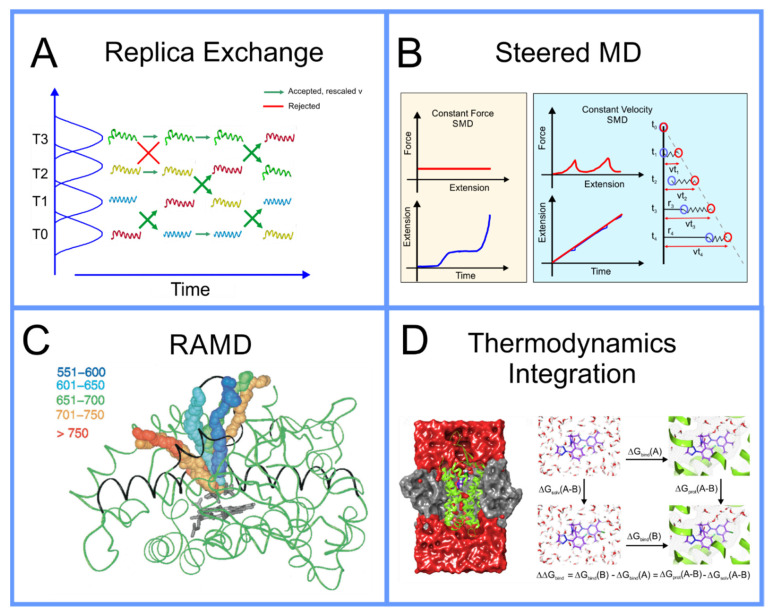
(**A**) Schematic representation of replica exchange (based on ref. [28]); (**B**) force bias/Jarzynsky, reproduced with permission from ref. [94]; (**C**) RAMD, reproduced with permission from ref. [102]; (**D**) thermodynamics integration, reproduced with permission from ref. [103].

In replica exchange [79,80,81], originally described as *parallel tempering* in the seminal literature, the system of N particles governed by potential $U({\vec{r}}^{N})$ that inhabits the space $\left\{{\vec{r}}^{N}\right\}$ being simulated at temperature T, using either MD or MC, can be simulated in parallel with a set of other systems with the same set of particles, however, with other parameters altered. The set of systems can be simulated as a single extended ensemble; this can be seen as a metaverse of parallel universes, a concept that a broader segment of the audience of this paper can understand intuitively now, due to the popularity of recent movies and television shows that have featured such plot elements. Within this new extended ensemble, switching the parameters between two, so to speak, universes within the multiverse that we have created, can be seen as an MC step that can be made with probability $e^{-\Delta \gamma }$ where γ is as above, however, now rather than alteration of particle positions, i.e., a change in ${\vec{r}}^{N}$, the change in γ, Δγ, results from temperatures and/or potential functions that governs the systems switching; a schematic of this algorithm is found in Figure 3A. It can be demonstrated mathematically that not only does the collective metaverse that we have created move to equilibrium, but each individual system, separately, moves to equilibrium, thus systems with parameter sets that move more slowly through conformation space are effectively pulled along by those that move more quickly. Initially, this was performed with multiple systems that differed in thermodynamic parameters of the system, like temperature and pressure [82]. Then, Bunker and Dünweg realized that one could more precisely accelerate the dynamics by having the systems differ not in a thermodynamic parameter, but rather a term in the interaction potential $U({\vec{r}}^{N})$; specifically, they proposed eroding the repulsive core of the Lennard–Jones potentials, allowing for a finite acceptance of MC pivot moves hybridized with MD simulation in the backbones of polymer chains [76] and referred to this as parallel excluded volume tempering. Fukushini et al., then generalized this and applied it to protein structure determination and coined the term by which this procedure is known today: Hamiltonian replica exchange [83].

**Figure 4:**
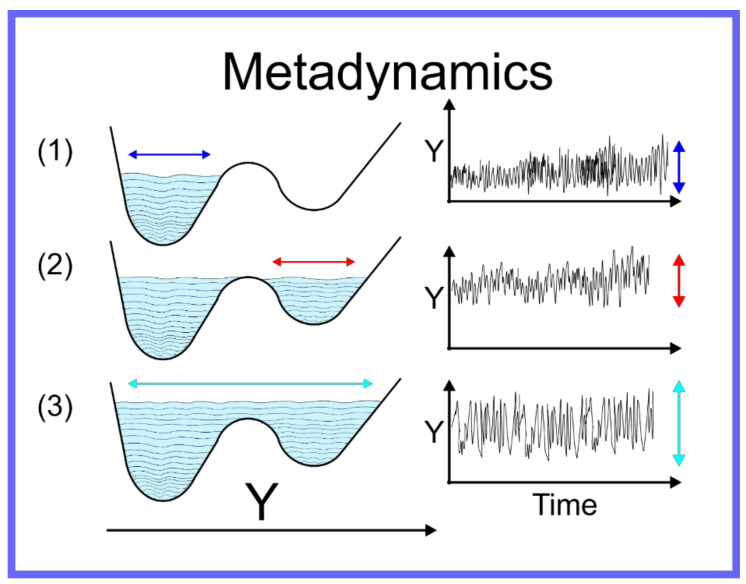
Schematic representation of metadynamics.

The aforementioned techniques globally accelerate the motion through conformation space, thus the efficiency with which the quality of $\bar{P({\vec{r}}^{N},T)}\;$as an approximation of $P({\vec{r}}^{N},T)$ is improved. There are, however, other techniques capable of focusing more specifically on the local region of the conformation space where the system is located—specifically allowing the system to escape the local minimum within which it is trapped. For a system on a discrete space, that is where ${\vec{r}}^{N}$ is a set of specific points, or states, i.e., $\{{\vec{r}}^{N}\}\to x_{i=1,N}$, Wang and Landau developed an algorithm [84] where an MC simulation of a system on the discrete space is performed and the number of times the system samples a certain state is recorded and periodically a bias is added to the potential, proportional to the number of times a state has been sampled. Metaphorically, this can be seen as filling up the energy wells that the system has already explored with sand, pushing the system over energy barriers into new energy wells, and then filling them up and so on. This algorithm was adapted to continuum systems, on which MD simulation can be performed, as Statistical Temperature Molecular Dynamics (STMD) [85] and this has been formally shown to be mathematically equivalent [86] to the better-known technique of metadynamics [87,88,89,90,91]. An example of metadynamics at work is shown in Figure 4. An alternative to metadynamics is the application of a specific alteration to the potential to push the system out of a specific local minimum; this is possible when the form of the local minimum can be approximated by an analytical function that can be cancelled out. This technique is known as the addition of a *flooding potential* [92].

We have now described methods to globally accelerate the exploration of conformation space and to, more specifically, escape from the local minima that constrain the system; this, however, still often leaves a conformation space too large to explore to obtain a sufficiently accurate result for $\bar{P({\vec{r}}^{N},T)}$ in the region of conformation space that we are specifically interested in. Often, this is the transition between two specific local minima, in particular, calculating the free energy difference between them. A specific example of this is a ligand in solution vs. the same ligand docked to a specific active site of a specific protein. The most direct way to achieve this is to push the system through a path in conformation space between the two local minima. There are two means to achieve this: (1) umbrella sampling [93] and (2) steered MD [94,95]. 

**Figure 5:**
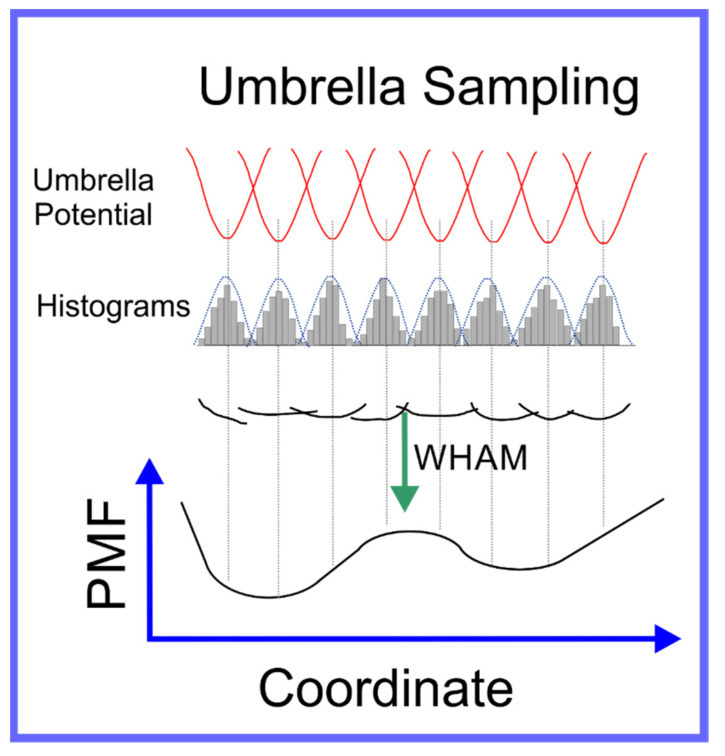
Schematic representation of umbrella sampling algorithm.

In umbrella sampling, a set of what we refer to as *windows* are created along a path in conformation space that connect the two local minima. This is achieved through the introduction of a biasing force that is in the form of a harmonic interaction, i.e., a spring, centered on a certain distance along the path in conformation space that connects the two local minima. Histograms of the distribution in position along the path in conformation space are determined for each window; a schematic of umbrella sampling is shown in Figure 5. The free energy along any path in conformation space, known as the Potential of Mean Force (PMF), can be determined from these histograms, if the overlap between their tails is sufficient, through a technique known as Weighted Histogram Analysis Method (WHAM) [96]. WHAM is itself an extension of a technique developed by Ferrenberg and Swendsen [97,98] primarily for spin models [99], to specifically adapt it to systems governed by a molecular mechanics potential. A specific example that elucidates the use of umbrella sampling is the study by Nandy et al. [100], where it was used to study the binding of PAMAM dendrimers of different generations to strands of ds-DNA; in the supplementary information of this paper the overlapping set of histograms is shown. The efficiency of the umbrella sampling algorithm can, in many cases, be significantly improved through hybridization with the replica exchange algorithm [101]; the set of all windows can be treated as a single extended ensemble where exchanges of the force biases between neighboring windows can be made through the aforementioned MC moves.

Steered MD is another technique capable of determining the free energy change along a path in conformation space that, in many cases, is significantly more computationally efficient than umbrella sampling. It is based on an astounding, counterintuitive, piece of theoretical statistical mechanics proposed by by Jarzynski in 1997, almost 20 years before the computational power to apply this algorithm effectively became available; the relation he derived [104,105], known as the *Jarzynski equality*, can be expressed as

(4) 〈e−WT〉=e−ΔFT

where 〈 〉 signifies the average over infinite samples, W is the work carried out along the path in conformation space, i.e., the integral of the force along the path, and ΔF is the difference in free energy between the two ends of the path. The free energy difference between two points in conformation space, a property defined only for a system in equilibrium, can actually be approximated from multiple samples of the work carried out pulling the system from one to the other, a driven system, i.e., a measurement of the system out of equilibrium. Steered MD involves simulating the system while pulling the system along a path in conformation space to determine the work carried out along this path; it is performed multiple times, each time a measurement of W is made. There are two fashions in which this pulling can be performed [94]: constant force, where the velocity is measured along the path, or constant velocity, where the force is measured along the path; this is shown as a schematic in Figure 3B. This algorithm was initially thought not to be practical as, in its early use, it obtained very poor results for the free energy; however, more recently, it was found that if you perform a much larger number of samples than previously feasible, one is able to obtain a result for the free energy difference that, in many cases, is significantly more accurate than that obtained using umbrella sampling using the same computational resources. This algorithm is increasingly being used in drug design [95].

The above methods presuppose that the path in conformation space that the system will traverse is known; however, this is not always the case. Often, the active site of a protein is within an internal cavity and the path through channels and mobile loops of the protein that a ligand takes to reach the active site, is unknown. In this case, a technique to not only find the free energy along a path in conformation space must be found, but the path itself must also be found. A technique capable of achieving this is Randomly Accelerated Molecular Dynamics (RAMD) [106]; this involves starting the ligand in its docked position, moving it in a random direction until it gets stuck, then picking another direction and repeating until the ligand has left the protein, bouncing back and forth until it wiggles out of the box it is in; an example of this is shown in Figure 3C. A modified version of RAMD, known as τRAMD allows for the study of the residence time of ligands in the binding pocket [107]. Two additional methods have been developed to find the optimal pathway in complex multidimensional space that bear mentioning: the first is based on the combined use of metadynamics and a path-searching algorithm [108] and the second is based on what is known as a “string method” involving a swarm of trajectories [109,110].

All of the above methods to determine the free energy between two regions of conformation space, usually two different local minima, involved moving the system through a path in conformation space. While accurate, this is not always the most efficient method if all you are interested in is a single quantitative approximation for the free energy difference between the end points of the path and not the free energy along the path, i.e., the PMF. While this information is often useful, as it provides insight into the kinetics in addition to the thermodynamics of the system [10], it does not come without a price; if you is only interested in the free energy difference between the end points of the path, there are more efficient means to obtain an approximation of this. Instead of charting a path through conformation space, you can instead move through a space composed of different possible forms of the interaction potentials, $U({\vec{r}}^{N})$, itself to determine this; if you start with, for example a ligand bound to the active site of a protein, and that same ligand free in solution, in each case you can gradually dissolve the interactions around the molecules to find the free energy difference to the molecule in empty space: subtracting these from one another determines the free energy difference between them. There are several different related methods that follow this scheme, each with different advantages and disadvantages, including Thermodynamics Integration (TI) [111], Free Energy Perturbation (FEP) [112,113,114], and Bennett Acceptance Ratio (BAR) [115,116]. If one is also only interested in the difference of the binding affinities of two different molecules and the absolute free energy of binding is not important, then one can perform what is known as *molecular alchemy*, calculating the free energy change along the path of metamorphosis from one molecule to the other, both in solution and at the binding site and subtracting these two from one another, as shown in the schematic in Figure 3D. These techniques in the context of drug design are discussed in detail in a review paper by Limongelli et al. [10].

Finally, it bears mentioning that, as in other fields of computational science, an emergent trend is the incorporation of the toolkit of novel computational tools widely known as Machine Learning (ML) into hybrid MD algorithms; for a recent review (2020) of achievements in the hybridization of MD with ML, see Noé et al. [117]. Recently, a hybrid FEP + ML algorithm was found to provide a more accurate result, given the same expenditure of computational resources, than the use of FEP alone, for the prediction of hydration free energies [118]; a hybrid MD + ML algorithm was used to predict self-solvation free energies and limiting activity coefficients [119].

## 3. Location and Orientation of Drug Molecules in the Lipid Bilayer

**Figure 6:**
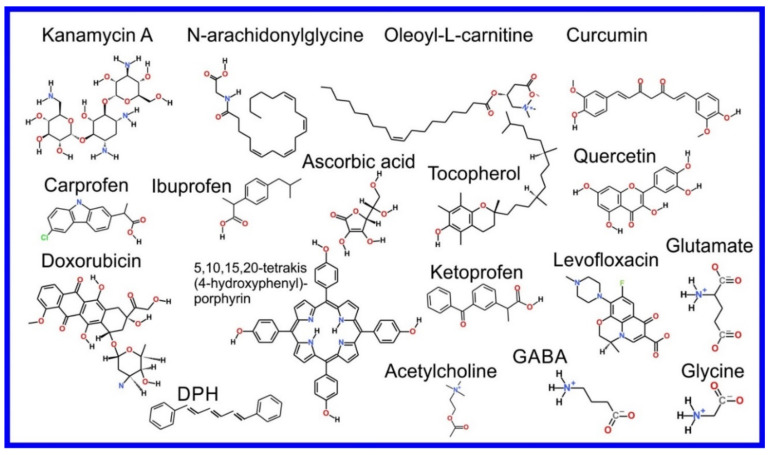
Chemical structure of drugs introduced in Section 3.

The beginning of the story of the role of lipid membranes in drug design is a discussion of what individual drugs do when they initially interact with a lipid membrane, where in the membrane they locate to and how they orient within the membrane; this is clearly a result of both the nature of the lipid membrane, i.e., what particular phospholipids and other amphiphilic molecules make up the membrane formulation and the structure of the drug molecule itself, i.e., location and relative dominance of polar, charged, and non–polar regions of the molecule and the flexibility/rigidity of the molecule (Figure 6).

The partitioning of small molecules, including proposed drug candidates (see Table 1) but also natural molecules (see Table 2) and other xenobiotic molecules (see Table 3), into lipid bilayers has been extensively studied via a combination of experimental and computational methods. How molecules interact with the membrane, i.e., how they orient and the different regions within the membrane that they locate to, is determined by their structure; MD simulation can provide significant insight into this specific selection. Through simulations carried out on many different drug molecules, a wide range of drug behavior in the membrane, i.e., location and orientation, has been observed.

**Figure 7:**
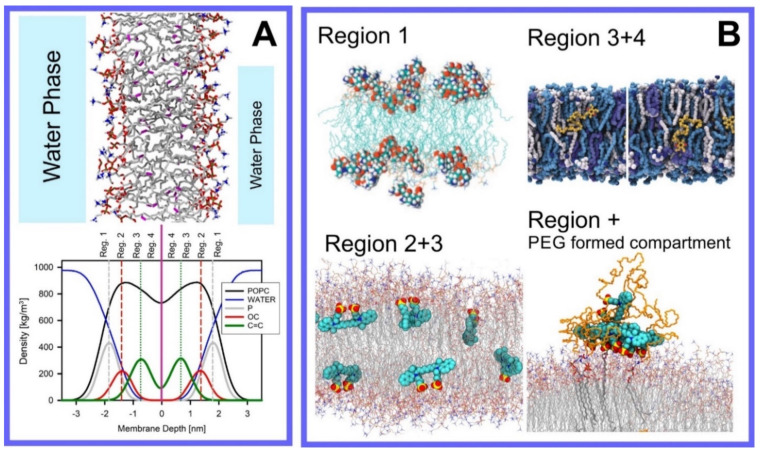
Location of the drugs and small molecules in four regions of a lipid bilayer. (**A**) Top panel: Snapshot of lipid bilayer composed of POPC; Lower panel: corresponding density profiles showing location of lipids, water, phosphate atoms, carbonyl atom (C35), and atoms of double bond of oleoyl chain (C9=C10) of POPC (see Figure 1). Data from 1000 ns simulation of hydrated POPC bilayer with our OPLSaa lipid force field [141,142,143]. (**B**) Snapshots showing location of small molecules in different regions of the bilayer: kanamycin A locates to region 1 (reproduced with permission from [133]), indocyanine green locates to region 2 and 4, and entangled in PEG corona (reproduced with permission from [144]), ubiquinone locates to region 3 and 4 (reproduced with permission from [145]).

Berendsen and coworkers [120,121] developed a classification scheme for the behavior of molecules within the membrane according to the region of the membrane that they locate to; they defined four separate regions, as shown in Figure 7: region (1) at the interface between the bulk solvent phase and the lipid headgroups, i.e., the region of the headgroups and the first shells of water adjacent to the membrane, region (2) just below the lipid headgroups, region (3) at the position of the hydrophobic lipid tails, and region (4) at the very center of the membrane core, between the two leaves of the bilayer. Through MD simulations, it has been demonstrated that small hydrophobic molecules e.g., benzene or toluene [122,123], and gases like xenon, O_2_, NO, CO, and CO_2_ [124,125,126,127,128,129] locate to region 4; larger hydrophobic molecules, e.g., porphyrins, locate to region 3 [130,131]; amphiphilic molecules, e.g., the majority of drug molecules, our primary interest in this review paper, locate to region 2, their polar components interacting with the polar groups of the lipids and water at the membrane surface and their hydrophobic components located among the hydrocarbon chains of the lipids, as shown in a study by Paloncýová et al., of drug molecules, natural compounds, and other xenobiotics [132]. Mostly polar molecules, including some drug molecules, locate to region 2. Finally, kanamycin A, a highly polar molecule with potential application as an antibiotic, locates to region 1 [133]; antibacterial peptides and saponins [134,135,136] also locate to this region prior to pore formation. It is possible for drugs to locate to the membrane in a fashion that spans more than one of these regions; examples of this case are particularly interesting as they include several lipid molecules with therapeutic potential. For example, N-arachidonylglycine and oleoyl-L-carnitine are glycine transporter GlyT2 inhibitors that span over regions 2 to 4 [137]. Nevertheless, even molecules that do not directly interact with the membrane can have a long–range membrane association. For example, the concentration of polar molecules relative to the bulk concentration may increase or decrease in the ~1 nm layer of water adjacent to the lipid bilayer; also, acetylcholine is attracted to bilayers that contain negatively charged lipids [138,139,140] and repulsed by bilayers composed of zwitterionic lipids, as determined through both the MD simulation and experimental study [139]. The zwitterionic neurotransmitters GABA and glycine are attracted to bilayers that contain anionic lipids but are, however, not affected by zwitterionic lipids [139]. Glutamate, another polar, anionic, neurotransmitter, is repulsed from the vicinity of negatively charged lipids [139].

Since drugs and lipids are generally small molecules without the structural complexity often found in biomolecules, studies of drug molecule–lipid bilayer (membrane) interactions are usually relatively straightforward to carry out, even given limited computational resources; the systems studied, lipid membranes with small drug molecules, equilibrate relatively quickly and can be studied effectively using relatively small systems. It is thus not surprising that interactions of proposed drug molecules with lipid bilayers were among the first topics to be studied using MD simulations of lipid membranes [19], for example the studies by Tu et al. [146], Pasenkiewicz-Gierula et al. [147] and Bemporad et al. [148,149] carried out in 1998, 2003, and 2005, respectively. More recently, a particularly noteworthy study by Abdiche and Myszka measured the liposome partitioning of 86 drugs and observed an astounding affinity range of ~1000 fold [150]. In another impressive study, Natesan et al., proposed a theoretical method based on drug structure that allowed for the prediction of the location of drug molecules within the bilayer [151]; this work was performed using experimental results of 107 separate small molecules interacting with lipid membranes. An extensive database was compiled that contains over 3600 cases of compound–membrane interactions gathered from both experimental and theoretical studies, known as the “Molecules on Membranes Database” (MolMeDB), [152]; MolMeDB provides data concerning drug–membrane partitioning, penetration, and positioning.

As is the case for all phenomena studied through MD simulation, the precise values for the parameters of the potential set used are of critical importance, thus the testing and validation of the model are crucial [153,154,155,156,157,158,159,160,161,162]. Partition coefficients of the molecule (log K) provide an important test of the potential set that can be directly correlated to experimental measurements. For membrane partitioning of small molecules, comparative studies have demonstrated that the best models predict the partition coefficients (log K) with an accuracy of 0.5–0.8 log units [163]. This result thus provides us with a certain degree of confidence in the accuracy with which MD simulation is capable of effectively modeling the behavior of molecules in the membrane; the partition coefficient plays a dominant role in the drug–membrane interaction. 

Simulation studies using MD have demonstrated that the location of the drug molecule is dependent on the hydration level or lipid composition; this relationship has also been studied using MD simulation: a reduced hydration level can be simulated by reducing the thickness of the solvent phase of the simulation box. For example, in a hydrated bilayer, curcumin has been found to locate below the water–membrane interface and orient parallel to the bilayer normal [164]. Interestingly, curcumin, in bilayers with low hydration, locates outside the bilayer and adopts an orientation parallel to the membrane surface, forming a carpet-like structure. Another MD simulation study determined that the distribution of carprofen derivatives along the bilayer normal is bimodal, with the first density maximum located at the position of the lipid headgroup and a second deeper below the membrane–water interface [165]. When the bilayer is in the liquid disordered phase, the second position has been found to be more frequent than in the liquid-ordered phase. The location of ibuprofen is sensitive to cholesterol [166], thus also to the membrane phase. In the DPPC bilayer with 25 mol% of cholesterol, ibuprofen locates slightly closer to the water–membrane interface in comparison to the case of the pure DMPC bilayer (0.81 and 0.95 nm below the position of the phosphate group, respectively); in the bilayer with 50 mol% of cholesterol, ibuprofen, however, is found below the lipid headgroups, within the hydrophobic bilayer core (regions 3 and 4). 

Extra complexity to the drug molecule–bilayer interaction is added when a lipid membrane with polymers conjugated to a set of the lipid headgroups is considered [167]. For example, lipids functionalized with poly(ethylene–glycol) (PEG) are most frequently used to fabricate so-called stealth liposomes applied as drug carriers in drug delivery [168]; PEG forms a polymer mesh on the top of the membrane surface, the PEG corona covering the liposome, where hydrophobic and amphipathic molecules may locate instead of within the bilayer. Here, they are shielded, by the polymer, from unfavorable contact with the polar solvent (water), in the same fashion as they would be if located within the membrane if the PEG were not present, however, with a reduced free energy penalty to their normal location within the membrane, leading them to locate instead within the PEG corona [144,168,169,170]; this can thus be seen as a potential additional compartment for small molecules.

The importance of lipid affinity and exact location in various membrane compartments has also been demonstrated for antioxidants that protect lipids from peroxidation. Two MD simulation studies have found evidence that that the functionalization of antioxidants with lipophilic groups, incapable of free–radical scavenging, decreases the extent of lipid peroxidation [171,172]. This effect results from the increased concentration of functionalized antioxidants in the hydrocarbon phase (regions 3 and 4). Another study, combining MD simulation with experiments, elucidated the mechanism of the effect of flavonoids on the antioxidant activity of tocopherol and ascorbic acid in lipid bilayers [173]; flavonoids, quercetin in this case, synergistically increase the antioxidant activity of tocopherol and ascorbic acid. Through MD simulation, it was demonstrated that the orientation and location within the lipid bilayer of the three antioxidants differ: tocopherol locates below the lipid headgroups (region 2) and is thus capable of translocating through the bilayer, while ascorbic acid locates among the lipid headgroups (region 1), and quercetin prefers an intermediate position (regions 2 and 3). Moreover, these three antioxidants can form non-covalent complexes: quercetin–tocopherol, quercetin–ascorbic acid, tocopherol–ascorbic acid, and tocopherol–tocopherol. The formation of these complexes was observed in MD simulations and subsequently confirmed through explicit quantum mechanical calculations; the formation of quercetin–tocopherol complexes was then also confirmed experimentally, via fluorescence spectroscopy [173]. This formation of non–covalent complexes of the molecules provides a rational explanation for the synergistic effect of using a mixture of antioxidants: (1) the regeneration of tocopherol (a reaction transforming a tocopherol radical back to its native form) is facilitated and (2) quercetin locates deeper within the hydrocarbon phase when complexed with tocopherol. 

Biomembranes provide a variety of local environments with physicochemical properties that vary considerably, dependent on the lipid composition; this allows for the optimization of membranes for different roles [174,175,176]. Even within single biomembrane domains, the lipid composition and properties may vary [177,178]. Here, MD simulation of drug molecules in different membranes can be used as a tool to investigate how drug molecules will interact with different biomembranes encountered in the body or even different lipid domains. For example, Alves et al. [179] studied the well-known chemotherapy agent doxorubicin in both a cholesterol rich, ordered, environment and in a liquid-disordered cholesterol poor environment using a combination of experimental analysis and MD simulation. They found evidence that the presence of cholesterol reduces the effect on membrane fluidity, thus possibly increasing the density of doxorubicin in cholesterol rich membrane domains, where the efflux P-gP protein is found, thus providing a possible explanation of the observed heightened vulnerability to efflux proteins of doxorubicin. On the contrary, amantadine [180] and chlorzoxazone [181] are less soluble in bilayers containing cholesterol.

Drug molecules can be functionalized to polymers to improve their bioavailability and achieve passive targeting. A common choice is direct covalent conjugation to a PEG polymer; however, other polymers have also been considered [43,182]. Conjugation to a polymer will clearly alter the interaction between drug molecules and lipid membranes; this can be studied through MD simulation. Tetraphenyl–porphyrin is a photosensitizer used in photodynamic therapy for cancer treatment. In MD simulations, we observed that tetraphenyl-porphyrin locates to regions 1 and 2 and orients within the lipid bilayer such that the two hydroxyl groups that it possesses are located at the water–membrane interface; PEGylated tetraphenyl–porphyrin, however, remains in the water phase. This result has been validated through MD simulations performed with its initial position within the membrane; it was seen to leave the membrane as the system equilibrated [183].

**Figure 8:**
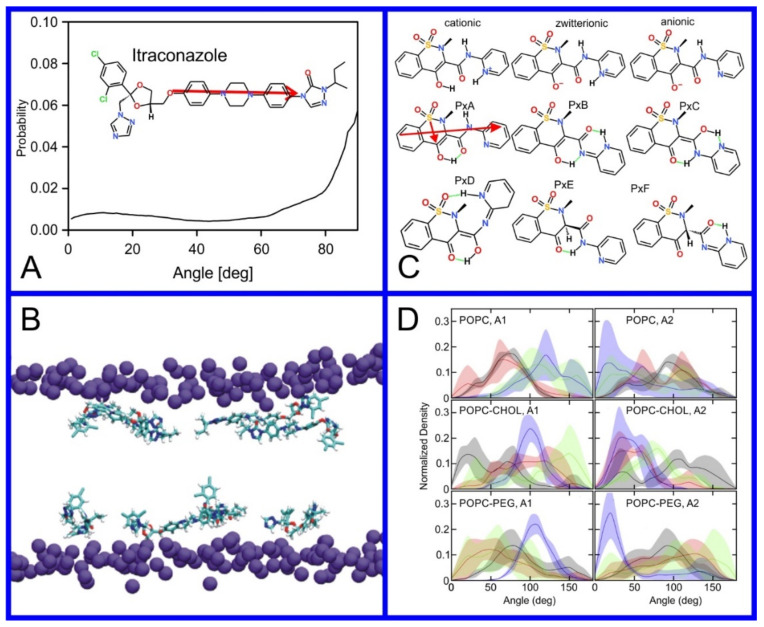
(**A**) Distribution of values of the angle between the vector representing the itraconazole long axis (red arrow at the chemical structure of itraconazole) and the bilayer normal [184]. (**B**) Snapshot showing itraconazole molecules in a lipid bilayer, purple spheres are phosphate groups of the POPC molecules [184]. (**C**) Chemical structures of piroxicam tautomers. (**D**) Distribution of angles between the vector representing the piroxicam long and short axes (red arrows at the chemical structure of PxA) and bilayer normal; PxA—black line, PxE—read line, zwitterionic—green line, cationic—blue line [185].

As we discussed above, for the case of tetraphenyl porphyrin, in addition to location in the lipid bilayer, the orientation of the molecule with respect to the membrane normal will also characterize its behavior. The antifungal drug itraconazole is a rigid and long molecule with weakly polar groups distributed along its backbone (long axis of the molecule); as a result of this particular structure, it adopts an orientation parallel to the membrane surface and locates to the region of the upper segments of the lipid acyl tails (region 3), close to the lipid headgroups (Figure 8) [184]. Due to the rigidity of the itraconazole molecule, its presence can affect the orientation of other molecules in the bilayer. For example, the fluorescent probe 1,6-Diphenyl-1,3,5-hexatriene (DPH) alters its orientation from parallel to the lipid chains to parallel to the itraconazole molecules within the membrane [186]; this change could lead to an incorrect interpretation of steady–state fluorescence anisotropy and fluorescence lifetime measurements. Finally, in bilayers containing cholesterol, itraconazole molecules prefer bilayer regions depleted of cholesterol due to the incompatible orientation of the cholesterol molecules parallel to the membrane normal [187]. For this reason, cholesterol, typically present in liposome formulations used in drug delivery [188,189], is not useful as a component of a vesicle used as a delivery mechanism for itraconazole. Conversely, there are also drug molecules that are sufficiently lipophilic to locate within the membrane that, however, do not adopt a specific orientation when there, i.e., their orientation in the membrane is isotropic. For example, there are many tautomers of the drug molecule piroxicam: six are uncharged (PxA, PxB, PxC, PxD, PxE, and PxF) [190] in addition to zwitterionic, cationic, and anionic tautomers (see Figure 8C). Although, PxA and zwitterionic tautomers are dominant, in vacuum and water solutions respectively, the probability of occurrence of all possible tautomers, when in the environment of the lipid headgroups (regions 2 and 3), is not known; MD simulation studies of four piroxicam tautomers demonstrated that the orientation of the molecules differs significantly between tautomers and, for most cases with the exception of the cationic tautomer, there are no strong preferences for a specific orientation in the bilayer (Figure 8D) [185].

**Table 1:** List of recent studies of drug–membrane interactions.

Application and Target	Drugs and Pharmaceutics
High blood pressure treatment, Angiotensin II AT1 receptor	Losartan [191,192], Candesartan [193]
High blood pressure treatment, β–adrenergic receptors, GPCR	Acebutolol [194], Alprenolol [148,195,196], AS408 [197], Atenolol [148,196,198,199], Carazolol [200], Formoterol [201], Idacaterol and its analogs [201], Metoprolol [196], Nadolol [196], Oxprenolol [194], Pindolol [148,196], Propranolol [194,196,202], Salbutamol [199], Salmeterol [201]
High blood pressure treatment	Amlodipine [198,203], Lisinopril [198], Debrisoquine [132]
Anticancer drug	Tamoxifen [204], Cytarabine [205], 5-Fluorouracil [206,207], Daunorubicin [208,209], β-Lapachone [210], Minerval [211], Miltefosine [212], Tofacitinib [213], Edelfosine [214], Miltefosine [214], Perifosine [214], Camptothecin [215,216], Pirarubicin, Ellipticine [217], Perillyl alcohol [218], Cisplatin [219,220], Doxorubicin [179,217,221], 5-fluorouracil [206]; Previous reviews [222,223], Chlorambucil [199], Camptothecin [224], Ohmline [225]
Photosensitizer used in cancer treatment	Tetra–phenylporphyrin [169,183], Hematoporphyrin [130], 1-BODIPY (6,7-dibromo-2-ethyl-1,3-dimethyl-4,4-difluoro-4-bora-3a,4a-diaza-s-indacene) [226], Indocyanine green [144,170]
Potential anticancer drug	Curcumin [164,227,228,229], Aplysiatoxin [230], Bryostatin [231], Phorbol [231], 12,13-dibutyrate [231], Prostratin [231]
Antibiotics	Imipenem [232], Doripenem [232], Ertapenem [232], Meropenem [232], Ciprofloxacin [233,234], Ciprofloxacin ternary copper complex [235], Daunorubicin [209], Idarubicin [209], Levofloxacin [236,237,238], Clarithromycin [236], Isoniazid N′-acylated derivatives [239], Rifampicin [234,240], Mangostin [241], Trimethoprim [242], Negamycin [243]
Potential antibiotic	Kanamycin A [133], nTZDpa and its derivatives [244], Cholic acid derived amphiphiles [245], γ-terpineol [246], Bithionol [247]
Antimicrobial compound	Chlorhexidine [248,249,250], Triclosan [251], Octenidine [250]
Antiparasitic	Praziquantel [252]
Antiviral drugs	Darunavir [253], Amantadine [254,255,256], Spiro[pyrrolidine-2,2′-adamantane] [254,255], 20,30-dideoxyadenosine (Didanosine) [242], Saffron [257]
Antifungal drug	Itraconazole, [184,186,187,258], Nystatin [259], Amphotericin B [260]
Rheumatoid arthritis	Lapatinib [213]
Nonsteroidal antiinflammatory drugs, inhibitor of cyclooxygenase-1 and -2	Ketoprofen [261,262,263,264], Aspirin [199,229,261,265,266,267,268,269,270,271], Piroxicam [185,261], Ibuprofen [132,166,199,203,265,270,272,273,274,275,276,277], Indomethacin [277], Diclofenac [132,270], Xanthone derivatives (KS1, KS2, KS3) [278], Indomethacin [279], Carane derivatives [147], Carprofen [165], Phenylbutazone [199]
Steroids	Danazol [280], Hydrocortisone [281]
Antiinflammatory drugs	Colchicine [282],
Pain medication	Paracetamol [283,284,285]
Pain medication, opioid receptors	Morphine [132], Fentanyl [132], Fentanyl and its analogues [286], Codeine [287]
Local anesthetics	Benzocaine [288,289,290], KP-23 [147], Dibucaine [291], Lidocaine [289,292,293], Articaine [289], Tetracaine [294,295], Prilocaine [296], Dyclonine, Butamben [290]
General anesthetic	Xenon [124,125], Chloroform [292,297,298,299,300,301], Halothane [146,298,302,303], Isoflurane [297,299,304,305], Phenyl-ethanol [306], Desflurane [305,307], Sevoflurane [305,308], Propofol [305,309,310], Diethyl ether [298,308], Enflurane [298], Ketamine [311]
Antihistamine	Cetirizine [199], Cimetidine [199], Doxylamine [199], Icotidine [199]
Fibrotic skin disorders	p-aminobenzoic acid [132,290]
Statins	Atorvastatin, Cerivastatin, Fluvastatin, Rosuvastatin, Lovastatin, Pravastatin, Simvastatin [312,313,314,315,316,317]
Antidepressant	Amitriptyline [318], Fluoxetine [319,320], Thioridazine [321], Sertraline [199], Bupropion [199], Imipramine [199]
Antipsychotic	Clozapine [318,322], Haloperidol [322], Promazine [199], Chlorpromazine [199], Olanzapine [199], Alprazolam [199],
Neuroleptics	Trifluoperazine, Haloperidol decanoate, Clozapine, Quetiapine, Olanzapine, Aripiprazole, Amisulpride [323]
Alzheimer disease	Pregnanolone sulfate, Pregnanolone glutamate [324], Carbazoles [325]
Anticonvulsant and muscle relaxant	Carbamazepine [326,327,328], Nordazepam [199], Lamotrigine [199], Chlorzoxazone [132]
Cardiac arrhythmias	Dronedarone [312]
P2Y1 antagonist	BPTU [329]
Urea cycle disorders	4-phenylbutyrate
Immunosuppressant	Cyclosporine A and E [330]
Cardiac Ca^2+^ pump inhibitors	CDN1163, CP-154526, Ro 41-0960 [331]
Eye drops components	Cetalkonium chloride, Poloxamer 188 [332]
Vaccine adjuvant	Cobalt porphyrin phospholipid [333], Lipidated nicotine [334]
Other potential drugs	Baicalin [282], Emodin [282], Siramesine [335], HMI and HMI-1a3 [336], Peptide mimicking GM1 [337], AMG3 [338], 1,8-naphthyridine derivatives [339], Protein kinase inhibitors [340], Bile salt export pump inhibitors [341]

Pulmonary surfactants form a complex structure on the inner lung surfaces, the alveoli, including the only monolayer in the human body at the boundary between the pulmonary liquids and air. For drugs designed to treat lung conditions that are delivered through pulmonary administration, e.g., corticosteroid and salbutamol inhalers for the treatment of asthma, this monolayer is the first barrier that they encounter. Pulmonary surfactant monolayers are composed predominately of DPPC (85%), and the remaining components are POPG (11%) and cholesterol (4%). A study by Hu et al., comprised a set of MD simulations of a lung surfactant monolayer models at five different surface tensions, representing various stages of monolayer expansion and compression, demonstrated that the nonsteroidal anti-inflammatory drug ketoprofen changes its location in the monolayer depending on the degree of monolayer compression [263]. In expanded monolayers with a surface area per lipid of 0.9 nm^2^, ketoprofen locates to the water–membrane interface; in the condensed monolayer, with a surface area per lipid of 0.52 nm^2^, ketoprofen locates to the hydrocarbon phase, beneath the headgroups of the monolayer. In other studies that were performed on monolayers mimicking pulmonary surfactants at surface pressures of 55 and 43 mNm^−1^, the antibiotic levofloxacin located to the water–membrane interface with a tendency to locate deeper within the monolayer when the surface pressure was increased [238]. Moreover, levofloxacin aggregated in the hydrocarbon phase of the monolayer.

**Table 2:** List of recent studies of natural compound–membrane interactions.

Function	Compounds
Antioxidants	Quercetin [132,173,282,342,343,344,345,346,347,348], Biochanin [183], Argenteane [132,171], α-Tocopherol [173,349,350,351,352], Ascorbic acid [173], Carbazoles [172], Anthocyanin derivatives (Hemiketal, Chalcone, Pyranoanthocyanin, Aglycone, A4, A5, A7, A-4′7) [353], Trolox [354], PBN [354], Quinones [145,355], Menaquinone [356,357], Lutein [358], Glutathione [359], Flavonoids [360,361,362,363,364,365,366,367,368,369,370], Liponitroxides [371]
Amino acids	L-phenylalanine [372,373,374,375], L-Tyrosine [374], L-Phenylglycine, Phenylacetic acid [374,376], Tryptophan [376,377,378], Glycine [139,284], Glutamate [139], Aginine [379], Alanine [379], 5-aminolevulinic acid and its esters [380], L-dopa [138,199,381,382]
Nucleotides	ATP [383], UMP [384], DNA [385], ADOMET [381]
Sugars and carbohydrates	Trehalose [386], Gastrodin [387], Mannitol [199], 1,3,7-trimethyluric acid [388]
Neurotransmitters	Dopamine [138,389,390,391,392,393,394,395,396,397], Serotonin [138,378,389,396,398,399,400,401,402], Adenosine [138], Melatonin [138,229,378,389,403,404,405], Epinephrine [138], Norepinephrine [138], Trace amines (Tyramine and phenethylamine) [406], Acetylcholine [139], GABA [139], Histamine [138,393,407]
Hormones	Testosterone [132,408], Levothyroxine [409], Resolvins [410], Progesterone [326,327]
Vitamins	D2, D3 [411,412]
Alcohols and product of fermentation	Methanol [274,413,414], Ethanol [149,242,284,387,414,415,416,417,418,419,420], Propanol [414,421], Isopropanol [284], Buthanol [414,417,422], Caprate [423], Glycerol [424], Isopropanol [416], Thymol [425]
Natural polymers	Lignin [426,427,428], Polyphenols [429], Cellulose [430,431], Polysialic acid [432]
Gabaergic ketones	Carvone, Menthone, Pulegone, Dihydrocarvone, Thujone [433]
Taste and aroma	Menthol [342,434], Terpenoids [435], Coumarin [132,284,436,437], Limonene [132], 4-ethylphenol (wine/beer aroma) [406], Tannins (wine) [438], Catechin [282]
Caffeine and its derivatives	Caffeine [132,439], Rosmarinic acid [440,441,442], Caffeic acid [441,443], Chlorogenic acid [441], Paraxanthine [132], Caffeic acid derivatives [444]
Pigments	Violacein [445,446], Marennine [447]
Bile salts	[280,423,448]
Steroids	Betulin [449], Saponins [450,451], Glycyrrhizic acid (saponin) [252,452,453], Withaferin-A and Withanone [454]
Lipids	N-arachidonylglycine and oleoyl-L-carnitine [137], sn-2-arachidonoylglycerol [455], Triolein [456]
Osmolyte in Extremophiles	Trimethylamine-N-oxide [457]
Toxin	Veratridine [458]
Oxidation product	4-hydroxynonenal [459]
Metabolites	5-phenylvaleric acid [203], Ligustrazine [282], Ferulaic acid [282], Imperatorin [282],
Phenolic compounds	Artepillin C [460], Chlorogenic acid and Isochlorogenic acid [461], Proanthocyanidins [462,463], Oleuropein aglycone [464], Other [465,466,467,468,469]

The orientation and location of drug molecules in lipid membranes is clearly only the beginning of our story. Once molecules enter a biomembrane they can do three different things: (1) pass out of the membrane again, possibly on the other side, the membrane merely forming a biological barrier on their way to their eventual destination, (2) act collectively to instigate large scale alteration or even disruption of the membrane structure, or (3) interact with proteins that are associated with the membrane. These three phenomena are all of clear relevance to molecules designed to act as drugs. In the rest of this review article, we will discuss the use of MD simulation to elucidate each of these in the context of drug design.

**Table 3:** List of recent studies of xenobiotic–membrane interactions.

Application	Xenobiotic
Antiseptic	Picloxydine, Octenidine, Miramistin [470], Polyhexamethylene Biguanide [471]
Insecticide	Parathione [132], Fipronil [472], Dibutyl succinate [203]
Former Drugs	d-sotalol, cisapride [473], piracetam (status varies among countries) [474], ORG-12962 [199]
Toxic xenobiotic	Polybrominated-diphenyl-ethers [475], Bisphenol [476], Perfluoroalkyls [477], nitroaromatic explosives (TNT,2A, and 24DA) [478,479,480,481], 1,4-Dioxane [482], Benzo[a]pyrene [483]
Nanomaterials	Graphene [484,485,486,487], Carbon Dots [488], Phosphorene Oxide Nanosheets [489], Gold Nanoparticles [490,491,492], Titanium Dioxide Nanoparticles [493], Generic Nanoparticles (coarse grained) [494], Fullerene [495,496,497,498], Previous reviews [499]
Polymers	Poly(ethyleneoxide)-Poly-(propylene oxide) [500], polyethylenimine [501], Poloxamer [502,503], Pluronics [504,505], poly(ethyleneglycol)-desferrioxamine/gallium [506], PEG functionalized with carbochydrates [507] and peptides [508], poly(2-methyloxazoline) [509], Polyethylenimine and Polylysine [510]
Ionic liquid	Choline-glycine [511], Cholinium-phenylalaninate [512], Imidazolium-IL [bmim][Cl] [513]
Fluorescent labels	[514,515,516,517,518,519,520,521,522,523,524,525,526,527,528,529]
Fragrance	Musk xylene [132]
	2-aminoethoxydiphenyl borate (inhibitor of IP_3_ receptors and TRP channels) [530]
Organic solvents	Pentanol [422], Hexanol [422], Heptanol [422], Acetic acid [149,415,417], Tolune, Phenol [284,417], Styrene [417], Ethylbenzene [417], Benzaldehyde [531], Benzene [149,415,417], Hexane [415]
Other	Lauryl Ether Sulfate [532], Dodecyl Sulfate [533], CyMe4-BTPhen [534], Acetone [420], DMSO [417,420,535], bis-(3-hydroxy-4-pyridinonato) zinc(II) complex [536], calix[4], resorcinarenes [537], dihydropyrimidine analogues [538], Choline carboxylates [539], Synthetic xanthophylls [540], Triton X-100 [541,542], Benzoic acid [284,287,387], Methane [379], Borneol [282,543], Osthole [282,543], Isopulegol [544], Benzylpiperidine [545], Benzimidazole derivatives [546], Aldehydes [547]
Inorganic	Water [149,284,379,417,548,549,550,551,552,553,554,555], Ammonia [274,284,416,417,549], Urea [284,287,417,549], Na^+^ [379,556], Dithionite [550]
Gases	Gases [557], Oxygen [417,557,558], Ozone [558], Carbon dioxide [242,284,416], Propane [284], Fluoromethane [284], Ethylene [415], NO_2_ [558], SO_2_ [558], Butadiene [285], Gas bubbles [559]

## 4. Translocation through the Membrane

**Figure 9:**
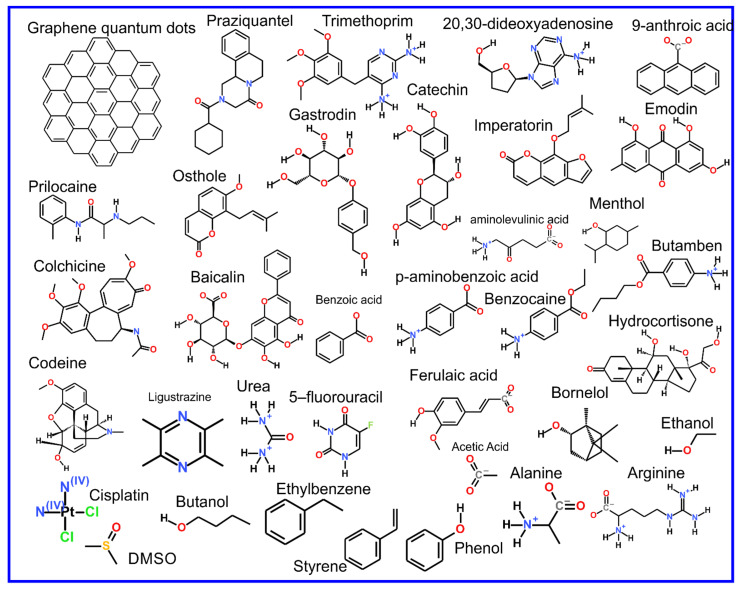
Chemical structure of the chemical compounds introduced in Section 4.

For all drugs, there is a ubiquitous membrane interaction they need to perform in every case: transfection, or translocation, through biomembranes that form biological barriers that need to be crossed in order to reach the target site for their action (Figure 9). This process has been studied intensively using MD simulations; for previous extensive reviews, see references [34,560,561,562,563]. Unsurprisingly, the permeation of small molecules through the lipid bilayer was among the first membrane-related topics studied through MD simulation, e.g., in 2004, Bemporad et al. [149] studied permeation of small organic molecules like benzene or ethanol, and in older studies, Marrink and Berendsen (1994) studied permeation of water [564]. A significant amount of work has been performed to predict the permeability of small molecules through lipid bilayers using various molecular modeling methods and theoretical tools [565,566,567,568,569,570,571,572,573,574]; this includes new computational methodologies, developed specifically for the study of membrane permeability [575,576,577]. A web server and database PerMM is dedicated to gathering experimental and computational data related to small molecule membrane partitioning and translocation [578]. For a recent review of the development of experimental methods used for studying passive diffusion through membranes, see reference [579].

**Figure 10:**
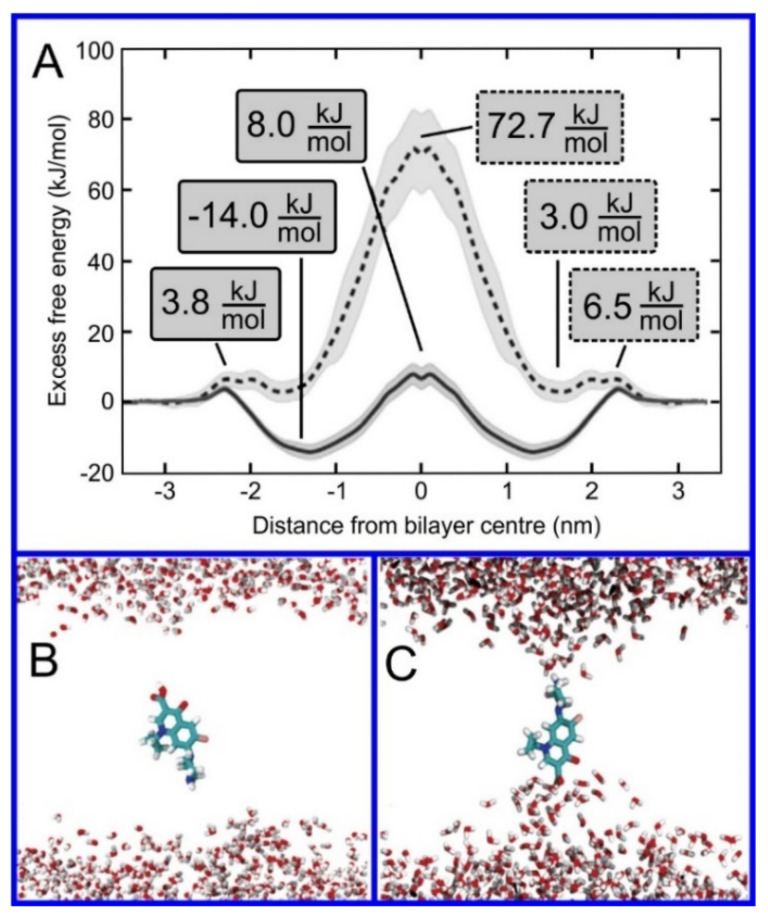
(**A**) Free energy profiles along the bilayer normal of zwitterionic (dashed line) and neutral (full line) ciprofloxacin molecules; the membrane center is at z = 0. Snapshots of ciprofloxacin molecule in the bilayer center: (**B**) uncharged, (**C**) zwitterionic form. Lipids are not shown for clarity. Reproduced with permission from ref. [233].

Regarding the study of drug translocation through the lipid bilayer, the main limitation is the time scale that is possible to reach with MD simulations with all atom resolution; this typically is not sufficient to allow for the observation of the entire translocation process without some form of force bias. Translocation in MD simulations can be observed for tiny molecules, e.g., gases [557] or water [548,552,555] in numbers allowing for the observation of a sufficient number of events to allow for a statistically significant result; for larger molecules, only single cases of translocation can be observed, e.g., [550,580,581,582,583,584,585]. Membrane transfection represents a transition through an energy barrier, as shown in Figure 10; the force biased methods discussed earlier in this review are thus the tools needed to effectively study this. The transition rate will be directly proportional to the height of the energy barrier and force biased methods are an effective means to calculate this. For the case of membrane translocation, the umbrella sampling method [93,586,587] is the most frequent choice to perform this calculation; umbrella sampling calculations show a profile of the potential of mean force (PMF) along selected transition pathways, which, for the case of membrane permeability, is normal to the bilayer surface (Figure 10). The recent review by Lee and Kuczera provides insight into methods used for the calculation of free energies of translocation through lipid bilayers [57].

As discussed previously, the computational cost of an umbrella sampling calculation is relatively high, but through clever tweaks to the algorithm this cost can be reduced; membrane transfection can be seen as a near perfect testbed for the comparison of variants of umbrella sampling and other algorithms for the study of transitions through energy barriers. Nitschke et al., developed a number of shortcuts regarding the calculation of the energy barrier to membrane transfection using umbrella sampling, including decreasing the size of the bilayer, parallel use of multiple solutes, and decreasing the cutoff radius for the Lennard–Jones interactions; this did not significantly affect the quantitative results of the calculations while reducing the computational resources required by up to a factor of 40 [274]. Other methodological studies have determined that adding the so-called flooding potential [92] to the force field allows for improved conformational sampling of the solute before umbrella sampling, thus improving the accuracy of the calculation of the free energy barrier [203]; this was discussed earlier in the paper. A comparison of the four methods used to obtain the free energy profiles (umbrella sampling, replica-exchange umbrella sampling, adaptive biasing force, and multiple-walker adaptive biasing force) for three solutes (urea, benzoic acid, and codeine) found no benefit in implementing the more advanced algorithms, i.e., no improvement in the quantitative accuracy of the results for a given expenditure of computational resources [287]. In another study, three methods were compared: metadynamics, umbrella sampling, and replica-exchange umbrella sampling [379]. Comparisons were performed for six compounds: arginine, sodium ion, side-chain analog of alanine (methane), alanine with neutral termini, zwitterionic alanine, and water. In partial disagreement with the previous result, this study found a significant deviation in the free energy profiles for the case of charged molecules: arginine, sodium ions, and zwitterionic alanine, while for neutral molecules such as methane and zwitterionic alanine, no significant differences were observed [379]. The reason for the discrepancy observed for charged molecules was the slow relaxation of electrostatic interactions between lipid headgroups and the solute. The only method available that allowed for sufficient sampling was replica-exchange umbrella sampling. Recently, Bennett et al., combined MD simulations with ML to study transfer free energies, i.e., free energy difference between two environments [571]. They calculated free energies of transfer from water to cyclohexane for 15,000 small molecules using MD simulations and next used obtained data to train the ML algorithm. The mean absolute error of the obtained prediction in comparison to MD data was only ~4 kJ/mol.

**Figure 11:**
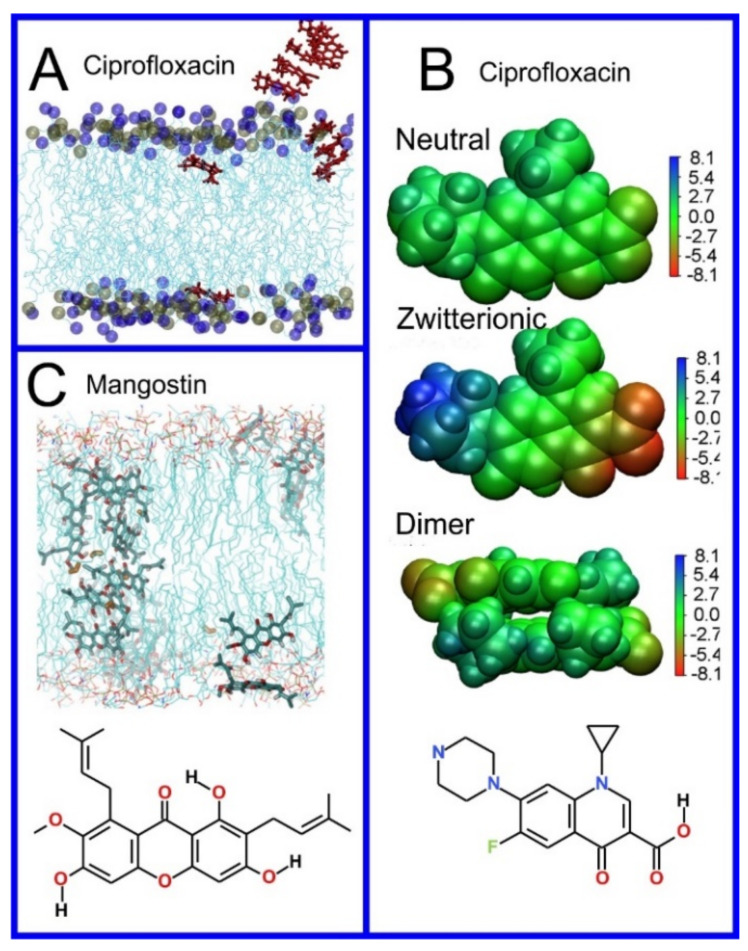
(**A**) Snapshots of MD simulation showing a stack of neutral ciprofloxacin entering the lipid bilayer. (**B**) Electrostatic potential maps at the molecular van der Waals surface in a dielectric continuum corresponding to the water phase calculated using the DFT method and chemical structure of ciprofloxacin. (**C**) Snapshots of MD simulation showing transmembrane arrangement of mangostin molecules and its chemical structure. A and B reproduced with permission from ref. [233]; C reproduced with permission from ref. [241].

A qualitative insight into the mechanism of drug translocation through a lipid bilayer, i.e., following what the molecule actually does in a physical sense as it passes through the membrane, can also be obtained through MD simulation. For example, Chipot and Comer demonstrated subdiffusive behavior in small molecules translocating through the membrane, with a mean squared displacement proportional to time t as $t^{0.7}$ [413] rather than the expected linear relationship. Cramariuc et al., proposed that the translocation of ciprofloxacin, a fluoroquinolone antibiotic, is facilitated through the collective entrance into the membrane of dimers, or even larger column-like stacks of ciprofloxacin molecules (Figure 11A,B) [233]. The column-like stacks of ciprofloxacin molecules have also been observed by Li et al. [343]. Complexed to form dimers, or larger aggregates, negatively and positively charged groups of ciprofloxacin neutralize each other and thus provide an easy avenue for proton translocation; this leads to the collective transformation of all the ciprofloxacin molecules to their uncharged form. Interestingly, in studies of the antibiotic mangostin (Figure 11C), the formation of a transmembrane aggregate of 16 molecules of the drug was observed [241]; MD simulations can find evidence of general trends connecting drug structure to permeability. For example, performing simulations of 49 compounds, Dickson et al., derived a simple correlation between passive permeation and the number of hydrogen bonds between solute and the surrounding molecules, i.e., water and lipids, with a correlation coefficient of 0.63 [588,589].

**Figure 12:**
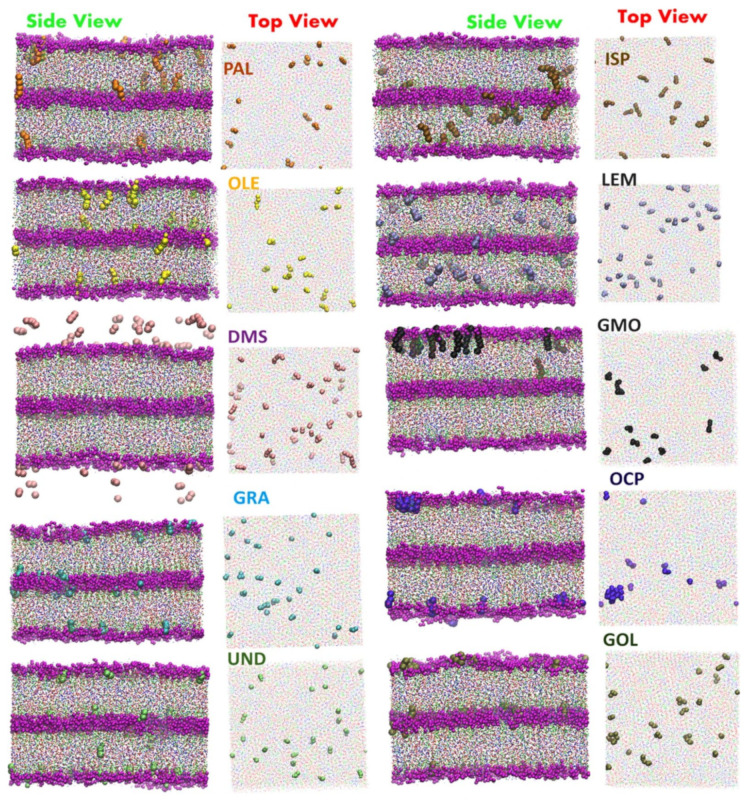
Snapshots of the *stratum corneum* model with selected enhancers: Oleic Acid (OLE), Palmitic Acid (PLA), Geranic Acid (GRA), Undecanoic acid (UND), DMSO (DMS), Geraniol (GOL), Glycerylmonooleate (GMO), Isopropyl palmiate (ISP), Limonene (LEM), and Octyl pyrrolidone (OCP). Reproduced from ref. [604].

The translocation of the drug molecules through lipid bilayers may be facilitated using additional molecules that assist the translocation of the drug through the membrane. Well known examples of such molecules include ionophores facilitating the translocation of ions, e.g., K^+^ [590], K^+^ and Mg^2+^ [591], Cl^−^ [592,593,594] and positively charged doxorubicin [221]. Other examples are so-called molecular umbrellas, capable of transporting hydrophilic cargo through lipid bilayers [595,596,597] or hydrophobic cargo through aqueous solution [598]. In a similar spirit, the use of conjugated antioxidants with antiretroviral drugs has been proposed to increase drug penetration into the central nervous system [599]. Additionally, peptides, including antimicrobial peptides, were proposed as an effective enhancer [600,601]. Recent MD simulations demonstrated that glycyrrhizic acid enhanced the translocation of the antiparasitic drug praziquantel through a lipid bilayer by lowering the free energy barrier associated with the hydrophobic center of the membrane along with a rearrangement of the lipid headgroups [252]. Similarly, studies of menthol, as an enhancer of translocation, found a large decrease in the free energy barrier in the bilayer center for the translocation of quercetin [342]. Additionally, graphene quantum dots have also been found to decrease the free energy barrier against the translocation of doxorubicin and deoxyadenosine [484]. Interestingly, carbon dioxide increases the permeability of ethanol, 20,30-dideoxyadenosine, and trimethoprim through the POPC bilayer [242]. Recently, cyclic peptides have been proposed as potential enhancers of drug permeation through lipid bilayers [602]. The cyclic peptide (Trp-D-Leu)4-Gln-D-Leu has the ability to assemble into tube-like structures in lipid bilayers; this has been demonstrated to possibly be capable of acting as an enhancer for the antitumor drug 5-fluorouracil [206]—the drug is believed to pass the bilayer through the tube created by the peptide as free energy calculations indicate the presence of only a small 5 kJ/mol barrier against such a translocation. In coarse-grained simulations of lidocaine translocation through a lipid bilayer, two enhancers, ethanol and linoleic acid, were shown to have a synergistic effect on lidocaine permeability [603]. Finally, Gupta et al. [604] performed extensive screening of possible enhancers through a massively parallel array of umbrella sampling calculations using the coarse-grained MARTINI model [605,606] to obtain approximate results for the free energy barriers to translocation for each case (Figure 12) [604].

The effect of the lipid composition of the membrane on its permeability to a broad range of molecules has also been studied though MD simulation. The presence of cholesterol, a major component of the cell membrane, is known to reduce the permeability of the membrane to various solutes [607,608,609]; this phenomenon has been examined through MD simulation. For example, in POPC bilayers, the addition of 33 mol% of cholesterol has been found to reduce the permeability of the membrane to 9-anthroic acid and 2’,3’-dideoxyadenosine by a factor of ten; however, the permeability to hydrocortisone is reduced by a remarkable factor of 600 [281]. In a lipid bilayer mimicking the cell membrane, with an asymmetric distribution of phospholipid types, i.e., the formulation of the two leaves of the membrane differed, the permeability was found to be lower by 5–6 orders of magnitude in comparison to that of a pure DOPC bilayer [219]. The permeability of this lipid bilayer was further reduced by an order of magnitude when 33 mol% of cholesterol was added to both leaflets [219]. In cancer cells, the cell membrane asymmetry frequently vanishes; thus, comparison of symmetric and asymmetric models of the cell membrane, are of significant interest. A comparison of the permeability to cisplatin of symmetric and asymmetric bilayers found a decrease in permeability in a membrane designed as a model of a cancer cell membrane by a factor of 11. It has also been shown through MD simulation studies that the addition of DOPE, representing a lipid type that does not form a lamellar structure, to a DOPC bilayer, reduces the permeability regarding small molecules (molecular weight less than 100 Da); however, for larger molecules the effect is the opposite [284]. Simulations of lipid bilayers containing products of lipid oxidations have found a decrease in permeability for the case of oxysterols [550] and tail oxidized phosphatidylcholine [481].

From the standpoint of pharmaceutical research, the most interesting are studies of membranes that form a boundary with an extracellular environment, including bacterial membranes [415,610,611,612,613,614,615,616], the *stratum corneum*, i.e., the most external layer of the skin [617,618,619,620,621,622,623,624,625,626], membranes present in the eyes [557,627,628,629,630,631], and the ocular mucous membrane [632], or lung surfactant monolayers [263,558,633,634,635,636,637,638]. Specifically, MD simulations have been used in studies of enhancer effects on the permeability of the *stratum corneum* (e.g., [544]). For example, studies of the effects of the terpene derivative borneol on the enhancement of *stratum corneum* permeability for osthole [543] and gastrodin, catechin, quercetin, emodin, imperatorin, ligustrazine, ferulaic acid, colchicine, and baicalin found that borneol facilitates drugs permeation via a destabilization of the condensed and ordered arrangement of ceramides and free fatty acids [282]. Another study found evidence that the destruction of the *stratum corneum*, caused by ethanol, leads to the extraction of lipids from the membrane and subsequently the formation of the pore–like structures that allow for benzoic acid to translocate through the membrane [387]. Next, extensive studies, using umbrella sampling methods, of the permeability of the *stratum corneum* for water, oxygen, ethanol, acetic acid, urea, butanol, benzene, dimethyl sulfoxide (DMSO), toluene, phenol, styrene, and ethylbenzene identified the locations of the free energy barriers to transit through the *stratum corneum* for these compounds [417]. The umbrella sampling method has also used been used for studies of the permeation of p–aminobenzoic acid, benzocaine, and butamben through a lipid bilayer in the gel phase composed of ceramide, one of the main components of the *stratum corneum* [290].

In recent studies, Liu et al., calculated the time of entry of 79 drugs into the lipid bilayer, mimicking the lipid composition of the COVID-19 envelope with and without the spike protein and five bilayers mimicking the lipid composition of the: (1) plasma membrane, (2) lysosome, (3) endoplasmic reticulum, (4) Golgi apparatus, and (5) mitochondrial membranes [639]. The set of 79 drugs was selected from the currently approved drugs as potential antiviral therapeutics [640]. These calculations demonstrated that the presence of the spike protein significantly reduces the time required for drugs to enter the lipid bilayer.

Important membrane-based systems that form barriers that, for the treatment of many conditions, drugs must pass, include the blood–brain barrier and the membrane lining the gastrointestinal tract. Intestinal permeability is essential for pharmacokinetics and is widely studied through both experimental and theoretical methods [641,642,643,644]. The blood–brain barrier is another significant intraorganismal barrier considered in treating numerous diseases, e.g., Parkinson’s disease [645,646] and even in screening potential drugs against SARS-CoV-2, capable of infecting brain tissue [647,648]. Steered MD simulations have found a correlation between experimental parameters that describe drug permeability through the blood–brain barrier and both the maximum force needed for pulling molecules through it and the overall non-equilibrium work performed during the pulling simulation [199]. Studies have been performed for 26 compounds in simple DOPC and DOPC–Cholesterol bilayers. In another study, unconstrained MD simulations were performed at elevated temperature to observe the spontaneous translocations of drug molecules through a multicomponent asymmetric lipid bilayer [416]. These studies produced results in agreement with the experimental data. Additionally, propionylated amylose has been used as a carrier for hydrophobic drugs designed to cross the blood–brain barrier. Amylose forms a helical structure that captures hydrophobic drugs and transports them through hydrophilic environments; however, when the drug-loaded amylose helix enters the membrane–water interface (region 1) the drugs are released [649]. Lipid membranes are, however, only one component of the structure of the blood–brain barrier; in fact, the most important elements are tight junctions controlling the entrance to the paracellular compartments. The main proteins forming these junctions belong to the claudin family. Since these junctions are large and complex, relatively few MD simulation studies of them have been performed; one example is a recent study of Claudin-5 [650].

It is commonly assumed that small molecules transform into their uncharged (unionized) form when they translocate through lipid bilayers, due to the prohibitively high free energy barriers for transporting charged species through a membrane (Figure 10). This assumption can be justified by, e.g., QM calculations performed in a polarizable continuum that demonstrates that proton transfer from the NH^3+^ to the COO^−^ group in the zwitterionic drug molecule will occur, so long as the dielectric constant is lower than 20 [233]; thus, molecules become neutral in the lipid headgroup region when the dielectric constant drops from 78.4 (water phase) to 12–18 (water–membrane interface) [651]. In order to understand the translocation process, one should thus perform calculations using both the uncharged and charged forms of the molecule. In recent studies, Yue et al., performed free energy calculations for uncharged and charged states of drugs and compared them with simulations where dynamic protonation was applied [202]. These calculations demonstrated that the free energy profile obtained with a more realistic dynamic protonation approach cannot be modeled as a simple superposition of the two other profiles; free energy barriers do not directly correspond to those observed in the simulation of the uncharged/charged form of the molecule; this assumption is thus an oversimplification.

Another methodological issue related to translocation and partitioning of small molecules into the lipid bilayer is the absence of explicit polarizability in the majority of the force fields commonly used for the simulation of biomolecules, i.e., potential sets used in the model. Jämbeck and Lyubartsev proposed a scheme to calculate free energy profiles through simulations with the partial charges in the potential set derived from QM calculations performed in a polarizable continuum; the calculations were performed twice with the dielectric constant set to 78.4 and 2.04, in order to mimic both water and hexane, respectively [292]. Based on these calculations, the authors define polarization correction terms for calculations of free energy differences between water and the environment of the membrane core.

A combination of atomistic and coarse-grained (CG) simulations have been applied to study the permeability of both planar lipid bilayers and a model of a small liposome with a radius of 10.1 nm, to 5-aminolevulinic acid and its esters [380]. In the first step, the free energy profile of solute translocation through a planar lipid bilayer was calculated for both coarse-grained and atomistic models. These calculations found a qualitative agreement between both models. In the second step, free energy profiles were calculated for the coarse-grained model of a liposome and compared with a coarse-grained model of a flat bilayer, demonstrating significant differences in the free energy barrier against translocation. In another coarse-grained study of liposomes, encapsulation and translocation between the outer and inner liposome leaflets, for the local anesthetic prilocaine, was observed [296]. Coarse-grained simulations have also been successfully used in the study of nanoparticles partitioning into lipid bilayers, e.g., [652,653] or where large-scale screening is performed for hundreds of drugs [654,655,656,657,658]. It should be noted that the MARTINI model was recently reparametrized (MARTINI 3) [659] to fix problems related to the imbalance of interactions between beads of various sizes leading to unrealistically strong interactions, e.g., protein–protein interactions [660,661,662].

## 5. Effect of Drug Molecules on Membrane Properties

### 5.1. Drug Molecules Can Do More in the Membrane than Merely Locate, Orient or Pass Through

**Figure 13:**
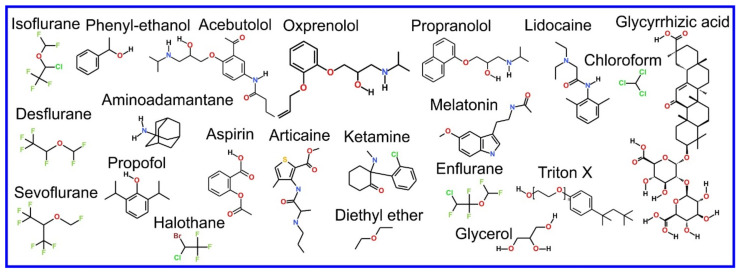
Chemical structure of drugs introduced in Section 5.

In many cases, once drug molecules locate to the membrane, they gather in sufficient numbers to alter the structure of the membrane itself; designing drugs to affect the function of membrane proteins via an indirect modification of the properties of the membrane environment of the protein has been proposed. This mechanism is clearly theoretically possible as it is well known that the function of membrane proteins is modulated by their membrane environment [21,663,664,665,666,667]. Additionally, drugs can have a mode of action that involves solely the disruption of specific membranes; the desired activity of the drug does not, in any fashion, involve affecting the behavior of proteins. Regarding this tantalizing possible drug design strategy, it must, however, be determined whether this kind of affect can be induced with a realistic concentration of the drug molecule; the same question is relevant when alteration of a membrane structure is considered as an unwanted side effect (Figure 13).

This question was first addressed, through experimental studies of the anesthetic isoflurane in physiologically relevant concentrations of 1 and 5 mM in 4 membrane types, using multiple methods including fluorescence microscopy, fluorescence recovery after photobleaching, and patch-clamp measurements, among others. The results of these studies indicated that isoflurane, at this concentration, significantly decreases the extent of lipid ordering [304]. In erythrocyte ghosts, i.e., erythrocytes with their contents removed, the effect of the presence of a 1 mM concentration of isoflurane was found to be more substantial than that of 52.2 mM of ethanol. In another experimental study, the anesthetic phenyl-ethanol was shown to affect the properties of hippocampal membranes at a drug/membrane-volume ratio as low as 0.008% [306]; this indicates that the modification of membrane properties as a mechanism of drug action could actually be feasible. Finally, some drugs accumulate in specific cellular organelles, e.g., cationic amphiphilic drugs accumulate in lysosomes, organelles with an internal pH of ~4–5, perturbing the lysosomal membrane [668,669], thus decreasing cellular viability [670]. Among these drugs are numerous psychotropic drugs, which have shown potency as anticancer therapeutics [671,672].

From what has been discussed so far in this review paper, regarding the use of MD simulation to study the behavior of drug molecules in lipid membranes, it should be clear that MD simulation can also act as a tool to study the effect on membrane properties of drug molecules that have entered the membrane; MD can model a small microcosm of the large scale global structural changes being made to the membrane. Through MD, it is also possible to measure changes to global properties of the membrane due to the presence of specific new foreign molecules; how this is determined through analysis of the MD trajectory is described in the following section.

### 5.2. Relevant Aside: Membrane Properties That Can Be Measured in MD Simulations

**Figure 14:**
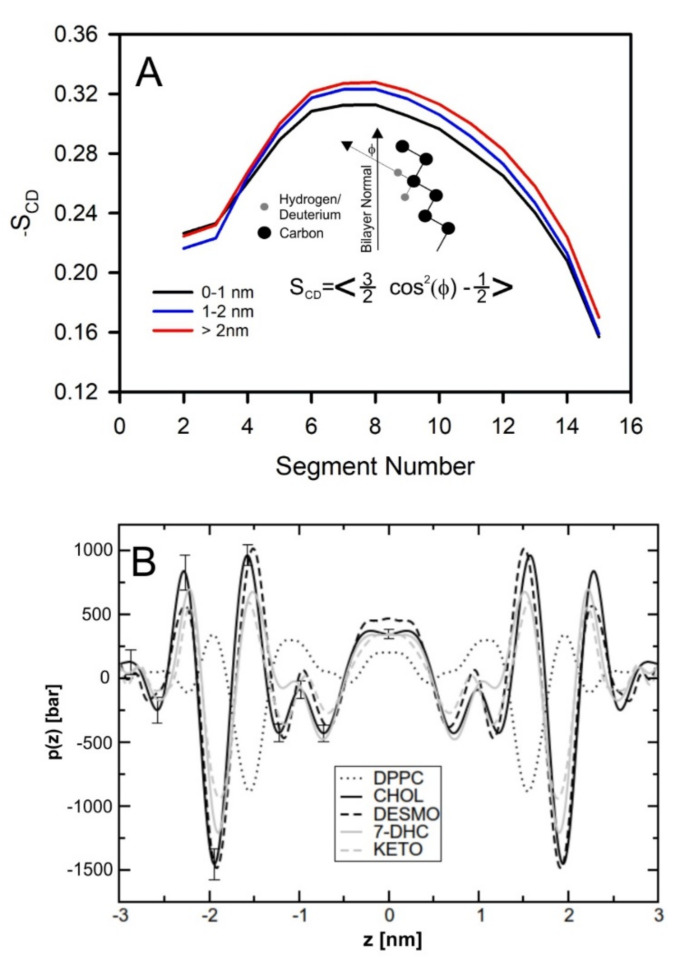
(**A**) Profile of the order parameter S_CD_ for lipids located in three zones: distances 0–1, 1–2, >2 nm from a drug molecule (reproduced with permission from ref. [187]). (**B**) Profile of the lateral pressure in bilayers composed of DPPC, DPPC and cholesterol and DPPC and other steroids (reproduced with permission from ref. [680]).

When studying the effect that drug molecules can have on lipid membranes, MD simulations can provide insight into numerous global structural, elastic, and dynamical properties of lipid bilayers [673,674]; however, in the context of drug–membrane interactions, one usually only calculates a few specific parameters. The results for the surface area per lipid molecule and membrane thickness provide information regarding the lipid bilayer size. The surface area per lipid molecule is easily calculated from MD simulations by dividing the simulation box size in the membrane plane by the number of lipids in a single leaflet of the bilayer. There is no unique definition of bilayer thickness; however, thickness can be estimated from the so-called P–P distance, the distance between the average positions along the membrane normal of the phosphorus atoms of the phosphate groups in opposite leaflets. Both of these parameters, the area per lipid and P–P distance, can be obtained from both X-ray and neutron scattering experiments [675,676,677]. Order parameters can be defined and calculated to describe the extent of ordering in the hydrocarbon chains; the most frequently used is the S_CD_ deuterium order parameter (Figure 14) that can also be measured experimentally through NMR spectroscopy [678,679]. Thickness and surface area are both global parameters, i.e., they represent the large-scale structure of the membrane; thus, their values will usually not be significantly affected by the presence of drug molecules at low concentration. Nevertheless, through MD simulations, these parameters can be calculated for the local region of the membrane around a specific drug molecule, i.e., by looking at a tiny microcosm of the membrane we can study the effect of a high concentration of many drug molecules entering the membrane.

One more useful parameter is the profile of the lateral pressure (pressure in the direction parallel to the membrane surface) along the bilayer normal [680,681]. It has been hypothesized by Cantor (1997) [682,683,684] that changes in the lateral pressure profile of the bilayer may affect the structure of membrane proteins, affecting their functionality. The local pressure inside a lipid bilayer can reach a value of as high as 1000 bar (Figure 14); it is thus clearly plausible that the lateral pressure from the membrane can affect protein structure. Finally, MD simulations can provide information that describes the properties of the water–membrane interface. For example, increased membrane hydration due to the presence of nonsteroidal anti-inflammatory drugs has been suggested as a possible cause of some of the side effects of these drugs [261,278]. Moreover, it has been shown that changes in the orientation of the lipid headgroups, involved in signaling, can affect the binding behavior of peripheral membrane proteins [685,686]; the design of drugs to alter the orientation of lipid headgroups can thus be seen as a drug design strategy to alter their signaling and thereby the related metabolic pathways.

### 5.3. Unwanted Side Effects of Drugs Due to Their Alteration of Membrane Properties

While the possibility of drugs designed to affect membrane properties is indeed an attainable goal, where MD simulation has been brought to bear as a design tool, a more common issue remains the undesired effects that drug molecules can have on the biomembranes they encounter, i.e., drug toxicity (aka side effects); MD simulation has also played a role in elucidating such phenomena. The most frequent effect that drugs have been found to have on biomembranes is a local decrease of the extent of ordering in the acyl tails of the lipids. Examples of such drugs include, e.g., acebutolol, oxprenolol, propranolol [194], and aminoadamantane [255]. A less frequent effect is an increase in the extent of lipid ordering; for example, the drug itraconazole increases the ordering of the acyl tails in the upper part of the chain, close to the water–membrane interface (regions 2 and 3) [186].

**Figure 15:**
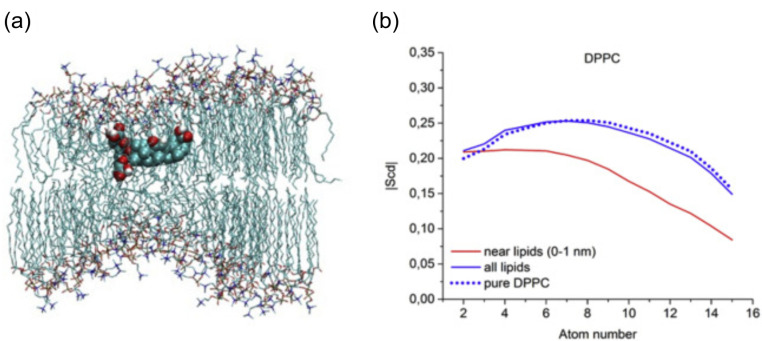
Snapshot of a lipid bilayer with a single glycyrrhizic acid molecule (**a**), and profiles of order parameter for lipids near and far from glycyrrhizic acid molecule (**b**). Reproduced with permission from ref. [453].

In most cases, for example, the aforementioned itraconazole, the mechanism of action of the drug is related to the inhibition of enzymes, channels, or receptors rather than their effect on a specific lipid membrane; alterations of the membrane properties caused by the drug are thus not relevant or even possibly an unwanted side effect. For example, glycyrrhizic acid is a saponin found in licorice root that is proposed as a potential component of drug delivery formulations, due to its ability to form complexes with a wide range of hydrophobic molecules. Using MD simulations, it has been demonstrated that glycyrrhizic acid locates inside the lipid bilayer (region 1 and 2) and significantly decreases the extent of lipid tail ordering, even at low concentration (Figure 15) [452,453]; this phenomenon could limit the pharmaceutical applications of this compound. Moreover, the gastrointestinal toxicity that has been observed in many nonsteroidal anti-inflammatory drugs, designed primarily as inhibitors of prostaglandin–endoperoxide synthase, is, at least in part, the result of the effect the drug has on membranes [687,688,689]. Furthermore, the cardiotoxicity of nonsteroidal anti-inflammatory drugs is in part induced by drug–lipid interactions [690]. In addition to demonstrating that certain molecules have unwanted effects on lipid membranes, as described above, MD simulation has found evidence of innocence on the part of several drug molecules; for example, lipid-like potential drugs N-arachidonylglycine and oleoyl-L-carnitine have been shown not to affect membrane properties [137].

### 5.4. Drug Membrane Interaction Can Play a Role in the Mechanism of Drug Action—Case of Anesthetics

Now, we address the possibility that drugs could influence the membrane structure in a fashion that is desirable, i.e., an element of a drug’s mechanism of action could be a direct alteration of the membrane’s properties; MD simulation can be used in the same fashion to elucidate such positive effects in addition to the negative effects described in the previous section. The very first instance of the proposal of a mechanism of drug action that solely involved an interaction with the lipid membrane, rather than the active site of a protein, was the case of the theorized mechanism of action of general anesthetics. This hypothesis originates from the correlation between the efficacy of different anesthetics and their respective oil–water partition coefficients (the Meyer–Overton correlation [691,692]); it was later found that their efficacy has an even stronger correlation with their membrane–water partition coefficients [693]. Thus, unsurprisingly, several MD simulation studies of the interaction between general anesthetics and lipids have been undertaken [694]. For example, results obtained from MD simulations of chloroform in DOPC and DSPC–cholesterol bilayers, representing liquid disordered and liquid-ordered phases respectively, have demonstrated a difference in the fashion with which the addition of chloroform affects their properties [695]. In the DOPC bilayer, chloroform induced a small increase in the extent of tail ordering, while, in the DSPC–cholesterol bilayer, it was seen to have the opposite effect, inducing a pronounced reduction in the extent of tail ordering; it is hypothesized that the preference for chloroform to interact with flexible acyl chains and not with rigid cholesterol molecules leads to this effect. As a result, the number of direct cholesterol–cholesterol contacts increased while the number of cholesterol–DSPC contacts decreased.

In MD simulation studies, four anesthetics, desflurane, isoflurane, sevoflurane, and propofol, were shown to have negligible influence on bilayer properties when present in a POPC bilayer [305]. Additionally, chloroform, halothane, diethyl ether, and enflurane in a DPPC membrane, at a temperature of 310 K, were shown to have a negligible effect on the extent of acyl tail ordering, with the exception of the case of diethyl ether, where a small increase in the extent of lipid ordering was observed [298]. The presence of diethyl ether and sevoflurane in the membrane leads to a decrease in the extent of ordering of the acyl tails for the case of both DPPC and PSM bilayers; this effect is more pronounced for the case of bilayers that contain ~50 mol% of cholesterol [308]. Interestingly, evidence has been found that aspirin, one of the most frequently used analgesics, can also disrupt liquid-ordered domains or prevent their formation altogether [271]. The results of MD simulations have indicated that aspirin molecules affect the structure of the acyl tails of the lipids up to a depth of 1 nm into the bilayer with 30 mol% of cholesterol; coherent inelastic neutron scattering experiments have demonstrated liquid-ordered domain disruption, i.e., transition from the liquid ordered to the liquid disordered phase [266]. The local anesthetics lidocaine and articaine have also been found to decrease the extent of ordering of the acyl tails when in their charged (ionized) form; the results, however, indicate that, in their neutral form, their effect on the membrane structure is negligible [289].

Several experimental studies have been carried out that support the conclusions of the above MD simulations. For example, it has been demonstrated that the addition of chloroform loosens the structure of membranes in both gel and liquid-ordered phases [696]. Recent studies performed using multiple experimental techniques (Super–Resolution Microscopy dSTORM, Patch–Clamp, Fluorescence Resonance Energy Transfer) have demonstrated that both chloroform and isoflurane disrupt lipid domains (rafts) that contain the ganglioside GM1 [299]. Another anesthetic, propofol, at concentrations in the range 1 μM to 5 μM, has also been found to destabilize nanodomains in cellular membranes via Binned Imaging Fluorescence Correlation Spectroscopy [309]. Thus, the significant perturbations of the structure of bilayers in the liquid ordered phase, observed in MD simulation studies [271,308], are in agreement with experimental observations.

The aforementioned studies mainly focused on the effect of anesthetic molecules on the properties of the lipids related to the ordering behavior of the hydrocarbon chains of the lipids, i.e., order parameter, phase behavior, bilayer thickness, and surface area per lipid; this is, however, not the entire story. In 1997, Cantor proposed that general anesthesia is related to an alteration of the lateral pressure profile [683], a parameter not easily measured experimentally but relatively easy to calculate from MD simulations. An MD simulation study of ketamine, in a POPC bilayer, found evidence that the presence of the drug molecule could possibly give rise to a significant alteration to the lateral pressure profile with only a very limited alteration to the membrane structure, e.g., the global properties of the membrane discussed previously: membrane thickness, surface area, or the extent of ordering of the acyl chains [311]. Another MD simulation demonstrated that two other anesthetics, diethyl ether and sevoflurane in (1) DPPC, (2) DPPC–cholesterol 50 mol% (3) PSM, and (4) PSM–cholesterol 50 mol% bilayers, were found to significantly affect the lateral pressure profile [308]. It has also been demonstrated, through MD simulation, that the anesthetic chloroform (CHCl_3_) and the relatively similar molecule carbon tetrachloride (CCl_4_), a non–anesthetic, affect the membranes in strikingly different fashions, as measured by the effect on the lateral pressure and electrostatic potential profiles of the presence of one or the other of these two molecules in the membrane [300]. Experimental studies have also been carried out that provide evidence of the activation of transmembrane proteins as a result of alterations to the lipid composition, e.g., using the mild detergent Triton X-100 [541,542], the relationship between lateral pressure profile and lipid composition was investigated both experimentally [697] and through MD simulation [698]. A few MD simulation studies found specific effects due to direct lipid interaction on protein behavior e.g., [699,700,701,702,703,704,705]; in most cases, the cholesterol molecules in the membrane were, however, most frequently shown to play the role of modulating the behavior of membrane proteins [706,707,708,709,710].

The entry of certain molecules into the biomembrane can affect its properties in a more complex fashion than just altering the aforementioned parameters: it can affect the extent to which undulations are present in the membrane structure. In MD simulation studies of lipid bilayers using the coarse-grained MARTINI potential, it was found that the addition of chloroform can decrease the degree to which undulations are present in a membrane that contains ordered and disordered domains [301]. In the bilayer free of the anesthetic, the ordered domains in opposite leaflets were not registered, i.e., they were not across from one another. In this case, the bilayer was characterized by clearly visible undulations; the addition of chloroform led to the rearrangement of the bilayer, and ordered domains became registered and the bilayer thus became flat.

Although the above discussed computational and experimental studies demonstrate the possible membrane–mediated mechanisms of action of molecules designed as general anesthetics, namely the disruption of ordered lipid domains, such drug–membrane interactions cannot explain the sensitivity of the potency of anesthetic molecules to drug stereochemistry or single point mutations on the proteins involved in anesthesia [711]. Moreover, binding sites for anesthetics have been found from crystal structures of the membrane proteins [712,713,714], docking calculations [715], and MD simulation [307]. For these reasons the membrane mediated mechanism of anesthesia is frequently questioned [711], however, the above discussed studies provide clear evidence of anesthetic induced effects on membrane properties; additionally, evidence that the properties of the membrane influence protein functionality exist. Thus, a membrane-associated mechanism of action for general anesthetics cannot be discarded as one of the mechanisms involved in what is, admittedly, a complex process. Nevertheless, the interpretation of the Meyer–Overton correlation as an indication of a membrane-mediated mechanism is incorrect; we return to this issue later, in Section 6.2.1; this point will become clearer to the reader when they have read this section.

### 5.5. Can Drugs Prevent Amyloid Formation via the Modification of Membrane Properties?

The formation of amyloid plaques deposited on the extracellular membranes of neurons is one of the primary features of Alzheimer’s disease [716]. This phenomenon is affected by specific lipids including cholesterol, sphingomyelin, and gangliosides [717,718]: lipid types typically present in the outer leaflet of the cell membrane. Amyloid fibers at a membrane surface have thus been frequently studied using MD simulations, e.g., [719,720,721,722,723,724,725,726,727,728,729,730], however, a surprisingly small fraction of this work has been performed in the context of drug design [731,732]. Khondker et al., proposed designing drugs with a mode of action that involved modulating membrane properties to affect the aggregation of amyloid-β25-35 at the bilayer surface [229]. They wished to study the modulation of the membrane thickness, ordering, and other properties, as possible modes of drug action; thus, they selected three molecules to study: curcumin, acetylsalicylic acid, and melatonin. Using MD simulations, they demonstrated that curcumin decreases membrane thickness, decreasing the extent of membrane ordering and rigidity; however, acetylsalicylic acid increases membrane thickness, thereby increasing membrane ordering and rigidity while melatonin does not affect the membrane properties at all; it was thus verified that these three molecules represent the needed set of examples of molecules that affect membranes in different ways. It was then determined experimentally that the decrease in the volume fraction of the cross-β sheets was ~70% for the case of curcumin in the membrane; evidence was thus found that decreasing membrane thickness in the vicinity of the amyloid-β25-35 molecules could possibly be a mechanism of drug action for the treatment of Alzheimer’s disease.

### 5.6. A Clear Case of Drug Membrane Interaction as Mechanism of Action—Antimicrobial Agents

Antimicrobial agents represent a clear case of an established therapy where the global alteration of the properties of specific membranes is the clear mechanism of drug action. In general, biomembranes have a high degree of stability under physiological conditions and possess the capacity to adapt to extreme changes in their environment via changes in the lipid composition, e.g., [733,734,735,736]; evolution has provided biomembranes with several tools to preserve themselves over a wide range of conditions. They are, however, not completely invulnerable: numerous chemical agents and certain external conditions can disrupt their structure. The lipid bilayer structure emerges from weak, nonspecific, interactions and, additionally, the structure of biomembranes is formed by the cell, in driven processes and can include lipids that do not spontaneously form bilayers; it is thus unsurprising that they have vulnerabilities to several sources of disruption. The structure of a lipid bilayer can be compromised through the formation of pores that perforate the membrane, due to the presence specific molecules or physical stimuli, e.g., electric field [737], ultrasound [738,739], and charge imbalance [740,741]. Finally, a lipid bilayer can be dissolved completely at the molecular level by, e.g., detergents [742,743] or organic solvents [744].

Procaryotic (bacterial) and eurkaryotic (animal, i.e., patient) membranes differ substantially regarding the lipids of which they are composed. The extracellular leaflet of a eukaryotic cell membrane is comprised of cholesterol, saturated phospholipids, and sphingolipids, while procaryotic membranes contain mainly phosphatidylethanolamines, phosphatidylglycerols, and cardiolipin. This substantial difference, combined with the fact that some molecules can disrupt a cell membrane catastrophically enough to lead to cell death, opens the door to a tantalizing opportunity: the design of molecules that severely disrupt the structure of bacterial membranes while leaving eukaryotic membranes, i.e., the membranes of the cells of the patient being treated, relatively intact: they act as an antibiotic. The bacterial membrane is, however, not the only possible target for peptides designed to destroy specific lipid membranes. A recent development is the emergence of a second important target: cancer cells. The overall lipid composition of the cancer cell resembles that of normal cells, however, the asymmetric distribution of lipid classes between the inner and outer leaflets, normally found in eukaryotic cell membranes [745], is lost; they instead display a substantial concentration of phosphatidylserine in the outer leaflet. The selective disruption of lipid membranes rich in phosphatidylserine becomes a possible mode of action for cancer therapy: anticancer peptides [746,747] or drugs affecting the membranes of cancer cells [748].

Peptides that selectively compromise bacterial but not eukaryotic (host/patient) membranes, are a large and diverse group of potential drugs with a long history of research [749]. Currently, the term “antimicrobial peptides” is used as an umbrella term for peptides that display a wide range of different activities against different pathogens. For example, the APD3 database (2016) of natural antimicrobial peptides includes 2169 antibacterial, 172 antiviral, 961 antifungal, 185 anticancer, 307 hemolytic, 80 antiparasitic, 11 spermicidal, 27 insecticidal, and four anti-protist peptides [750]. The current number of antimicrobial peptide sequences deposited in “DBAASP v3: the database of antimicrobial/cytotoxic peptides” is 16,633 (01.02.2021); this number includes over 12,000 synthetic peptides [751]. Interestingly, over 3200 peptides have been the subject of MD simulations. Finally, dbAMP database contains over 12,000 entries [752]. There are at least six other databases dedicated to antimicrobial peptides [753,754,755,756,757,758] (for review, see [759,760]). Surprisingly, the number of clinical studies of antimicrobial peptides is, as of yet, relatively low (only 76) and the majority of these have ended in failure [761]; so far, only seven peptides have been approved for clinical use by the FDA [762]. However, the literature concerning antimicrobial peptides is extensive; prior comprehensive reviews of various aspects of antimicrobial and anticancer peptides have been published [763,764,765,766,767,768,769,770,771,772,773,774,775,776,777,778,779,780,781,782,783,784,785], including reviews focused specifically on computational studies [46,47,48,49,50,51,52,53,54]; we provide only a brief overview of this topic. Table 4 includes all antimicrobial peptides discussed in this paragraph.

Molecular dynamics simulations are frequently used to provide information regarding (1) peptide conformation (e.g., % of helicity) [786,787,788,789,790,791,792,793,794,795,796,797,798], (2) peptide location (at the membrane interface or inserted into the bilayer core), and (3) orientation in the membrane [783,787,788,789,790,792,793,794,796,799,800,801,802,803,804,805,806]. As we stated previously, simulations can also provide information regarding the effects of the peptides on membrane properties, including (1) the extent of ordering in the acyl tails, (2) the overall membrane thickness, and (3) curvature [793,794,799,800,807] as well as the formation of membrane defects [794]. MD simulations and other computational methods, combined with complementary experimental methods, can be used to design more effective antimicrobial [783,798,808,809,810,811] and anticancer peptides [747,789,812,813,814]. For designing new peptides, an understanding of the interactions between peptides and lipids is of particular interest. The results of MD simulations can provide explicit information concerning, for example, (1) hydrogen bonds, (2) hydrophobic contacts, and (3) electrostatic and Van der Waals interaction energies [792,793,794,799,800,815]. For example, studies of pardaxin in the membrane have demonstrated the importance of cationic residues and phenylalanine residues on peptide association with lipids [806]. Finally, MD simulations allow for the study of the effect of chemical modification on the peptides (e.g., amidation) [787] and the effect of helical kink on peptide insertion into the bilayer [816,817].

The above discussed studies considered only peptides statically located at a membrane; the main mechanism through which antimicrobial peptides kill cells is, however, the formation of transmembrane pores. Simulating pore formation is computationally expensive; thus in a few older studies, to avoid long simulations, antimicrobial peptides were initially placed in the lipid bilayer core [818,819,820] or simulations were performed using the coarse-grained MARTINI model [821,822,823,824,825]. A few studies set out to elucidate the entire process of pore formation; however, the protocol of the MD simulations was altered e.g., by running simulations at an artificially high temperature or through a biased selection of the initial configuration. For example, Sun et al., modeled the formation of transmembrane pores, created by melittin, by initializing the simulated membrane with artificially created defects already in place [826,827]. The N-terminus of the peptide stabilized these defects, resulting in their transformation into small pores following the recruitment of two additional peptides. Wang et al., performed MD simulation at temperatures in the range 80–120 °C; this allowed for the observation of pore formation by the peptide maculatin [828]. Interestingly, they observed the formation of every variety of possible oligomers, from dimers to octamers. In simulations of the antimicrobial peptide pleurocidin, using a model with all atom resolution, the formation of a small pore composed of two helices was observed; however, in much longer coarse-grained simulations a larger number of helices were seen to be recruited to form a single pore [829].

Unbiased MD simulations of transmembrane pore formation were used to design a new 14 residue long peptide, LDKL, which was then modified to include input from the analysis of known antimicrobial peptides [830]. The final peptide, LDKA, contained only four amino acid types: (1) leucine, (2) aspartic acid, (3) lysine, and (4) alanine. Large pores formed by the LDKA peptide consisted of two overlapping pores formed separately in both membrane monolayers and occurred at low peptide/lipid ratios (1:1000). The ability of the peptide to kill both Gram-positive and Gram-negative bacteria without harming erythrocytes has been confirmed experimentally [830].

The results of MD simulations of magainin 2 in its native form and its covalently bound dimer, provided evidence for the formation of a disordered toroidal pore. The concentration of the peptide necessary for pore formation was substantially lower for the dimeric than the native form of magainin [831].

**Figure 16:**
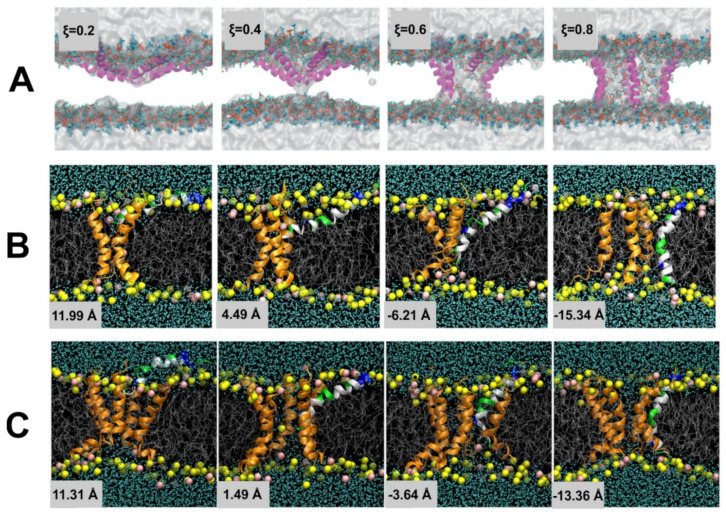
(**A**) Snapshots of stages of pore formation along the collective variable ξ. Reproduced with permission from ref [832]. Snapshot demonstrating the insertion of a single helix into the membrane (colored by residue polarity) where pores formed of (**B**) 3 and (**C**) 5 helices are present. Reproduced with permission from ref [833].

Calculations of the free energy of collective pore formation using umbrella sampling (Figure 16A) found that six melittin molecules can stabilize a pore; formation is associated with a small free energy (activation) barrier of ~10 kJ/mol. For seven peptide molecules, the barrier is absent; however, after pore formation, one molecule was seen to be diffusing away from the pore. When the number of melittin molecules is lower than six, the free energy barrier becomes significant, but five peptide molecules were also able to form a stable pore [832]. In another study, the free energy for the insertion of a single melittin molecule into a bilayer containing 0 to 6 peptide molecules was calculated (Figure 16B,C), a pore composed of three or more peptide molecules was seen to form [833]. The free energy barrier is reduced when a larger number of peptides is present in the bilayer and almost disappears, i.e., is close to 0 kJ/mol, when six peptides are present in the membrane. Atomistic MD simulation studies have also found that synthetic polycations [834], itraconazole [258], and DMSO [258] induce a decrease in the free energy barrier against pore nucleation.

It is worth noting that the results of calculations of the free energy of pore formation are strongly dependent on the force field used [258,835]; other methodological issues related to the simulation of membrane active peptides are (1) the size of the simulation box, (2) electrostatic interaction treatment, (3) initial conditions, and (4) the simulation protocol used [836].

Peptoids, N-substituted glycine oligomers, are a possible alternative class of molecules to antimicrobial peptides with the advantage of increased flexibility in functionalization via the presence of an amide bond [837]. Peptoids have not been studied with theoretical methods in the context of their membrane activity, but rather in the context of their applications in nanotechnology, e.g., the study by Jin et al., in 2016 [838]. Recently, a coarse-grained force-field created within the MARTINI framework [605] for peptoids was developed [839]. Nevertheless, the interactions between peptoids and membranes have been investigated experimentally [840]. These studies have found that antimicrobial peptoids increase the permeability of bacterial membranes; this effect is increased via cyclization [841]. Moreover, peptoids have recently been found to be potent antiviral agents and have been successfully tested for activity against the SARS-CoV-2 virus [842].

Another class of drug molecules derived from peptides that has been proposed for use as an antimicrobial agent are the β^2,2^-amino acid derivatives. In a simulation study carried out by Koivuniemi et al., MD simulations of these derivatives interacting with both prokaryotic and eukaryotic model membranes found evidence of important differences in their behavior in the two membrane types [843]. Additionally, antimicrobial peptides can have their desired properties enhanced through the introduction of non–natural amino acids, e.g., the azoALY peptide derived from ALY (full sequence: ALYLAIRKR) by functionalization of the sole tyrosine residue present with an azobenzene group [844]. Both MD simulations and experimental studies have found evidence that this modification results in a peptide with an enhanced interaction with lipid bilayers. Finally, peptides conjugated with dendrimers have been proposed as a novel form of potential antimicrobial compound; MD simulations have demonstrated the presence of strong interactions between these molecules and bilayers composed of POPG [802].

In the above discussed studies, the bacterial membranes were modeled as simple mixtures of either PC or PE with PG lipids, in spite of the fact that lipopolysaccharides (LPS) are known to be present in bacterial membranes; aspects of the membrane structure, behavior, and interactions with other molecules resulting from the presence of LPS in the membrane were not explored. The reason for this is that when LPS molecules are included in the membranes, the size of the system and timescale needed for system equilibration becomes too large for the system to be tractable for MD simulation with a model with all atom resolution. Theoretical studies have, however, been carried out that indicate LPS plays a protective role against antibacterial peptides, e.g., magainin [845].

Antimicrobial peptides are not the only group of chemical compounds capable of selectively disrupting the bacterial membrane; for example, recent studies of potential antibiotics kanamycin A [133] and bithionol [247], provide evidence that these drugs have an affinity towards model membranes that mimic the bacterial membrane and either do not interact with, or only weakly affect, model membranes designed to mimic eukaryotic membranes. Another set of potential antimicrobial compounds were synthetized on the basis of cholic acid esterified with three glycine and an aliphatic alcohol of length 1, 6, or 12 carbons; MD simulations found evidence of the presence of strong interactions between these compounds and bilayers composed of lipopolysaccharides from Gramm–negative bacteria [846]. The strongest effects on bilayer properties were observed for the compound with the six-carbon long alcohol. Membrane perturbation by antibiotics (e.g., fluoroquinolones and aminoglycosides) was proposed as an additional bactericidal mechanism for these drugs [847]; recent studies of aminoglycosides, macrolides, and fluoroquinolones determined that these antibiotics have a disordering effect on bacterial membranes; however, they found no evidence of large-scale membrane disruption or pore formation [848]. Interestingly, colchicine, a drug frequently used in chemotherapy, is capable of inducing pore formation in lipid bilayers [849].

### 5.7. Other Effects on Lipid Layers—Pulmonary Surfactants and Indirect Effect on Membrane Proteins

Drug molecules have also been shown to affect the properties of lipid monolayers, e.g., disrupting lung surfactants [264]. To condense pulmonary surfactant monolayers doped with ketoprofen, higher surface pressures were necessary in comparison to the case of the pure monolayer [263]. The difference was found to increase with decreasing surface area per lipid. This observation was in qualitative agreement with data obtained from Langmuir–Blodgett monolayer experiments. A similar result was obtained for pulmonary surfactant monolayers doped with levofloxacin [238]. The increase was more significant when the model was simulated with an elevated value for surface pressure. Additionally, the presence of levofloxacin in the membrane had a disordering effect on the acyl tails; again, the effect was more substantial for the model simulated with a higher surface pressure. Glycerol is often used in the formulation of pulmonary drugs and the liquid medium in electronic cigarettes. It has been demonstrated, both experimentally and through MD simulation, that glycerol affects lipid monolayers and bilayers; its presence leads to significant monolayer expansion, even at the low *w*/*w* concentration level of 1% [424].

**Table 4:** Antimicrobial peptides.

Peptide	Source	Refs.
Alamethicin	Fungus, Trichoderma viride	[818]
Aurein	Frog, Litoria aurea	[787,791]
azoALY	Synthetic with non–natural amino acids	[844]
Bombinins	Toad, Bombina variegata	[783,850,851]
Cathelicidins	Innate immunological system	[792,808,852]
Clavanin A	Tunicate, Styela clava	[799]
Crabrolin	Wasp, Vespa crabro	[786,798]
Daptomycin	Actinobacteria, Streptomyces roseosporus	[853,854,855]
Dermcidin	Human sweat	[801]
Designed peptides	Synthetic	[795,796,797,856,857,858,859,860,861,862,863,864,865,866]
Esculentin 2	Frog, Glandirana emeljanovi	[867]
Gramicidins	Gram–Positive bacteria, Bacillus brevis	[853,868]
Kalata B1	Cyclotide from Oldenlandia affinis (plant)	[869]
LDKL, LDKA	Synthetic	[830]
LL-3	Human	[793]
Maculatin	Frog, Litoria aurea	[828]
Magainin 2	African clawed frog Xenopus laevis	[822,831,870,871]
Melittin	Honeybee, Apis mellifera	[807,818,819,820,822,824,825,826,827,832,872,873,874,875,876]
MSI-103	Synthetic	[796]
Nisin	Lactic acid bacteria	[877]
Pardaxin	Fish, Pardachirus marmoratus	[800,806]
PGLa	African clawed frog Xenopus laevis	[788,796]
Pleurocidin	Fish, Pleuronectes americanus	[871]
Polymyxins	Gram–positive bacteria e.g., Paenibacillus polymyxa	[853,878]
Thanatins	Insect defense peptides	[815]
Trichogin	Fungus, Trichoderma longibrachiatum	[879]
β-Defensin	Innate immunological system	[794]
Cecropin	Moth Hyalophora cecropia	[789]
Peptoids	Synthetic	[837,838,839,840,841]

## 6. Role of Membrane in Substrate (Drug) Selection of Membrane Proteins

### 6.1. Membrane Proteins: The Majority of Drug Targets

**Figure 17:**
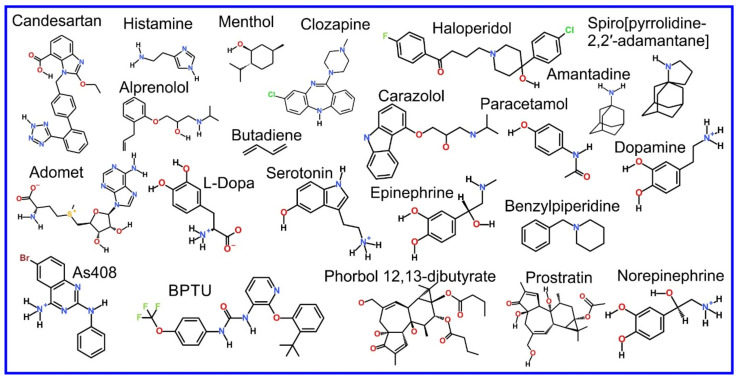
Chemical structure of drugs introduced in Section 6.

In addition to direct drug–membrane interactions, lipid membranes also play a role in the interaction between substrates and membrane–associated proteins, with both substrate–membrane and protein–membrane interactions playing roles in the substrate, and thus the drug selection mechanism of membrane-associated proteins. Since membrane–associated proteins are, in fact, the majority of drug targets [880,881], this issue should be central to drug design. However, it can be argued that, so far, it has not received the attention it deserves (Figure 17).

Membrane associated proteins can be subdivided into three categories: (1) multi–pass (integral) membrane proteins, where the ligand/substrate binding site is frequently within the membrane core (39% of membrane proteins [882]), (2) bitopic (single-pass) membrane proteins with one or more domains outside the membrane but anchored to the membrane through a single trans-membrane helix, with the functional domain, thus the active site, located outside the membrane (36% of membrane proteins [882]), and (3) peripheral membrane proteins, with no permanent association with the membrane (16% of membrane proteins [882]); however, for catalytic activity that involves interaction with a membrane, catalysis occurs at the membrane surface.

There is what can arguably be described as a fourth variety of membrane associated proteins that we, however, do not discuss in this review paper as they have not been studied to as significant an extent: 9% of membrane proteins are covalently bound with lipids; a common example is a glycosylphosphatidylinositol (GPI) molecule bound to the C terminus of the protein, known as a GPI-anchor [882]. As is the case with bitopic membrane proteins, the functional domain of the protein is located completely outside the membrane but is bound to a lipid membrane through an anchor.

The term “multi-pass” to describe integral membrane proteins refers to the structure of the trans-membrane domain, composed of several (up to 14 [883]) α-helices as the protein threads back and forth through the membrane; another class of integral membrane protein exists with a β–barrel rather than a set of α-helices as the trans-membrane domain. However, such proteins are not common [884] and have not been used as drug targets, thus they are not discussed in this review; yet, it should be mentioned that β-barrel membrane proteins are predominately found in bacterial membranes, e.g., the outer membrane of gram negative bacteria (in eukaryotic cells only in mitochondria and chloroplasts), thus could possibly in the future be potential targets for antibiotics, though, as far as we are aware, this is not yet the case.

A mechanistic understanding of the role the lipid plays in the substrate selection of membrane proteins is a key element of the design of drugs that target them, and MD simulation has a central role to play in obtaining this; we now discuss examples where MD simulation has obtained insight relevant to drug design, for all three varieties of membrane associated proteins.

In contrast to multi-pass (integral) membrane proteins, where considerable work has been performed, in particular the GPCR class of membrane proteins e.g., [885,886,887,888,889,890,891,892,893,894,895,896,897,898,899] (for review see references: [31,900,901,902,903]), that are a very hot topic, peripheral and bitopic (single pass) membrane proteins, which constitute 43–45% of transmembrane proteins [883], are underrepresented in MD simulation studies.

One of the reasons for this is that obtaining experimental insight into the nature of the membrane–protein interaction for weakly membrane associated proteins is extremely challenging. Obtaining structures from x-ray crystallography or cryo-EM, while less straightforward than for the case of water-soluble proteins, is still possible for integral membrane proteins within membrane-like nanodiscs [904,905,906] or a detergent environment [907,908,909]; obtaining such information for the more weakly associated proteins directly from experiment is challenging and rarely achieved. The rare cases of success in this regard represent a Herculean effort: one must work with incomplete structures combining fragments originating from several PDB entries and, frequently, the assistance of theoretical methods is necessary (e.g., homology modeling [910,911,912] and modeling of short loops [913]). Moreover, extracellular domains of bitopic receptors are heavily glycosylated and the precise sequences of these complex carbohydrate branches have, in most cases, not been determined. The few MD simulation studies of glycosylated receptor proteins that have been carried out have demonstrated that glycosylation determines the behavior of these receptors [914,915,916]. Interactions of these latter two classes of membrane proteins with lipids differ significantly in comparison to multi-pass (integral) membrane proteins since interactions with the lipid headgroups become dominant over interactions with the membrane core. As mentioned in the beginning of this review, the solution of the structure from sequence problem by the Alphafold project [1] completely changes the game; initial results show promise for even the structure of bitopic membrane proteins, so there may soon be rapid progress in this area.

### 6.2. Multi-Pass (Integral) Membrane Proteins

#### 6.2.1. Our Discussion Follows the Framework of Vauquelin

Multi pass membrane proteins are the class of membrane proteins that have been experimentally and computationally studied the most in their context as drug targets, primarily due to their functions as receptors (40% of multi pass membrane proteins), channels and transporters (another 24%), and enzymes (yet another 16%). The importance of this group of proteins may be exemplified by the Nobel prizes awarded for the study of various aspects of an important family of 901 (in human) integral membrane proteins [917], the G coupled receptors, including Nobel prizes in Medicine and Physiology: 1967 (studies of rhodopsin), 1988 (discovery of β-blockers), 1994 (discovery of G-protein), 2004 (odorant receptors), and even a Nobel prize in Chemistry: 2012 (3D structure of GPCRs) (GPCR) [918,919,920,921,922]. Issues relating to the design of drugs to target integral membrane proteins are covered in several already published reviews [923,924,925,926].

**Figure 18:**
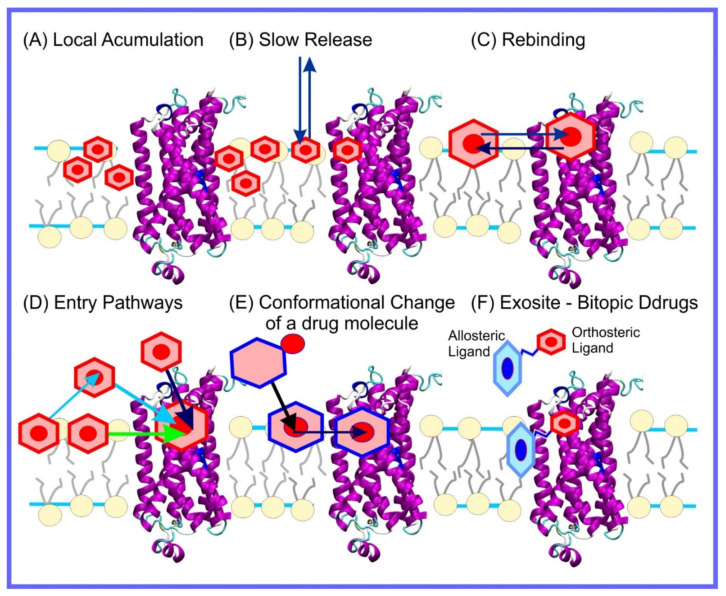
Accumulation of drugs in the membrane and its consequences for drug–protein interactions. Based on Figure 2 from ref. [927].

In describing the role played by MD simulation in the study of multi-pass membrane proteins, in their context as drug targets, we will follow the categorization scheme developed by Vauquelin, presented in ref [927], shown as a schematic in Figure 18, i.e., we draw attention to the contribution that MD simulations and related methods have made towards understanding drug–membrane–target protein relationships using their classification scheme, as a framework for our discussion. The scheme is summarized (see Figure 18) as: (1) the effective increase in both the kinetics and affinity due to drug accumulation in the membrane and the resultant reduced dimensionality of drug diffusion (Figure 18A–C); (2) how the membrane affects the entry of the ligand into the binding site (Figure 18D); (3) the role the membrane plays in the ligand interaction with the protein (Figure 18E); and (4) the possible design of hybrid drugs connecting orthosteric and allosteric pockets (Figure 18F). Our discussion will now follow this framework in order.

**(1) The accumulation of drug molecules in membranes significantly increases the local concentration of the drug molecule in the vicinity of the targeted membrane proteins in comparison to bulk solution, thus increasing the apparent affinity of the drug for the active site and making the drug kinetics more favorable (Figure 18A).**

Due to the high affinity of drugs for the lipid membrane, the release of the drug from the membrane environment is slow and the local concentration of the drug remains enhanced in relation to its bulk concentration in the solvent medium. This results in drug molecules being predominantly confined to the two-dimensional environment of the membrane that the protein is associated with, rather than the three-dimensional environment of the bulk solution around the protein. Thus, as a result of geometry alone, diffusion confined to two dimensions is significantly more limited in comparison to diffusion in three dimensions; the drug is more likely to remain in the vicinity of the protein with an increased chance of binding again as a result of locating preferentially to the lipid membrane as opposed to being randomly distributed in the bulk solution. This phenomenon is known as “rebinding” and has been documented experimentally [928] and considered in models of drug kinetics [929]. In order to take this into account, micro–pharmacokinetic models, which consider local drug concentration, have been proposed.

Extensive studies of ligands of the β2-adrenergic receptor have demonstrated that lipophilic drugs that accumulate on the lipid bilayer are characterized by a higher rate of drug−target protein association (*K_on_*) in comparison to the more hydrophilic drugs with the same pharmacophore [930]. On the other hand, the dissociation rate (*K_off_*) was not found to be dependent on drug lipophilicity [930], as lipids do not affect protein–drug interactions inside the binding site. Subsequent studies that used fluorescently labeled propranolol found an accelerated accumulation of the drug on the cell membrane and suggested that the actual affinity of propranolol for the β2-adrenergic receptor was significantly less than previously anticipated, based on a standard measurement assuming a uniform distribution of the drug in the volume of a sample [931].

In addition to the kinetics of binding, this phenomenon will affect the effective affinity, of the binding site, for the ligand; it will be a factor that is missed by conventional docking and scoring studies that do not take the membrane into account. While this will not affect the enthalpic term of the binding affinity, it will have a marked effect on the entropic component; bias of drug molecules to confinement to a two-dimensional plane rather than the three-dimensional bulk medium lowers the entropic penalty of binding. Techniques that use MD simulation with a force bias, that we discussed previously, can be used to obtain a quantitative estimation of this effect. For example, a deconvolution of the membrane and protein binding contributions, i.e., separating the contributions to affinity due to the membrane and the binding site, performed for β2 adrenergic receptor ligands, improved the accuracy of the results of a structure–activity relationship analysis [932].

We can now, in light of the discussion of the activity of membrane proteins, return to our discussion of the mechanism of action of anesthetics. The aforementioned Meyer–Overton correlation is the result that more lipophilic anesthetics have a tendency to produce stronger effects. The correlation can be most easily explained through a micro-pharmacokinetic model. However, this interpretation, while the most elegant, does not support a membrane mediated mechanism for the action of anesthetics but rather suggests the involvement of membrane proteins; nevertheless, the possibility that modification of the membrane properties plays some role in a more complex mechanism of action for anesthetics is not excluded.

**(2) The accumulation of drugs in the membrane will affect the entry pathway of a ligand into the binding site as this will create a bias, increasing the likelihood that drugs approach the protein from within the lipid membrane (Figure 18D).**

As discussed previously, the accumulation of ligand molecules in the membrane will increase both the entrance frequency and apparent affinity of ligands for the active site; this is, however, not the only effect of the preferential location of ligands to the membrane: the path taken to enter the protein will also be affected, resulting in further inaccuracy in the standard “lock and key” paradigm that does not take the membrane into account. In many cases, the active site is entirely within the membrane core, with entry being made from the lipid phase into an opening between trans-membrane helices. In fact, the direct entry of a ligand from a membrane into a receptor can be anticipated based on the crystal structure of the receptor. For example, for the case of the sphingosine 1–phosphate receptor 1 the extracellular opening is blocked by the N–terminus and extracellular loops [933]; the entry location for ligands is rather from within the membrane core to a receptor deep within the lipid phase of the membrane between trans membrane (TM) helices TMI and TMVII. For the protein Opsin, the retinal binding site opens directly into the membrane core at two points between TM1–TM7 and TM5–TM6 [934]. The two openings and the central cavity, constitute a channel through the protein, with a length of 7 nm and a width of 1.16–0.32 nm. The entry of a ligand into the binding pocket has been studied via MD simulations, in some cases using force bias methods and these will be discussed in detail later in this section. Membrane mediated ligand entry/exit pathways to the binding pocket have been discussed in previously published review articles for the case of GPCRs [935,936], but also for the larger group of membrane proteins that includes GPCRs, ion channels, and transporters [41].

**(3) Drug partitioning into the membrane can alter the conformation of the drug molecule, thus affecting its interaction with the binding site of the target protein.**

When immersed in the hydrophobic environment of the membrane core, the situation is roughly equivalent to immersion in a non-polar solvent. Many molecules exist that are soluble in both polar and non-polar solvents and achieve this through changing their conformation; it is thus possible for a molecule to exist that first enters the membrane, then alters its conformation within the membrane core and, only at this point, with its new conformation adopted within the hydrophobic environment of the membrane core, has a structure with a high affinity for a specific binding site of a membrane protein (Figure 18E). Through MD simulations, we can directly observe such behavior, for example, the candesartan antagonist of the GPCR AT1 receptor changes conformation after entry into the membrane [193]. Another example is the cannabinoid analog AMG3. This molecule is in possession of six flexible bonds, thus allowing for significant conformational change; MD simulations of AMG3 in a lipid bilayer were used to find the optimal conformation for binding to both the CB1 and CB2 receptors [338].

**(4) The exosite model (Figure 18F) assumes that the drug molecule is composed of two linked component ligands: the first binding to the orthosteric pocket and the second to the allosteric site [937].**

The allosteric sites are frequently located at the protein–lipid interphase and allow for increased specificity in comparison to a drug that targets only the orthosteric (binding) pocket. Due to the highly conserved structure of orthosteric pockets, e.g., among GPCRs, it is difficult to find a highly selective ligand based on this criterion alone; an inclusion of the allosteric site into the computational drug design approach allows for the necessary targeting specificity. The use of the allosteric site in addition to the orthosteric pocket in drug design has been referred to as the “exosite model” and “the design of bitopic ligands/drugs” by two separate research communities [938,939]. Modeling methods, including MD simulations, are one of the tools used in the design of bitopic drugs [900,940,941]; however, in these studies lipids are only considered as a passive component and their complete role in substrate selection is not fully elucidated.

#### 6.2.2. Exploring the Complex Pathways to the Active Sites of Integral Membrane Proteins

The time scale that can be investigated through MD simulations is a limiting factor for a direct observation of ligand entry into the binding site of receptors or enzymes; only a few studies have thus addressed this problem with unbiased MD simulations. A study of histamine entry into the H4 receptor found evidence of two possible entry pathways [407], one of which overlaps with the previously determined Na^+^ entry pathway [556]. In extensive simulations of alprenolol, an inhibitor of the β2 adrenergic receptor, the entrance of the drug into the binding pocket of the receptor was observed 12 times; entry, however, occurred from the water phase despite strong preferences observed for the drug molecule to locate to the lipid phase and remain there for the majority of the simulation [195]; in this case, the membrane acts as a drug reservoir, and its mechanism described in Figure 18A. The ligand entrance into the protein binding site, directly from within the lipid core of the membrane, was demonstrated for the example of the cannabinoid sn-2-arachidonoylglycerol (2-AG) and the CB2 receptors [455]. The entrance gate for the ligand locates between TM6 and TM7. The entry of the ligand into the sphingosine-1-phosphate receptor-1 was characterized using multiple, microsecond long simulations [942]. The entry path began in the vestibule at the top of TM7, then preceded to enter the protein between TM7 and TM1. A similar MD study of the binding and activation of Orexin–A was also carried out [943]. In studies of the dopamine D3 receptor, a set of 1000 short 20 ns simulations of systems, where dopamine was initially located in the water phase, found only 22 cases of direct entrance of dopamine into the binding pocket of the receptor [394]. In 736 cases, the dopamine molecule located to the membrane, settling among the lipids but not in contact with the protein; in another 180 cases, it located to the protein–lipid interface and in the remaining 62 cases located to the protein surface exposed to the water phase. These numbers closely correspond to the surface area of the membrane occupied by lipids and protein in the studied model; thus, this provides evidence that the protein has no inherent properties that attract the ligand, i.e., the relative number of binding events correspond to the relative surface areas, an unexpected result.

**Figure 19:**
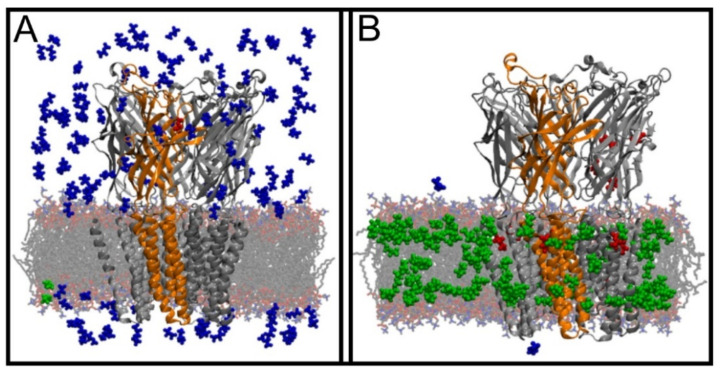
Partitioning of desflurane into the membrane and Gloeobacter violaceus ligand-gated ion channel studied using a flooding simulation. Snapshots of the simulated model at the (**A**) beginning and (**B**) end of the simulation. Desflurane molecules were colored according to their location: blue—water phase, green—lipid bilayer, red—ion channel. Reproduced with permission from ref. [307].

Flooding simulation was used to study the interaction between the *Gloeobacter violaceus* ligand-gated ion channel and the general anesthetic desflurane [307]. These studies demonstrated that the desflurane molecule enters a binding site, with a structure known from X-ray crystallography [712], via a membrane-mediated pathway. Moreover, simulations revealed an additional binding site not anticipated in previous studies. In this case, desflurane entered the binding site directly from the lipid core (Figure 19). In another flooding simulation study, menthol, a small compound extracted from mint that acts as a local anesthetic, bound to the α4β2 nicotinic acetylcholine receptor, to a binding site located at the lipid–protein interface via a membrane–mediated pathway [434]. In other flooding simulations of two anesthetics, chloroform and isoflurane, the molecules first entered the lipid bilayer and then subsequently diffused to the allosteric binding sites of the vanilloid-1 receptor (TRPV1) [297]. These studies identified five binding sites for chloroform and three for isoflurane, in spite of the fact that the overall affinity of the aforementioned drug molecules toward TRPV1 was relatively small.

In some cases, MD simulations determined only a fragment of the drug entry pathway or merely demonstrated the accumulation of the drug in the vicinity of possible entry points. For example, multiple 500 ns MD simulations of the drugs clozapine and haloperidol, acting as ligands of the dopamine D2 and D3 receptors, determined the pathway between the binding site and the receptor vestibule, where drugs were placed into the initial structure; however, the pathway between the vestibule and membrane remains unknown [322]. Amantadine is a drug used to treat influenza. At the molecular level, amantadine inhibits the M2 transmembrane proton channel, preventing virus budding. Through MD simulation, it has been demonstrated that the more hydrophobic derivative of amantadine, spiro[pyrrolidine2,2′-adamantane], has a tendency to aggregate around the M2 channel to a greater extent than amantadine; this could be the mechanism behind the experimentally observed higher binding affinity of spiro[pyrrolidine2,2′-adamantane] for the M2 protein in comparison to the case for amantadine [254]. Simulations of candesartan, an antagonist of the GPCR AT1 receptor, an integral membrane protein, found evidence that that the drug approaches the opening between helices 4 and 5 from where entry into the binding site has a high probability [193]. This study then made the important observation that the two-dimensional diffusion coefficient of the drug, in the plane of the membrane, calculated from the MD simulation trajectories, was in excellent agreement with the diffusion coefficient measured experimentally using liposomes as a membrane model. This result further supports the 2D model of drug diffusion when approaching the previously discussed receptor. Finally, Kiriakidi et al., suggest that lipids affect the conformation of the candesartan molecule, potentially affecting the affinity of the drug for the binding site. Yet, for two lipid-like drugs, N-arachidonylglycine, and oleoyl-L-carnitine, the only possible pathway to access the binding site is from within the membrane. Extensive MD simulations of the glycine transporter GlyT2 embedded into the lipid bilayer found evidence for numerous possible drug interactions with the protein; unfortunately, this study was unable to determine the specific binding site responsible for receptor inhibition [137].

**Figure 20:**
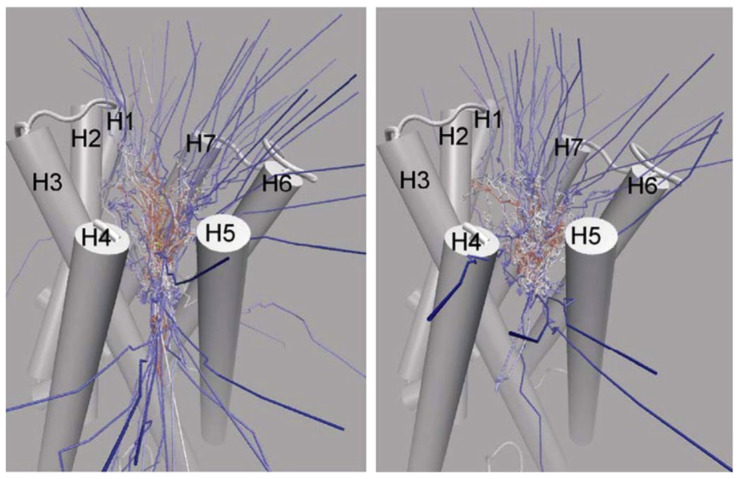
Entry/exit pathways of carazolol observed in the RAMD simulations. Left and right panels show sets of simulations with different acceleration magnitudes. The color scale is from yellow at the beginning of the simulation to blue at the end of the simulation. The starting conformation of the receptor is shown in a cartoon representation. Reproduced with permission from ref. [200].

As stated above, the timescales involved limit the extent to which unbiased MD simulation can elucidate the interaction between potential ligands and integral membrane proteins. As a result, several groups have made use of a variety of force bias, and other enhanced sampling techniques to gain further insight; the situation is similar to that regarding membrane translocation discussed earlier, though the specific methods that are optimal differ. Studies of the β-adrenergic receptor using RAMD found five possible exit pathways of the receptor antagonist carazolol [200]. The most frequent entry/exit pathway was passing through the extracellular opening, i.e., the opening exposed to the solvent, at the top of the receptor; however, in 30% of runs, carazolol exited the binding pocket through clefts between the transmembrane helices. The most frequently utilized alternate pathway was the cleft between the TM4 and TM5 helices (TM4–TM5); other pathways pass between helices: TM5–TM6, TM1–TM2, TM1–TM7, and TM6–TM7 (see Figure 20). For the case of dopamine, D3 receptor RAMD simulations revealed an alternative pathway that passed through a lateral gate between helices TM5 and TM6 [394]. Subsequent free energy calculation using the umbrella sampling algorithm [93] demonstrated that there is no energy barrier along this pathway. Temperature accelerated molecular dynamics simulation was applied to the study of protease-activated receptor-1, a GPCR activated by the binding of a peptide; it was determined that an exit/entry patch of a ligand from the binding pocket directly faces into the membrane hydrocarbon core [944]. The two observed exit/entry points were located between helices TM4, TM5, TM6, and TM7. These simulations also found evidence for a third possible entry/exit pathway located between the extracellular loops of the receptor and the water phase.

Most drugs bind to the orthosteric binding pockets of proteins where their natural ligands bind; however, it is possible for proteins to be modulated by a drug binding to an allosteric site, e.g., for the GPCR family, 7 allosteric pockets facing into the membrane hydrocarbon core have been found in 12 GPCRs [945]. Yet, the most recent studies of M5 muscarinic acetylcholine receptor revealed the presence of three allosteric sites including one novel site [946]. Recent studies provide an example of such an allosteric modulator of the β2-adrenergic receptor: compound AS408 was shown to bind between transmembrane helices TM3 and TM5 at the surface, directly facing the membrane core, thus stabilizing the inactive conformation of the receptor [197]. In MD simulations, Yuan et al., demonstrated the binding of the P2Y1 receptor antagonist, BPTU, to the extra-helical site between helices 1 and 3 [329]. The binding took place via the lipid bilayer in three steps: first, BPTU enters the water–membrane interface from the water phase; next, BPTU forms an initial set of interactions with the extracellular loop 2 of the receptor; finally, the drug translocates to the binding site. Similarly, the binding site for ZM241385 in adenosine receptor type 2A was identified at the protein–lipid interface [947]; in this case, lipids stabilized the binding pose of the ligand.

### 6.3. Bitopic Membrane Proteins

#### 6.3.1. Proteins with a Tenuous, but Permanent Connection to a Specific Lipid Bilayer

As described above, for the case of bitopic proteins, the protein is tethered to the membrane through a single transmembrane helix, but with the functional domain/domains, thus active site/sites, located outside the membrane; there is, however, considerable evidence that the lipid membrane plays a role in substrate selection [381]. Monk et al., [948] in the very first complete structure of a bitopic protein to be determined, already found evidence that the trans–membrane helix and linker segment controls the orientation of the catalytic domain relative to the membrane, thus indicating a role for the membrane in catalysis; bitopic proteins cannot be thought of as merely a catalytic domain loosely attached to the membrane like a balloon with the linker segment as a piece of string attaching it to a the trans membrane helix acting like a pin in a cork board: the linker segment and trans-membrane helix play an active role in positioning the catalytic domain relative to the membrane.

We will now focus on four cases where MD simulation has seen considerable success in elucidating the role the membrane plays in substrate selection (thus drug affinity) for bitopic membrane proteins: (1) cytochrome P450, (2) the membrane bound isoform of catechol-o-methyltransferase (MB–COMT), (3) monoamine oxidase, and (4) tropomyosin receptor kinase B. In many cases, for example MB–COMT, these are proteins with water–soluble in addition to bitopic, membrane bound, isoforms.

#### 6.3.2. Cytochrome P450

Cytochrome P450 (CYP) is a family of 41 (in humans) bitopic membrane enzymes responsible for both the catabolism (synthesis) and anabolism (degradation) of a variety of small molecules [949]. For example, the synthesis of cholesterol, steroid hormones, bile acids, vitamin D3, and second messengers derived from polyunsaturated lipids is performed by CYPs. CYPs are also responsible for the degradation of small molecules, e.g., neurotransmitters, and various xenobiotics, including about 50% of all approved drugs [950,951,952,953,954]. A review of MD simulation studies of CYPs is given in ref. [955].

The catalytic domains of proteins belonging to the cytochrome P450 family have been studied extensively, in a water environment without the trans-membrane helix present, using MD simulation and related molecular modeling methodologies in the context of both drug design (e.g., [956,957]) and their mechanism of catalysis (e.g., [958]). It has been argued that these studies can elucidate the catalytic mechanism of membrane bound proteins, since the catalytic site is placed in an internal pocket of the protein and does not interact directly with the membrane. An analysis of the conformation of the active site of CYP 3A4 in both membrane bound and water-soluble forms reveals a lack of significant difference, the volume of the binding site was, however, larger in the water-soluble simulations [959].

The majority of known crystal structures of CYP P450 are only partial structures, containing only the catalytic domain. The only complete protein structure (including linker, transmembrane helix, and attached membrane) belonging to this family is yeast lanosterol 14α-demethylase [948]. The structure of lanosterol 14α-demethylase suggests that the transmembrane helix affects the orientation of the catalytic domain towards the lipid bilayer. Unsurprisingly, numerous MD simulation studies have been dedicated to elucidating the orientation of the catalytic domain relative to the membrane surface and its specific interactions with lipids: for example, this was achieved for CYP 2C9 [960]; CYP 17A1 [961]; CYP1A2, 2A6, 2C9, 2D6, 2E1, and 3A4 [326,962,963] through MD simulations, then subsequently validated experimentally. Interestingly, the orientation of the catalytic domains of CYP 2C9 and CYP 2C19 were found to differ from each other significantly, in spite of the relatively high extent of sequence similarity; through MD simulation it was determined that the difference was induced by only three residues located at the membrane–protein interface [964]. Interestingly, truncation of the transmembrane helix in CYP 19A1 and 17A1 did not change the orientation of the catalytic domain significantly; however, mutations in the transmembrane helix of CYP 17A1 destabilized the interaction between the membrane and catalytic domain of the protein [965]. MD simulations also demonstrated that lipid composition, e.g., the addition of cholesterol [966] or charged lipids [967], affects the orientation of the extra-membrane domain with respect to the membrane surface. Additionally, the choice of force field (parameters set) may have a measurable, but not significant, effect on the orientation of the domains of the protein that are outside the membrane [968].

**Figure 21:**
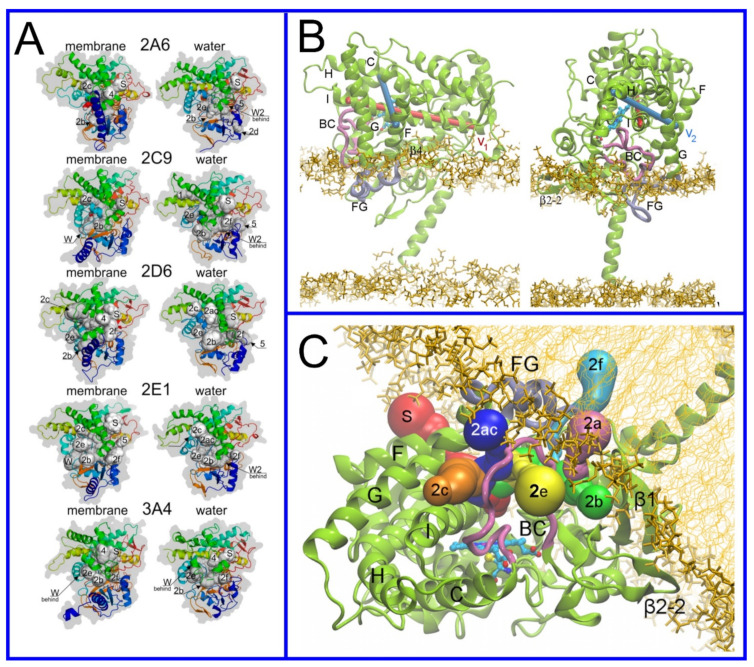
(**A**) Snapshots of five CYP enzymes simulated attached to the membrane (left) and simulated in water (right) with open (minimum radius 1.5 Å) channels shown. Reproduced with permission from ref. [962]. (**B**) Snapshots of the models of membrane-bound CYP 2C9 and (**C**) visualization of the channels observed in MD simulations of these models. Reproduced with permission from ref. [960].

The active site of CYP enzymes is located inside the protein, thus substrates and products enter/exit the active site through numerous possible channels [969] (Figure 21). Such channels have been observed in crystal structures [969], MD simulations [969], RAMD calculations [970,971], and steered MD simulations [972]. The channels found can be subdivided into seven classes: channel 1, channel 2, channel 3, channel 4, channel 5, the solvent channel and the water channel; in turn, channel 2 has 7 subclasses. The aforementioned channels open towards the membrane core, membrane–water interface, and directly into the solvent phase (water) (Figure 21).

Free energy calculations have demonstrated that the substrate may enter/exit via various channels; however, the free energy barriers encountered traversing the channels, i.e., largest barrier along the pathway for each case, differ by up to 8 kJ/mol [388]. An MD simulation study that compared the aforementioned channels in the human and human parasite *Trypanosoma brucei* versions of CYP51s provided evidence that subtle differences in structure exist which could possibly be taken advantage of to design a drug to specifically target the parasite enzyme [973], i.e., design drugs based on transit through the channels, selecting a drug that has a lower free energy barrier for traversing the channels of the form of the enzyme in the parasite than that present for the host.

The interactions of CYPs with lipids affects the behavior of the entry channels significantly. In studies of CYP 1A2, the authors demonstrated that the probability of the opening of a specific channel is dependent on contact with the membrane, with the radius of the channel regulated through interactions with lipids in the membrane [974]; through very subtle conformational change in the protein, the membrane facing channels 2b, 2d, and 4 are enlarged, i.e., “opened”, when the protein is in contact with the membrane while the water facing channel 2c is enlarged when the protein is in the water phase. For the case of the protein CYP 2B4, free energy calculations have determined that the transition from the open to the closed state is associated with a free energy change of 10 Kcal/mol for the membrane-bound protein and 25 Kcal/mol for the protein in solution [975]. Moreover, for the case of CYP 17A1, the membrane facilitates the opening of the entry channels [961]. This opening of channels or catalytic pockets is also observed in peripheral membrane proteins (discussed in more detail in the following section) due to interactions with the lipid membrane, e.g., simulations have observed this occurring for the phospholipase A2 [976,977], dihydroorotate dehydrogenase [978], monoglycosyltransferase [979], and other proteins that we will later discuss.

Combined experimental and simulation studies of CYP 2B4 reveal the presence of a sphingomyelin binding region on the protein and a protein-induced increase in the level of cholesterol and sphingomyelin in nanodiscs incubated with micelles containing lipids [980]. Putting these together, the authors concluded that CYP 2B4 induces the formation of ordered raft-like domains in its environment as lipids are exchanged between the micelles and the nanodisks. In turn, at the ordered membrane, the thermal stability of CYP 2B4 is significantly increased. The lipid environment also affected the affinity of hydrophobic substrates for CYP 2B4: highest in the raft-like environment and lowest in water solution. The behavior of CYPs may also be modulated due to the formation of heterodimers with cytochrome b5 or cytochrome P450 reductase [981,982].

The results of older MD simulation studies have provided evidence that the location and membrane orientation of the entry channels of CYP 2C9 correspond well with possible substrate positions [272]. In recent unbiased MD simulations, spontaneous events of the entry into the catalytic pocket of CYP 2D6, by substrates paracetamol and butadiene, from the water–membrane interface, were seen [285]. In eight observed events of paracetamol entry into the catalytic pocket, three possible channels were used; thus, there is no specificity for the entry pathway for this compound. For butadiene, only two events of entry occurred, both through channel 2c. Using a combination of extensive, unbiased MD simulations and RAMD, the entrance pathway from the membrane to the binding site was determined for testosterone [408]; umbrella sampling calculations along this pathway revealed the free energy landscape.

Through MD simulations, the effect of point mutations on the enzymatic activity of CYP 2C19 was analyzed, providing evidence for a possible mechanism responsible for the reduction of the affinity of the enzyme for the substrate, e.g., local deformation of the secondary structure of the protein results in a change in the shape and the dynamics of the catalytic pocket that, in turn, gives rise to the formation of a new stable network of hydrogen bonds [983]. A study of CYP 1A1 found that point mutations may facilitate the interactions of organic pollutants with the protein [984], while CYP 2D6 is characterized by a high degree of genetic polymorphism that leads to several possible phenotypes [985]. The results of MD simulations of a few variants of CYP 2D6 indicated that a decrease, or even a complete loss, of enzymatic activity may arise from a decrease in the frequency of channel openings, decreased minimal diameters of channels, or decreased catalytic site volume [986]. On the other hand, an increase of active site volume facilitates enzyme activity. MD simulations have been used to design mutations of CYP 3A4, designed to enhance the rate with which it epoxides carbamazepine [328]. Finally, interactions of the drugs progesterone and carbamazepine [326,327] and atorvastatin and dronedarone [312] have been studied using a combination of experimental methods and MD simulations.

#### 6.3.3. Catechol-O-methyltransferase

**Figure 22:**
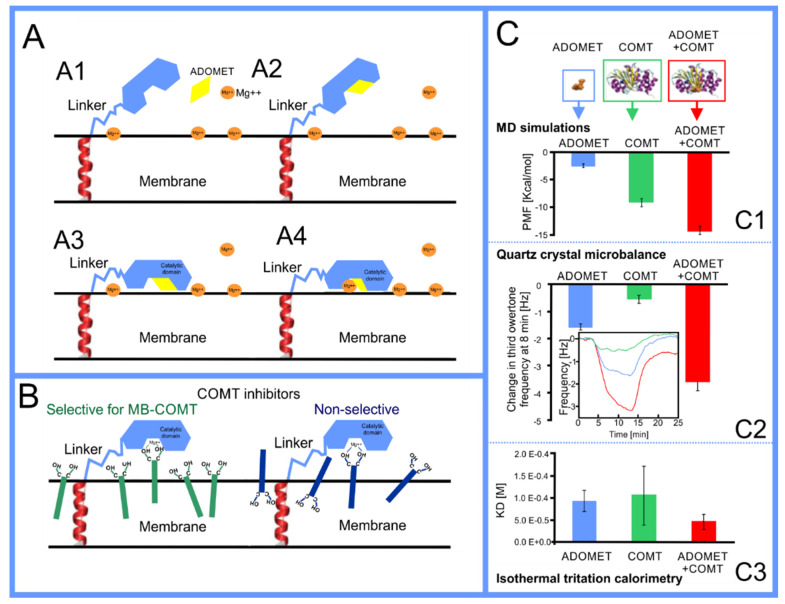
(**A**) Schematic of the catalytic mechanism of MB–COMT. (**B**) The behavior of MB–COMT selective vs. non–selective inhibitors in the membrane. (**C**) Quantitative experimental and computational results for the interactions of the ADOMET and catalytic domain of COMT in complex and separately with lipids: (**C1**) the free energy changes calculated computationally with umbrella sampling methods; (**C2**) quartz crystal microbalance (QCM) frequency changes during interaction of COMT with the lipid bilayer; (**C3**) dissociation constant from liposomes determined by isothermal calorimetry. Reproduced with permission from ref. [381]. Copyright 2018 the Royal Society of Chemistry.

The enzyme catechol-O-methyltransferase (COMT) is an exciting example of a protein that has two isoforms: a water–soluble form (S–COMT) that is generally thought to perform its catalysis in solution but with the capacity to act also as a peripheral membrane protein and a membrane–bound isoform (MB–COMT), a bitopic protein [987]. The difference between S–COMT and MB–COMT is a 26 residue long transmembrane α–helix and a 24-residue long linker segment connecting the catalytic domain with the transmembrane segment. The function of COMT is the catabolism of catechols (Figure 22), including the neurotransmitters dopamine, epinephrine, and norepinephrine. Inhibitors for COMT are thus used to treat Parkinson’s disease together with the dopamine precursor L–DOPA [645,646]; the goal is to prevent dopamine deficiency in neurons, and the role of COMT inhibitors is to avoid L–DOPA degradation before it is converted to dopamine. Importantly, in the central nervous system, MB–COMT is the dominant form of the enzyme; thus, selective inhibition could possibly be profitable for patients. In our studies of MB–COMT, we found evidence, through MD simulation, that a group of inhibitors specific for MB–COMT is characterized by a clear orientation, when in the lipid membrane, with the catechol group oriented outwards and towards the water phase (Figure 22) [381]. The catalytic activity of COMT first involves the binding of the S-adenosyl methionine (ADOMET) cofactor, then an Mg^++^ ion is bound to form the binding pocket (Figure 22). As part of the same study, a simulation of the entire MB–COMT in the lipid membrane in both holo- and apo- (with and without ADOMET cofactor bound) states was performed and it was determined that the binding of ADOMET leads, i.e., orients, the catalytic surface towards the lipid headgroups, through the creation of a membrane binding patch similar to that found in peripheral membrane proteins, where the substrates are methylated at the membrane–water interface. Through MD simulation, the catalytic mechanism, unique to the membrane bound isoform of COMT, has been determined, presenting the tantalizing possibility of designing drugs that selectively target MB–COMT over S–COMT. Through datamining, other examples of proteins where the same mechanism can be exploited have been found.

#### 6.3.4. Monoamine Oxidase

Monoamine oxidase (MAO) A and B are bitopic proteins involved in the degradation of monoamines, particularly monoamine neurotransmitters: dopamine, serotonin, epinephrine, norepinephrine, histamine, and trace amines, as well as the catabolism of xenobiotics. As a result of this role, they are a drug target for inhibition in the treatment of Parkinson’s and Alzheimer’s disease and major depressive disorder [988,989,990]. Not surprisingly, MAO inhibitors are frequently studied using computational modeling methods including docking calculations, free energy calculations, and hybrid quantum mechanics/molecular mechanics (QM–MM) simulations, e.g., [991,992,993]. A recent example is the study of MAO–A and MAO–B inhibition by naturally occurring flavonoids that originate from medicinal plants [994]. Quantum mechanics/molecular mechanics simulations have also been used to elucidate the mechanism of enzymatic reaction catalyzed by MAO–A (e.g., [995]) and MOA–B (e.g., [996,997]); for a review of this topic, see ref. [998].

Simulations of MAO–A at the membrane−water interface provided evidence of the opening of a channel for the substrate and the reaction products that was not seen to occur in simulations performed without the lipid membrane, i.e., the channel remained closed [999]. For the case of MAO–B, the lipid bilayer was found to control the behavior of the two loops at the entrance of the active site, allowing for the opening of a channel [1000]. The other channel-facing membrane was observed in simulations of the MOA–B dimer [1001]. These studies also found evidence of changes in protein dynamics induced by the membrane, potentially affecting MOA–B activity.

It has been attempted, through experimental mutagenesis studies, to engineer MAOs from *Aspergillus niger* to act on larger substrates like benzyl-piperidine, thus transforming it into a device to synthesize chiral pharmaceuticals based on the benzo-piperidine scaffold; simulations were carried out to assist this endeavor [545] and residues in the channels leading to the catalytic pocket and residues inside the pocket were mutated in silico. The MD simulations demonstrated that increased hydrophobicity of the entrance channel and alteration of shape and size of the binding pocket provided the most efficient version of the enzyme.

#### 6.3.5. Tropomyosin Receptor Kinase B

**Figure 23:**
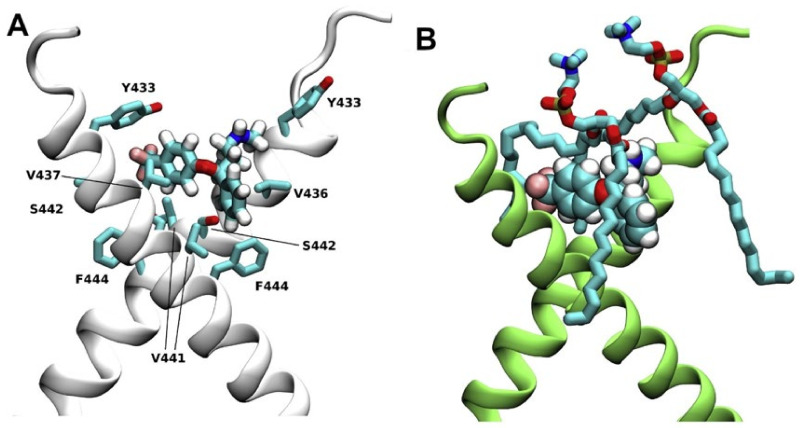
Transmembrane domain of TRKB dimer formed by the crisscross oriented helices forms a pocket for antidepressant binding. (**A**) Antidepressant fluoxetine is embedded in the crevice between the two helices of the TRKB transmembrane domain. Protein backbone shown in white cartoon and protein residues and fluoxetine in licorice representation. (**B**) Binding site for antidepressants at the outer opening of the crossed dimer is stabilized by the phospholipids. Protein backbone shown as green cartoon, the phospholipids in licorice and fluoxetine in vdW representations.

**Figure 24:**
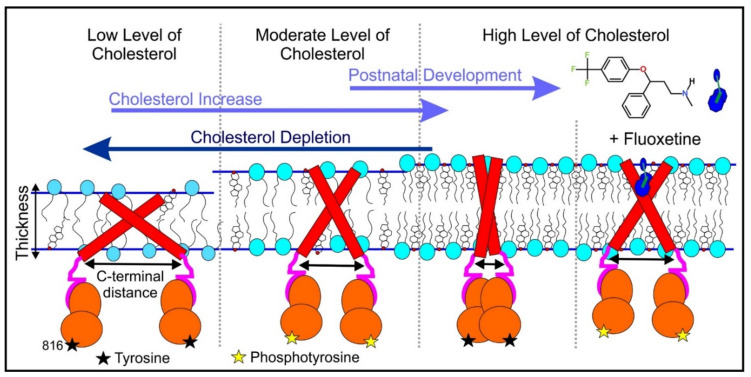
Schematic representation of how cholesterol and antidepressants regulate the activity of TRKB receptors through driving the orientation of its transmembrane helices. The rise of cholesterol content in the membrane increases its thickness. This forces the transition of TRKB transmembrane dimers towards the states with shorter C-terminal distances between the ends of the helixes (red rectangles). In turn, the C-terminal distances determine the arrangement of TRKB kinase domains (shown in orange) and the phosphorylation states of tyrosine 816 (black and yellow stars for native and phosphorylated states, respectively). The antidepressant fluoxetine (shown in blue), when bound to the pocket, preserves the stable transmembrane dimer conformation in a similar fashion to that observed at moderate cholesterol level and optimal for receptor activation.

The tropomyosin receptor kinase B (TRKB) belongs to the tyrosine kinase receptor family of proteins: a set of bitopic membrane proteins that operate as dimers with their trans–membrane α-helices paired in a crisscross orientation (see Figure 23 and Figure 24); their main function is to pass signals through the cell membrane [1002,1003]. The specific tyrosine kinase receptor TRKB is the main receptor of brain-derived neurotrophic factor (BDNF) and plays an important role both in neuron survival and the growth and differentiation of new neurons and synapses; it can thus be seen, on the scale of overall brain function, as an agent that promotes neuron plasticity, the increase of which, through a combination of drug and talk therapies, is currently seen as one of the most promising routes, following a mechanistic biophysical paradigm, for the treatment of clinical depression. Thus, combating the inhibition of TRKB activation by BDNF is a possible strategy for drug therapy [1004].

A recent study of TRKB by Casarotto et al., that utilized numerous experimental and computational methods demonstrated a possible mechanism through which the antidepressant fluoxetine can increase the extent to which TRKB is activated by BDNF, resulting in the desired increase in neuronal plasticity [320]. The angle between the two alpha helices of the TRKB dimer, arranged in the crisscross orientation mentioned above (see Figure 23), is determined by the thickness of the hydrophobic core of the lipid membrane; the two identical alpha helices each have the same fixed hydrophobic length and cholesterol is known to affect the thickness of a lipid membrane, thus the level of cholesterol in the lipid membrane will alter this angle. The angle between the helices determines the distance between the intracellular ends of the helices that, in turn, controls the positioning of the intracellular domains of the proteins; this mechanism switches the protein between the active or inactive states. Through a combination of experimental methodologies, Casarotto et al., found that TRKB activation is affected by the level of cholesterol in the neuronal membranes where the TRKB receptors reside; the study reveals a bell-shaped dependence between the level of cholesterol in the membrane and the extent to which TRKB activation by BDNF occurs: both low and high levels of cholesterol were found to inhibit BDNF receptor activation, while a moderate level of cholesterol was found to be optimal, i.e., the level of TRKB activation had a maximum at a certain level of cholesterol in the membrane. The same bell-shaped dependency was also observed in a direct binding assay of BDNF to TRKB [1005]. It can thus be surmised that BDNF activation is dependent on the angle between the two trans-membrane helices with a certain finite angle being optimal. It was then found that the presence of the antidepressant fluoxetine caused the extent of TRKB activation not to decrease when the cholesterol level in the membrane was raised above the formerly optimal level, i.e., the inhibitory effect of elevated cholesterol level was negated by the presence of fluoxetine.

Next, Casarotto et al., performed MD simulations of a TRKB dimer in a membrane with the antidepressant fluoxetine present; they made a truly startling discovery: a binding site for the fluoxetine molecule was found to exist at the point the two helices crossed; when a fluoxetine molecule was bound to this site, the fluoxetine molecule effectively jammed the two transmembrane helices like a rock in a pair of scissors, as shown in Figure 23. The study thus demonstrates how MD simulation has connected a set of experimental results to provide a complete picture of a hypothesis of the mechanism of action of the antidepressant fluoxetine, shown as a schematic in Figure 24. Mutagenesis experiments were then carried out to verify this; the fluoxetine binding site, found from MD simulation, was removed and the effect of fluoxetine on the cholesterol dependence of TRKB activity was found to disappear, as expected.

This mechanism also explains why antidepressants start activating TRKB only after 2 weeks of postnatal development: data from lipidomics indicates that, at the end of the second week of postnatal development, the level of cholesterol in the synaptic membranes of rodents drastically increases [1006], exactly the time when, in separate studies, antidepressants were found to start to become capable of activating TRKB [1007,1008].

The revolutionary potential of this possible discovery and the key role MD has played in it cannot be understated and already have been widely commented upon in the neuroscience community [1009,1010,1011,1012]; attempts have already been made to find small molecules which directly activate TRKB [1013] or positively stimulate BDNF signaling [1014].

### 6.4. Peripheral Membrane Proteins

#### 6.4.1. Proteins That Live in the Cytoplasm, but Work at the Membrane Surface

We finally come to the last category of membrane-associated proteins, and arguably the most difficult to study of all: peripheral membrane proteins. While these proteins have no permanent association with the membrane, the activity of the protein takes place at a lipid membrane; interaction with the lipid membrane plays a clear role in its activity. Peripheral membrane proteins are considered as possible drug targets [1015], in particular, the protein domains known to specifically bind lipid headgroups at the membrane surface [1016]; for example, it has been demonstrated that domains that bind phosphatidylinositols could be a possible drug target in cancer therapy [1017,1018].

Peripheral membrane proteins are frequently studied through MD simulations, but frequently without association with the lipid bilayers involved in their activity. For example, galectins, an important class of protein that recognizes carbohydrates, including glycolipids, have been studied in the context of protein–ganglioside carbohydrate headgroup interactions [1019,1020] or in the context of interactions with potential inhibitors [1021,1022]; the lipid bilayer has, however, not been included in any computational studies of galectins.

We will now outline a few examples of peripheral membrane proteins, including domains that recognize phosphatidylinositols and phosphatidylserine, protein toxin, and protein kinase C (PKC) where MD simulation has elucidated key elements of their mechanism of action.

#### 6.4.2. Protein Kinase C

Protein kinase C (PKC) plays many important roles in human physiology and aspects of its activity are thus also involved in several pathologies, including heart failure, cancer, Alzheimer’s disease, and diabetes [1023,1024,1025]. The catalytic activity occurs at the surface of a biomembrane, when activated by di-acyl-glycerol (DAG) in the membrane. In many cases moderate activation has been found to be beneficial while prolonged activation detrimental. PKC is thus a drug target for controlled activation and a detailed mechanistic understanding of the factors that modulate its activity and the role the membrane plays in mediating the DAG induced activation is pharmaceutically relevant. For a review of the use of computational methods for the development of ligands of PKC, see Katti and Igumenova (2021) [1026].

A set of MD simulation studies of the C1 and C2 domains of PKC have demonstrated the importance of specific lipids in regard to the docking of the protein to the lipid bilayer; specifically, the importance of anionic lipids has been demonstrated [1027,1028,1029,1030]. In other studies, PKC and its activator aplysiatoxin have been studied using MD simulations of this complex interacting with a lipid bilayer, in order to describe the binding mode of this potential anticancer drug [230]. In another computational study of PKC, the C1B domain was placed at the water–membrane interface and docked to four potential activators bryostatin, a bryostatin analog, phorbol 12,13–dibutyrate, and prostratin [231]; long MD simulations demonstrated that the C1B domain adopted a different orientation and positioning for each of the activators. These differences originated from variation in activator interactions with both water and lipids.

**Figure 25:**
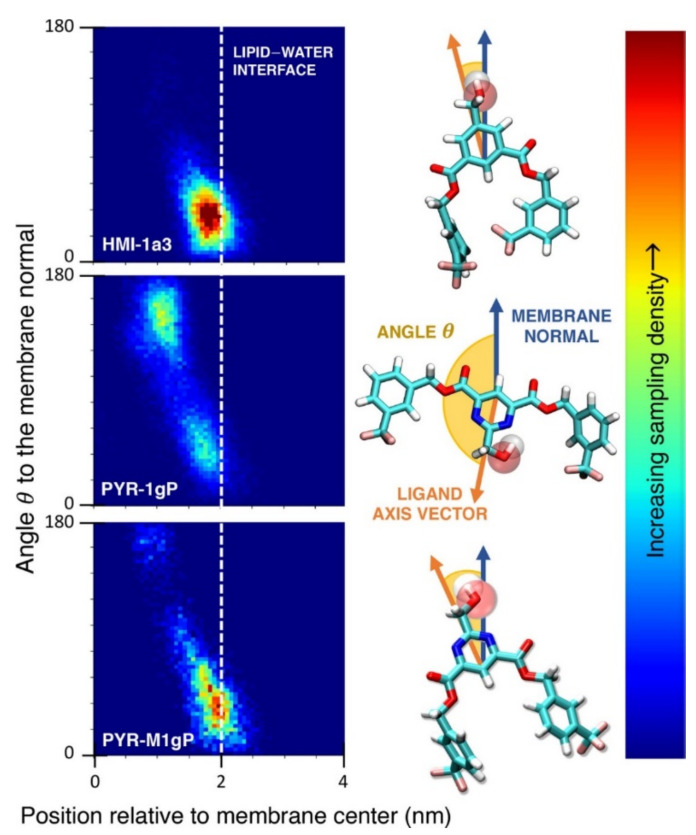
Maps showing orientation-position relations of the hydroxyl groups of HMI-1a3 and PYR-1gP. Reproduced with permission from ref. [336].

A series of experimental assays were carried out on different DAG mimics based on hydrophobic isophthalic acid derivatives (HMI**s**) [1031,1032,1033,1034]. Among these, the HMI known as HMI-1a3 (Figure 25) was found to be a particularly effective activator of PKC, however its application is limited due to its low solubility. An analog of HMI-1a3 with a phenyl ring substituted with pyrimidine (PYR-1gP) was synthesized that, unlike HMI-1a3, had an acceptable solubility profile, but this was found in experimental assays not to be effective as a PKC activator. An MD simulation study was carried out by Lautala et al., to determine the cause of this; they found that one of the reasons for this observed low potency to be a drastic change in the orientation of the functional group involved in binding from exposed to the water phase to embedded into the bilayer; this results from the formation of an internal H-bond that increase its effective hydrophobicity [336].

#### 6.4.3. The Binding Domains of PIPs

Phosphatidylinositols (PIPs) are a class of lipid of particular importance in cellular signaling [1035]. There are 8 PIP headgroup types characterized by different behaviors at the membrane surface, e.g., orientation in the membrane [1036]. There are 14 known protein domains that bind PIPs [1037,1038,1039] including: (1) pleckstrin homology (PH), (2) FYVE (the first letter of the first four proteins in which domain was identified), (3) phox homology (PX), (4) epsin N-terminal homology (ENTH), (5) FERM (F stands for 4.1 protein, E for ezrin, R for radixin and M for moesin), (6) PROPPINS (b-propellers that bind polyphosphoinositides), (7) Tubby, and (8) BAR (name from first letter of proteins: Bin, Amphiphysin, Rvs) domains and others not discussed in this paper.

The PH domain is the most common PIP binding domain, there are at least 329 different PH domains present in 284 human proteins [1040]. The PH domain is built from 100–150 residues and binds various PIPs. Unsurprisingly, it is the most frequently studied among PIP binding domains. A few MD simulation studies provide insight into PH domain interactions with PIP headgroups and details of interactions with lipids in the bilayer in addition to the orientation of the domain towards the membrane surface [1041,1042,1043,1044,1045,1046,1047,1048] or characterized dynamics of bound domain at the membrane surface [1030]; for example, Yamamoto et al., characterized PH domains originating from 13 different proteins [1049]. Interactions of PH domains with lipid bilayers were also studied through computational free energy calculations. For example, it has been shown that the PH domain from the GRP1 protein has stronger binding to a lipid bilayer with PIP3 than a bilayer with PIP2 [1050]. In another study of the GRP1 PH domain, it was shown that in the canonical binding mode, the free energy difference between the bound and unbound state is about 9 kcal/mol, while in an alternate, non–canonical, binding mode, it is about 7 kcal/mol [1047]. Moreover, it has been shown that the GRP1 PH domain has the ability to bind multiple PIP3 molecules simultaneously, increasing the strength of the binding to the lipid bilayer [1051]. Finally, MD simulations have demonstrated that the binding of the PH domain to the membrane could possibly be facilitated by phosphatidylserine; however, for stable binding, PIP is necessary [1048].

The ACAP1 protein, recently studied with MD simulations, is in possession of two PIP binding domains: the BAR and PH domains [1052]; the orientation of the PH domain at the membrane surface is affected by the BAR domain. The PH domain of ACAP1 has two binding pockets for PIP2 headgroups (pocket 1 and pocket 2); free energy calculations determined that the PIP2 occupancy of pocket 1 decreases the free energy of binding to the membrane by 1 kcl/mol in comparison to when PIP2 is not present, while the occupancy of pocket 2 decreases the free energy of binding by 3.5 kcl/mol.

The over-expression of Grb2-associated binding protein 1 (GAB1) occurs in numerous cancers, thus drugs designed to inhibit the activity of GAB1 are studied, including drugs that target the PH domain. Extensive modeling studies, including virtual screening of five million compounds and MD simulations of the five most promising molecules, have demonstrated that selected molecules strongly bind to the PH domain and induced significant changes to the protein conformation [1053]. Next, it has been shown experimentally that these potential drugs have tumor-specific cytotoxicity against two breast cancer cell lines. The ceramide transfer protein contains both PH and START domains, and dimerization of these domains leads to protein inhibition. Docking calculations, combined with MD simulation, allowed for the selection of a few possible protein inhibitors, which bind to the PH–START domain interface, increasing the strength of the association between these two domains [1054]. The protein AKT1 kinase, also in possession of one or more PH domains, has been studied, using docking calculations and MD simulation, to understand the effect of the cancerous E17K mutation [1055]. It has been observed that inhibitor 7, known to be ineffective for the case of this mutation being present, is characterized by a much lower affinity towards the mutated version of the protein. Moreover, mutation leads to conformational changes in the protein.

The FYVE domain that contains only 70–80 amino acids, has been identified in 30 human proteins. The FYVE domain only binds PIP3 and not the other PIPs, i.e., it is specific for PIP3. The PX domain is present in 49 human proteins and binds several different PIPs [1056]. Studies of these two domain interactions with lipid bilayers, carried out through MD simulation, have demonstrated that, after binding with a lipid bilayer and PIP3, both domains lose their flexibility that is observed when the domains are in the water phase [1057]. This effect was more pronounced for the FYVE domain. Both domains remained firmly bound to the PIP3 molecule and both domains penetrate the bilayer core, the FYVE domain via the N-terminal hydrophobic loop and the PX domain via two loops: α1α2 and β1β2. In another MD simulation study, phospholipase D2 (PLD2) that has both PX and PH domains, was shown to interact with PIP2 predominantly via these specific domains [1058]. Moreover, a second anionic lipid, POPA, was found to participate in the protein-membrane interactions.

**Figure 26:**
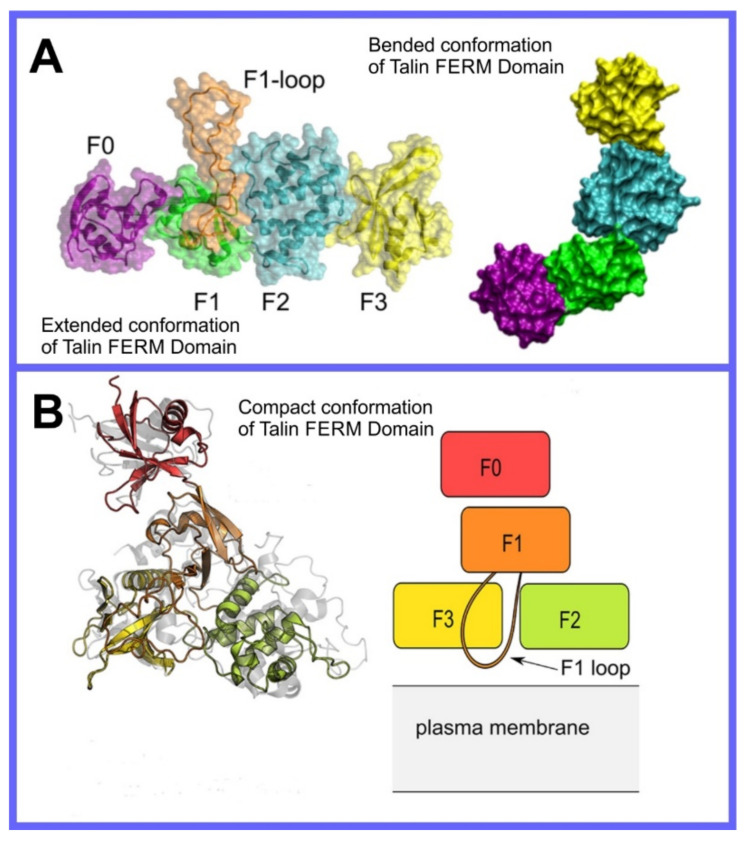
Conformation of talin FERM domain (**A**) extended conformation from crystal structure (PBD ID 3IVF) and after CG simulations [1059], (**B**) compact conformation [1061].

The FERM domain was included in a few MD simulation studies of the proteins talin [1059,1060,1061] and focal adhesion kinase (FAK) [1062,1063]. Studies of FAK showed a strong effect of PIP2 on the protein orientation at the membrane surface [1063]. Studies of talin elucidated how talin, in cooperation with PIP2, affects the interaction of dimers of the transmembrane helices of integrins leading to dimer dissociation, the first step in integrin activation [1059,1060]. MD simulation also found a change in the FERM domain conformation [1059]; the FERM domain is composed of four units F0, F1, F2 and F3 which are organized in a linear fashion (Figure 26) as seen in its crystal structure (PBD ID 3IVF) [1064] that is, however, missing loop F1 which was approximated computationally. Due to the presence of the loop F1, the FERM domain became V-shaped (Figure 26). Additionally, a study that combined MD simulations with multiple experimental methods, demonstrated that FERM in talin is more compact and nonlinear [1061] (Figure 26).

The mechanism through which PROPPINS docks to the membrane has been studied using coarse-grained and atomistic MD simulations; the study demonstrated the importance of the loop 6CD for protein binding to the membrane [1065]; a combination of experimental and theoretical studies revealed the presence of two phosphoinositide binding sites in the β-propeller domain [1066]. Coarse-grained MD simulations were used in studies of the aggregation of the ENTH domains at membrane vesicles and membrane tubes, i.e., cylinders formed from lipid membranes [1067]. These studies found evidence that the formation of dimers and larger oligomers occurs within membrane tubes. Finally, MD simulation studies of the tubby domain have demonstrated that it is in possession of two binding sites for PIP2 [1068].

#### 6.4.4. Other Examples

Coagulation factor X is one of several proteins involved in the initiation of the coagulation cascade; its activity is dramatically increased when it binds to a membrane that contains negatively charged lipids, in particular phosphatidylserines. The GLA domain is responsible for the binding of factor X to the lipid bilayers, its characteristic feature is posttranslational modification of glutamic acid residues, which transform the glutamic acid sidechain into γ-carboxyglutamic acid (GLA); GLA is in possession of two carboxylic groups with the ability to strongly bind divalent cations, predominately Ca^++^. Through MD simulation, evidence has been found that GLA residues with bound Ca^++^ ions bind directly to phosphatidylserine [1069]. Altogether, 7 GLA residues participate in interactions with lipid bilayers and short loops insert three hydrophobic residues directly into the bilayer core.

Protein toxins, including bacterial toxin, have specific requirements to recognize and enter cells [1070], i.e., certain molecules must be present in the membrane. Cholera toxin is a protein, which requires gangliosides, e.g., GM1, to be present in the membrane in order for it to bind and is a potential drug target; for example, peptide mimicking GM1 carbohydrates prevent the binding of toxin to intestinal cells [337]. Through MD simulation, the mechanism of toxin docking to the lipid bilayer has been studied, including its interactions with lipids, in particular GM1 and the effects of the toxin binding on the overall properties of the lipid bilayer [525,1071]. Docking studies have proposed three possible drug candidates with sufficient potency to prevent the binding of the toxin with ganglioside; MD simulations were used next to evaluate the stability of the binding of drug candidates with toxin [1072]. Two compounds, A6225 and A16503, formed a stable complex with the toxin. Cardiotoxin CTII, a component of cobra venom, is known to disrupt mitochondrial membranes. Through MD simulation, the interactions of CTII with a lipid bilayer composed of lipids present in the outer mitochondrial membrane [1073] were elucidated. In particular, residues involved in interactions with cardiolipin were identified.

The enzymes responsible for the metabolism and transportation of lipids, are often peripheral membrane proteins not explored as a drug target although proposed as a target for antiviral therapy against flaviviruses [1074]. Examples of MD simulation studies of enzymes that catalyze the metabolism of lipids include monogalactosyl-diacyl-glycerol synthase 1 [1075], phosphatidylinositol phospholipase Cγ and its cancerous mutation [1076], and lysophosphatidic acid acyltransferase [1077]. Glutathione peroxidase 4 is an enzyme responsible for the removal of lipid hydroperoxides, thus preventing lipid oxidation; MD simulations have recently been used to elucidate the process through which a substrate enters into the active pocket of the enzyme from the membrane surface [1078]. Lipid transporters are proteins that shuttle lipids between two separate membranes, thus acting as peripheral membrane proteins when loading/unloading their cargo and water-soluble proteins when transporting it through the cytoplasm. Lipid transporters studied through MD simulation include the mammalian phosphatidylinositol transfer protein; these studies provided insight into the mechanism and energetics of the loading/unloading of PIPs into the binding cavity of the protein [1079] and studies of the UPS1 protein transporting phosphatidic acid between the inner and outer mitochondrial membrane [1080].

As a large group of peripheral membrane proteins recognize specific lipids, it is also possible to develop drugs that bind to specific lipids, thus preventing the recognition of these lipids by specific peripheral membrane proteins. An example of such a strategy is the study of siramesine, a small compound characterized by its high affinity to phosphatidic acid [335].

## 7. Conclusions

Through a detailed discussion of how MD simulation can be used to elucidate the roles the interaction with lipid membranes play in drug action, we hope we have provided the reader with an intuitive understanding of the broader landscape within which drug molecules act, beyond the limiting paradigm of ADME and “docking and scoring”. Xenobiotics are foreign molecules travelling through the body, uninvited intruders that become involved in the complex melee that is physiology on the molecular length scale. A drug molecule is a xenobiotic with a specific purpose, designed by its maker, like a foreign agent with a mission to perform a specific task travelling across the countryside to reach the city where they must perform it. In the previous review paper, we discussed the role MD simulation can play in providing mechanistic insight relevant to the development of the mechanisms through which this agent can reach their destination; now we have discussed how MD simulation can aid in the design of a more complex set of tasks for the agent than possible within the confines of the “docking and scoring” paradigm. Through MD simulation, (1) drug molecules can be rationally designed to perform more complex tasks than merely fitting to a specific protein and (2) even if the target is the active site of a specific protein, far more is involved in the determination of the effective affinity of a specific drug molecule structure for a specific active site than can be investigated through the “lock and key” paradigm. While there are many possible factors other than the role played by lipid membrane interactions that MD simulation can address, we hope that this provides a case study that expands the intuitive picture the reader has of the scope of computational drug design and the role the specific toolkit of MD simulation can play.

Lipid membranes are fundamental structures with no less complex or important a role in molecular level physiology than proteins; the mechanisms through which small drug molecules can interact with them are equally complex. Drug molecules must cross them, can affect their properties through collective action, and can have their interactions with target proteins mediated by them; a comprehensive inclusion of the role the drug structure plays in its interaction with lipid membranes and the use of MD simulation to model this interaction is clearly a component of the future of drug design.

In the beginning of the paper, we mentioned that we can envision a time in the future when, through the combined solution of the human proteome and the general structure from sequence problem, a program can exist that could input any molecular structure and output its binding affinity for every active site of every protein in the human proteome. Hopefully, when reading this review paper, the reviewer can see that even this fantastic device would merely be one component of the advanced computational toolkit of drug design of the future. This information would form a starting foundation that then would be combined with MD simulation able to investigate the broader context of the protein environment, or possible modes of action of a specific drug molecule that involve different elements of physiology than active sites of proteins. The lipid membrane is an important component of this where we have shown that MD simulation can play a leading role. As in all cases, the computational effort must be matched with complementary experimental techniques that themselves are undergoing revolutionary upgrades as we speak. As recent events have shown, the health challenges to humanity are mounting, but so is our arsenal in combating them and MD simulation as a component of computational drug design will grow in importance, with the increase in computational power and data stored.

## Data Availability

Not applicable.

## References

[1] Jumper J., Evans R., Pritzel A., Green T., Figurnov M., Ronneberger O., Tunyasuvunakool K., Bates R., Žídek A., Potapenko A. (2021). Highly accurate protein structure prediction with AlphaFold. Nature.

[2] Adhikari S., Nice E.C., Deutsch E.W., Lane L., Omenn G.S., Pennington S.R., Paik Y.-K., Overall C.M., Corrales F.J., Cristea I.M. (2020). A high-stringency blueprint of the human proteome. Nat. Commun..

[3] Bunker A., Róg T. (2020). Mechanistic Understanding From Molecular Dynamics Simulation in Pharmaceutical Research 1: Drug Delivery. Front. Mol. Biosci..

[4] Jorgensen W.L. (1991). Rusting of the lock and key model for protein-ligand binding. Science.

[5] Tutone M., Almerico A.M., Labrou N.E. (2020). The In Silico Fischer Lock-and-Key Model: The Combined Use of Molecular Descriptors and Docking Poses for the Repurposing of Old Drugs BT—Targeting Enzymes for Pharmaceutical Development: Methods and Protocols. Targeting Enzymes for Pharmaceutical Development.

[6] Balani S.K., Miwa G.T., Gan L.-S., Wu J.-T., Lee F.W. (2005). Strategy of Utilizing In Vitro and In Vivo ADME Tools for Lead Optimization and Drug Candidate Selection. Curr. Top. Med. Chem..

[7] Su C., Liu Y., Li R., Wu W., Fawcett J.P., Gu J. (2019). Absorption, distribution, metabolism and excretion of the biomaterials used in Nanocarrier drug delivery systems. Adv. Drug Deliv. Rev..

[8] Nirogi R., Bhyrapuneni G., Muddana N.R., Manoharan A., Shinde A.K., Mohammed A.R., Padala N.P., Ajjala D.R., Subramanian R., Palacharla V.R.C. (2020). Absorption, distribution, metabolism, excretion (ADME), drug-drug interaction potential and prediction of human pharmacokinetics of SUVN-G3031, a novel histamine 3 receptor (H3R) inverse agonist in clinical development for the treatment of narcolepsy. Eur. J. Pharm. Sci..

[9] Hall J., Matos S., Gold S., Severino L.S. (2018). The paradox of sustainable innovation: The ‘Eroom’ effect (Moore’s law backwards). J. Clean. Prod..

[10] Limongelli V. (2020). Ligand binding free energy and kinetics calculation in 2020. Wiley Interdiscip. Rev. Comput. Mol. Sci..

[11] Wong C.F. (2014). Molecular simulation of drug-binding kinetics. Mol. Simul..

[12] Schuetz D.A., Bernetti M., Bertazzo M., Musil D., Eggenweiler H.M., Recanatini M., Masetti M., Ecker G.F., Cavalli A. (2019). Predicting Residence Time and Drug Unbinding Pathway through Scaled Molecular Dynamics. J. Chem. Inf. Model..

[13] Bruce N.J., Ganotra G.K., Richter S., Wade R.C. (2019). KBbox: A Toolbox of Computational Methods for Studying the Kinetics of Molecular Binding. J. Chem. Inf. Model..

[14] Ahn S.H., Jagger B.R., Amaro R.E. (2020). Ranking of ligand binding kinetics using a weighted ensemble approach and comparison with a multiscale milestoning approach. J. Chem. Inf. Model..

[15] Efremov R.G. (2021). Dynamic “molecular portraits” of biomembranes drawn by their lateral nanoscale inhomogeneities. Int. J. Mol. Sci..

[16] Watson H. (2015). Biological membranes. Essays Biochem..

[17] Luckey M. (2008). Membrane Structural Biology: With Biochemical and Biophysical Foundations.

[18] Nicolson G.L. (2014). The Fluid—Mosaic Model of Membrane Structure: Still relevant to understanding the structure, function and dynamics of biological membranes after more than 40 years. Biochim. Biophys. Acta-Biomembr..

[19] Enkavi G., Javanainen M., Kulig W., Róg T., Vattulainen I. (2019). Multiscale Simulations of Biological Membranes: The Challenge to Understand Biological Phenomena in a Living Substance. Chem. Rev..

[20] Friedman R., Khalid S., Aponte-Santamaría C., Arutyunova E., Becker M., Boyd K.J., Christensen M., Coimbra J.T.S., Concilio S., Daday C. (2018). Understanding Conformational Dynamics of Complex Lipid Mixtures Relevant to Biology. J. Membr. Biol..

[21] Muller M.P., Jiang T., Sun C., Lihan M., Pant S., Mahinthichaichan P., Trifan A., Tajkhorshid E. (2019). Characterization of Lipid-Protein Interactions and Lipid-Mediated Modulation of Membrane Protein Function through Molecular Simulation. Chem. Rev..

[22] Miranda W.E., Ngo V.A., Perissinotti L.L., Noskov S.Y. (2017). Computational membrane biophysics: From ion channel interactions with drugs to cellular function. Biochim. Biophys. Acta-Proteins Proteomics.

[23] Oakes V., Domene C. (2017). Chapter 2 Molecular Dynamics Simulations: Principles and Applications for the Study of Membrane Proteins. Computational Biophysics of Membrane Proteins.

[24] Vattulainen I., Róg T. (2016). Lipid membranes: Theory and simulations bridged to experiments. Biochim. Biophys. Acta-Biomembr..

[25] Loschwitz J., Olubiyi O.O., Hub J.S., Strodel B., Poojari C.S. (2020). Computer simulations of protein–membrane systems. Progress in Molecular Biology and Translational Science.

[26] Manna M., Róg T., Vattulainen I. (2014). The challenges of understanding glycolipid functions: An open outlook based on molecular simulations. Biochim. Biophys. Acta-Mol. Cell Biol. Lipids.

[27] Vattulainen I., Róg T. (2011). Lipid simulations: A perspective on lipids in action. Cold Spring Harb. Perspect. Biol..

[28] Salo-Ahen O.M.H., Alanko I., Bhadane R., Bonvin A.M.J.J., Honorato R.V., Hossain S., Juffer A.H., Kabedev A., Lahtela-kakkonen M., Larsen A.S. (2021). Molecular Dynamics Simulations in Drug Discovery and Pharmaceutical Development. Processes.

[29] 2Zhao B., Li W., Sun L., Fu W. (2020). The Use of Computational Approaches in the Discovery and Mechanism Study of Opioid Analgesics. Front. Chem..

[30] Salmaso V., Jacobson K.A. (2020). In silico drug design for purinergic gpcrs: Overview on molecular dynamics applied to adenosine and p2y receptors. Biomolecules.

[31] Zou Y., Ewalt J., Ng H.L. (2019). Recent insights from molecular dynamics simulations for g protein-coupled receptor drug discovery. Int. J. Mol. Sci..

[32] Yuan X., Xu Y. (2018). Recent trends and applications of molecular modeling in GPCR–Ligand recognition and structure-based drug design. Int. J. Mol. Sci..

[33] Mayne C.G., Arcario M.J., Mahinthichaichan P., Baylon J.L., Vermaas J.V., Navidpour L., Wen P.-C., Thangapandian S., Tajkhorshid E. (2016). The cellular membrane as a mediator for small molecule interaction with membrane proteins. Biochim. Biophys. Acta-Biomembr..

[34] Di Meo F., Fabre G., Berka K., Ossman T., Chantemargue B., Paloncýová M., Marquet P., Otyepka M., Trouillas P. (2016). In silico pharmacology: Drug membrane partitioning and crossing. Pharmacol. Res..

[35] Cramariuc O., Rogl T., Vattulainen I. (2012). Drug-lipid membrane interaction mechanisms revealed through molecular simulations. Curr. Phys. Chem..

[36] Lopes D., Jakobtorweihen S., Nunes C., Sarmento B., Reis S. (2017). Shedding light on the puzzle of drug-membrane interactions: Experimental techniques and molecular dynamics simulations. Prog. Lipid Res..

[37] Filipe H.A.L., Cardoso R.M.S., Loura L.M.S., Moreno M.J., Chattopadhyay A. (2017). Interaction of Amphiphilic Molecules with Lipid Bilayers: Kinetics of Insertion, Desorption and Translocation. Membrane Organization and Dynamics.

[38] Martinotti C., Ruiz-Perez L., Deplazes E., Mancera R.L. (2020). Molecular Dynamics Simulation of Small Molecules Interacting with Biological Membranes. Chem. Phys. Chem..

[39] 3Seddon A.M., Casey D., Law R.V., Gee A., Templer R.H., Ces O. (2009). Drug interactions with lipid membranes. Chem. Soc. Rev..

[40] Kopec W., Telenius J., Khandelia H. (2013). Molecular dynamics simulations of the interactions of medicinal plant extracts and drugs with lipid bilayer membranes. FEBS J..

[41] Payandeh J., Volgraf M. (2021). Ligand binding at the protein-lipid interface: Strategic considerations for drug design. Nat. Rev. Drug Discov..

[42] Katiyar R.S., Jha P.K. (2018). Molecular simulations in drug delivery: Opportunities and challenges. WIREs Comput. Mol. Sci..

[43] Lee H. (2020). Molecular simulations of PEGylated biomolecules, liposomes, and nanoparticles for drug delivery applications. Pharmaceutics.

[44] Casalini T. (2021). Not only in silico drug discovery: Molecular modeling towards in silico drug delivery formulations. J. Control. Release.

[45] Shariatinia Z., Ahmad Taher Azar (2021). Chapter 10—Molecular Dynamics Simulations on Drug Delivery Systems. Modeling and Control of Drug Delivery Systems.

[46] Mondal J. (2019). A brief appraisal of computational modeling of antimicrobial peptides’ activity. Drug Dev. Res..

[47] De Paula V.S., Valente A.P. (2018). A dynamic overview of antimicrobial peptides and their complexes. Molecules.

[48] Lipkin R., Lazaridis T. (2017). Computational studies of peptide-induced membrane pore formation. Philos. Trans. R. Soc. B Biol. Sci..

[49] Chen C.H., Melo M.C., Berglund N., Khan A., de la Fuente C., Ulmschneider J.P., Ulmschneider M.B. (2020). Understanding and modelling the interactions of peptides with membranes: From partitioning to self-assembly. Curr. Opin. Struct. Biol..

[50] Bertrand B., Garduño-Juárez R., Munoz-Garay C. (2021). Estimation of pore dimensions in lipid membranes induced by peptides and other biomolecules: A review. Biochim. Biophys. Acta-Biomembr..

[51] Der Torossian Torres M., De La Fuente-Nunez C. (2019). Reprogramming biological peptides to combat infectious diseases. Chem. Commun..

[52] Cardoso M.H., Orozco R.Q., Rezende S.B., Rodrigues G., Oshiro K.G.N., Cândido E.S., Franco O.L. (2020). Computer-Aided Design of Antimicrobial Peptides: Are We Generating Effective Drug Candidates?. Front. Microbiol..

[53] Palmer N., Maasch J.R.M.A., Torres M.D.T., de la Fuente-Nunez C. (2021). Molecular Dynamics for Antimicrobial Peptide Discovery. Infect. Immun..

[54] Aronica P.G.A., Reid L.M., Desai N., Li J., Fox S.J., Yadahalli S., Essex J.W., Verma C.S. (2021). Computational Methods and Tools in Antimicrobial Peptide Research. J. Chem. Inf. Model..

[55] Lin X., Li X., Lin X. (2020). A review on applications of computational methods in drug screening and design. Molecules.

[56] Li J., Fu A., Zhang L. (2019). An Overview of Scoring Functions Used for Protein–Ligand Interactions in Molecular Docking. Interdiscip. Sci. Comput. Life Sci..

[57] Lee B.L., Kuczera K. (2018). Simulating the free energy of passive membrane permeation for small molecules. Mol. Simul..

[58] Armacost K.A., Riniker S., Cournia Z. (2020). Exploring Novel Directions in Free Energy Calculations. J. Chem. Inf. Model..

[59] Boittier E.D., Tang Y.Y., Buckley M.E., Schuurs Z.P., Richard D.J., Gandhi N.S. (2020). Assessing molecular docking tools to guide targeted drug discovery of cd38 inhibitors. Int. J. Mol. Sci..

[60] Lee T.-S., Allen B.K., Giese T.J., Guo Z., Li P., Lin C., McGee T.D., Pearlman D.A., Radak B.K., Tao Y. (2020). Alchemical Binding Free Energy Calculations in AMBER20: Advances and Best Practices for Drug Discovery. J. Chem. Inf. Model..

[61] Braun E., Gilmer J., Mayes H.B., Mobley D.L., Monroe J.I., Prasad S., Zuckerman D.M. (2019). Best Practices for Foundations in Molecular Simulations [Article v1.0]. Living J. Comput. Mol. Sci..

[62] Hollingsworth S.A., Dror R.O. (2018). Molecular Dynamics Simulation for All. Neuron.

[63] Lazim R., Suh D., Choi S. (2020). Advances in molecular dynamics simulations and enhanced sampling methods for the study of protein systems. Int. J. Mol. Sci..

[64] Childers M.C., Daggett V. (2017). Insights from molecular dynamics simulations for computational protein design. Mol. Syst. Des. Eng..

[65] Hospital A., Goñi J.R., Orozco M., Gelpí J.L. (2015). Molecular dynamics simulations: Advances and applications. Adv. Appl. Bioinform. Chem..

[66] Pak A.J., Voth G.A. (2018). Advances in coarse-grained modeling of macromolecular complexes. Curr. Opin. Struct. Biol..

[67] Kmiecik S., Gront D., Kolinski M., Wieteska L., Dawid A.E., Kolinski A. (2016). Coarse-Grained Protein Models and Their Applications. Chem. Rev..

[68] Singh N., Li W. (2019). Recent Advances in Coarse-Grained Models for Biomolecules and Their Applications. Int. J. Mol. Sci..

[69] Joshi S.Y., Deshmukh S.A. (2020). A review of advancements in coarse-grained molecular dynamics simulations. Mol. Simul..

[70] Arnarez C., Uusitalo J.J., Masman M.F., Ingólfsson H.I., De Jong D.H., Melo M.N., Periole X., De Vries A.H., Marrink S.J. (2015). Dry martini, a coarse-grained force field for lipid membrane simulations with implicit solvent. J. Chem. Theory Comput..

[71] Huang K. (2001). Introduction to Statistical Physics.

[72] 7Landau D.P., Binder K. (2014). A Guide to Monte Carlo Simulations in Statistical Physics.

[73] Paquet E., Viktor H.L. (2015). Molecular dynamics, monte carlo simulations, and langevin dynamics: A computational review. Biomed. Res. Int..

[74] Chen J. (2018). The Development and Comparison of Molecular Dynamics Simulation and Monte Carlo Simulation. IOP Conf. Ser. Earth Environ. Sci..

[75] 75Binder K. (1995). Monte Carlo and Molecular Dynamics Simulations in Polymer Science.

[76] Bunker A., Dünweg B. (2001). Parallel excluded volume tempering for polymer melts. Phys. Rev. E Stat. Nonlin Soft Matter Phys..

[77] Gray G.M., Ma N., Wagner C.E., van der Vaart A. (2017). Molecular dynamics simulations and molecular flooding studies of the retinoid X-receptor ligand binding domain. J. Mol. Model..

[78] Maragliano L., Vanden-Eijnden E. (2006). A temperature accelerated method for sampling free energy and determining reaction pathways in rare events simulations. Chem. Phys. Lett..

[79] Oshima H., Re S., Sugita Y. (2019). Replica-Exchange Umbrella Sampling Combined with Gaussian Accelerated Molecular Dynamics for Free-Energy Calculation of Biomolecules. J. Chem. Theory Comput..

[80] Sugita Y., Okamoto Y. (1999). Replica exchange molecular dynamics method for protein folding simulation. Chem. Phys. Lett..

[81] Swendsen R.H., Wang J.-S. (1986). Replica Monte Carlo Simulation of Spin-Glasses. Phys. Rev. Lett..

[82] Yan Q., de Pablo J.J. (1999). Hyper-parallel tempering Monte Carlo: Application to the Lennard-Jones fluid and the restricted primitive model. J. Chem. Phys..

[83] Fukunishi H., Watanabe O., Takada S. (2002). On the Hamiltonian replica exchange method for efficient sampling of biomolecular systems: Application to protein structure prediction. J. Chem. Phys..

[84] Wang F., Landau D.P. (2001). Efficient, Multiple-Range Random Walk Algorithm to Calculate the Density of States. Phys. Rev. Lett.

[85] Kim J., Straub J.E., Keyes T. (2006). Statistical-Temperature Monte Carlo and Molecular Dynamics Algorithms. Phys. Rev. Lett..

[86] Junghans C., Perez D., Vogel T. (2014). Molecular Dynamics in the Multicanonical Ensemble: Equivalence of Wang–Landau Sampling, Statistical Temperature Molecular Dynamics, and Metadynamics. J. Chem. Theory Comput..

[87] Bussi G., Laio A. (2020). Using metadynamics to explore complex free-energy landscapes. Nat. Rev. Phys..

[88] Laio A., Parrinello M. (2002). Escaping free-energy minima. Proc. Natl. Acad. Sci. USA.

[89] Valsson O., Tiwary P., Parrinello M. (2016). Enhancing Important Fluctuations: Rare Events and Metadynamics from a Conceptual Viewpoint. Annu. Rev. Phys. Chem..

[90] Invernizzi M., Parrinello M. (2020). Rethinking Metadynamics: From Bias Potentials to Probability Distributions. J. Phys. Chem. Lett..

[91] Bussi G., Laio A., Tiwary P., Andreoni W., Yip S. (2018). Metadynamics: A Unified Framework for Accelerating Rare Events and Sampling Thermodynamics and Kinetics BT. Handbook of Materials Modeling: Methods: Theory and Modeling.

[92] Lange O.E., Schafer L.V., Grubmuller H. (2006). Flooding in GROMACS: Accelerated barrier crossings in molecular dynamics. J. Comput. Chem..

[93] Kästner J. (2011). Umbrella sampling: Umbrella sampling. Wiley Interdiscip. Rev. Comput. Mol. Sci..

[94] Sotomayor M. (2015). Computational exploration of single-protein mechanics by steered molecular dynamics. AIP Conf. Proc..

[95] Do P.C., Lee E.H., Le L. (2018). Steered Molecular Dynamics Simulation in Rational Drug Design. J. Chem. Inf. Model..

[96] Kumar S., Rosenberg J.M., Bouzida D., Swendsen R.H., Kollman P.A. (1992). THE weighted histogram analysis method for free-energy calculations on biomolecules. I. The method. J. Comput. Chem..

[97] Ferrenberg A.M., Swendsen R.H. (1989). Optimized Monte Carlo data analysis. Phys. Rev. Lett..

[98] Ferrenberg A.M., Swendsen R.H. (1988). New Monte Carlo technique for studying phase transitions. Phys. Rev. Lett..

[99] Bunker A., Gaulin B.D., Kallin C. (1993). Multiple-histogram Monte Carlo study of the Ising antiferromagnet on a stacked triangular lattice. Phys. Rev. B.

[100] Nandy B., Maiti P.K., Bunker A. (2013). Force Biased Molecular Dynamics Simulation Study of Effect of Dendrimer Generation on Interaction with DNA. J. Chem. Theory Comput..

[101] Park S., Kim T., Im W. (2012). Transmembrane helix assembly by window exchange umbrella sampling. Phys. Rev. Lett..

[102] Lüdemann S.K., Lounnas V., Wade R.C. (2000). How do substrates enter and products exit the buried active site of cytochrome P450cam? 2. Steered molecular dynamics and adiabatic mapping of substrate pathways11Edited by J. Thornton. J. Mol. Biol..

[103] Deflorian F., Perez-Benito L., Lenselink E.B., Congreve M., van Vlijmen H.W.T., Mason J.S., Graaf C.D., Tresadern G. (2020). Accurate Prediction of GPCR Ligand Binding Affinity with Free Energy Perturbation. J. Chem. Inf. Model..

[104] Jarzynski C. (1997). Nonequilibrium equality for free energy differences. Phys. Rev. Lett..

[105] Hartmann C., Schütte C., Zhang W. (2019). Jarzynski’s Equality, Fluctuation Theorems, and Variance Reduction: Mathematical Analysis and Numerical Algorithms. J. Stat. Phys..

[106] Lüdemann S.K., Lounnas V., Wade R.C. (2000). How do substrates enter and products exit the buried active site of cytochrome P450cam? 1. Random expulsion molecular dynamics investigation of ligand access channels and mechanisms. J. Mol. Biol..

[107] Kokh D.B., Amaral M., Bomke J., Grädler U., Musil D., Buchstaller H.P., Dreyer M.K., Frech M., Lowinski M., Vallee F. (2018). Estimation of drug-target residence times by τ-random acceleration molecular dynamics simulations. J. Chem. Theory Comput..

[108] Fu H., Chen H., Wang X., Chai H., Shao X., Cai W., Chipot C. (2020). Finding an optimal pathway on a multidimensional free-energy landscape. J. Chem. Inf. Model..

[109] Lev B., Allen T.W. (2020). Simulating ion channel activation mechanisms using swarms of trajectories. J. Comput. Chem..

[110] Patel S.J., Van Lehn R.C. (2021). Analysis of Charged Peptide Loop-Flipping across a Lipid Bilayer Using the String Method with Swarms of Trajectories. J. Phys. Chem. B.

[111] Kirkwood J.G. (1935). Statistical Mechanics of Fluid Mixtures. J. Chem. Phys..

[112] Zwanzig R.W. (1954). High-Temperature Equation of State by a Perturbation Method. I. Nonpolar Gases. J. Chem. Phys..

[113] Song L.F., Merz K.M. (2020). Evolution of Alchemical Free Energy Methods in Drug Discovery. J. Chem. Inf. Model..

[114] Schindler C.E.M., Baumann H., Blum A., Böse D., Buchstaller H.-P., Burgdorf L., Cappel D., Chekler E., Czodrowski P., Dorsch D. (2020). Large-Scale Assessment of Binding Free Energy Calculations in Active Drug Discovery Projects. J. Chem. Inf. Model..

[115] Bennett C.H. (1976). Efficient estimation of free energy differences from Monte Carlo data. J. Comput. Phys..

[116] Shirts M.R., Chodera J.D. (2008). Statistically optimal analysis of samples from multiple equilibrium states. J. Chem. Phys..

[117] Noé F., Tkatchenko A., Müller K.R., Clementi C. (2020). Machine learning for molecular simulation. Annu. Rev. Phys. Chem..

[118] Scheen J., Wu W., Mey A.S.J.S., Tosco P., Mackey M., Michel J. (2020). Hybrid alchemical free Energy/Machine-Learning methodology for the computation of hydration free energies. J. Chem. Inf. Model..

[119] Gebhardt J., Kiesel M., Riniker S., Hansen N. (2020). Combining molecular dynamics and machine learning to predict self-solvation free energies and limiting activity coefficients. J. Chem. Inf. Model..

[120] MacCallum J.L., Tieleman D.P. (2008). Chapter 8 Interactions between Small Molecules and Lipid Bilayers. Curr. Top. Membr..

[121] Marrink S.J., Berendsen H.J. (1996). Permeation process of small molecules across lipid mem- branes studied by molecular dynamics simulations. J. Phys. Chem..

[122] Odinokov A., Ostroumov D. (2015). Structural Degradation and Swelling of Lipid Bilayer under the Action of Benzene. J. Phys. Chem. B.

[123] Gupta R., Dwadasi B.S., Rai B. (2016). Molecular Dynamics Simulation of Skin Lipids: Effect of Ceramide Chain Lengths on Bilayer Properties. J. Phys. Chem. B.

[124] Stimson L.M., Vattulainen I., Róg T., Karttunen M. (2005). Exploring the effect of xenon on biomembranes. Cell. Mol. Biol. Lett..

[125] Booker R.D., Sum A.K. (2013). Biophysical changes induced by xenon on phospholipid bilayers. Biochim. Biophys. Acta-Biomembr..

[126] Jedlovszky P., Mezei M. (2000). Calculation of the Free Energy Profile of H_2_O, O_2_, CO, CO_2_, NO, and CHCl_3_ in a Lipid Bilayer with a Cavity Insertion Variant of the Widom Method. J. Am. Chem. Soc..

[127] Wang Y., Tajkhorshid E. (2010). Nitric Oxide Conduction by the Brain Aquaporin AQP4 Yi. Proteins Struct. Funct. Genet..

[128] Bacellar I.O.L., Cordeiro R.M., Mahling P., Baptista M.S., Röder B., Hackbarth S. (2019). Oxygen distribution in the fluid/gel phases of lipid membranes. Biochim. Biophys. Acta-Biomembr..

[129] Dotson R.J., Smith C.R., Bueche K., Angles G., Pias S.C. (2017). Influence of Cholesterol on the Oxygen Permeability of Membranes: Insight from Atomistic Simulations. Biophys. J..

[130] Stepniewski M., Kepczynski M., Jamróz D., Nowakowska M., Rissanen S., Vattulainen I., Róg T. (2012). Interaction of hematoporphyrin with lipid membranes. J. Phys. Chem. B.

[131] Magarkar A., Stepniewski M., Karakas E., Róg T., Yliperttula M., Urtti A., Bunker A. (2012). Molecular modeling of the PEGylated bilayer as a model for the PEGylated liposome surface in the bloodstream. NSTI-Nanotech.

[132] Paloncýová M., Devane R., Murch B., Berka K., Otyepka M. (2014). Amphiphilic drug-like molecules accumulate in a membrane below the head group region. J. Phys. Chem. B.

[133] John T., Thomas T., Abel B., Wood B.R., Chalmers D.K., Martin L.L. (2017). How kanamycin A interacts with bacterial and mammalian mimetic membranes. Biochim. Biophys. Acta-Biomembr..

[134] Lorent J.H., Quetin-Leclercq J., Mingeot-Leclercq M.P. (2014). The amphiphilic nature of saponins and their effects on artificial and biological membranes and potential consequences for red blood and cancer cells. Org. Biomol. Chem..

[135] Sudji I.R., Subburaj Y., Frenkel N., García-Sáez A.J., Wink M. (2015). Membrane disintegration caused by the steroid saponin digitonin is related to the presence of cholesterol. Molecules.

[136] Sreij R., Dargel C., Schweins R., Prévost S., Dattani R., Hellweg T. (2019). Aescin-Cholesterol Complexes in DMPC Model Membranes: A DSC and Temperature-Dependent Scattering Study. Sci. Rep..

[137] Schumann-Gillett A., Mara M.L.O. (2019). Lipid-Based Inhibitors Act Directly on GlyT2. ACS Chem. Biol..

[138] Postila P.A., Vattulainen I., Róg T. (2016). Selective effect of cell membrane on synaptic neurotransmission. Sci. Rep..

[139] Wang C., Ye F., Valardez G.F., Peters G.H., Westh P. (2011). Affinity of four polar neurotransmitters for lipid bilayer membranes. J. Phys. Chem. B.

[140] Pérez-Isidoro R., Ruiz-Suárez J.C. (2016). Calcium and protons affect the interaction of neurotransmitters and anesthetics with anionic lipid membranes. Biochim. Biophys. Acta-Biomembr..

[141] Kulig W., Pasenkiewicz-Gierula M., Róg T. (2015). Topologies, structures and parameter files for lipid simulations in GROMACS with the OPLS-aa force field: DPPC, POPC, DOPC, PEPC, and cholesterol. Data Br..

[142] Kulig W., Pasenkiewicz-Gierula M., Róg T., Róg T. (2016). Cis and trans unsaturated phosphatidylcholine bilayers: A molecular dynamics simulation study. Chem. Phys. Lipids.

[143] Maciejewski A., Pasenkiewicz-Gierula M., Cramariuc O., Vattulainen I., Róg T. (2014). Refined OPLS-AA Force Field for Saturated Phosphatidylcholine Bilayers at Full Hydration. J. Phys. Chem. B.

[144] Lajunen T., Kontturi L.S., Viitala L., Manna M., Cramariuc O., Róg T., Bunker A., Laaksonen T., Viitala T., Murtomäki L. (2016). Indocyanine green loaded liposomes for light triggered drug release. Mol. Pharm..

[145] Kaurola P., Sharma V., Vonk A., Vattulainen I., Róg T. (2016). Distribution and dynamics of quinones in the lipid bilayer mimicking the inner membrane of mitochondria. Biochim. Biophys. Acta-Biomembr..

[146] Tu K., Tarek M., Klein M.L., Scharf D. (1998). Effects of anesthetics on the structure of a phospholipid bilayer: Molecular dynamics investigation of halothane in the hydrated liquid crystal phase of dipalmitoylphosphatidylcholine. Biophys. J..

[147] Pasenkiewicz-Gierula M., Róg T., Grochowski J., Serda P., Czarnecki R., Librowski T., Lochynski S. (2003). Effects of carane derivative local anesthetic on a phsopholipid bilayer studied by molecular dynamics simulation. Biophys. J..

[148] Bemporad D., Luttmann C., Essex J.W. (2005). Behaviour of small solutes and large drugs in a lipid bilayer from computer simulations. Biochim. Biophys. Acta-Biomembr..

[149] Bemporad D., Luttmann C., Essex J.W. (2004). Computer simulation of small moleculle permeation across a lipid bilayer: Dependence on bilayer properties and solute volume, size, and cross-sectional area. Biophys. J..

[150] Abdiche Y.N., Myszka D.G. (2004). Probing the mechanism of drug/lipid membrane interactions using Biacore. Anal. Biochem..

[151] Natesan S., Lukacova V., Peng M., Subramaniam R., Lynch S., Wang Z., Tandlich R., Balaz S. (2014). Structure-Based Prediction of Drug Distribution Across the Headgroup and Core Strata of a Phospholipid Bilayer Using Surrogate Phases. Mol. Pharm..

[152] Juračka J., Šrejber M., Melíková M., Bazgier V., Berka K. (2019). MolMeDB: Molecules on Membranes Database. Database.

[153] Ollila O.H.S., Pabst G. (2016). Atomistic resolution structure and dynamics of lipid bilayers in simulations and experiments. Biochim. Biophys. Acta-Biomembr..

[154] Botan A., Favela-Rosales F., Fuchs P.F.J., Javanainen M., Kanduč M., Kulig W., Lamberg A., Loison C., Lyubartsev A., Miettinen M.S. (2015). Toward atomistic resolution structure of phosphatidylcholine headgroup and glycerol backbone at different ambient conditions. J. Phys. Chem. B.

[155] Catte A., Girych M., Javanainen M., Loison C., Melcr J., Miettinen M.S., Monticelli L., Määttä J., Oganesyan V.S., Ollila O.H.S. (2016). Molecular electrometer and binding of cations to phospholipid bilayers. Phys. Chem. Chem. Phys..

[156] Antila H., Buslaev P., Favela-Rosales F., Ferreira T.M., Gushchin I., Javanainen M., Kav B., Madsen J.J., Melcr J., Miettinen M.S. (2019). Headgroup Structure and Cation Binding in Phosphatidylserine Lipid Bilayers. J. Phys. Chem. B.

[157] Owen M.C., Karner A., Šachl R., Preiner J., Amaro M., Vácha R. (2019). Force Field Comparison of GM1 in a DOPC Bilayer Validated with AFM and FRET Experiments. J. Phys. Chem. B.

[158] Klauda J.B. (2021). Considerations of Recent All-Atom Lipid Force Field Development. J. Phys. Chem. B.

[159] Shih A.Y., Hack M., Mirzadegan T. (2020). Impact of Protein Preparation on Resulting Accuracy of FEP Calculations. J. Chem. Inf. Model..

[160] Tsai H.-C., Tao Y., Lee T.-S., Merz K.M., York D.M. (2020). Validation of Free Energy Methods in AMBER. J. Chem. Inf. Model..

[161] Capelli R., Gardin A., Empereur-mot C., Doni G., Pavan G.M. (2021). A Data-Driven Dimensionality Reduction Approach to Compare and Classify Lipid Force Fields. J. Phys. Chem. B.

[162] Bratek M., Wójcik-Augustyn A., Kania A., Majta J., Murzyn K. (2020). Condensed phase properties of n-pentadecane emerging from application of biomolecular force fields. Acta Biochim. Pol..

[163] Paloncýova M., Fabre G., Devane R.H., Trouillas P., Berka K., Otyepka M. (2014). Benchmarking of Force Fields for Molecule—Membrane Interactions. J. Chem. Theory Comput..

[164] Alsop R.J., Dhaliwal A., Rheinstädter M.C. (2017). Curcumin Protects Membranes through a Carpet or Insertion Model Depending on Hydration. Langmuir.

[165] Salnikov E., Drung B., Fabre G., Itkin A., Otyepka M., Dencher N.A., Schmidt B., Hauß T., Trouillas P., Bechinger B. (2018). Lipid bilayer position and orientation of novel carprofens, modulators of γ-secretase in Alzheimer’s disease. Biochim. Biophys. Acta-Biomembr..

[166] Khajeh A., Modarress H. (2014). The influence of cholesterol on interactions and dynamics of ibuprofen in a lipid bilayer. Biochim. Biophys. Acta-Biomembr..

[167] Wong W.C., Juo J.Y., Lin C.H., Liao Y.H., Cheng C.Y., Hsieh C.L. (2019). Characterization of Single-Protein Dynamics in Polymer-Cushioned Lipid Bilayers Derived from Cell Plasma Membranes. J. Phys. Chem. B.

[168] Bunker A., Magarkar A., Viitala T. (2016). Rational design of liposomal drug delivery systems, a review: Combined experimental and computational studies of lipid membranes, liposomes and their PEGylation. Biochim. Biophys. Acta-Biomembr..

[169] Dzieciuch M., Rissanen S., Szydłowska N., Bunker A., Kumorek M., Jamróz D., Vattulainen I., Nowakowska M., Róg T., Kepczynski M. (2015). Pegylated liposomes as carriers of hydrophobic porphyrins. J. Phys. Chem. B.

[170] Lajunen T., Nurmi R., Wilbie D., Ruoslahti T., Johansson N.G., Korhonen O., Róg T., Bunker A., Ruponen M., Urtti A. (2018). The effect of light sensitizer localization on the stability of indocyanine green liposomes. J. Control. Release.

[171] Podloucká P., Berka K., Fabre G., Paloncýová M., Duroux J.L., Otyepka M., Trouillas P. (2013). Lipid bilayer membrane affinity rationalizes inhibition of lipid peroxidation by a natural lignan antioxidant. J. Phys. Chem. B.

[172] Fabre G., Hänchen A., Calliste C.A., Berka K., Banala S., Otyepka M., Süssmuth R.D., Trouillas P. (2015). Lipocarbazole, an efficient lipid peroxidation inhibitor anchored in the membrane. Bioorganic Med. Chem..

[173] Fabre G., Bayach I., Berka K., Paloncýová M., Starok M., Rossi C., Duroux J.L., Otyepka M., Trouillas P. (2015). Synergism of antioxidant action of vitamins E, C and quercetin is related to formation of molecular associations in biomembranes. Chem. Commun..

[174] Yang Y., Lee M., Fairn G.D. (2018). Phospholipid subcellular localization and dynamics. J. Biol. Chem..

[175] Van Meer G., Voelker D.R., Feigenson G.W. (2008). Membrane lipids: Where they are and how they behave. Nat. Rev. Mol. Cell Biol..

[176] Menon A.K., Levine T.P. (2016). The cellular lipid landscape. Biochim. Biophys. Acta-Mol. Cell Biol. Lipids.

[177] Lingwood D., Kaiser H.-J.H.-J., Levental I., Simons K. (2009). Lipid rafts as functional heterogeneity in cell membranes. Biochem. Soc. Trans..

[178] Lingwood D., Simons K. (2012). Lipid Rafts As a Membrane-Organizing Principle. Science.

[179] Alves A.C., Magarkar A., Horta M., Lima J.L.F.C., Bunker A., Nunes C., Reis S. (2017). Influence of doxorubicin on model cell membrane properties: Insights from in vitro and in silico studies. Sci. Rep..

[180] Yang Y., Dong H., Zhou H.X. (2021). Effects of Cholesterol on the Partitioning of a Drug Molecule in Lipid Bilayers. J. Phys. Chem. B.

[181] Yuan J., Meng F. (2021). Effects of cholesterol on chlorzoxazone translocation across POPC bilayer. J. Mol. Model..

[182] Li Y.-C., Rissanen S., Stepniewski M., Cramariuc O., Róg T., Mirza S., Xhaard H., Wytrwal M., Kepczynski M., Bunker A. (2012). Study of interaction between PEG carrier and three relevant drug molecules: Piroxicam, paclitaxel, and hematoporphyrin. J. Phys. Chem. B.

[183] Rissanen S., Kumorek M., Martinez-Seara H., Li Y.C., Jamróz D., Bunker A., Nowakowska M., Vattulainen I., Kepczynski M., Róg T. (2014). Effect of PEGylation on drug entry into lipid bilayer. J. Phys. Chem. B.

[184] Dzieciuch-Rojek M., Poojari C., Bednar J., Bunker A., Kozik B., Nowakowska M., Vattulainen I., Wydro P.P., Kepczynski M., Róg T. (2017). Effects of membrane PEGylation on entry and location of antifungal drug itraconazole and their pharmacological implications. Mol. Pharmacol..

[185] Wilkosz N., Rissanen S., Cyza M., Szybka R., Nowakowska M., Bunker A., Róg T., Kepczynski M. (2017). Effect of piroxicam on lipid membranes: Drug encapsulation and gastric toxicity aspects. Eur. J. Pharm. Sci..

[186] Poojari C., Wilkosz N., Lira R.B., Dimova R., Jurkiewicz P., Petka R., Kepczynski M., Róg T. (2019). Behavior of the DPH fluorescence probe in membranes perturbed by drugs. Chem. Phys. Lipids.

[187] Poojari C., Zak A., Dzieciuch-Rojek M., Bunker A., Kepczynski M., Róg T. (2020). Cholesterol Reduces Partitioning of Antifungal Drug Itraconazole into Lipid Bilayers. J. Phys. Chem. B.

[188] Bulbake U., Doppalapudi S., Kommineni N., Khan W. (2017). Liposomal formulations in clinical use: An updated review. Pharmaceutics.

[189] Juszkiewicz K., Sikorski A.F., Czogalla A. (2020). Building blocks to design liposomal delivery systems. Int. J. Mol. Sci..

[190] Ivanova D., Deneva V., Nedeltcheva D., Kamounah F.S., Gergov G., Hansen P.E., Kawauchi S., Antonov L. (2015). Tautomeric transformations of piroxicam in solution: A combined experimental and theoretical study. RSC Adv..

[191] Lamprakis C., Stocker A., Cascella M. (2015). Mechanisms of recognition and binding of α-TTP to the plasma membrane by multi-scale molecular dynamics simulations. Front. Mol. Biosci..

[192] Fotakis C., Christodouleas D., Zoumpoulakis P., Kritsi E., Benetis N.P., Mavromoustakos T., Reis H., Gili A., Papadopoulos M.G., Zervou M. (2011). Comparative biophysical studies of sartan class drug molecules losartan and candesartan (CV-11974) with membrane bilayers. J. Phys. Chem. B.

[193] Kiriakidi S., Chatzigiannis C., Papaemmanouil C., Tzakos A.G., Mavromoustakos T. (2020). Exploring the role of the membrane bilayer in the recognition of candesartan by its GPCR AT1 receptor. Biochim. Biophys. Acta-Biomembr..

[194] Först G., Cwiklik L., Jurkiewicz P., Schubert R., Hof M. (2014). Interactions of beta-blockers with model lipid membranes: Molecular view of the interaction of acebutolol, oxprenolol, and propranolol with phosphatidylcholine vesicles by time-dependent fluorescence shift and molecular dynamics simulations. Eur. J. Pharm. Biopharm..

[195] Dror R.O., Pan A.C., Arlow D.H., Borhani D.W., Maragakis P., Shan Y., Xu H., Shaw D.E. (2011). Pathway and mechanism of drug binding to G-protein-coupled receptors. Proc. Natl. Acad. Sci. USA.

[196] Wang H., Ren X., Meng F. (2016). Molecular dynamics simulation of six β-blocker drugs passing across POPC bilayer. Mol. Simul..

[197] Liu X., Kaindl J., Korczynska M., Stößel A., Dengler D., Stanek M., Hübner H., Clark M.J., Mahoney J., Matt R.A. (2020). An allosteric modulator binds to a conformational hub in the β2 adrenergic receptor. Nat. Chem. Biol..

[198] Yousefpour A., Modarress H., Goharpey F., Amjad-Iranagh S. (2015). Interaction of PEGylated anti-hypertensive drugs, amlodipine, atenolol and lisinopril with lipid bilayer membrane: A molecular dynamics simulation study. Biochim. Biophys. Acta-Biomembr..

[199] Thai N.Q., Theodorakis P.E., Li M.S. (2020). Fast Estimation of the Blood-Brain Barrier Permeability by Pulling a Ligand through a Lipid Membrane. J. Chem. Inf. Model..

[200] Wang T., Duan Y. (2009). Ligand Entry and Exit Pathways in the β2-Adrenergic Receptor. J. Mol. Biol..

[201] Yan S., Shaw D.E., Yang L., Sandham D.A., Healy M.P., Reilly J., Wang B. (2017). Interactions between β2-Adrenoceptor Ligands and Membrane: Atomic-Level Insights from Magic-Angle Spinning NMR. J. Med. Chem..

[202] Yue Z., Li C., Voth G.A., Swanson J.M.J. (2019). Dynamic Protonation Dramatically Affects the Membrane Permeability of Drug-like Molecules. J. Am. Chem. Soc..

[203] Coimbra J.T.S., Fernandes P.A., Ramos M.J. (2017). Revisiting Partition in Hydrated Bilayer Systems. J. Chem. Theory Comput..

[204] Khadka N.K., Cheng X., Ho C.S., Katsaras J., Pan J. (2015). Interactions of the Anticancer Drug Tamoxifen with Lipid Membranes. Biophys. J..

[205] Karami L., Jalili S. (2015). Effects of cholesterol concentration on the interaction of cytarabine with lipid membranes: A molecular dynamics simulation study. J. Biomol. Struct. Dyn..

[206] Liu H., Chen J., Shen Q., Fu W., Wu W. (2010). Molecular insights on the cyclic peptide nanotube-mediated transportation of antitumor drug 5-fluorouracil. Mol. Pharm..

[207] Khajeh A., Modarress H. (2014). Effect of cholesterol on behavior of 5-fluorouracil (5-FU) in a DMPC lipid bilayer, a molecular dynamics study. Biophys. Chem..

[208] Alves A.C., Ribeiro D., Horta M., Lima J.L.F.C., Nunes C., Reis S. (2017). The daunorubicin interplay with mimetic model membranes of cancer cells: A biophysical interpretation. Biochim. Biophys. Acta-Biomembr..

[209] Matyszewska D. (2020). The influence of charge and lipophilicity of daunorubicin and idarubicin on their penetration of model biological membranes—Langmuir monolayer and electrochemical studies. Biochim. Biophys. Acta-Biomembr..

[210] Wu X., Chantemargue B., Di Meo F., Bourgaux C., Chapron D., Trouillas P., Rosilio V. (2019). Deciphering the Peculiar Behavior of β-Lapachone in Lipid Monolayers and Bilayers. Langmuir.

[211] Węder K., Mach M., Hąc-Wydro K., Wydro P. (2018). Studies on the interactions of anticancer drug—Minerval—with membrane lipids in binary and ternary Langmuir monolayers. Biochim. Biophys. Acta-Biomembr..

[212] Petit K., Suwalsky M., Colina J.R., Aguilar L.F., Jemiola-Rzeminska M., Strzalka K. (2019). In vitro effects of the antitumor drug miltefosine on human erythrocytes and molecular models of its membrane. Biochim. Biophys. Acta-Biomembr..

[213] Haralampieva I., de Armiño D.J.A., Luck M., Fischer M., Abel T., Huster D., Di Lella S., Scheidt H.A., Müller P. (2020). Interaction of the small-molecule kinase inhibitors tofacitinib and lapatinib with membranes. Biochim. Biophys. Acta-Biomembr..

[214] Mahadeo M., Prenner E.J. (2020). Differential impact of synthetic antitumor lipid drugs on the membrane organization of phosphatidic acid and diacylglycerol monolayers. Chem. Phys. Lipids.

[215] Tang P.K., Chakraborty K., Hu W., Kang M., Loverde S.M. (2020). Interaction of Camptothecin with Model Cellular Membranes. J. Chem. Theory Comput..

[216] Tang P.K., Manandhar A., Hu W., Kang M., Loverde S.M. (2021). The interaction of supramolecular anticancer drug amphiphiles with phospholipid membranes. Nanoscale Adv..

[217] Zhang L., Bennett W.F.D., Zheng T., Ouyang P.-K., Ouyang X., Qiu X., Luo A., Karttunen M., Chen P. (2016). Effect of Cholesterol on Cellular Uptake of Cancer Drugs Pirarubicin and Ellipticine. J. Phys. Chem. B.

[218] Da Fonseca C., Khandelia H., Salazar M., Schönthal A., Meireles O., Quirico-Santos T. (2016). Perillyl alcohol: Dynamic interactions with the lipid bilayer and implications for long-term inhalational chemotherapy for gliomas. Surg. Neurol. Int..

[219] Rivel T., Ramseyer C., Yesylevskyy S. (2019). The asymmetry of plasma membranes and their cholesterol content influence the uptake of cisplatin. Sci. Rep..

[220] Yesylevskyy S., Cardey B., Kraszewski S., Foley S., Enescu M., da Silva A.M., Santos H.F.D., Ramseyer C. (2015). Empirical force field for cisplatin based on quantum dynamics data: Case study of new parameterization scheme for coordination compounds. J. Mol. Model..

[221] Chakraborty K., Dutta C., Mukherjee S., Biswas A., Gayen P., George G., Raghothama S., Ghosh S., Dey S., Bhattacharyya D. (2018). Engineering Ionophore Gramicidin-Inspired Self-Assembled Peptides for Drug Delivery and Cancer Nanotherapeutics. Adv. Ther..

[222] Alves A.C., Ribeiro D., Nunes C., Reis S. (2016). Biophysics in cancer: The relevance of drug-membrane interaction studies. Biochim. Biophys. Acta-Biomembr..

[223] Bourgaux C., Couvreur P. (2014). Interactions of anticancer drugs with biomembranes: What can we learn from model membranes?. J. Control. Release.

[224] Almeida A., Fernandes E., Sarmento B., Lúcio M. (2021). A biophysical insight of camptothecin biodistribution: Towards a molecular understanding of its pharmacokinetic issues. Pharmaceutics.

[225] Herrera F.E., Sevrain C.M., Jaffrès P.-A., Couthon H., Grélard A., Dufourc E.J., Chantôme A., Potier-Cartereau M., Vandier C., Bouchet A.M. (2017). Singular Interaction between an Antimetastatic Agent and the Lipid Bilayer: The Ohmline Case. ACS Omega.

[226] Pederzoli M., Wasif Baig M., Kývala M., Pittner J., Cwiklik L. (2019). Photophysics of BODIPY-Based Photosensitizer for Photodynamic Therapy: Surface Hopping and Classical Molecular Dynamics. J. Chem. Theory Comput..

[227] Leite N.B., Martins D.B., Fazani V.E., Vieira M.R., dos Santos Cabrera M.P. (2018). Cholesterol modulates curcumin partitioning and membrane effects. Biochim. Biophys. Acta-Biomembr..

[228] Lyu Y., Xiang N., Mondal J., Zhu X., Narsimhan G. (2018). Characterization of Interactions between Curcumin and Different Types of Lipid Bilayers by Molecular Dynamics Simulation. J. Phys. Chem. B.

[229] Khondker A., Alsop R.J., Himbert S., Tang J., Shi A.C., Hitchcock A.P., Rheinstädter M.C. (2018). Membrane-Modulating Drugs can Affect the Size of Amyloid-β 25–35 Aggregates in Anionic Membranes. Sci. Rep..

[230] Ashida Y., Yanagita R.C., Takahashi C., Kawanami Y., Irie K. (2016). Binding mode prediction of aplysiatoxin, a potent agonist of protein kinase C, through molecular simulation and structure–activity study on simplified analogs of the receptor-recognition domain. Bioorganic Med. Chem..

[231] Ryckbosch S.M., Wender P.A., Pande V.S. (2017). Molecular dynamics simulations reveal ligand-controlled positioning of a peripheral protein complex in membranes. Nat. Commun..

[232] Khondker A., Malenfant D.J., Dhaliwal A.K., Rheinstädter M.C. (2018). Carbapenems and Lipid Bilayers: Localization, Partitioning, and Energetics. ACS Infect. Dis..

[233] Cramariuc O., Róg T., Javanainen M., Monticelli L., Polishchuk A.V., Vattulainen I. (2012). Mechanism for translocation of fluoroquinolones across lipid membranes. Biochim. Biophys. Acta-Biomembr..

[234] Cetuk H., Anishkin A., Scott A.J., Rempe S.B., Ernst R.K., Sukharev S. (2021). Partitioning of Seven Different Classes of Antibiotics into LPS Monolayers Supports Three Different Permeation Mechanisms through the Outer Bacterial Membrane. Langmuir.

[235] Sousa C.F., Coimbra J.T.S., Ferreira M., Pereira-Leite C., Reis S., Ramos M.J., Fernandes P.A., Gameiro P. (2021). Passive Diffusion of Ciprofloxacin and its Metalloantibiotic: A Computational and Experimental study. J. Mol. Biol..

[236] Ortiz-Collazos S., Picciani P.H.S., Oliveira O.N., Pimentel A.S., Edler K.J. (2019). Influence of levofloxacin and clarithromycin on the structure of DPPC monolayers. Biochim. Biophys. Acta-Biomembr..

[237] Vignoli Muniz G.S., Souza M.C., Duarte E.L., Lamy M.T. (2021). Comparing the interaction of the antibiotic levofloxacin with zwitterionic and anionic membranes: Calorimetry, fluorescence, and spin label studies. Biochim. Biophys. Acta-Biomembr..

[238] Ortiz-Collazos S., Estrada-López E.D., Pedreira A.A., Picciani P.H.S., Oliveira O.N., Pimentel A.S. (2017). Interaction of levofloxacin with lung surfactant at the air-water interface. Colloids Surf. B Biointerfaces.

[239] Vila-Viçosa D., Victor B.L., Ramos J., Machado D., Viveiros M., Switala J., Loewen P.C., Leitao R., Martins F., Machuqueiro M. (2017). Insights on the Mechanism of Action of INH-C10 as an Antitubercular Prodrug. Mol. Pharm..

[240] Samelo J., Mora M.J., Granero G.E., Moreno M.J. (2017). Partition of amphiphilic molecules to lipid bilayers by ITC: Low-affinity solutes. ACS Omega.

[241] Li J., Beuerman R.W., Verma C.S. (2018). Molecular Insights into the Membrane Affinities of Model Hydrophobes. ACS Omega.

[242] Zhang H., Shao X., Dehez F., Cai W., Chipot C. (2020). Modulation of membrane permeability by carbon dioxide. J. Comput. Chem..

[243] Hörömpöli D., Ciglia C., Glüsenkamp K.-H., Haustedt O.L., Falkenstein H.-P., Bendas G., Berscheid A., Brötz-Oesterhelt H. (2021). The Antibiotic Negamycin Crosses the Bacterial Cytoplasmic Membrane by Multiple Routes. Antimicrob. Agents Chemother..

[244] Kim W., Steele A.D., Zhu W., Csatary E.E., Fricke N., Dekarske M.M., Jayamani E., Pan W., Kwon B., Sinitsa I.F. (2018). Discovery and Optimization of nTZDpa as an Antibiotic Effective Against Bacterial Persisters. ACS Infect. Dis..

[245] Kumar S., Thakur J., Yadav K., Mitra M., Pal S., Ray A., Gupta S., Medatwal N., Gupta R., Mishra D. (2019). Cholic Acid-Derived Amphiphile which Combats Gram-Positive Bacteria-Mediated Infections via Disintegration of Lipid Clusters. ACS Biomater. Sci. Eng..

[246] Jaroque G.N., Sartorelli P., Caseli L. (2020). The effect of the monocyclic monoterpene tertiary alcohol γ-terpineol on biointerfaces containing cholesterol. Chem. Phys. Lipids.

[247] Kim W., Zou G., Hari T.P.A., Wilt I.K., Zhu W., Galle N., Faizi H.A., Hendricks G.L., Tori K., Pan W. (2019). A selective membrane-targeting repurposed antibiotic with activity against persistent methicillin-resistant Staphylococcus aureus. Proc. Natl. Acad. Sci. USA.

[248] Van Oosten B., Marquardt D., Harroun T.A. (2017). Testing High Concentrations of Membrane Active Antibiotic Chlorhexidine Via Computational Titration and Calorimetry. J. Phys. Chem. B.

[249] Van Oosten B., Marquardt D., Komljenović I., Bradshaw J.P., Sternin E., Harroun T.A. (2014). Small molecule interaction with lipid bilayers: A molecular dynamics study of chlorhexidine. J. Mol. Graph. Model..

[250] Rzycki M., Drabik D., Szostak-Paluch K., Hanus-Lorenz B., Kraszewski S. (2021). Unraveling the mechanism of octenidine and chlorhexidine on membranes: Does electrostatics matter?. Biophys. J..

[251] Poger D., Mark A.E. (2019). Effect of Triclosan and Chloroxylenol on Bacterial Membranes. J. Phys. Chem. B.

[252] Kim A.V., Shelepova E.A., Selyutina O.Y., Meteleva E.S., Dushkin A.V., Medvedev N.N., Polyakov N.E., Lyakhov N.Z. (2019). Glycyrrhizin-assisted transport of praziquantel anthelmintic drug through the lipid membrane: An experiment and MD simulation. Mol. Pharm..

[253] Leonis G., Czyżnikowska Ż., Megariotis G., Reis H., Papadopoulos M.G. (2012). Computational studies of darunavir into HIV-1 protease and DMPC bilayer: Necessary conditions for effective binding and the role of the flaps. J. Chem. Inf. Model..

[254] Konstantinidi A., Chountoulesi M., Naziris N., Sartori B., Amenitsch H., Plakantonaki M., Triantafyllakou I., Tselios T., Demetzos C., Busath D.D. (2020). The boundary lipid around DMPC-spanning in fl uenza A M2 transmembrane domain channels: Its structure and potential for drug accommodation. Biochim. Biophys. Acta-Biomembr..

[255] Konstantinidi A., Naziris N., Chountoulesi M., Kiriakidi S., Sartori B., Kolokouris D., Amentisch H., Mali G., Ntountaniotis D., Demetzos C. (2018). Comparative Perturbation Effects Exerted by the Influenza A M2 WT Protein Inhibitors Amantadine and the Spiro[pyrrolidine-2,2′-adamantane] Variant AK13 to Membrane Bilayers Studied Using Biophysical Experiments and Molecular Dynamics Simulations. J. Phys. Chem. B.

[256] Khurana E., Devane R.H., Dal Peraro M., Klein M.L. (2011). Computational study of drug binding to the membrane-bound tetrameric M2 peptide bundle from influenza A virus. Biochim. Biophys. Acta-Biomembr..

[257] Kordzadeh A., Ramazani Saadatabadi A., Hadi A. (2020). Investigation on penetration of saffron components through lipid bilayer bound to spike protein of SARS-CoV-2 using steered molecular dynamics simulation. Heliyon.

[258] Kasparyan G., Poojari C., Róg T., Hub J.S. (2020). Cooperative Effects of an Antifungal Moiety and DMSO on Pore Formation over Lipid Membranes Revealed by Free Energy Calculations. J. Phys. Chem. B.

[259] Szomek M., Reinholdt P., Petersen D., Caci A., Kongsted J., Wüstner D. (2021). Direct observation of nystatin binding to the plasma membrane of living cells. Biochim. Biophys. Acta-Biomembr..

[260] Grela E., Wieczór M., Luchowski R., Zielinska J., Barzycka A., Grudzinski W., Nowak K., Tarkowski P., Czub J., Gruszecki W.I. (2018). Mechanism of Binding of Antifungal Antibiotic Amphotericin B to Lipid Membranes: An Insight from Combined Single-Membrane Imaging, Microspectroscopy, and Molecular Dynamics. Mol. Pharm..

[261] Markiewicz M., Pasenkiewicz-Gierula M. (2011). Comparative model studies of gastric toxicity of nonsteroidal anti-inflammatory drugs. Langmuir.

[262] Blasi P., Casagrande S., Pedretti A., Fioretto D., Vistoli G., Corezzi S. (2020). Ketoprofen poly(lactide-co-glycolide) physical interaction studied by Brillouin spectroscopy and molecular dynamics simulations. Int. J. Pharm..

[263] Hu J., Liu H.H., Xu P., Shang Y., Liu H.H. (2019). Investigation of Drug for Pulmonary Administration-Model Pulmonary Surfactant Monolayer Interactions Using Langmuir-Blodgett Monolayer and Molecular Dynamics Simulation: A Case Study of Ketoprofen. Langmuir.

[264] Da Silva E., Autilio C., Hougaard K.S., Baun A., Cruz A., Perez-Gil J., Sørli J.B. (2021). Molecular and biophysical basis for the disruption of lung surfactant function by chemicals. Biochim. Biophys. Acta-Biomembr..

[265] Sodeifian G., Razmimanesh F. (2019). Diffusional interaction behavior of NSAIDs in lipid bilayer membrane using molecular dynamics (MD) simulation: Aspirin and Ibuprofen. J. Biomol. Struct. Dyn..

[266] Alsop R.J., Himbert S., Dhaliwal A., Schmalzl K., Rheinstädter M.C. (2018). Aspirin locally disrupts the liquid-ordered phase. R. Soc. Open Sci..

[267] Barrett M.A., Zheng S., Roshankar G., Alsop R.J., Belanger R.K.R., Rheinsta M.C., Huynh C., Kuc N. (2012). Interaction of Aspirin (Acetylsalicylic Acid) with Lipid Membranes. PLoS ONE.

[268] Sharma V.K., Mamontov E., Ohl M., Tyagi M. (2017). Incorporation of aspirin modulates the dynamical and phase behavior of the phospholipid membrane. Phys. Chem. Chem. Phys..

[269] Alsop R.J., Barrett M.A., Zheng S. (2014). Acetylsalicylic acid (ASA) increases the solubility of cholesterol when incorporated in lipid membranes. Soft Matter.

[270] Jämbeck J.P.M., Lyubartsev A.P. (2013). Exploring the free energy landscape of solutes embedded in lipid bilayers. J. Phys. Chem. Lett..

[271] Alsop R.J., Toppozini L., Marquardt D., Ku N., Harroun T.A., Rheinstädter M.C. (2015). Aspirin inhibits formation of cholesterol rafts in fluid lipid membranes. Biochim. Biophys. Acta-Biomembr..

[272] Berka K., Hendrychová T., Anzenbacher P., Otyepka M. (2011). Membrane position of ibuprofen agrees with suggested access path entrance to cytochrome P450 2C9 active site. J. Phys. Chem. A.

[273] Kremkow J., Luck M., Huster D., Müller P., Scheidt H.A. (2020). Membrane Interaction of Ibuprofen with Cholesterol-Containing Lipid Membranes. Biomolecules.

[274] Nitschke N., Atkovska K., Hub J.S. (2016). Accelerating potential of mean force calculations for lipid membrane permeation: System size, reaction coordinate, solute-solute distance, and cutoffs. J. Chem. Phys..

[275] Boggara M.B., Mihailescu M., Krishnamoorti R. (2012). Structural association of nonsteroidal anti-inflammatory drugs with lipid membranes. J. Am. Chem. Soc..

[276] Rojas-Valencia N., Gómez S., Montillo S., Manrique-Moreno M., Cappelli C., Hadad C., Restrepo A. (2020). Evolution of Bonding during the Insertion of Anionic Ibuprofen into Model Cell Membranes. J. Phys. Chem. B.

[277] Sharma V.K., Mamontov E., Tyagi M. (2020). Effects of NSAIDs on the nanoscopic dynamics of lipid membrane. Biochim. Biophys. Acta-Biomembr..

[278] Markiewicz M., Librowski T., Lipkowska A., Serda P., Baczynski K., Pasenkiewicz-Gierula M. (2017). Assessing gastric toxicity of xanthone derivatives of anti-inflammatory activity using simulation and experimental approaches. Biophys. Chem..

[279] Fearon A.D., Stokes G.Y. (2017). Thermodynamics of Indomethacin Adsorption to Phospholipid Membranes. J. Phys. Chem. B.

[280] Kabedev A., Hossain S., Hubert M., Larsson P., Bergström C.A.S. (2021). Molecular Dynamics Simulations Reveal Membrane Interactions for Poorly Water-Soluble Drugs: Impact of Bile Solubilization and Drug Aggregation. J. Pharm. Sci..

[281] Tse C.H., Comer J., Wang Y., Chipot C. (2018). Link between Membrane Composition and Permeability to Drugs. J. Chem. Theory Comput..

[282] Yang C., Guo S., Wu X., Yang P., Han L., Dai X., Shi X. (2020). Multiscale study on the enhancing effect and mechanism of borneolum on transdermal permeation of drugs with different log P values and molecular sizes. Int. J. Pharm..

[283] Nademi Y., Amjad Iranagh S., Yousefpour A., Mousavi S.Z., Modarress H. (2014). Molecular dynamics simulations and free energy profile of Paracetamol in DPPC and DMPC lipid bilayers. J. Chem. Sci..

[284] Palaiokostas M., Ding W., Shahane G., Orsi M. (2018). Effects of lipid composition on membrane permeation. Soft Matter.

[285] Fischer A., Smieško M. (2019). Spontaneous Ligand Access Events to Membrane-Bound Cytochrome P450 2D6 Sampled at Atomic Resolution. Sci. Rep..

[286] Faulkner C., de Leeuw N.H. (2021). Predicting the Membrane Permeability of Fentanyl and Its Analogues by Molecular Dynamics Simulations. J. Phys. Chem. B.

[287] Lee C.T., Comer J., Herndon C., Leung N., Pavlova A., Swift R.V., Tung C., Rowley C.N., Amaro R.E., Chipot C. (2016). Simulation-Based Approaches for Determining Membrane Permeability of Small Compounds. J. Chem. Inf. Model..

[288] López Cascales J.J.J., Oliveira Costa S.D.D. (2013). Effect of the interfacial tension and ionic strength on the thermodynamic barrier associated to the benzocaine insertion into a cell membrane. Biophys. Chem..

[289] Saeedi M., Lyubartsev A.P., Jalili S. (2017). Anesthetics mechanism on a DMPC lipid membrane model: Insights from molecular dynamics simulations. Biophys. Chem..

[290] Paloncyová M., Devane R.H., Murch B.P., Berka K., Otyepka M. (2014). Rationalization of reduced penetration of drugs through ceramide gel phase membrane. Langmuir.

[291] Lopes S.C., Ivanova G., de Castro B., Gameiro P. (2019). Cardiolipin and phosphatidylethanolamine role in dibucaine interaction with the mitochondrial membrane. Biochim. Biophys. Acta-Biomembr..

[292] Jambeck J.P.M., Lyubartsev A.P. (2013). Implicit inclusion of atomic polarization in modeling of partitioning between water and lipid bilayers. Phys. Chem. Chem. Phys..

[293] Santa-Maria A.R., Walter F.R., Valkai S., Brás A.R., Mészáros M., Kincses A., Klepe A., Gaspar D., Castanho M.A.R.B., Zimányi L. (2019). Lidocaine turns the surface charge of biological membranes more positive and changes the permeability of blood-brain barrier culture models. Biochim. Biophys. Acta-Biomembr..

[294] Hu S., Zhao T., Li H., Cheng D., Sun Z. (2020). Effect of tetracaine on dynamic reorganization of lipid membranes. Biochim. Biophys. Acta-Biomembr..

[295] Velez-Saboyá C.S., Oropeza-Guzman E., Sierra-Valdez F.J., Ruiz-Suárez J.C. (2021). Ca2+-mediated enhancement of anesthetic diffusion across phospholipid multilamellar systems. Biochim. Biophys. Acta-Biomembr..

[296] Pickholz M., Giupponi G. (2010). Coarse grained simulations of local anesthetics encapsulated into a liposome. J. Phys. Chem. B.

[297] Jorgensen C., Domene C. (2018). Location and Character of Volatile General Anesthetics Binding Sites in the Transmembrane Domain of TRPV1. Mol. Pharm..

[298] Fábián B., Darvas M., Picaud S., Sega M., Jedlovszky P. (2015). The effect of anaesthetics on the properties of a lipid membrane in the biologically relevant phase: A computer simulation study. Phys. Chem. Chem. Phys..

[299] Pavel M.A., Petersen E.N., Wang H., Lerner R.A., Hansen S.B. (2020). Studies on the mechanism of general anesthesia. Proc. Natl. Acad. Sci. USA.

[300] Reigada R. (2013). Atomistic Study of Lipid Membranes Containing Chloroform: Looking for a Lipid-Mediated Mechanism of Anesthesia. PLoS ONE.

[301] Reigada R., Sagues F. (2015). Chloroform alters interleaflet coupling in lipid bilayers: An entropic mechanism. J. R. Soc. Interface.

[302] Chau P.-L., Tu K.M., Liang K.K., Todorov I.T., Roser S.J., Barker R., Matubayasi N. (2012). The effect of pressure on halothane binding to hydrated DMPC bilayers. Mol. Phys..

[303] Tu K.M., Matubayasi N., Liang K.K., Todorov I.T., Chan S.L., Chau P.-L. (2012). A possible molecular mechanism for the pressure reversal of general anaesthetics: Aggregation of halothane in POPC bilayers at high pressure. Chem. Phys. Lett..

[304] Patel J., Chowdhury E.A., Noorani B., Bickel U., Huang J. (2020). Isoflurane increases cell membrane fluidity significantly at clinical concentrations. Biochim. Biophys. Acta-Biomembr..

[305] Arcario M.J., Mayne C.G., Tajkhorshid E. (2014). Atomistic Models of General Anesthetics for Use in in Silico Biological Studies. J. Phys. Chem. B.

[306] Rao B.D., Shrivastava S., Pal S., Chattopadhyay A. (2019). Effect of Local Anesthetics on the Organization and Dynamics of Hippocampal Membranes: A Fluorescence Approach. J. Phys. Chem. B.

[307] Arcario M.J., Mayne C.G., Tajkhorshid E. (2017). A membrane-embedded pathway delivers general anesthetics to two interacting binding sites in the *Gloeobacter violaceus* ion channel. J. Biol. Chem..

[308] Hantal G., Fábián B., Sega M., Jójárt B., Jedlovszky P. (2019). Effect of general anesthetics on the properties of lipid membranes of various compositions. Biochim. Biophys. Acta-Biomembr..

[309] Jin W., Zucker M., Pralle A. (2021). Membrane nanodomains homeostasis during propofol anesthesia as function of dosage and temperature. Biochim. Biophys. Acta-Biomembr..

[310] Hansen A.H., Sørensen K.T., Mathieu R., Serer A., Duelund L., Khandelia H., Hansen P.L., Simonsen A.C. (2013). Propofol modulates the lipid phase transition and localizes near the headgroup of membranes. Chem. Phys. Lipids.

[311] Jerabek H., Pabst G., Rappolt M., Stockner T. (2010). Membrane-Mediated Effect on Ion Channels Induced by the Anesthetic Drug Ketamine. J. Am. Chem. Soc..

[312] Denisov I.G., Baylon J.L., Grinkova Y.V., Tajkhorshid E., Sligar S.G. (2018). Drug-Drug Interactions between Atorvastatin and Dronedarone Mediated by Monomeric CYP3A4. Biochemistry.

[313] Galiullina L.F., Scheidt H.A., Huster D., Aganov A., Klochkov V. (2019). Interaction of statins with phospholipid bilayers studied by solid-state NMR spectroscopy. Biochim. Biophys. Acta-Biomembr..

[314] Sodero A.O., Barrantes F.J. (2020). Pleiotropic effects of statins on brain cells. Biochim. Biophys. Acta-Biomembr..

[315] Kuba J.O., Yu Y., Klauda J.B. (2021). Estimating localization of various statins within a POPC bilayer. Chem. Phys. Lipids.

[316] Teo R.D., Tieleman D.P. (2021). Modulation of Phospholipid Bilayer Properties by Simvastatin. J. Phys. Chem. B.

[317] Murphy C., Deplazes E., Cranfield C.G., Garcia A. (2020). The role of structure and biophysical properties in the pleiotropic effects of statins. Int. J. Mol. Sci..

[318] Ma J., Domicevica L., Schnell J.R., Biggin P.C. (2015). Position and orientational preferences of drug-like compounds in lipid membranes: A computational and NMR approach. Phys. Chem. Chem. Phys..

[319] Xie B., Hao C., Sun R. (2020). Effect of fluoxetine at different concentrations on the adsorption behavior of Langmuir monolayers. Biochim. Biophys. Acta-Biomembr..

[320] Casarotto P.C., Girych M., Fred S.M., Kovaleva V., Moliner R., Enkavi G., Biojone C., Cannarozzo C., Sahu M.P., Kaurinkoski K. (2021). Antidepressant drugs act by directly binding to TRKB neurotrophin receptors. Cell.

[321] Kopec W., Khandelia H. (2014). Reinforcing the membrane-mediated mechanism of action of the anti-tuberculosis candidate drug thioridazine with molecular simulations. J. Comput. Aided. Mol. Des..

[322] Thomas T., Fang Y., Yuriev E., Chalmers D.K. (2016). Ligand Binding Pathways of Clozapine and Haloperidol in the Dopamine D2 and D3 Receptors. J. Chem. Inf. Model..

[323] Pérez-Isidoro R., Costas M. (2020). The effect of neuroleptic drugs on DPPC/sphingomyelin/cholesterol membranes. Chem. Phys. Lipids.

[324] Riedlová K., Nekardová M., Kačer P., Syslová K., Vazdar M., Jungwirth P., Kudová E., Cwiklik L. (2017). Distributions of therapeutically promising neurosteroids in cellular membranes. Chem. Phys. Lipids.

[325] Heydari Dokoohaki M., Zolghadr A.R., Klein A. (2020). Impact of the chemical structure on the distribution of neuroprotective: N -alkyl-9 H -carbazoles at octanol/water interfaces. New J. Chem..

[326] Denisov I.G., Grinkova Y.V., Baylon J.L., Tajkhorshid E., Sligar S.G. (2015). Mechanism of Drug-Drug Interactions Mediated by Human Cytochrome P450 CYP3A4 Monomer. Biochemistry.

[327] Denisov I.G., Grinkova Y.V., Nandigrami P., Shekhar M., Tajkhorshid E., Sligar S.G. (2019). Allosteric Interactions in Human Cytochrome P450 CYP3A4: The Role of Phenylalanine 213. Biochemistry.

[328] Müller C.S., Knehans T., Davydov D.R., Bounds P.L., Von Mandach U., Halpert J.R., Caflisch A., Koppenol W.H. (2015). Concurrent cooperativity and substrate inhibition in the epoxidation of carbamazepine by cytochrome P450 3A4 active site mutants inspired by molecular dynamics simulations. Biochemistry.

[329] Yuan X., Raniolo S., Limongelli V., Xu Y. (2018). The Molecular Mechanism Underlying Ligand Binding to the Membrane-Embedded Site of a G-Protein-Coupled Receptor. J. Chem. Theory Comput..

[330] Witek J., Mühlbauer M., Keller B.G., Blatter M., Meissner A., Wagner T., Riniker S. (2017). Interconversion Rates between Conformational States as Rationale for the Membrane Permeability of Cyclosporines. Chem. Phys. Chem..

[331] Aguayo-Ortiz R., Creech J., Jiménez-Vázquez E.N., Guerrero-Serna G., Wang N., da Rocha A.M., Herron T.J., Espinoza-Fonseca L.M. (2021). From atoms to cells: Bridging the gap between potency, efficacy, and safety of small molecules directed at a membrane protein. bioRxiv.

[332] Eftimov P., Olżyńska A., Melcrová A., Georgiev G.A., Daull P., Garrigue J.S., Cwiklik L. (2020). Improving stability of tear film lipid layer via concerted action of two drug molecules: A biophysical view. Int. J. Mol. Sci..

[333] Federizon J., Feugmo C.G.T., Huang W.C., He X., Miura K., Razi A., Ortega J., Karttunen M., Lovell J.F. (2021). Experimental and computational observations of immunogenic cobalt porphyrin lipid bilayers: Nanodomain-enhanced antigen association. Pharmaceutics.

[334] Chen X.Z., Zhang R.Y., Wang X.F., Yin X.G., Wang J., Wang Y.C., Liu X., Du J.J., Liu Z., Guo J. (2019). Peptide-free Synthetic Nicotine Vaccine Candidates with α-Galactosylceramide as Adjuvant. Mol. Pharm..

[335] Parry M.J., Alakoskela J.M.I., Khandelia H., Kumar S.A., Jäättelä M., Mahalka A.K., Kinnunen P.K.J. (2008). High-affinity small molecule-phospholipid complex formation: Binding of siramesine to phosphatidic acid. J. Am. Chem. Soc..

[336] Lautala S., Provenzani R., Koivuniemi A., Kulig W., Talman V., Róg T., Tuominen R.K., Yli-Kauhaluoma J., Bunker A. (2020). Rigorous computational study reveals what docking overlooks: Double trouble from Membrane Association in Protein Kinase C Modulators. J. Chem. Inf. Model..

[337] Yu R.K., Usuki S., Itokazu Y., Wu H.C. (2015). Novel GM1 ganglioside-like peptide mimics prevent the association of cholera toxin to human intestinal epithelial cells in vitro. Glycobiology.

[338] Durdagi S., Papadopoulos M.G., Mavromoustakos T. (2012). An effort to discover the preferred conformation of the potent AMG3 cannabinoid analog when reaching the active sites of the cannabinoid receptors. Eur. J. Med. Chem..

[339] Guest E.E., Oatley S.A., Macdonald S.J.F., Hirst J.D. (2020). Molecular simulation of αvβ6 integrin inhibitors. J. Chem. Inf. Model..

[340] Luck M., Fischer M., Werle M., Scheidt H.A., Müller P. (2021). Impact of selected small-molecule kinase inhibitors on lipid membranes. Pharmaceuticals.

[341] Toroz D., Khanna T., Gould I.R. (2019). Modeling the Effect of BSEP Inhibitors in Lipid Bilayers by Means of All-Atom Molecular Dynamics Simulation. ACS Omega.

[342] Huang C., Wang H., Tang L., Meng F. (2019). Penetration enhancement of menthol on quercetin through skin: Insights from atomistic simulation. J. Mol. Model..

[343] Sinha R., Gadhwal M.K., Joshi U.J., Srivastava S., Govil G. (2011). Interaction of quercetin with DPPC model membrane: Molecular dynamic simulation, DSC and multinuclear NMR studies. J. Indian Chem. Soc..

[344] Pawlikowska-Pawlȩga B., Dziubińska H., Król E., Trȩbacz K., Jarosz-Wilkołazka A., Paduch R., Gawron A., Gruszecki W.I. (2014). Characteristics of quercetin interactions with liposomal and vacuolar membranes. Biochim. Biophys. Acta-Biomembr..

[345] Pawlikowska-Pawlega B., Kapral J., Gawron A., Stochmal A., Zuchowski J., Pecio L., Luchowski R., Grudzinski W., Gruszecki W.I. (2018). Interaction of a quercetin derivative-lensoside Aβ with liposomal membranes. Biochim. Biophys. Acta-Biomembr..

[346] Eid J., Jraij A., Greige-Gerges H., Monticelli L. (2021). Effect of quercetin on lipid membrane rigidity: Assessment by atomic force microscopy and molecular dynamics simulations. BBA Adv..

[347] De Granada-Flor A., Sousa C., Filipe H.A.L., Santos M.S.C.S., de Almeida R.F.M. (2019). Quercetin dual interaction at the membrane level. Chem. Commun..

[348] Strugała P., Tronina T., Huszcza E., Gabrielska J. (2017). Bioactivity In Vitro of Quercetin Glycoside Obtained in Beauveria bassiana Culture and Its Interaction with Liposome Membranes. Molecules.

[349] Boonnoy P., Karttunen M., Wong-Ekkabut J. (2018). Does α-Tocopherol Flip-Flop Help to Protect Membranes Against Oxidation?. J. Phys. Chem. B.

[350] Boonnoy P., Karttunen M., Wong-Ekkabut J. (2017). Alpha-tocopherol inhibits pore formation in oxidized bilayers. Phys. Chem. Chem. Phys..

[351] DiPasquale M., Nguyen M.H.L., Rickeard B.W., Cesca N., Tannous C., Castillo S.R., Katsaras J., Kelley E.G., Heberle F.A., Marquardt D. (2020). The antioxidant vitamin E as a membrane raft modulator: Tocopherols do not abolish lipid domains. Biochim. Biophys. Acta-Biomembr..

[352] Neunert G., Tomaszewska-Gras J., Baj A., Gauza-Włodarczyk M., Witkowski S., Polewski K. (2021). Phase Transitions and Structural Changes in DPPC Liposomes Induced by a 1-Carba-Alpha-Tocopherol Analogue. Molecules.

[353] Ossman T., Fabre G., Trouillas P. (2016). Interaction of wine anthocyanin derivatives with lipid bilayer membranes. Comput. Theor. Chem..

[354] Socrier L., Rosselin M., Gomez Giraldo A.M., Chantemargue B., Di Meo F., Trouillas P., Durand G., Morandat S. (2019). Nitrone-Trolox conjugate as an inhibitor of lipid oxidation: Towards synergistic antioxidant effects. Biochim. Biophys. Acta-Biomembr..

[355] Teixeira M.H., Arantes G.M. (2019). Effects of lipid composition on membrane distribution and permeability of natural quinones. RSC Adv..

[356] Feng S., Wang R., Pastor R.W., Klauda J.B., Im W. (2021). Location and Conformational Ensemble of Menaquinone and Menaquinol, and Protein–Lipid Modulations in Archaeal Membranes. J. Phys. Chem. B.

[357] Van Cleave C., Murakami H.A., Samart N., Koehn J.T., Maldonado P., Kreckel H.D., Cope E.J., Basile A., Crick D.C., Crans D.C. (2020). Location of menaquinone and menaquinol headgroups in model membranes. Can. J. Chem..

[358] Makuch K., Markiewicz M., Pasenkiewicz-Gierula M. (2019). Asymmetric Spontaneous Intercalation of Lutein into a Phospholipid Bilayer, a Computational Study. Comput. Struct. Biotechnol. J..

[359] Garcia A., Eljack N.D., Sani M.A., Separovic F., Rasmussen H.H., Kopec W., Khandelia H., Cornelius F., Clarke R.J. (2015). Membrane accessibility of glutathione. Biochim. Biophys. Acta-Biomembr..

[360] Selvaraj S., Krishnaswamy S., Devashya V., Sethuraman S., Krishnan U.M. (2015). Influence of membrane lipid composition on flavonoid–membrane interactions: Implications on their biological activity. Prog. Lipid Res..

[361] Saha S., Panieri E., Suzen S., Saso L. (2020). The Interaction of Flavonols with Membrane Components: Potential Effect on Antioxidant Activity. J. Membr. Biol..

[362] Fernandes I., Pérez-Gregorio R., Soares S., Mateus N., De Freitas V. (2017). Wine Flavonoids in Health and Disease Prevention. Molecules.

[363] Sadžak A., Brkljača Z., Crnolatac I., Baranović G., Šegota S. (2020). Flavonol clustering in model lipid membranes: DSC, AFM, force spectroscopy and MD simulations study. Colloids Surf. B Biointerfaces.

[364] Nie R., Dang M., Ge Z., Huo Y., Yu B., Tang S. (2021). Influence of the gallate moiety on the interactions between green tea polyphenols and lipid membranes elucidated by molecular dynamics simulations. Biophys. Chem..

[365] Prates É.T., Rodrigues da Silva G.H., Souza T.F., Skaf M.S., Pickholz M., de Paula E. (2020). Articaine interaction with phospholipid bilayers. J. Mol. Struct..

[366] Verstraeten S.V., Fraga C.G., Oteiza P.I. (2015). Interactions of flavan-3-ols and procyanidins with membranes: Mechanisms and the physiological relevance. Food Funct..

[367] Chulkov E.G., Schagina L.V., Ostroumova O.S. (2015). Membrane dipole modifiers modulate single-length nystatin channels via reducing elastic stress in the vicinity of the lipid mouth of a pore. Biochim. Biophys. Acta-Biomembr..

[368] Souza F.R., Fornasier F., Souza L.M.P., Peñafiel M.P., Nascimento J.B., Malfatti-Gasperini A.A., Pimentel A.S. (2020). Interaction of naringin and naringenin with DPPC monolayer at the air-water interface. Colloids Surf. A Physicochem. Eng. Asp..

[369] Sadžak A., Mravljak J., Maltar-Strmečki N., Arsov Z., Baranović G., Erceg I., Kriechbaum M., Strasser V., Přibyl J., Šegota S. (2020). The Structural Integrity of the Model Lipid Membrane during Induced Lipid Peroxidation: The Role of Flavonols in the Inhibition of Lipid Peroxidation. Antioxidants.

[370] Kosina P., Paloncýová M., Rajnochová Svobodová A., Zálešák B., Biedermann D., Ulrichová J., Vostálová J. (2019). Dermal Delivery of Selected Polyphenols from Silybum marianum. Theoretical and Experimental Study. Molecules.

[371] Laudadio E., Galeazzi R., Mobbili G., Minnelli C., Barbon A., Bortolus M., Stipa P. (2019). Depth Distribution of Spin-Labeled Liponitroxides within Lipid Bilayers: A Combined EPR and Molecular Dynamics Approach. ACS Omega.

[372] Griffith E.C., Perkins R.J., Telesford D.M., Adams E.M., Cwiklik L., Allen H.C., Roeselová M., Vaida V. (2015). Interaction of L-Phenylalanine with a Phospholipid Monolayer at the Water-Air Interface. J. Phys. Chem. B.

[373] Perkins R., Vaida V. (2017). Phenylalanine Increases Membrane Permeability. J. Am. Chem. Soc..

[374] 3Perkins R.J., Kukharchuk A., Delcroix P., Shoemaker R.K., Roeselová M., Cwiklik L., Vaida V. (2016). The Partitioning of Small Aromatic Molecules to Air-Water and Phospholipid Interfaces Mediated by Non-Hydrophobic Interactions. J. Phys. Chem. B.

[375] Nandi S., Ghosh B., Ghosh M., Layek S., Nandi P.K., Sarkar N. (2021). Phenylalanine Interacts with Oleic Acid-Based Vesicle Membrane. Understanding the Molecular Role of Fibril–Vesicle Interaction under the Context of Phenylketonuria. J. Phys. Chem. B.

[376] MacCallum J.L., Bennett W.F.D., Tieleman D.P. (2008). Distribution of Amino Acids in a Lipid Bilayer from Computer Simulations. Biophys. J..

[377] Cardenas A.E., Anderson C.M., Elber R., Webb L.J. (2019). Partition of Positively and Negatively Charged Tryptophan Ions in Membranes with Inverted Phospholipid Heads: Simulations and Experiments. J. Phys. Chem. B.

[378] Robinson M., Turnbull S., Lee B.Y., Leonenko Z. (2020). The effects of melatonin, serotonin, tryptophan and NAS on the biophysical properties of DPPC monolayers. Biochim. Biophys. Acta-Biomembr..

[379] Pokhrel N., Maibaum L. (2018). Free Energy Calculations of Membrane Permeation: Challenges Due to Strong Headgroup-Solute Interactions. J. Chem. Theory Comput..

[380] Genheden S., Eriksson L.A. (2016). Estimation of Liposome Penetration Barriers of Drug Molecules with All-Atom and Coarse-Grained Models. J. Chem. Theory Comput..

[381] Magarkar A., Parkkila P., Viitala T., Lajunen T., Mobarak E., Licari G., Cramariuc O., Vauthey E., Róg T., Bunker A. (2018). Membrane bound COMT isoform is an interfacial enzyme: General mechanism and new drug design paradigm. Chem. Commun..

[382] Megariotis G., Romanos N., Avramopoulos A., Mikaelian G., Theodorou D.N. (2021). In silico study of levodopa in hydrated lipid bilayers at the atomistic level. J. Mol. Graph. Model..

[383] Garcia A., Pochinda S., Elgaard-Jørgensen P.N., Khandelia H., Clarke R.J. (2019). Evidence for ATP Interaction with Phosphatidylcholine Bilayers. Langmuir.

[384] Sasidharan S., Pochinda S., Elgaard-Jørgensen P.N., Rajamani S., Khandelia H., Raghunathan V.A. (2019). Interaction of the mononucleotide UMP with a fluid phospholipid bilayer. Soft Matter.

[385] Kato A., Tsuji A., Yanagisawa M., Saeki D., Juni K., Morimoto Y., Yoshikawa K. (2010). Phase separation on a phospholipid membrane inducing a characteristic localization of DNA accompanied by its structural transition. J. Phys. Chem. Lett..

[386] Link K.A., Spurzem G.N., Tuladhar A., Chase Z., Wang Z., Wang H., Walker R.A. (2019). Cooperative Adsorption of Trehalose to DPPC Monolayers at the Water-Air Interface Studied with Vibrational Sum Frequency Generation. J. Phys. Chem. B.

[387] Gupta R., Badhe Y., Rai B., Mitragotri S. (2020). Molecular mechanism of the skin permeation enhancing effect of ethanol: A molecular dynamics study. RSC Adv..

[388] Paloncýová M., Navrátilová V., Berka K., Laio A., Otyepka M. (2016). Role of Enzyme Flexibility in Ligand Access and Egress to Active Site: Bias-Exchange Metadynamics Study of 1,3,7-Trimethyluric Acid in Cytochrome P450 3A4. J. Chem. Theory Comput..

[389] Postila P.A., Róg T. (2020). A perspective: Active role of lipids in neurotransmitter dynamics. Mol. Neurobiol..

[390] Orłowski A., Grzybek M., Bunker A., Pasenkiewicz-Gierula M., Vattulainen I., Männistö P.T., Róg T. (2012). Strong preferences of dopamine and l -dopa towards lipid head group: Importance of lipid composition and implication for neurotransmitter metabolism. J. Neurochem..

[391] Shen C., Xue M., Qiu H., Guo W. (2017). Insertion of Neurotransmitters into a Lipid Bilayer Membrane and Its Implication on Membrane Stability: A Molecular Dynamics Study. Chem. Phys. Chem..

[392] Mokkila S., Postila P.A., Rissanen S., Juhola H., Vattulainen I., Róg T. (2017). Calcium Assists Dopamine Release by Preventing Aggregation on the Inner Leaflet of Presynaptic Vesicles. ACS Chem. Neurosci..

[393] Juhola H., Postila P.A., Rissanen S., Lolicato F., Vattulainen I., Róg T. (2018). Negatively Charged Gangliosides Promote Membrane Association of Amphipathic Neurotransmitters. Neuroscience.

[394] Lolicato F., Juhola H., Zak A., Postila P.A., Saukko A., Rissanen S., Enkavi G., Vattulainen I., Kepczynski M., Róg T. (2020). Membrane-Dependent Binding and Entry Mechanism of Dopamine into Its Receptor. ACS Chem. Neurosci..

[395] Parkkila P., Viitala T. (2020). Partitioning of Catechol Derivatives in Lipid Membranes: Implications for Substrate Specificity to Catechol- O-methyltransferase. ACS Chem. Neurosci..

[396] Biswas B., Singh P.C. (2021). Restructuring of Membrane Water and Phospholipids in Direct Interaction of Neurotransmitters with Model Membranes Associated with Synaptic Signaling: Interface-Selective Vibrational Sum Frequency Generation Study. J. Phys. Chem. Lett..

[397] Megariotis G., Romanos N.A., Theodorou D.N. (2021). Molecular simulations of dopamine in a lipid bilayer. AIP Conf. Proc..

[398] Peters G.H., Wang C., Cruys-Bagger N., Velardez G.F., Madsen J.J., Westh P. (2013). Binding of serotonin to lipid membranes. J. Am. Chem. Soc..

[399] Josey B.P., Heinrich F., Silin V., Lösche M. (2020). Association of Model Neurotransmitters with Lipid Bilayer Membranes. Biophys. J..

[400] Dey S., Surendran D., Enberg O., Gupta A., Fanibunda S.E., Das A., Maity B.K., Dey A., Visvakarma V., Kallianpur M. (2021). Altered Membrane Mechanics Provides a Receptor-Independent Pathway for Serotonin Action. Chem.—A Eur. J..

[401] 4Bochicchio A., Brandner A.F., Engberg O., Huster D., Böckmann R.A. (2020). Spontaneous Membrane Nanodomain Formation in the Absence or Presence of the Neurotransmitter Serotonin. Front. Cell Dev. Biol..

[402] Engberg O., Bochicchio A., Brandner A.F., Gupta A., Dey S., Böckmann R.A., Maiti S., Huster D. (2020). Serotonin Alters the Phase Equilibrium of a Ternary Mixture of Phospholipids and Cholesterol. Front. Physiol..

[403] Choi Y., Attwood S.J., Hoopes M.I., Drolle E., Karttunen M., Leonenko Z. (2014). Melatonin directly interacts with cholesterol and alleviates cholesterol effects in dipalmitoylphosphatidylcholine monolayers. Soft Matter.

[404] Drolle E., Kučerka N., Hoopes M.I., Choi Y., Katsaras J., Karttunen M., Leonenko Z. (2013). Effect of melatonin and cholesterol on the structure of DOPC and DPPC membranes. Biochim. Biophys. Acta-Biomembr..

[405] Kondela T., Dushanov E., Vorobyeva M., Mamatkulov K., Drolle E., Soloviov D., Hrubovčák P., Kholmurodov K., Arzumanyan G., Leonenko Z. (2021). Investigating the competitive effects of cholesterol and melatonin in model lipid membranes. Biochim. Biophys. Acta-Biomembr..

[406] Tejwani R.W., Davis M.E., Anderson B.D., Stouch T.R. (2011). An atomic and molecular view of the depth dependence of the free energies of solute transfer from water into lipid bilayers. Mol. Pharm..

[407] Wittmann H.J., Strasser A. (2015). Binding pathway of histamine to the hH4R, observed by unconstrained molecular dynamics. Bioorganic Med. Chem. Lett..

[408] Hackett J.C. (2018). Membrane-embedded substrate recognition by cytochrome P450 3A4. J. Biol. Chem..

[409] Aleskndrany A., Sahin I. (2020). The effects of Levothyroxine on the structure and dynamics of DPPC liposome: FTIR and DSC studies. Biochim. Biophys. Acta-Biomembr..

[410] Gc J.B., Szlenk C.T., Gao J., Dong X., Wang Z., Natesan S. (2020). Molecular Dynamics Simulations Provide Insight into the Loading Efficiency of Proresolving Lipid Mediators Resolvin D1 and D2 in Cell Membrane-Derived Nanovesicles. Mol. Pharm..

[411] Devarajan A., Kim Y.C., Isakovic A.F., Gater D.L. (2021). Effect of cholecalciferol on unsaturated model membranes. Chem. Phys. Lipids.

[412] Sofferman D.L., Konar A., Mastron J.N., Spears K.G., Cisneros C., Smith A.C., Tapavicza E., Sension R.J. (2021). Probing the Formation and Conformational Relaxation of Previtamin D3and Analogues in Solution and in Lipid Bilayers. J. Phys. Chem. B.

[413] Chipot C., Comer J. (2016). Subdiffusion in Membrane Permeation of Small Molecules. Sci. Rep..

[414] Comer J., Schulten K., Chipot C. (2017). Permeability of a Fluid Lipid Bilayer to Short-Chain Alcohols from First Principles. J. Chem. Theory Comput..

[415] Carpenter T.S., Parkin J., Khalid S. (2016). The Free Energy of Small Solute Permeation through the Escherichia coli Outer Membrane Has a Distinctly Asymmetric Profile. J. Phys. Chem. Lett..

[416] Wang Y., Gallagher E., Jorgensen C., Troendle E.P., Hu D., Searson P.C., Ulmschneider M.B. (2019). An experimentally validated approach to calculate the blood-brain barrier permeability of small molecules. Sci. Rep..

[417] Gupta R., Sridhar D.B., Rai B. (2016). Molecular Dynamics Simulation Study of Permeation of Molecules through Skin Lipid Bilayer. J. Phys. Chem. B.

[418] Kumari P., Kumari M., Kashyap H.K. (2019). Counter-effects of Ethanol and Cholesterol on the Heterogeneous PSM-POPC Lipid Membrane: A Molecular Dynamics Simulation Study. J. Phys. Chem. B.

[419] Eslami H., Das S., Zhou T., Müller-Plathe F. (2020). How Alcoholic Disinfectants Affect Coronavirus Model Membranes: Membrane Fluidity, Permeability, and Disintegration. J. Phys. Chem. B.

[420] Kumari P., Kaur S., Sharma S., Kashyap H.K. (2018). Impact of amphiphilic molecules on the structure and stability of homogeneous sphingomyelin bilayer: Insights from atomistic simulations. J. Chem. Phys..

[421] Menichetti R., Kremer K., Bereau T. (2018). Efficient potential of mean force calculation from multiscale simulations: Solute insertion in a lipid membrane. Biochem. Biophys. Res. Commun..

[422] Algaba J., Mĺguez J.M., Gómez-Álvarez P., Mejĺa A., Blas F.J. (2020). Preferential Orientations and Anomalous Interfacial Tensions in Aqueous Solutions of Alcohols. J. Phys. Chem. B.

[423] Hossain S., Joyce P., Parrow A., Jõemetsa S., Höök F., Larsson P., Bergström C.A.S. (2020). Influence of Bile Composition on Membrane Incorporation of Transient Permeability Enhancers. Mol. Pharm..

[424] Terakosolphan W., Trick J.L., Royall P.G., Rogers S.E., Lamberti O., Lorenz C.D., Forbes B., Harvey R.D. (2018). Glycerol Solvates DPPC Headgroups and Localizes in the Interfacial Regions of Model Pulmonary Interfaces Altering Bilayer Structure. Langmuir.

[425] Ferreira J.V.N., Capello T.M., Siqueira L.J.A., Lago J.H.G., Caseli L. (2016). Mechanism of Action of Thymol on Cell Membranes Investigated through Lipid Langmuir Monolayers at the Air–Water Interface and Molecular Simulation. Langmuir.

[426] Tong X., Moradipour M., Novak B., Kamali P., Asare S.O., Knutson B.L., Rankin S.E., Lynn B.C., Moldovan D. (2019). Experimental and Molecular Dynamics Simulation Study of the Effects of Lignin Dimers on the Gel-to-Fluid Phase Transition in DPPC Bilayers. J. Phys. Chem. B.

[427] Vermaas J.V., Dixon R.A., Chen F., Mansfield S.D., Boerjan W., Ralph J., Crowley M.F., Beckham G.T. (2019). Passive membrane transport of lignin-related compounds. Proc. Natl. Acad. Sci. USA..

[428] Moradipour M., Tong X., Novak B., Kamali P., Asare S.O., Lynn B.C., Moldovan D., Rankin S.E., Knutson B.L. (2021). Interaction of lignin dimers with model cell membranes: A quartz crystal microbalance and molecular dynamics simulation study. Biointerphases.

[429] Reis A., Soares S., Sousa C.F., Dias R., Gameiro P., Soares S., de Freitas V. (2020). Interaction of polyphenols with model membranes: Putative implications to mouthfeel perception. Biochim. Biophys. Acta-Biomembr..

[430] Gurtovenko A.A., Mukhamadiarov E.I., Kostritskii A.Y., Karttunen M. (2018). Phospholipid-Cellulose Interactions: Insight from Atomistic Computer Simulations for Understanding the Impact of Cellulose-Based Materials on Plasma Membranes. J. Phys. Chem. B.

[431] Gurtovenko A.A., Karttunen M. (2019). Controlled On-Off Switching of Tight-Binding Hydrogen Bonds between Model Cell Membranes and Acetylated Cellulose Surfaces. Langmuir.

[432] Sapoń K., Janas T., Sikorski A.F., Janas T. (2019). Polysialic acid chains exhibit enhanced affinity for ordered regions of membranes. Biochim. Biophys. Acta-Biomembr..

[433] Miguel V., Sánchez-Borzone M.E., García D.A. (2018). Interaction of gabaergic ketones with model membranes: A molecular dynamics and experimental approach. Biochim. Biophys. Acta-Biomembr..

[434] Shahoei R., Tajkhorshid E. (2020). Menthol Binding to the Human α4β2 Nicotinic Acetylcholine Receptor Facilitated by Its Strong Partitioning in the Membrane. J. Phys. Chem. B.

[435] Vermaas J.V., Bentley G.J., Beckham G.T., Crowley M.F. (2018). Membrane Permeability of Terpenoids Explored with Molecular Simulation. J. Phys. Chem. B.

[436] Paloncýová M., Berka K., Otyepka M. (2012). Convergence of free energy profile of coumarin in lipid bilayer. J. Chem. Theory Comput..

[437] Duncan K.M., Casey A., Gobrogge C.A., Trousdale R.C., Piontek S.M., Cook M.J., Steel W.H., Walker R.A. (2020). Coumarin Partitioning in Model Biological Membranes: Limitations of log P as a Predictor. J. Phys. Chem. B.

[438] Dufourc E.J. (2021). Wine tannins, saliva proteins and membrane lipids. Biochim. Biophys. Acta-Biomembr..

[439] Tavagnacco L., Corucci G., Gerelli Y. (2021). Interaction of Caffeine with Model Lipid Membranes. J. Phys. Chem. B.

[440] Sherratt S.C.R., Villeneuve P., Durand E., Mason R.P. (2019). Rosmarinic acid and its esters inhibit membrane cholesterol domain formation through an antioxidant mechanism based, in nonlinear fashion, on alkyl chain length. Biochim. Biophys. Acta-Biomembr..

[441] Filipe H.A.L., Sousa C., Marquês J.T., Vila-Viçosa D., de Granada-Flor A., Viana A.S., Santos M.S.C.S., Machuqueiro M., de Almeida R.F.M. (2018). Differential targeting of membrane lipid domains by caffeic acid and its ester derivatives. Free Radic. Biol. Med..

[442] Huang J., Chen P.X., Rogers M.A., Wettig S.D. (2019). Investigating the phospholipid effect on the bioaccessibility of rosmarinic acid-phospholipid complex through a dynamic gastrointestinal in vitro model. Pharmaceutics.

[443] Colina J.R., Suwalsky M., Manrique-Moreno M., Petit K., Aguilar L.F., Jemiola-Rzeminska M., Strzalka K. (2019). An in vitro study of the protective effect of caffeic acid on human erythrocytes. Arch. Biochem. Biophys..

[444] Lopes R., Costa M., Ferreira M., Gameiro P., Fernandes S., Catarino C., Santos-Silva A., Paiva-Martins F. (2021). Caffeic acid phenolipids in the protection of cell membranes from oxidative injuries. Interaction with the membrane phospholipid bilayer. Biochim. Biophys. Acta-Biomembr..

[445] Gupta R., Mitra S., Chowdhury S., Das G., Priyadarshini R. (2021). Discerning perturbed assembly of lipids in a model membrane in presence of violacein. Biochim. Biophys. Acta-Biomembr..

[446] Cauz A.C.G., Carretero G.P.B., Saraiva G.K.V., Park P., Mortara L., Cuccovia I.M., Brocchi M., Gueiros-Filho F.J. (2019). Violacein Targets the Cytoplasmic Membrane of Bacteria. ACS Infect. Dis..

[447] Bouhlel Z., Arnold A.A., Deschênes J.-S., Mouget J.-L., Warschawski D.E., Tremblay R., Marcotte I. (2021). Investigating the action of the microalgal pigment marennine on Vibrio splendidus by in vivo 2H and 31P solid-state NMR. Biochim. Biophys. Acta-Biomembr..

[448] Neves M.C., Filipe H.A.L., Reis R.L., Ramalho J.P.P., Coreta-Gomes F., Moreno M.J., Loura L.M.S. (2019). Interaction of bile salts with lipid bilayers: An atomistic molecular dynamics study. Front. Physiol..

[449] Dubinin M.V., Semenova A.A., Ilzorkina A.I., Mikheeva I.B., Yashin V.A., Penkov N.V., Vydrina V.A., Ishmuratov G.Y., Sharapov V.A., Khoroshavina E.I. (2020). Effect of betulin and betulonic acid on isolated rat liver mitochondria and liposomes. Biochim. Biophys. Acta-Biomembr..

[450] Jurek I., Góral I., Mierzyńska Z., Moniuszko-Szajwaj B., Wojciechowski K. (2019). Effect of synthetic surfactants and soapwort (*Saponaria officinalis* L.) extract on skin-mimetic model lipid monolayers. Biochim. Biophys. Acta-Biomembr..

[451] Geisler R., Dargel C., Hellweg T. (2020). The biosurfactant β-aescin: A review on the physico-chemical properties and its interaction with lipid model membranes and langmuir monolayers. Molecules.

[452] Selyutina O.Y., Apanasenko I.E., Kim A.V., Shelepova E.A., Khalikov S.S., Polyakov N.E. (2016). Spectroscopic and molecular dynamics characterization of glycyrrhizin membrane-modifying activity. Colloids Surf. B Biointerfaces.

[453] Selyutina O.Y., Shelepova E.A., Paramonova E.D., Kichigina L.A., Khalikov S.S., Polyakov N.E. (2020). Glycyrrhizin-induced changes in phospholipid dynamics studied by 1H NMR and MD simulation. Arch. Biochem. Biophys..

[454] Wadhwa R., Yadav N.S., Katiyar S.P., Yaguchi T., Lee C., Ahn H., Yun C.-O., Kaul S.C., Sundar D. (2021). Molecular dynamics simulations and experimental studies reveal differential permeability of withaferin-A and withanone across the model cell membrane. Sci. Rep..

[455] Hurst D.P., Grossfield A., Lynch D.L., Feller S., Romo T.D., Gawrisch K., Pitman M.C., Reggio P.H. (2010). A lipid pathway for ligand binding is necessary for a cannabinoid G protein-coupled receptor. J. Biol. Chem..

[456] Kim S., Voth G.A. (2021). Physical Characterization of Triolein and Implications for Its Role in Lipid Droplet Biogenesis. J. Phys. Chem. B.

[457] Maiti A., Daschakraborty S. (2021). Effect of TMAO on the Structure and Phase Transition of Lipid Membranes: Potential Role of TMAO in Stabilizing Cell Membranes under Osmotic Stress. J. Phys. Chem. B.

[458] Craig R.A., Garrison C.E., Nguyen P.T., Yarov-Yarovoy V., Du Bois J. (2020). Veratridine: A Janus-Faced Modulator of Voltage-Gated Sodium Ion Channels. ACS Chem. Neurosci..

[459] Vazdar M., Jurkiewicz P., Hof M., Jungwirth P., Cwiklik L. (2012). Behavior of 4-hydroxynonenal in phospholipid membranes. J. Phys. Chem. B.

[460] Pazin W.M., da Silva Olivier D., Vilanova N., Ramos A.P., Voets I.K., Soares A.E.E., Ito A.S. (2017). Interaction of Artepillin C with model membranes. Eur. Biophys. J..

[461] Nie R., Dang M., Ge Z., Huo Y., Yu B., Tang S. (2021). Interactions of chlorogenic acid and isochlorogenic acid A with model lipid bilayer membranes: Insights from molecular dynamics simulations. Chem. Phys. Lipids.

[462] Zhu W., Xiong L., Peng J., Deng X., Gao J., Li C. (2016). Molecular Insight into Affinities of Gallated and Nongallated Proanthocyanidins Dimers to Lipid Bilayers. Sci. Rep..

[463] Zhu W., Khalifa I., Peng J., Li C. (2018). Position and orientation of gallated proanthocyanidins in lipid bilayer membranes: Influence of polymerization degree and linkage type. J. Biomol. Struct. Dyn..

[464] Galiano V., Villalaín J. (2015). Oleuropein aglycone in lipid bilayer membranes. A molecular dynamics study. Biochim. Biophys. Acta-Biomembr..

[465] Hossain S.I., Saha S.C., Deplazes E. (2021). Phenolic compounds alter the ion permeability of phospholipid bilayers via specific lipid interactions. Phys. Chem. Chem. Phys..

[466] Wang R., Zhu W., Peng J., Li K., Li C. (2020). Lipid rafts as potential mechanistic targets underlying the pleiotropic actions of polyphenols. Crit. Rev. Food Sci. Nutr..

[467] Reis A., Perez-Gregorio R., Mateus N., de Freitas V. (2021). Interactions of dietary polyphenols with epithelial lipids: Advances from membrane and cell models in the study of polyphenol absorption, transport and delivery to the epithelium. Crit. Rev. Food Sci. Nutr..

[468] Šturm L., Poklar Ulrih N. (2021). Basic Methods for Preparation of Liposomes and Studying Their Interactions with Different Compounds, with the Emphasis on Polyphenols. Int. J. Mol. Sci..

[469] Reis A., de Freitas V. (2020). When polyphenols meet lipids: Challenges in membrane biophysics and opportunities in epithelial lipidomics. Food Chem..

[470] Kholina E.G., Kovalenko I.B., Bozdaganyan M.E., Strakhovskaya M.G., Orekhov P.S. (2020). Cationic Antiseptics Facilitate Pore Formation in Model Bacterial Membranes. J. Phys. Chem. B.

[471] Sowlati-Hashjin S., Carbone P., Karttunen M. (2020). Insights into the Polyhexamethylene Biguanide (PHMB) Mechanism of Action on Bacterial Membrane and DNA: A Molecular Dynamics Study. J. Phys. Chem. B.

[472] Felsztyna I., Sánchez-Borzone M.E., Miguel V., García D.A. (2020). The insecticide fipronil affects the physical properties of model membranes: A combined experimental and molecular dynamics simulations study in Langmuir monolayers. Biochim. Biophys. Acta-Biomembr..

[473] DeMarco K.R., Bekker S., Clancy C.E., Noskov S.Y., Vorobyov I. (2018). Digging into lipid membrane permeation for cardiac ion channel blocker d-sotalol with all-atom simulations. Front. Pharmacol..

[474] Ribeiro R.P., Coimbra J.T.S., Ramos M.J., Fernandes P.A. (2017). Diffusion of the small, very polar, drug piracetam through a lipid bilayer: An MD simulation study. Theor. Chem. Acc..

[475] Ermilova I., Stenberg S., Lyubartsev A.P. (2017). Quantum chemical and molecular dynamics modelling of hydroxylated polybrominated diphenyl ethers. Phys. Chem. Chem. Phys..

[476] Chen L., Chen J., Zhou G., Wang Y., Xu C., Wang X. (2016). Molecular Dynamics Simulations of the Permeation of Bisphenol A and Pore Formation in a Lipid Membrane. Sci. Rep..

[477] Shen Z., Ge J., Ye H., Tang S., Li Y. (2020). Cholesterol-like Condensing Effect of Perfluoroalkyl Substances on a Phospholipid Bilayer. J. Phys. Chem. B.

[478] Yang H., Li H., Liu L., Zhou Y., Long X. (2019). Molecular Simulation Studies on the Interactions of 2,4,6-Trinitrotoluene and Its Metabolites with Lipid Membranes. J. Phys. Chem. B.

[479] Yang H., Li H., Zhou M., Wei T., Tang C., Liu L., Zhou Y., Long X. (2020). A relationship between membrane permeation and partitioning of nitroaromatic explosives and their functional groups. A computational study. Phys. Chem. Chem. Phys..

[480] Golius A., Gorb L., Isayev O., Leszczynski J. (2019). Diffusion of energetic compounds through biological membrane: Application of classical MD and COSMOmic approximations. J. Biomol. Struct. Dyn..

[481] Yang H., Zhou M., Li H., Wei T., Tang C., Zhou Y., Long X. (2020). Effects of Low-level Lipid Peroxidation on the Permeability of Nitroaromatic Molecules across a Membrane: A Computational Study. ACS Omega.

[482] Rózsa Z.B., Németh L.J., Jójárt B., Nehéz K., Viskolcz B., Szöri M. (2019). Molecular Dynamics and Metadynamics Insights of 1,4-Dioxane-Induced Structural Changes of Biomembrane Models. J. Phys. Chem. B.

[483] Stachowicz-Kuśnierz A., Trojan S., Cwiklik L., Korchowiec B., Korchowiec J. (2017). Modeling Lung Surfactant Interactions with Benzo[a]pyrene. Chem.—A Eur. J..

[484] Xue Z., Sun Q., Zhang L., Kang Z., Liang L., Wang Q., Shen J.W. (2019). Graphene quantum dot assisted translocation of drugs into a cell membrane. Nanoscale.

[485] Raczyński P., Górny K., Bełdowski P., Yuvan S., Dendzik Z. (2020). Application of graphene as a nanoindenter interacting with phospholipid membranes-computer simulation study. J. Phys. Chem. B.

[486] Zhu X., Li N., Huang C., Li Z., Fan J. (2020). Membrane Perturbation and Lipid Flip-Flop Mediated by Graphene Nanosheet. J. Phys. Chem. B.

[487] Moore T.C., Yang A.H., Ogungbesan O., Hartkamp R., Iacovella C.R., Zhang Q., McCabe C. (2019). Influence of single-stranded DNA Coatings on the Interaction between Graphene Nanoflakes and Lipid Bilayers. J. Phys. Chem. B.

[488] Kaminari A., Nikoli E., Athanasopoulos A., Sakellis E., Sideratou Ζ. (2021). Engineering Mitochondriotropic Carbon Dots for Targeting Cancer Cells. Pharmaceuticals.

[489] Chen Y., Zhang W., Huang C., Feng M., Yang Y., Gou Y. (2021). Destructive Extraction and Enhanced Diffusion of Phospholipids on Lipid Membranes by Phosphorene Oxide Nanosheets. J. Phys. Chem. B.

[490] Ou L., Corradi V., Tieleman D.P., Liang Q. (2020). Atomistic simulations on interactions between amphiphilic Janus nanoparticles and lipid bilayers: Effects of lipid ordering and leaflet asymmetry. J. Phys. Chem. B.

[491] Das M., Dahal U., Mesele O., Liang D., Cui Q. (2019). Molecular dynamics simulation of interaction between functionalized nanoparticles with lipid membranes: Analysis of coarse-grained models. J. Phys. Chem. B.

[492] 4Salassi S., Canepa E., Ferrando R., Rossi G. (2019). Anionic nanoparticle-lipid membrane interactions: The protonation of anionic ligands at the membrane surface reduces membrane disruption. RSC. Adv..

[493] Ivanov M., Lyubartsev A.P. (2021). Atomistic Molecular Dynamics Simulations of Lipids Near TiO 2 Nanosurfaces. J. Phys. Chem. B.

[494] Zhang L., Chen H., Xie J., Becton M., Wang X. (2019). Interplay of Nanoparticle Rigidity and Its Translocation Ability through Cell Membrane. J. Phys. Chem. B.

[495] Nalakarn P., Boonnoy P., Nisoh N., Karttunen M., Wong-ekkabut J. (2019). Dependence of fullerene aggregation on lipid saturation due to a balance between entropy and enthalpy. Sci. Rep..

[496] Nisoh N., Jarerattanachat V., Karttunen M., Wong-ekkabut J. (2020). Formation of aggregates, icosahedral structures and percolation clusters of fullerenes in lipids bilayers: The key role of lipid saturation. Biochim. Biophys. Acta-Biomembr..

[497] Nisoh N., Karttunen M., Monticelli L., Wong-Ekkabut J. (2015). Lipid monolayer disruption caused by aggregated carbon nanoparticles. RSC Adv..

[498] Alves E.D., Colherinhas G., Mendanha S.A. (2020). Assessing the DOPC-cholesterol interactions and their influence on fullerene C60 partitioning in lipid bilayers. J. Mol. Liq..

[499] Jiménez-Jiménez C., Manzano M., Vallet-Regí M. (2020). Nanoparticles coated with cell membranes for biomedical applications. Biology.

[500] Zhang W., Metzger J.M., Hackel B.J., Bates F.S., Lodge T.P. (2020). Influence of the Headgroup on the Interaction of Poly(ethylene oxide)-Poly(propylene oxide) Block Copolymers with Lipid Bilayers. J. Phys. Chem. B.

[501] Gurtovenko A.A. (2019). Molecular-Level Insight into the Interactions of DNA/Polycation Complexes with Model Cell Membranes. J. Phys. Chem. B.

[502] Zaki A.M., Carbone P. (2019). Amphiphilic copolymers change the nature of the ordered-to-disordered phase transition of lipid membranes from discontinuous to continuous. Phys. Chem. Chem. Phys..

[503] Houang E.M., Haman K.J., Kim M., Zhang W., Lowe D.A., Sham Y.Y., Lodge T.P., Hackel B.J., Bates F.S., Metzger J.M. (2017). Chemical End Group Modified Diblock Copolymers Elucidate Anchor and Chain Mechanism of Membrane Stabilization. Mol. Pharm..

[504] Pérez-Sánchez G., Vicente F.A., Schaeffer N., Cardoso I.S., Ventura S.P.M., Jorge M., Coutinho J.A.P. (2020). Unravelling the interactions between surface-active ionic liquids and triblock copolymers for the design of thermal responsive systems. J. Phys. Chem. B.

[505] Ileri Ercan N., Stroeve P., Tringe J.W., Faller R. (2016). Understanding the Interaction of Pluronics L61 and L64 with a DOPC Lipid Bilayer: An Atomistic Molecular Dynamics Study. Langmuir.

[506] 5Qiao J., Purro M., Liu Z., Xiong M.P. (2021). Effects of Polyethyelene Glycol-Desferrioxamine:Gallium Conjugates on Pseudomonas aeruginosa Outer Membrane Permeability and Vancomycin Potentiation. Mol. Pharm..

[507] Martin A., Tomasini M., Kholodovych V., Gu L., Sommerfeld S., Uhrich K., Murthy N., Welsh W., Moghe P. (2015). Carbohydrate-Derived Amphiphilic Macromolecules: A Biophysical Structural Characterization and Analysis of Binding Behaviors to Model Membranes. J. Funct. Biomater..

[508] Lehtinen J., Magarkar A., Stepniewski M., Hakola S., Bergman M., Róg T., Yliperttula M., Urtti A., Bunker A. (2012). Analysis of cause of failure of new targeting peptide in PEGylated liposome: Molecular modeling as rational design tool for nanomedicine. Eur. J. Pharm. Sci..

[509] Magarkar A., Róg T., Bunker A. (2017). A computational study suggests that replacing PEG with PMOZ may increase exposure of hydrophobic targeting moiety. Eur. J. Pharm. Sci..

[510] Jeong H., Hwang J., Lee H., Hammond P.T., Choi J., Hong J. (2017). In vitro blood cell viability profiling of polymers used in molecular assembly. Sci. Rep..

[511] Kumari P., Kashyap H.K. (2019). Sensitivity and Resilience of Phosphatidylcholine and Phosphatidylethanolamine Lipid Membranes against Cholinium Glycinate Biocompatible Ionic Liquid. J. Phys. Chem. B.

[512] Kumari M., Gupta A., Shobhna, Kashyap H.K. (2020). Molecular Dynamics Evaluation of the Effect of Cholinium Phenylalaninate Biocompatible Ionic Liquid on Biomimetic Membranes. J. Phys. Chem. B.

[513] Kumari P., Faraone A., Kelley E.G., Benedetto A. (2021). Stiffening Effect of the [Bmim][Cl] Ionic Liquid on the Bending Dynamics of DMPC Lipid Vesicles. J. Phys. Chem. B.

[514] Cardoso R.M.S., Martins P.A.T., Ramos C.V., Cordeiro M.M., Leote R.J.B., Razi Naqvi K., Vaz W.L.C., Moreno M.J. (2020). Effect of dipole moment on amphiphile solubility and partition into liquid ordered and liquid disordered phases in lipid bilayers. Biochim. Biophys. Acta-Biomembr..

[515] Kofod C.S., Prioli S., Hornum M., Kongsted J., Reinholdt P. (2020). Computational Characterization of Novel Malononitrile Variants of Laurdan with Improved Photophysical Properties for Sensing in Membranes. J. Phys. Chem. B.

[516] Do Canto A.M.T.M., Robalo J.R., Santos P.D., Carvalho A.J.P., Ramalho J.P.P., Loura L.M.S. (2016). Diphenylhexatriene membrane probes DPH and TMA-DPH: A comparative molecular dynamics simulation study. Biochim. Biophys. Acta-Biomembr..

[517] Filipe H.A.L., Santos L.S., Prates Ramalho J.P., Moreno M.J., Loura L.M.S. (2015). Behaviour of NBD-head group labelled phosphatidylethanolamines in POPC bilayers: A molecular dynamics study. Phys. Chem. Chem. Phys..

[518] Filipe H.A.L., Pokorná Š., Hof M., Amaro M., Loura L.M.S. (2019). Orientation of nitro-group governs the fluorescence lifetime of nitrobenzoxadiazole (NBD)-labeled lipids in lipid bilayers. Phys. Chem. Chem. Phys..

[519] Kulkarni R.U., Yin H., Pourmandi N., James F., Adil M.M., Schaffer D.V., Wang Y., Miller E.W. (2017). A Rationally Designed, General Strategy for Membrane Orientation of Photoinduced Electron Transfer-Based Voltage-Sensitive Dyes. ACS Chem. Biol..

[520] Chmielińska A., Stepien P., Bonarek P., Girych M., Enkavi G., Róg T., Dziedzicka-Wasylewska M., Polit A. (2021). Can di-4-ANEPPDHQ reveal the structural differences between nanodiscs and liposomes?. Biochim. Biophys. Acta-Biomembr..

[521] Bouquiaux C., Castet F., Champagne B. (2021). Unravelling the Effects of Cholesterol on the Second-Order Nonlinear Optical Responses of Di-8-ANEPPS Dye Embedded in Phosphatidylcholine Lipid Bilayers. J. Phys. Chem. B.

[522] Suhaj A., Gowland D., Bonini N., Owen D.M., Lorenz C.D. (2020). Laurdan and Di-4-ANEPPDHQ influence the properties of lipid membranes: A classical molecular dynamics and fluorescence study. J. Phys. Chem. B.

[523] Filipe H.A.L., Moreno M.J., Loura L.M.S. (2020). The secret lives of fluorescent membrane probes as revealed by molecular dynamics simulations. Molecules.

[524] Thomas D., Rubio V., Iragavarapu V., Guzman E., Pelletier O.B., Alamgir S., Zhang Q., Stawikowski M.J. (2021). Solvatochromic and pH-Sensitive Fluorescent Membrane Probes for Imaging of Live Cells. ACS Chem. Neurosci..

[525] Rissanen S., Grzybek M., Orłowski A., Róg T., Cramariuc O., Levental I., Eggeling C., Sezgin E., Vattulainen I. (2017). Phase partitioning of GM1 and its bodipy-labeled analog determine their different binding to Cholera Toxin. Front. Physiol..

[526] Filipe H.A.L., Moreno M.J., Róg T., Vattulainen I., Loura L.M.S. (2014). How to tackle the issues in free energy simulations of long amphiphiles interacting with lipid membranes: Convergence and local membrane deformations. J. Phys. Chem. B.

[527] Kepczynski M., Kumorek M., Stepniewski M., Róg T., Kozik B., Jamróz D., Bednar J., Nowakowska M. (2010). Behavior of 2,6-Bis(decyloxy)naphthalene inside lipid bilayer. J. Phys. Chem. B.

[528] Baig M.W., Pederzoli M., Jurkiewicz P., Cwiklik L., Pittner J. (2018). Orientation of Laurdan in phospholipid bilayers influences its fluorescence: Quantum mechanics and classical molecular dynamics study. Molecules.

[529] Licari G., Cwiklik L., Jungwirth P., Vauthey E. (2017). Exploring Fluorescent Dyes at Biomimetic Interfaces with Second Harmonic Generation and Molecular Dynamics. Langmuir.

[530] Zhu Q., Lu Y., He X., Liu T., Chen H., Wang F., Zheng D., Dong H., Ma J. (2017). Entropy and Polarity Control the Partition and Transportation of Drug-like Molecules in Biological Membrane. Sci. Rep..

[531] Wen W., Luo J., Li P., Huang W., Wang P., Xu S. (2021). Benzaldehyde, A New Absorption Promoter, Accelerating Absorption on Low Bioavailability Drugs Through Membrane Permeability. Front. Pharmacol..

[532] Song Y., Lee J.H., Jung I., Seo B., Hwang H. (2020). Molecular Dynamics Simulations of Micelle Properties and Behaviors of Sodium Lauryl Ether Sulfate Penetrating Ceramide and Phospholipid Bilayers. J. Phys. Chem. B.

[533] Liu J., Li X., Hou J., Liu F. (2020). Electric-Field-Induced Interface Behavior of Dodecyl Sulfate with Large Organic Counterions: A Molecular Dynamics Study. J. Phys. Chem. B.

[534] Liu Z., Ren X., Tan R., Chai Z., Wang D. (2020). Key Factors Determining Efficiency of Liquid−Liquid Extraction: Implications from Molecular Dynamics Simulations of Biphasic Behaviors of CyMe4-BTPhen and Its Am(III) Complexes. J. Phys. Chem. B.

[535] de Ménorval M.A., Mir L.M., Fernández M.L., Reigada R. (2012). Effects of dimethyl sulfoxide in cholesterol-containing lipid membranes: A comparative study of experiments in silico and with cells. PLoS ONE.

[536] Coimbra J.T.S., Brás N.F., Fernandes P.A., Rangel M., Ramos M.J. (2018). Membrane partition of bis-(3-hydroxy-4-pyridinonato) zinc(ii) complexes revealed by molecular dynamics simulations. RSC Adv..

[537] Zappacosta R., Aschi M., Ammazzalorso A., Di Profio P., Fontana A., Siani G. (2019). Embedding calix [4] resorcinarenes in liposomes: Experimental and computational investigation of the effect of resorcinarene inclusion on liposome properties and stability. Biochim. Biophys. Acta-Biomembr..

[538] Sánchez-Borzone M.E., Mariani M.E., Miguel V., Gleiser R.M., Odhav B., Venugopala K.N., García D.A. (2017). Membrane effects of dihydropyrimidine analogues with larvicidal activity. Colloids Surf. B Biointerfaces.

[539] Duša F., Chen W., Witos J., Rantamäki A.H., King A.W.T., Sklavounos E., Roth M., Wiedmer S.K. (2020). Immobilization of natural lipid biomembranes and their interactions with choline carboxylates. A nanoplasmonic sensing study. Biochim. Biophys. Acta-Biomembr..

[540] Lopes R., Costa M., Ferreira M., Gameiro P., Paiva-Martins F. (2021). A new family of hydroxytyrosol phenolipids for the antioxidant protection of liposomal systems. Biochim. Biophys. Acta-Biomembr..

[541] Schmidt A., Lenzig P., Oslender-Bujotzek A., Kusch J., Lucas S.D., Gründer S., Wiemuth D. (2014). The Bile Acid-Sensitive Ion Channel (BASIC) Is activated by alterations of its membrane environment. PLoS ONE.

[542] Lundbæk J.A., Koeppe R.E., Andersen O.S. (2010). Amphiphile regulation of ion channel function by changes in the bilayer spring constant. Proc. Natl. Acad. Sci. USA.

[543] Yin Q., Wang R., Yang S., Wu Z., Guo S., Dai X., Qiao Y., Shi X. (2017). Influence of temperature on transdermal penetration enhancing mechanism of borneol: A multi-scale study. Int. J. Mol. Sci..

[544] Liu X., Liu M., Liu C., Quan P., Zhao Y., Fang L. (2017). An insight into the molecular mechanism of the temporary enhancement effect of isopulegol decanoate on the skin. Int. J. Pharm..

[545] Li G., Yao P., Gong R., Li J., Liu P., Lonsdale R., Wu Q., Lin J., Zhu D., Reetz M.T. (2017). Simultaneous engineering of an enzyme’s entrance tunnel and active site: The case of monoamine oxidase MAO-N. Chem. Sci..

[546] Aragón-Muriel A., Liscano Y., Morales-Morales D., Polo-Cerón D., Oñate-Garzón J. (2021). A study of the interaction of a new benzimidazole schiff base with synthetic and simulated membrane models of bacterial and mammalian membranes. Membranes.

[547] Bakarić D., Carić D., Vazdar K., Vazdar M. (2019). Vibrational spectroscopy combined with molecular dynamics simulations as a tool for studying behavior of reactive aldehydes inserted in phospholipid bilayers. Chem. Phys. Lipids.

[548] Issack B.B., Peslherbe G.H. (2019). Accuracy and precision of simulated free energies: Water permeation of hydrated DPPC bilayers as a paradigm. Mol. Simul..

[549] Hub J.S., Winkler F.K., Merrick M., de Groot B.L. (2010). Potentials of Mean Force and Permeabilities for Carbon Dioxide, Ammonia, and Water Flux across a Rhesus Protein Channel and Lipid Membranes. J. Am. Chem. Soc..

[550] Olzynska A., Kulig W., Mikkolainen H., Czerniak T., Jurkiewicz P., Cwiklik L., Róg T., Hof M., Jungwirth P., Vattulainen I. (2020). Tail-Oxidized Cholesterol Enhances Membrane Permeability for Small Solutes. Langmuir.

[551] Bu B., Crowe M., Diao J., Ji B., Li D. (2018). Cholesterol suppresses membrane leakage by decreasing water penetrability. Soft Matter.

[552] Hong C., Tieleman D.P., Wang Y. (2014). Microsecond Molecular Dynamics Simulations of Lipid Mixing. Langmuir.

[553] Saito H., Shinoda W. (2011). Cholesterol effect on water permeability through DPPC and PSM lipid bilayers: A molecular dynamics study. J. Phys. Chem. B.

[554] Issack B.B., Peslherbe G.H. (2015). Effects of Cholesterol on the Thermodynamics and Kinetics of Passive Transport of Water through Lipid Membranes. J. Phys. Chem. B.

[555] Hartkamp R., Moore T.C., Iacovella C.R., Thompson M.A., Bulsara P.A., Moore D.J., McCabe C. (2018). Composition Dependence of Water Permeation Across Multicomponent Gel-Phase Bilayers. J. Phys. Chem. B.

[556] Wittmann H.J., Seifert R., Strasser A. (2014). Sodium binding to hH3R and hH4R—A molecular modeling study. J. Mol. Model..

[557] Plesnar E., Szczelina R., Subczynski W.K., Pasenkiewicz-Gierula M. (2018). Is the cholesterol bilayer domain a barrier to oxygen transport into the eye lens?. Biochim. Biophys. Acta-Biomembr..

[558] Yuan Y., Liu X., Liu T., Liu W., Zhu Y., Zhang H., Zhao C. (2020). Molecular dynamics exploring of atmosphere components interacting with lung surfactant phospholipid bilayers. Sci. Total Environ..

[559] Man V.H., Truong P.M., Li M.S., Wang J., Van-Oanh N.-T., Derreumaux P., Nguyen P.H. (2019). Molecular Mechanism of the Cell Membrane Pore Formation Induced by Bubble Stable Cavitation. J. Phys. Chem. B.

[560] Venable R.M., Krämer A., Pastor R.W. (2019). Molecular Dynamics Simulations of Membrane Permeability. Chem. Rev..

[561] Awoonor-Williams E., Rowley C.N. (2016). Molecular simulation of nonfacilitated membrane permeation. Biochim. Biophys. Acta-Biomembr..

[562] Amaro R.E., Mulholland A.J. (2018). Multiscale methods in drug design bridge chemical and biological complexity in the search for cures. Nat. Rev. Chem..

[563] Hannesschlaeger C., Horner A., Pohl P. (2019). Intrinsic Membrane Permeability to Small Molecules. Chem. Rev..

[564] Marrink S.-J., Berendsen H.J.C. (1994). Simulation of Water Transport through a Lipid Membrane Siewert-Jan. J. Phys. Chem..

[565] Ingram T., Storm S., Kloss L., Mehling T., Jakobtorweihen S., Smirnova I. (2013). Prediction of micelle/water and liposome/water partition coefficients based on molecular dynamics simulations, COSMO-RS, and COSMOmic. Langmuir.

[566] Schwöbel J.A.H., Ebert A., Bittermann K., Huniar U., Goss K.U., Klamt A. (2020). COSMO perm: Mechanistic Prediction of Passive Membrane Permeability for Neutral Compounds and Ions and Its pH Dependence. J. Phys. Chem. B.

[567] Turchi M., Kognole A.A., Kumar A., Cai Q., Lian G., Mackerell A.D. (2020). Predicting Partition Coefficients of Neutral and Charged Solutes in the Mixed SLES-Fatty Acid Micellar System. J. Phys. Chem. B.

[568] Bennion B.J., Be N.A., McNerney M.W., Lao V., Carlson E.M., Valdez C.A., Malfatti M.A., Enright H.A., Nguyen T.H., Lightstone F.C. (2017). Predicting a Drug’s Membrane Permeability: A Computational Model Validated with in Vitro Permeability Assay Data. J. Phys. Chem. B.

[569] Lomize A.L., Pogozheva I.D. (2019). Physics-Based Method for Modeling Passive Membrane Permeability and Translocation Pathways of Bioactive Molecules. J. Chem. Inf. Model..

[570] Fukunishi Y., Mashimo T., Kurosawa T., Wakabayashi Y., Nakamura H.K., Takeuchi K. (2020). Prediction of Passive Membrane Permeability by Semi-Empirical Method Considering Viscous and Inertial Resistances and Different Rates of Conformational Change and Diffusion. Mol. Inform..

[571] Bennett D.W.F., He S., Bilodeau C.L., Jones D., Sun D., Kim H., Allen J.E., Lightstone F.C., Ingólfsson H.I. (2020). Predicting small molecule transfer free energies by combining molecular dynamics simulations and deep learning. J. Chem. Inf. Model..

[572] Brocke S.A., Degen A., Mackerell A.D., Dutagaci B., Feig M. (2019). Prediction of Membrane Permeation of Drug Molecules by Combining an Implicit Membrane Model with Machine Learning. J. Chem. Inf. Model..

[573] Sun R., Han Y., Swanson J.M.J., Tan J.S., Rose J.P., Voth G.A. (2018). Molecular transport through membranes: Accurate permeability coefficients from multidimensional potentials of mean force and local diffusion constants. J. Chem. Phys..

[574] Sun R., Dama J.F., Tan J.S., Rose J.P., Voth G.A. (2016). Transition-Tempered Metadynamics Is a Promising Tool for Studying the Permeation of Drug-like Molecules through Membranes. J. Chem. Theory Comput..

[575] Tse C.H., Comer J., Sang Chu S.K., Wang Y., Chipot C. (2019). Affordable Membrane Permeability Calculations: Permeation of Short-Chain Alcohols through Pure-Lipid Bilayers and a Mammalian Cell Membrane. J. Chem. Theory Comput..

[576] Badaoui M., Kells A., Molteni C., Dickson C.J., Hornak V., Rosta E. (2018). Calculating Kinetic Rates and Membrane Permeability from Biased Simulations. J. Phys. Chem. B.

[577] Votapka L.W., Lee C.T., Amaro R.E. (2016). Two Relations to Estimate Membrane Permeability Using Milestoning. J. Phys. Chem. B.

[578] Lomize A.L., Hage J.M., Schnitzer K., Golobokov K., Lafaive M.B., Forsyth A.C., Pogozheva I.D. (2019). PerMM: A Web Tool and Database for Analysis of Passive Membrane Permeability and Translocation Pathways of Bioactive Molecules. J. Chem. Inf. Model..

[579] Sharifian G.M. (2021). Recent Experimental Developments in Studying Passive Membrane Transport of Drug Molecules. Mol. Pharm..

[580] Neuvonen M., Manna M., Mokkila S., Javanainen M., Róg T., Liu Z., Bittman R., Vattulainen I., Ikonen E. (2014). Enzymatic oxidation of cholesterol: Properties and functional effects of cholestenone in cell membranes. PLoS ONE.

[581] Róg T., Stimson L.M., Pasenkiewicz-Gierula M., Vattulainen I., Karttunen M. (2008). Replacing the cholesterol hydroxyl group with the ketone group facilitates sterol flip-flop and promotes membrane fluidity. J. Phys. Chem. B.

[582] Kulig W., Mikkolainen H., Olzyńska A., Jurkiewicz P., Cwiklik L., Hof M., Vattulainen I., Jungwirth P., Róg T., Olżyńska A. (2018). Bobbing of Oxysterols: Molecular Mechanism for Translocation of Tail-Oxidized Sterols through Biological Membranes. J. Phys. Chem. Lett..

[583] Stimson L.M., Dong L., Karttunen M., Wisniewska A., Dutka M., Róg T. (2007). Stearic acid spin labels in lipid bilayers: Insight through atomistic simulations. J. Phys. Chem. B.

[584] Kulig W., Olzyńska A., Jurkiewicz P., Kantola A.M., Komulainen S., Manna M., Pourmousa M., Vazdar M., Cwiklik L., Róg T. (2015). Cholesterol under oxidative stress—How lipid membranes sense oxidation as cholesterol is being replaced by oxysterols. Free Radic. Biol. Med..

[585] Yee S.M., Lorenz C.D. (2021). On the Structure and Flip-Flop of Free Docosahexaenoic Acid in a Model Human Brain Membrane. J. Phys. Chem. B.

[586] Roux B. (1995). The calculation of the potential of mean force using computer simulations. Comput. Phys. Commun..

[587] Neale C., Pomès R. (2016). Sampling errors in free energy simulations of small molecules in lipid bilayers. Biochim. Biophys. Acta-Biomembr..

[588] Dickson C.J., Hornak V., Bednarczyk D., Duca J.S. (2019). Using Membrane Partitioning Simulations to Predict Permeability of Forty-Nine Drug-Like Molecules. J. Chem. Inf. Model..

[589] Dickson C.J., Hornak V., Pearlstein R.A., Duca J.S. (2017). Structure–Kinetic Relationships of Passive Membrane Permeation from Multiscale Modeling. J. Am. Chem. Soc..

[590] Debnath M., Chakraborty S., Kumar Y.P., Chaudhuri R., Jana B., Dash J. (2020). Ionophore constructed from non-covalent assembly of a G-quadruplex and liponucleoside transports K+-ion across biological membranes. Nat. Commun..

[591] Kim G., Han S., Won H. (2015). Isolation of Microcystin-LR and Its Potential Function of Ionophore. J. Korean Magn. Reson. Soc..

[592] Marques I., Costa P.M.R., Miranda M.Q., Busschaert N., Howe E.N.W., Clarke H.J., Haynes C.J.E., Kirby I.L., Rodilla A.M., Pérez-Tomás R. (2018). Full elucidation of the transmembrane anion transport mechanism of squaramides using: In silico investigations. Phys. Chem. Chem. Phys..

[593] Spooner M.J., Li H., Marques I., Costa P.M.R., Wu X., Howe E.N.W., Busschaert N., Moore S.J., Light M.E., Sheppard D.N. (2019). Fluorinated synthetic anion carriers: Experimental and computational insights into transmembrane chloride transport. Chem. Sci..

[594] Pilato S., Aschi M., Bazzoni M., Bonati F.C., Cera G., Moffa S., Canale V., Ciulla M., Secchi A., Arduini A. (2021). Calixarene-based artificial ionophores for chloride transport across natural liposomal bilayer: Synthesis, structure-function relationships, and computational study. Biochim. Biophys. Acta-Biomembr..

[595] Janout V., Cline L.L., Feuston B.P., Klein L., O’Brien A., Tucker T., Yuan Y., O’Neill-Davis L.A., Peiffer R.L., Nerurkar S.S. (2014). Molecular umbrella conjugate for the ocular delivery of siRNA. Bioconjugate Chem..

[596] Janout V., Regen S.L. (2005). A needle-and-thread approach to bilayer transport: Permeation of a molecular umbrella-oligonucliotide conjugation across a phospholipid membrane. J. Am. Chem. Soc..

[597] Janout V., Regen S.L. (2009). Bioconjugate-based molecular umbrellas. Bioconjugate Chem..

[598] Chen A., Karanastasis A., Casey K.R., Necelis M., Carone B.R., Caputo G.A., Palermo E.F. (2020). Cationic Molecular Umbrellas as Antibacterial Agents with Remarkable Cell-Type Selectivity. ACS Appl. Mater. Interfaces.

[599] Danta C.C., Piplani P. (2020). Investigation of Molecular Properties of Antiretroviral Agents to Enhance CNS Penetration Abilities for the Treatment of Cognitive Impairment in HIV-Associated Neurocognitive Disorder. ACS Chem. Neurosci..

[600] Ashrafuzzaman M. (2021). The antimicrobial peptide gramicidin s enhances membrane adsorption and ion pore formation potency of chemotherapy drugs in lipid bilayers. Membranes.

[601] Pereira R., Silva S.G., Pinheiro M., Reis S., Luísa Do Vale M. (2021). Current status of amino acid-based permeation enhancers in transdermal drug delivery. Membranes.

[602] Park S.E., Sajid M.I., Parang K., Tiwari R.K. (2019). Cyclic cell-penetrating peptides as efficient intracellular drug delivery tools. Mol. Pharm..

[603] Bozdaganyan M.E., Orekhov P.S. (2021). Synergistic effect of chemical penetration enhancers on lidocaine permeability revealed by coarse-grained molecular dynamics simulations. Membranes.

[604] Gupta R., Dwadasi B.S., Rai B., Mitragotri S. (2019). Effect of Chemical Permeation Enhancers on Skin Permeability: In silico screening using Molecular Dynamics simulations. Sci. Rep..

[605] Marrink S.J., Risselada H.J., Yefimov S., Tieleman D.P., de Vries A.H. (2007). The MARTINI force field: Coarse grained model for biomolecular simulations. J. Phys. Chem. B.

[606] Monticelli L., Kandasamy S.K., Periole X., Larson R.G., Tieleman D.P., Marrink S.-J. (2008). The MARTINI Coarse-Grained Force Field: Extension to Proteins. J. Chem. Theory Comput..

[607] Róg T., Pasenkiewicz-Gierula M. (2006). Cholesterol-sphingomyelin interactions: A molecular dynamics simulation study. Biophys. J..

[608] Róg T., Pasenkiewicz-Gierula M., Vattulainen I., Karttunen M. (2009). Ordering effects of cholesterol and its analogues. Biochim. Biophys. Acta-Biomembr..

[609] Róg T., Vattulainen I. (2014). Cholesterol, sphingolipids, and glycolipids: What do we know about their role in raft-like membranes?. Chem. Phys. Lipids.

[610] Khalid S., Berglund N.A., Holdbrook D.A., Leung Y.M., Parkin J. (2015). The membranes of Gram-negative bacteria: Progress in molecular modelling and simulation. Biochem. Soc. Trans..

[611] Pavlova A., Hwang H., Lundquist K., Balusek C., Gumbart J.C. (2016). Living on the edge: Simulations of bacterial outer-membrane proteins. Biochim. Biophys. Acta-Biomembr..

[612] Shearer J., Marzinek J.K., Bond P.J., Khalid S. (2020). Molecular dynamics simulations of bacterial outer membrane lipid extraction: Adequate sampling?. J. Chem. Phys..

[613] Nickels J.D., Chatterjee S., Mostofian B., Stanley C.B., Ohl M., Zolnierczuk P., Schulz R., Myles D.A.A., Standaert R.F., Elkins J.G. (2017). Bacillus subtilis Lipid Extract, A Branched-Chain Fatty Acid Model Membrane. J. Phys. Chem. Lett..

[614] Gao Y., Lee J., Widmalm G., Im W. (2020). Modeling and Simulation of Bacterial Outer Membranes with Lipopolysaccharides and Enterobacterial Common Antigen. J. Phys. Chem. B.

[615] 6Pluhackova K., Horner A. (2021). Native-like membrane models of E. coli polar lipid extract shed light on the importance of lipid composition complexity. BMC Biol..

[616] Mostofian B., Zhuang T., Cheng X., Nickels J.D. (2019). Branched-Chain Fatty Acid Content Modulates Structure, Fluidity, and Phase in Model Microbial Cell Membranes. J. Phys. Chem. B.

[617] Gupta R., Rai B. (2015). Molecular Dynamics Simulation Study of Skin Lipids: Effects of the Molar Ratio of Individual Components over a Wide Temperature Range. J. Phys. Chem. B.

[618] Paloncýová M., Vávrová K., Sovová Ž., DeVane R., Otyepka M., Berka K. (2015). Structural Changes in Ceramide Bilayers Rationalize Increased Permeation through Stratum Corneum Models with Shorter Acyl Tails. J. Phys. Chem. B.

[619] Podewitz M., Wang Y., Gkeka P., Von Grafenstein S., Liedl K.R., Cournia Z. (2018). Phase Diagram of a Stratum Corneum Lipid Mixture. J. Phys. Chem. B.

[620] Badhe Y., Gupta R., Rai B. (2019). Structural and barrier properties of the skin ceramide lipid bilayer: A molecular dynamics simulation study. J. Mol. Model..

[621] Han S. (2019). Effect of Hydration on a Lipid Membrane Composed of Ceramide[NP]24, Lignoceric Acid, and Cholesterol: A Molecular Dynamics Simulation Study. Bull. Korean Chem. Soc..

[622] Wang E., Klauda J.B. (2019). Molecular Structure of the Long Periodicity Phase in the Stratum Corneum. J. Am. Chem. Soc..

[623] Wang E., Klauda J.B. (2019). Structure and Permeability of Ceramide Bilayers and Multilayers. J. Phys. Chem. B.

[624] Otto D.P., Combrinck J., Otto A., Tiedt L.R., De Villiers M.M. (2018). Dissipative particle dynamics investigation of the transport of salicylic acid through a simulated in vitro skin permeation model. Pharmaceuticals.

[625] Gorzelanny C., Mess C., Schneider S.W., Huck V., Brandner J.M. (2020). Skin barriers in dermal drug delivery: Which barriers have to be overcome and how can we measure them?. Pharmaceutics.

[626] Neupane R., Boddu S.H.S., Renukuntla J., Babu R.J., Tiwari A.K. (2020). Alternatives to biological skin in permeation studies: Current trends and possibilities. Pharmaceutics.

[627] Paananen R.O., Javanainen M., Holopainen J.M., Vattulainen I. (2019). Crystalline Wax Esters Regulate the Evaporation Resistance of Tear Film Lipid Layers Associated with Dry Eye Syndrome. J. Phys. Chem. Lett..

[628] Mainali L., Pasenkiewicz-Gierula M., Subczynski W.K. (2020). Formation of cholesterol Bilayer Domains Precedes Formation of Cholesterol Crystals in Membranes Made of the Major Phospholipids of Human Eye Lens Fiber Cell Plasma Membranes. Curr. Eye Res..

[629] Olżyńska A., Delcroix P., Dolejšová T., Krzaczek K., Korchowiec B., Czogalla A., Cwiklik L. (2020). Properties of Lipid Models of Lung Surfactant Containing Cholesterol and Oxidized Lipids: A Mixed Experimental and Computational Study. Langmuir.

[630] Paananen R.O., Viitaja T., Olżyńska A., Ekholm F.S., Moilanen J., Cwiklik L. (2020). Interactions of polar lipids with cholesteryl ester multilayers elucidate tear film lipid layer structure. Ocul. Surf..

[631] Cwiklik L. (2016). Tear film lipid layer: A molecular level view. Biochim. Biophys. Acta-Biomembr..

[632] Pai R.V., Monpara J.D., Vavia P.R. (2019). Exploring molecular dynamics simulation to predict binding with ocular mucin: An in silico approach for screening mucoadhesive materials for ocular retentive delivery systems. J. Control. Release.

[633] Liekkinen J., de Santos Moreno B., Paananen R.O., Vattulainen I., Monticelli L., Bernardino de la Serna J., Javanainen M. (2020). Understanding the Functional Properties of Lipid Heterogeneity in Pulmonary Surfactant Monolayers at the Atomistic Level. Front. Cell Dev. Biol..

[634] Liekkinen J., Enkavi G., Javanainen M., Olmeda B., Pérez-Gil J., Vattulainen I. (2020). Pulmonary Surfactant Lipid Reorganization Induced by the Adsorption of the Oligomeric Surfactant Protein B Complex. J. Mol. Biol..

[635] Sou T., Bergström C.A.S. (2021). Contemporary Formulation Development for Inhaled Pharmaceuticals. J. Pharm. Sci..

[636] Sou T., Kukavica-Ibrulj I., Levesque R.C., Friberg L.E., Bergström C.A.S. (2020). Model-Informed Drug Development in Pulmonary Delivery: Semimechanistic Pharmacokinetic-Pharmacodynamic Modeling for Evaluation of Treatments against Chronic Pseudomonas aeruginosa Lung Infections. Mol. Pharm..

[637] Sou T., Soukarieh F., Williams P., Stocks M.J., Cámara M., Bergström C.A.S. (2020). Model-Informed Drug Discovery and Development in Pulmonary Delivery: Biopharmaceutical Pharmacometric Modeling for Formulation Evaluation of Pulmonary Suspensions. ACS Omega.

[638] Sou T., Kukavica-Ibrulj I., Soukarieh F., Halliday N., Levesque R.C., Williams P., Stocks M., Cámara M., Friberg L.E., Bergström C.A.S. (2019). Model-Based Drug Development in Pulmonary Delivery: Pharmacokinetic Analysis of Novel Drug Candidates for Treatment of Pseudomonas aeruginosa Lung Infection. J. Pharm. Sci..

[639] Liu C., Elvati P., Violi A. (2021). On Drug-Membrane Permeability of Antivirals for SARS-CoV-2. J. Phys. Chem. Lett..

[640] Gordon D.E., Jang G.M., Bouhaddou M., Xu J., Obernier K., White K.M., O’Meara M.J., Rezelj V.V., Guo J.Z., Swaney D.L. (2020). A SARS-CoV-2 protein interaction map reveals targets for drug repurposing. Nature.

[641] Dahlgren D., Lennernäs H. (2019). Intestinal permeability and drug absorption: Predictive experimental, computational and in vivo approaches. Pharmaceutics.

[642] Hermann K.F., Neuhaus C.S., Micallef V., Wagner B., Hatibovic M., Aschmann H.E., Paech F., Alvarez-Sanchez R., Krämer S.D., Belli S. (2017). Kinetics of lipid bilayer permeation of a series of ionisable drugs and their correlation with human transporter-independent intestinal permeability. Eur. J. Pharm. Sci..

[643] Fagerberg J.H., Karlsson E., Ulander J., Hanisch G., Bergström C.A.S. (2015). Computational prediction of drug solubility in fasted simulated and aspirated human intestinal fluid. Pharm. Res..

[644] Bergstrom C.A.S., Parrow A., Larsson P., Augustijns P. (2020). Molecular dynamics simulations on interindividual variability of intestinal fluids: Impact on drug solubilization. Mol. Pharm..

[645] Lewitt P.A. (2015). Levodopa therapy for Parkinson’s disease: Pharmacokinetics and pharmacodynamics. Mov. Disord..

[646] Detrait E.R., Carr G.V., Weinberger D.R., Lamberty Y. (2016). Brain catechol-O-methyltransferase (COMT) inhibition by tolcapone counteracts recognition memory deficits in normal and chronic phencyclidine-treated rats and in COMT-Val transgenic mice. Behav. Pharmacol..

[647] Danta C.C. (2020). CNS Penetration Ability: A Critical Factor for Drugs in the Treatment of SARS-CoV-2 Brain Infection. ACS Chem. Neurosci..

[648] Verma K., Amitabh, Prasad D.N., Kumar B., Kohli E. (2020). Brain and COVID-19 Crosstalk: Pathophysiological and Psychological Manifestations. ACS Chem. Neurosci..

[649] Gao W., Liu Y., Jing G., Li K., Zhao Y., Sha B., Wang Q., Wu D. (2017). Rapid and efficient crossing blood-brain barrier: Hydrophobic drug delivery system based on propionylated amylose helix nanoclusters. Biomaterials.

[650] Rajagopal N., Irudayanathan F.J., Nangia S. (2019). Computational nanoscopy of tight junctions at the blood–brain barrier interface. Int. J. Mol. Sci..

[651] Nymeyer H., Zhou H.X. (2008). A method to determine dielectric constants in nonhomogeneous systems: Application to biological membranes. Biophys. J..

[652] Oroskar P., Jameson C.J., Murad S. (2017). Molecular dynamics simulations reveal how characteristics of surface and permeant affect permeation events at the surface of soft matter. Mol. Simul..

[653] Smith D.J., Leal L.-G., Mitragorti S., Shell M.S. (2018). Nanoparticle transport across model cellular membranes: When do solubility-diffusion models break down?. J. Phys. D Appl. Phys..

[654] Hoffmann C., Centi A., Menichetti R., Bereau T. (2020). Molecular dynamics trajectories for 630 coarse-grained drug-membrane permeations. Sci. Data.

[655] Menichetti R., Kanekal K.H., Bereau T. (2019). Drug-Membrane Permeability across Chemical Space. ACS Cent. Sci..

[656] Menichetti R., Kanekal K.H., Kremer K., Bereau T. (2017). In silico screening of drug-membrane thermodynamics reveals linear relations between bulk partitioning and the potential of mean force. J. Chem. Phys..

[657] Menichetti R., Bereau T. (2019). Revisiting the Meyer-Overton rule for drug-membrane permeabilities. Mol. Phys..

[658] Centi A., Dutta A., Parekh S.H., Bereau T. (2020). Inserting Small Molecules across Membrane Mixtures: Insight from the Potential of Mean Force. Biophys. J..

[659] Souza P.C.T., Alessandri R., Barnoud J., Faustino I., Grunewald F., Patmanidis I., Abdizadeh H., Bruininks B.M.H., Wassenaar T.A., Kroon P.C. (2021). Martini 3: A General Purpose Force Field for Coarse-Grained Molecular Dynamics. Nat. Methods.

[660] Alessandri R., Souza P.C.T., Thallmair S., Melo M.N., De Vries A.H., Marrink S.J. (2019). Pitfalls of the Martini Model. J. Chem. Theory Comput..

[661] Javanainen M., Martinez-Seara H., Vattulainen I. (2017). Excessive aggregation of membrane proteins in the Martini model. PLoS ONE.

[662] Jarin Z., Newhouse J., Voth G.A. (2021). Coarse-Grained Force Fields from the Perspective of Statistical Mechanics: Better Understanding of the Origins of a MARTINI Hangover. J. Chem. Theory Comput..

[663] Cornelius F., Habeck M., Kanai R., Toyoshima C., Karlish S.J.D. (2015). General and specific lipid-protein interactions in Na,K-ATPase. Biochim. Biophys. Acta-Biomembr..

[664] Brown M.F. (2017). Soft Matter in Lipid—Protein Interactions. Annu. Rev. Biophys..

[665] Gu R.X., de Groot B.L. (2020). Lipid-protein interactions modulate the conformational equilibrium of a potassium channel. Nat. Commun..

[666] Corradi V., Sejdiu B.I., Mesa-Galloso H., Abdizadeh H., Noskov S.Y., Marrink S.J., Tieleman D.P. (2019). Emerging Diversity in Lipid-Protein Interactions. Chem. Rev..

[667] 6Haghighi F., Yesylevskyy S., Davani S., Ramseyer C. (2021). Membrane environment modulates ligand-binding propensity of P2Y12 receptor. Pharmaceutics.

[668] Schmitt M.V., Lienau P., Fricker G., Reichel A. (2019). Quantitation of lysosomal trapping of basic lipophilic compounds using in vitro assays and in silico predictions based on the determination of the full pH profile of the endo-/lysosomal system in rat hepatocytes. Drug Metab. Dispos..

[669] Lu S., Sung T., Lin N., Abraham R.T., Jessen B.A. (2017). Lysosomal adaptation: How cells respond to lysosomotropic compounds. PLoS ONE.

[670] Villamil Giraldo A.M., Appelqvist H., Ederth T., Öllinger K. (2014). Lysosomotropic agents: Impact on lysosomal membrane permeabilization and cell death. Biochem. Soc. Trans..

[671] Varalda M., Antona A., Bettio V., Roy K., Vachamaram A., Yellenki V., Massarotti A., Baldanzi G., Capello D. (2020). Psychotropic Drugs Show Anticancer Activity by Disrupting Mitochondrial and Lysosomal Function. Front. Oncol..

[672] Petersen N.H.T., Olsen O.D., Groth-Pedersen L., Ellegaard A.M., Bilgin M., Redmer S., Ostenfeld M.S., Ulanet D., Dovmark T.H., Lønborg A. (2013). Transformation-Associated Changes in Sphingolipid Metabolism Sensitize Cells to Lysosomal Cell Death Induced by Inhibitors of Acid Sphingomyelinase. Cancer Cell.

[673] Javanainen M., Martinez-Seara H. (2016). Efficient preparation and analysis of membrane and membrane protein systems. Biochim. Biophys. Acta-Biomembr..

[674] Venable R.M., Brown F.L.H., Pastor R.W. (2015). Mechanical properties of lipid bilayers from molecular dynamics simulation. Chem. Phys. Lipids.

[675] Pan J., Heberle F.A., Tristram-Nagle S., Szymanski M., Koepfinger M., Katsaras J., Kučerka N. (2012). Molecular structures of fluid phase phosphatidylglycerol bilayers as determined by small angle neutron and X-ray scattering. Biochim. Biophys. Acta-Biomembr..

[676] Nagle J.F., Tristram-Nagle S. (2000). Structure of lipid bilayers. Biochim. Biophys. Acta-Rev. Biomembr..

[677] Kučerka N., Heberle F.A., Pan J., Katsaras J. (2015). Structural significance of lipid diversity as studied by small angle neutron and X-ray scattering. Membranes.

[678] Vermeer L.S., de Groot B.L., Réat V., Milon A., Czaplicki J. (2007). Acyl chain order parameter profiles in phospholipid bilayers: Computation from molecular dynamics simulations and comparison with 2H NMR experiments. Eur. Biophys. J..

[679] Lafleur M., Fine B., Sternin E., Cullis P.R., Bloom M. (1989). Smoothed orientational order profile of lipid bilayers by 2H-nuclear magnetic resonance. Biophys. J..

[680] Ollila S.O.H., Róg T., Karttunen M., Vattulainen I. (2007). Role of sterol type on lateral pressure profiles of lipid membranes affecting membrane protein functionality: Comparison between cholesterol, desmosterol, 7-dehydrocholesterol and ketosterol. J. Struct. Biol..

[681] Xie J.Y., Ding G.H., Karttunen M. (2014). Molecular dynamics simulations of lipid membranes with lateral force: Rupture and dynamic properties. Biochim. Biophys. Acta-Biomembr..

[682] Cantor R.S. (1997). Lateral Pressures in Cell Membranes: A Mechanism for Modulation of Protein Function. J. Phys. Chem. B.

[683] Cantor R.S. (1997). The lateral pressure profile in membranes: A physical mechanism of general anesthesia. Biochemistry.

[684] Cantor R.S. (1999). The influence of membrane lateral pressures on simple geometric models of protein conformational equilibria. Chem. Phys. Lipids.

[685] Lingwood D., Binnington B., Róg T., Vattulainen I., Grzybek M., Coskun Ü., Lingwood C.A., Simons K. (2011). Cholesterol modulates glycolipid conformation and receptor activity. Nat. Chem. Biol..

[686] Bilkova E., Pleskot R., Rissanen S., Sun S., Czogalla A., Cwiklik L., Róg T., Vattulainen I., Cremer P.S., Jungwirth P. (2017). Calcium Directly Regulates Phosphatidylinositol 4,5-Bisphosphate Headgroup Conformation and Recognition. J. Am. Chem. Soc..

[687] Pereira-Leite C., Nunes C., Reis S. (2013). Interaction of nonsteroidal anti-inflammatory drugs with membranes: In vitro assessment and relevance for their biological actions. Prog. Lipid Res..

[688] Lichtenberger L.M., Zhou Y., Dial E.J., Raphael R.M. (2006). NSAID injury to the gastrointestinal tract: Evidence that NSAIDs interact with phospholipids to weaken the hydrophobic surface barrier and induce the formation of unstable pores in membranes. J. Pharm. Pharmacol..

[689] Nunes C., Brezesinski G., Pereira-Leite C., Lima J.L.F.C., Reis S., Lúcio M. (2011). NSAIDs interactions with membranes: A biophysical approach. Langmuir.

[690] Pereira-Leite C., Figueiredo M., Burdach K., Nunes C., Reis S. (2021). Unraveling the role of drug-lipid interactions in nsaids-induced cardiotoxicity. Membranes.

[691] Overton E. (1901). Studien uber die Narkose Zugleich ein Beitrag zur Allgemeinen Pharmakologie.

[692] Meyer H. (1899). Zur theorie der alkoholnarkose. Naunyn. Schmiedebergs. Arch. Pharmacol..

[693] Janoff A.S., Pringle M.J., Miller K.W. (1981). Correlation of general anesthetic potency with solubility in membranes. Biochim. Biophys. Acta.

[694] Oakes V., Domene C. (2019). Capturing the Molecular Mechanism of Anesthetic Action by Simulation Methods. Chem. Rev..

[695] Reigada R. (2011). Influence of chloroform in liquid-ordered and liquid-disordered phases in lipid membranes. J. Phys. Chem. B.

[696] Turkyilmaz S., Chen W.H., Mitomo H., Regen S.L. (2009). Loosening and reorganization of fluid phospholipid bilayers by chloroform. J. Am. Chem. Soc..

[697] De Vlugt J.E., Xiao P., Munro R., Charchoglyan A., Brewer D., Al-Abdul-Wahid M.S., Brown L.S., Ladizhansky V. (2020). Identifying lipids tightly bound to an integral membrane protein. Biochim. Biophys. Acta-Biomembr..

[698] Sharma V., Belevich G., Gamiz-Hernandez A.P., Róg T., Vattulainen I., Verkhovskaya M.L., Wikström M., Hummer G., Kaila V.R.I. (2015). Redox-induced activation of the proton pump in the respiratory complex I. Proc. Natl. Acad. Sci. USA.

[699] Bruzzese A., Dalton J.A.R., Giraldo J. (2020). Insights into adenosine A2A receptor activation through cooperative modulation of agonist and allosteric lipid interactions. PLoS Comput. Biol..

[700] Takahashi H., Yoshino M., Morita K., Takagi T., Yokoyama Y., Kikukawa T., Amii H., Kanamori T., Sonoyama M. (2019). Stability of the two-dimensional lattice of bacteriorhodopsin reconstituted in partially fluorinated phosphatidylcholine bilayers. Biochim. Biophys. Acta-Biomembr..

[701] Mao X., Yao S., Yi Q., Xu Z.M., Cang X. (2021). Function-related asymmetry of the specific cardiolipin binding sites on the mitochondrial ADP/ATP carrier. Biochim. Biophys. Acta-Biomembr..

[702] Pöyry S., Cramariuc O., Postila P.A., Kaszuba K., Sarewicz M., Osyczka A., Vattulainen I., Róg T., Róg T. (2013). Atomistic simulations indicate cardiolipin to have an integral role in the structure of the cytochrome bc1 complex. Biochim. Biophys. Acta-Bioenerg..

[703] Škulj S., Brkljača Z., Vazdar M. (2020). Molecular Dynamics Simulations of the Elusive Matrix-Open State of Mitochondrial ADP/ATP Carrier. Isr. J. Chem..

[704] Manna M., Nieminen T., Vattulainen I. (2019). Understanding the Role of Lipids in Signaling Through Atomistic and Multiscale Simulations of Cell Membranes. Annu. Rev. Biophys..

[705] Jodaitis L., van Oene T., Martens C. (2021). Assessing the role of lipids in the molecular mechanism of membrane proteins. Int. J. Mol. Sci..

[706] Legler D.F., Matti C., Laufer J.M., Jakobs B.D., Purvanov V., Uetz-von Allmen E., Thelen M. (2017). Modulation of Chemokine Receptor Function by Cholesterol: New Prospects for Pharmacological Intervention. Mol. Pharmacol..

[707] McGraw C., Yang L., Levental I., Lyman E., Robinson A.S. (2019). Membrane cholesterol depletion reduces downstream signaling activity of the adenosine A 2A receptor. Biochim. Biophys. Acta-Biomembr..

[708] Manna M., Niemelä M., Tynkkynen J., Javanainen M., Kulig W., Müller D.J., Róg T., Vattulainen I. (2016). Mechanism of allosteric regulation of β 2 -adrenergic receptor by cholesterol. Elife.

[709] Delle Bovi R.J., Kim J.H., Suresh P., London E., Miller W.T. (2019). Sterol structure dependence of insulin receptor and insulin-like growth factor 1 receptor activation. Biochim. Biophys. Acta-Biomembr..

[710] Lemel L., Nieścierowicz K., García-Fernández M.D., Darré L., Durroux T., Busnelli M., Pezet M., Rébeillé F., Jouhet J., Mouillac B. (2021). The ligand-bound state of a G protein-coupled receptor stabilizes the interaction of functional cholesterol molecules. J. Lipid Res..

[711] Bovill J.G. (2000). Mechanisms of anaesthesia: Time to say farewell to the Meyer-Overton rule. Curr. Opin. Anaesthesiol..

[712] Nury H., Van Renterghem C., Weng Y., Tran A., Baaden M., Dufresne V., Changeux J.-P., Sonner J.M., Delarue M., Corringer P.-J. (2011). X-ray structures of general anesthetics bound to a pentameric ligand-gated ion channel. Nature.

[713] Pan J., Chen Q., Willenbring D., Mowrey D., Kong X.P., Cohen A., Divito C.B., Xu Y., Tang P. (2012). Structure of the pentameric ligand-gated ion channel GLIC bound with anesthetic ketamine. Structure.

[714] Gamal El-Din T.M., Lenaeus M.J., Zheng N., Catterall W.A. (2018). Fenestrations control resting-state block of a voltage-gated sodium channel. Proc. Natl. Acad. Sci. USA.

[715] Liu R., Perez-Aguilar J.M., Liang D., Saven J.G. (2012). Binding site and affinity prediction of general anesthetics to protein targets using docking. Anesth. Analg..

[716] Selkoe D.J., Hardy J. (2016). The amyloid hypothesis of Alzheimer’s disease at 25 years. EMBO Mol. Med..

[717] Scollo F., Rosa C. (2020). La Amyloidogenic intrinsically disordered proteins: New insights into their self-assembly and their interaction with membranes. Life.

[718] Errico S., Ramshini H., Capitini C., Canale C., Spaziano M., Barbut D., Calamai M., Zasloff M., Oropesa-Nuñez R., Vendruscolo M. (2021). Quantitative Measurement of the Affinity of Toxic and Nontoxic Misfolded Protein Oligomers for Lipid Bilayers and of its Modulation by Lipid Composition and Trodusquemine. ACS Chem. Neurosci..

[719] Banchelli M., Cascella R., D’Andrea C., La Penna G., Li M.S., Machetti F., Matteini P., Pizzanelli S. (2021). Probing the Structure of Toxic Amyloid-β Oligomers with Electron Spin Resonance and Molecular Modeling. ACS Chem. Neurosci..

[720] Manna M., Murarka R.K. (2021). Polyunsaturated fatty acid modulates membrane-bound monomeric α-synuclein by modulating membrane microenvironment through preferential interactions. ACS Chem. Neurosci..

[721] Khayat E., Lockhart C., Delfing B.M., Smith A.K., Klimov D.K. (2021). Met35 Oxidation Hinders Aβ25-35 Peptide Aggregation within the Dimyristoylphosphatidylcholine Bilayer. ACS Chem. Neurosci..

[722] Yang Y., Jalali S., Nilsson B.L., Dias C.L. (2021). Binding Mechanisms of Amyloid-like Peptides to Lipid Bilayers and Effects of Divalent Cations. ACS Chem. Neurosci..

[723] Khayat E., Klimov D.K., Smith A.K. (2020). Phosphorylation Promotes Aβ25-35 Peptide Aggregation within the DMPC Bilayer. ACS Chem. Neurosci..

[724] Banerjee S., Hashemi M., Zagorski K., Lyubchenko Y.L. (2021). Cholesterol in membranes facilitates aggregation of amyloid β protein at physiologically relevant concentrations. ACS Chem. Neurosci..

[725] Owen M.C., Kulig W., Poojari C., Róg T., Strodel B. (2018). Physiologically-relevant levels of sphingomyelin, but not GM1, induces a β-sheet-rich structure in the amyloid-β(1-42) monomer. Biochim. Biophys. Acta-Biomembr..

[726] Dias C.L., Jalali S., Yang Y., Cruz L. (2020). Role of Cholesterol on Binding of Amyloid Fibrils to Lipid Bilayers. J. Phys. Chem. B.

[727] Ngo S.T., Nguyen P.H., Derreumaux P. (2021). Cholesterol Molecules Alter the Energy Landscape of Small Aβ1-42 Oligomers. J. Phys. Chem. B.

[728] Ngo S.T., Nguyen P.H., Derreumaux P. (2021). Impact of the rat R5G, Y10F, and H13R mutations on tetrameric Aβ42 β-barrel in a lipid bilayer membrane model. J. Phys. Chem. B.

[729] Kawasaki T., Man V.H., Sugimoto Y., Sugiyama N., Yamamoto H., Tsukiyama K., Wang J., Derreumaux P., Nguyen P.H. (2020). Infrared Laser-Induced Amyloid Fibril Dissociation: A Joint Experimental/Theoretical Study on the GNNQQNY Peptide. J. Phys. Chem. B.

[730] Kurochka A.S., Yushchenko D.A., Bouř P., Shvadchak V.V. (2021). Influence of lipid membranes on α-synuclein aggregation. ACS Chem. Neurosci..

[731] Doig A.J., Derreumaux P. (2015). Inhibition of protein aggregation and amyloid formation by small molecules. Curr. Opin. Struct. Biol..

[732] Mrdenovic D., Zarzycki P., Majewska M., Pieta I.S., Nowakowski R., Kutner W., Lipkowski J., Pieta P. (2021). Inhibition of Amyloid β-Induced Lipid Membrane Permeation and Amyloid β Aggregation by K162. ACS Chem. Neurosci..

[733] Barrero-Sicilia C., Silvestre S., Haslam R.P., Michaelson L.V. (2017). Lipid remodelling: Unravelling the response to cold stress in Arabidopsis and its extremophile relative Eutrema salsugineum. Plant Sci..

[734] Hassan N., Anesio A.M., Rafiq M., Holtvoeth J., Bull I., Haleem A., Shah A.A., Hasan F. (2020). Temperature Driven Membrane Lipid Adaptation in Glacial Psychrophilic Bacteria. Front. Microbiol..

[735] Siliakus M.F., van der Oost J., Kengen S.W.M. (2017). Adaptations of archaeal and bacterial membranes to variations in temperature, pH and pressure. Extremophiles.

[736] Bejaoui F., Salas J.J., Nouairi I., Smaoui A., Abdelly C., Martínez-Force E., Youssef N. (2016). Ben Changes in chloroplast lipid contents and chloroplast ultrastructure in Sulla carnosa and Sulla coronaria leaves under salt stress. J. Plant Physiol..

[737] Kotnik T., Rems L., Tarek M., Miklavcic D. (2019). Membrane Electroporation and Electropermeabilization: Mechanisms and Models. Annu. Rev. Biophys..

[738] Koshiyama K., Kodama T., Yano T., Fujikawa S. (2008). Molecular dynamics simulation of structural changes of lipid bilayers induced by shock waves: Effects of incident angles. Biochim. Biophys. Acta-Biomembr..

[739] Man V.H., Li M.S., Wang J., Derreumaux P., Nguyen P.H. (2019). Interaction mechanism between the focused ultrasound and lipid membrane at the molecular level. J. Chem. Phys..

[740] Gurtovenko A.A., Vattulainen I. (2005). Pore formation coupled to ion transport through lipid membranes as induced by transmembrane ionic charge imbalance: Atomistic molecular dynamics study. J. Am. Chem. Soc..

[741] Lin J., Dargazany R., Alexander-Katz A. (2017). Lipid Flip-Flop and Pore Nucleation on Zwitterionic Bilayers are Asymmetric under Ionic Imbalance. Small.

[742] Lete M.G., Monasterio B.G., Collado M.I., Medina M., Sot J., Alonso A., Goñi F.M. (2019). Fast and slow biomembrane solubilizing detergents: Insights into their mechanism of action. Colloids Surf. B Biointerfaces.

[743] Lichtenberg D., Ahyayauch H., Alonso A., Goñi F.M. (2013). Detergent solubilization of lipid bilayers: A balance of driving forces. Trends Biochem. Sci..

[744] Dyrda G., Boniewska-Bernacka E., Man D., Barchiewicz K., Słota R. (2019). The effect of organic solvents on selected microorganisms and model liposome membrane. Mol. Biol. Rep..

[745] Clarke R.J., Hossain K.R., Cao K. (2020). Physiological roles of transverse lipid asymmetry of animal membranes. Biochim. Biophys. Acta-Biomembr..

[746] Chang W., Fa H., Xiao D., Wang J. (2020). Targeting phosphatidylserine for Cancer therapy: Prospects and challenges. Theranostics.

[747] Ma R., Kwok H.F. (2020). New opportunities and challenges of venom-based and bacteria-derived molecules for anticancer targeted therapy. Semin. Cancer Biol..

[748] Bernardes N., Fialho A.M. (2018). Perturbing the dynamics and organization of cell membrane components: A new paradigm for cancer-targeted therapies. Int. J. Mol. Sci..

[749] Nakatsuji T., Gallo R.L. (2012). Antimicrobial peptides: Old molecules with new ideas. J. Invest. Dermatol..

[750] Wang G., Li X., Wang Z. (2016). APD3: The antimicrobial peptide database as a tool for research and education. Nucleic Acids Res..

[751] Pirtskhalava M., Amstrong A.A., Grigolava M., Chubinidze M., Alimbarashvili E., Vishnepolsky B., Gabrielian A., Rosenthal A., Hurt D.E., Tartakovsky M. (2021). DBAASP v3: Database of antimicrobial/cytotoxic activity and structure of peptides as a resource for development of new therapeutics. Nucleic Acids Res..

[752] Jhong J.H., Chi Y.H., Li W.C., Lin T.H., Huang K.Y., Lee T.Y. (2019). DbAMP: An integrated resource for exploring antimicrobial peptides with functional activities and physicochemical properties on transcriptome and proteome data. Nucleic Acids Res..

[753] Waghu F.H., Barai R.S., Gurung P., Idicula-Thomas S. (2016). CAMPR3: A database on sequences, structures and signatures of antimicrobial peptides. Nucleic Acids Res..

[754] Zhao X., Wu H., Lu H., Li G., Huang Q. (2013). LAMP: A Database Linking Antimicrobial Peptides. PLoS ONE.

[755] Seebah S., Suresh A., Zhuo S., Choong Y.H., Chua H., Chuon D., Beuerman R., Verma C. (2007). Defensins knowledgebase: A manually curated database and information source focused on the defensins family of antimicrobial peptides. Nucleic Acids Res..

[756] Mulvenna J.P., Wang C., Craik D.J. (2006). CyBase: A database of cyclic protein sequence and structure. Nucleic Acids Res..

[757] Hammami R., Ben Hamida J., Vergoten G., Fliss I. (2009). PhytAMP: A database dedicated to antimicrobial plant peptides. Nucleic Acids Res..

[758] Das D., Jaiswal M., Khan F.N., Ahamad S., Kumar S. (2020). PlantPepDB: A manually curated plant peptide database. Sci. Rep..

[759] Liu S., Fan L., Sun J., Lao X., Zheng H. (2017). Computational resources and tools for antimicrobial peptides. J. Pept. Sci..

[760] Santos-Silva C.A.D., Zupin L., Oliveira-Lima M., Vilela L.M.B., Bezerra-Neto J.P., Ferreira-Neto J.R., Ferreira J.D.C., Oliveira-Silva R.L., de Pires C.J., Aburjaile F.F. (2020). Plant Antimicrobial Peptides: State of the Art, In Silico Prediction and Perspectives in the Omics Era. Bioinform. Biol. Insights.

[761] Kang X., Dong F., Shi C., Liu S., Sun J., Chen J., Li H., Xu H., Lao X., Zheng H. (2019). DRAMP 2.0, an updated data repository of antimicrobial peptides. Sci. Data.

[762] Chen C.H., Lu T.K. (2020). Development and challenges of antimicrobial peptides for therapeutic applications. Antibiotics.

[763] Cardoso P., Glossop H., Meikle T.G., Aburto-Medina A., Conn C.E., Sarojini V., Valery C. (2021). Molecular engineering of antimicrobial peptides: Microbial targets, peptide motifs and translation opportunities. Biophys. Rev..

[764] Lazzaro B.P., Zasloff M., Rolff J. (2020). Antimicrobial peptides: Application informed by evolution. Science.

[765] Roncevic T., Puizina J., Tossi A. (2019). Antimicrobial Peptides as Anti-Infective Agents in Pre-Post-Antibiotic Era?. Int. J. Mol. Sci..

[766] Guha S., Ghimire J., Wu E., Wimley W.C. (2019). Mechanistic Landscape of Membrane-Permeabilizing Peptides. Chem. Rev..

[767] Bhandari D., Rafiq S., Gat Y., Gat P., Waghmare R., Kumar V. (2020). A Review on Bioactive Peptides: Physiological Functions, Bioavailability and Safety. Int. J. Pept. Res. Ther..

[768] Huan Y., Kong Q., Mou H., Yi H. (2020). Antimicrobial Peptides: Classification, Design, Application and Research Progress in Multiple Fields. Front. Microbiol..

[769] Datta S., Roy A. (2021). Antimicrobial Peptides as Potential Therapeutic Agents: A Review. Int. J. Pept. Res. Ther..

[770] Gan B.H., Gaynord J., Rowe S.M., Deingruber T., Spring D.R. (2021). The multifaceted nature of antimicrobial peptides: Current synthetic chemistry approaches and future directions. Chem. Soc. Rev..

[771] Li W., Separovic F., O’Brien-Simpson N.M., Wade J.D. (2021). Chemically modified and conjugated antimicrobial peptides against superbugs. Chem. Soc. Rev..

[772] Boparai J.K., Sharma P.K. (2020). Mini Review on Antimicrobial Peptides, Sources, Mechanism and Recent Applications. Protein Pept. Lett..

[773] Wang J., Dou X., Song J., Lyu Y., Zhu X., Xu L., Li W., Shan A. (2019). Antimicrobial peptides: Promising alternatives in the post feeding antibiotic era. Med. Res. Rev..

[774] Pirtskhalava M., Vishnepolsky B., Grigolava M., Managadze G. (2021). Physicochemical features and peculiarities of interaction of amp with the membrane. Pharmaceuticals.

[775] Felício M.R., Silva O.N., Gonçalves S., Santos N.C., Franco O.L. (2017). Peptides with dual antimicrobial and anticancer activities. Front. Chem..

[776] Roque-borda C.A., da Silva P.B., Rodrigues M.C., Azevedo R.B., Di Filippo L., Duarte J.L., Chorilli M., Vicente E.F., Pavan F.R. (2021). Challenge in the discovery of new drugs: Antimicrobial peptides against who-list of critical and high-priority bacteria. Pharmaceutics.

[777] Gradisteanu Pircalabioru G., Popa L.I., Marutescu L., Gheorghe I., Popa M., Czobor Barbu I., Cristescu R., Chifiriuc M.C. (2021). Bacteriocins in the era of antibiotic resistance: Rising to the challenge. Pharmaceutics.

[778] Stiltner J., McCandless K., Zahid M. (2021). Cell-penetrating peptides: Applications in tumor diagnosis and therapeutics. Pharmaceutics.

[779] Gaspar D., Salomé Veiga A., Castanho M.A.R.B. (2013). From antimicrobial to anticancer peptides. A review. Front. Microbiol..

[780] Mahlapuu M., Håkansson J., Ringstad L., Björn C. (2016). Antimicrobial peptides: An emerging category of therapeutic agents. Front. Cell. Infect. Microbiol..

[781] Li J., Koh J.J., Liu S., Lakshminarayanan R., Verma C.S., Beuerman R.W. (2017). Membrane active antimicrobial peptides: Translating mechanistic insights to design. Front. Neurosci..

[782] Sani M.A., Separovic F. (2018). Antimicrobial Peptide Structures: From Model Membranes to Live Cells. Chem.—A Eur. J..

[783] Kang H.K., Kim C., Seo C.H., Park Y. (2017). The therapeutic applications of antimicrobial peptides (AMPs): A patent review. J. Microbiol..

[784] Mookherjee N., Anderson M.A., Haagsman H.P., Davidson D.J. (2020). Antimicrobial host defence peptides: Functions and clinical potential. Nat. Rev. Drug Discov..

[785] Hammond K., Ryadnov M.G., Hoogenboom B.W. (2021). Atomic force microscopy to elucidate how peptides disrupt membranes. Biochim. Biophys. Acta-Biomembr..

[786] Aschi M., Bozzi A., Luzi C., Bouchemal N., Sette M. (2017). Crabrolin, a natural antimicrobial peptide: Structural properties. J. Pept. Sci..

[787] Mura M., Wang J., Zhou Y., Pinna M., Zvelindovsky A.V., Dennison S.R., Phoenix D.A. (2016). The effect of amidation on the behaviour of antimicrobial peptides. Eur. Biophys. J..

[788] Ulmschneider J.P., Smith J.C., Ulmschneider M.B., Ulrich A.S., Strandberg E. (2012). Reorientation and dimerization of the membrane-bound antimicrobial peptide pgla from microsecond all-atom MD simulations. Biophys. J..

[789] Ramos-Martín F., D’Amelio N. (2021). Molecular basis of the anticancer and antibacterial properties of cecropinXJ peptide: An in silico study. Int. J. Mol. Sci..

[790] Pourmousa M., Karttunen M. (2013). Early stages of interactions of cell-penetrating peptide penetratin with a DPPC bilayer. Chem. Phys. Lipids.

[791] Mura M., Dennison S.R., Zvelindovsky A.V., Phoenix D.A. (2013). Aurein 2.3 functionality is supported by oblique orientated α-helical formation. Biochim. Biophys. Acta-Biomembr..

[792] Sahoo B.R., Fujiwara T. (2016). Membrane mediated antimicrobial and antitumor activity of cathelicidin 6: Structural insights from molecular dynamics simulation on multi-microsecond scale. PLoS ONE.

[793] Zhao L., Cao Z., Bian Y., Hu G., Wang J., Zhou Y. (2018). Molecular dynamics simulations of human antimicrobial peptide LL-37 in model POPC and POPG lipid bilayers. Int. J. Mol. Sci..

[794] Zhang L. (2020). Disulfide Bonds Affect the Binding Sites of Human β Defensin Type 3 on Negatively Charged Lipid Membranes. J. Phys. Chem. B.

[795] Pandit G., Biswas K., Ghosh S., Debnath S., Bidkar A.P., Satpati P., Bhunia A., Chatterjee S. (2020). Rationally designed antimicrobial peptides: Insight into the mechanism of eleven residue peptides against microbial infections. Biochim. Biophys. Acta-Biomembr..

[796] Reißer S., Strandberg E., Steinbrecher T., Elstner M., Ulrich A.S. (2018). Best of Two Worlds? How MD Simulations of Amphiphilic Helical Peptides in Membranes Can Complement Data from Oriented Solid-State NMR. J. Chem. Theory Comput..

[797] Ermondi G., Vallaro M., Camacho-Leal M.P., Potter T., Visentin S., Caron G. (2018). Charged cyclic hexapeptides: Updating molecular descriptors for permeability purposes. Eur. J. Pharm. Sci..

[798] Aschi M., Perini N., Bouchemal N., Luzi C., Savarin P., Migliore L., Bozzi A., Sette M. (2020). Structural characterization and biological activity of Crabrolin peptide isoforms with different positive charge. Biochim. Biophys. Acta-Biomembr..

[799] Duay S.S., Sharma G., Prabhakar R., Angeles-Boza A.M., May E.R. (2019). Molecular Dynamics Investigation into the Effect of Zinc(II) on the Structure and Membrane Interactions of the Antimicrobial Peptide Clavanin A. J. Phys. Chem. B.

[800] Jafari M., Mehrnejad F., Aghdami R., Chaparzadeh N., Razaghi Moghadam Kashani Z., Doustdar F. (2017). Identification of the Crucial Residues in the Early Insertion of Pardaxin into Different Phospholipid Bilayers. J. Chem. Inf. Model..

[801] Song C., de Groot B.L., Sansom M.S.P. (2019). Lipid Bilayer Composition Influences the Activity of the Antimicrobial Peptide Dermcidin Channel. Biophys. J..

[802] Fernandez J., Acosta G., Pulido D., Malý M., Copa-Patiño J.L., Soliveri J., Royo M., Gómez R., Albericio F., Ortega P. (2019). Carbosilane Dendron-Peptide Nanoconjugates as Antimicrobial Agents. Mol. Pharm..

[803] Grasso G., Muscat S., Rebella M., Morbiducci U., Audenino A., Danani A., Deriu M.A. (2018). Cell penetrating peptide modulation of membrane biomechanics by Molecular dynamics. J. Biomech..

[804] Irudayam S.J., Berkowitz M.L. (2012). Binding and reorientation of melittin in a POPC bilayer: Computer simulations. Biochim. Biophys. Acta-Biomembr..

[805] Lee H., Kim H.R., Larson R.G., Park J.C. (2012). Effects of the size, shape, and structural transition of thermosensitive polypeptides on the stability of lipid bilayers and liposomes. Macromolecules.

[806] Jafari M., Mehrnejad F., Doustdar F. (2017). Insight into the interactions, residue snorkeling, and membrane disordering potency of a single antimicrobial peptide into different lipid bilayers. PLoS ONE.

[807] Liu J., Xiao S., Li J., Yuan B., Yang K., Ma Y. (2018). Molecular details on the intermediate states of melittin action on a cell membrane. Biochim. Biophys. Acta-Biomembr..

[808] Waghu F.H., Joseph S., Ghawali S., Martis E.A., Madan T., Venkatesh K.V., Idicula-Thomas S. (2018). Designing antibacterial peptides with enhanced killing kinetics. Front. Microbiol..

[809] Ashrafuzzaman M., Tseng C.Y., Tuszynski J.A. (2020). Charge-based interactions of antimicrobial peptides and general drugs with lipid bilayers. J. Mol. Graph. Model..

[810] Cao Z., Liu L., Hu G., Bian Y., Li H., Wang J., Zhou Y. (2020). Interplay of hydrophobic and hydrophilic interactions in sequence-dependent cell penetration of spontaneous membrane-translocating peptides revealed by bias-exchange metadynamics simulations. Biochim. Biophys. Acta-Biomembr..

[811] Walrant A., Bauzá A., Girardet C., Alves I.D., Lecomte S., Illien F., Cardon S., Chaianantakul N., Pallerla M., Burlina F. (2020). Ionpair-π interactions favor cell penetration of arginine/tryptophan-rich cell-penetrating peptides. Biochim. Biophys. Acta-Biomembr..

[812] Capozzi E., Aureli S., Minicozzi V., Rossi G.C., Stellato F., Morante S. (2018). Designing effective anticancer-radiopeptides. A Molecular Dynamics study of their interaction with model tumor and healthy cell membranes. Biochim. Biophys. Acta-Biomembr..

[813] Ma R., Wong S.W., Ge L., Shaw C., Siu S.W.I., Kwok H.F. (2020). In Vitro and MD Simulation Study to Explore Physicochemical Parameters for Antibacterial Peptide to Become Potent Anticancer Peptide. Mol. Ther.-Oncolytics.

[814] Song J., Zhang W., Kai M., Chen J., Liang R., Zheng X., Li G., Zhang B., Wang K., Zhang Y. (2013). Design of an acid-activated antimicrobial peptide for tumor therapy. Mol. Pharm..

[815] Sinha S., Zheng L., Mu Y., Ng W.J., Bhattacharjya S. (2017). Structure and interactions of a host defense antimicrobial peptide thanatin in lipopolysaccharide micelles reveal mechanism of bacterial cell agglutination. Sci. Rep..

[816] Brožek R., Kabelka I., Vácha R. (2020). Effect of Helical Kink on Peptide Translocation across Phospholipid Membranes. J. Phys. Chem. B.

[817] Tuerkova A., Kabelka I., Králová T., Sukeník L., Pokorná Š., Hof M., Vácha R. (2020). Effect of Helical Kink in Antimicrobial Peptides on Membrane Pore Formation. Elife.

[818] Mihajlovic M., Lazaridis T. (2010). Antimicrobial peptides in toroidal and cylindrical pores. Biochim. Biophys. Acta-Biomembr..

[819] Mihajlovic M., Lazaridis T. (2012). Charge distribution and imperfect amphipathicity affect pore formation by antimicrobial peptides. Biochim. Biophys. Acta-Biomembr..

[820] Lyu Y., Zhu X., Xiang N., Narsimhan G. (2015). Molecular Dynamics Study of Pore Formation by Melittin in a 1,2-Dioleoyl-sn-glycero-3-phosphocholine and 1,2-Di(9Z-octadecenoyl)-sn-glycero-3-phospho-(1′-rac-glycerol) Mixed Lipid Bilayer. Ind. Eng. Chem. Res..

[821] Sun D., Forsman J., Woodward C.E. (2015). Amphipathic membrane-active peptides recognize and stabilize ruptured membrane pores: Exploring cause and effect with coarse-grained simulations. Langmuir.

[822] Santo K.P., Berkowitz M.L. (2012). Difference between magainin-2 and melittin assemblies in phosphatidylcholine bilayers: Results from coarse-grained simulations. J. Phys. Chem. B.

[823] Hu Y., Sinha S.K., Patel S. (2015). Investigating Hydrophilic Pores in Model Lipid Bilayers Using Molecular Simulations: Correlating Bilayer Properties with Pore-Formation Thermodynamics. Langmuir.

[824] Santo K.P., Irudayam S.J., Berkowitz M.L. (2013). Melittin creates transient pores in a lipid bilayer: Results from computer simulations. J. Phys. Chem. B.

[825] Li J., Lu X., Ma W., Chen Z., Sun S., Wang Q., Yuan B., Yang K. (2021). Cholesterols Work as a Molecular Regulator of the Antimicrobial Peptide-Membrane Interactions. Front. Mol. Biosci..

[826] Sun D., Forsman J., Woodward C.E. (2015). Multistep Molecular Dynamics Simulations Identify the Highly Cooperative Activity of Melittin in Recognizing and Stabilizing Membrane Pores. Langmuir.

[827] Sun D., Forsman J., Woodward C.E. (2017). Molecular Simulations of Melittin-Induced Membrane Pores. J. Phys. Chem. B.

[828] Wang Y., Chen C.H., Hu D., Ulmschneider M.B., Ulmschneider J.P. (2016). Spontaneous formation of structurally diverse membrane channel architectures from a single antimicrobial peptide. Nat. Commun..

[829] Talandashti R., Mehrnejad F., Rostamipour K., Doustdar F., Lavasanifar A. (2021). Molecular Insights into Pore Formation Mechanism, Membrane Perturbation, and Water Permeation by the Antimicrobial Peptide Pleurocidin: A Combined All-Atom and Coarse-Grained Molecular Dynamics Simulation Study. J. Phys. Chem. B.

[830] Chen C.H., Starr C.G., Troendle E., Wiedman G., Wimley W.C., Ulmschneider J.P., Ulmschneider M.B. (2019). Simulation-Guided Rational de Novo Design of a Small Pore-Forming Antimicrobial Peptide. J. Am. Chem. Soc..

[831] Lorenzón E.N., Nobre T.M., Caseli L., Cilli E.M., da Hora G.C.A., Soares T.A., Oliveira O.N. (2018). The “pre-assembled state” of magainin 2 lysine-linked dimer determines its enhanced antimicrobial activity. Colloids Surf. B Biointerfaces.

[832] Miyazaki Y., Okazaki S., Shinoda W. (2019). Free energy analysis of membrane pore formation process in the presence of multiple melittin peptides. Biochim. Biophys. Acta-Biomembr..

[833] Lyu Y., Xiang N., Zhu X., Narsimhan G. (2017). Potential of mean force for insertion of antimicrobial peptide melittin into a pore in mixed DOPC/DOPG lipid bilayer by molecular dynamics simulation. J. Chem. Phys..

[834] Awasthi N., Kopec W., Wilkosz N., Jamróz D., Hub J.S., Zatorska M., Petka R., Nowakowska M., Kepczynski M. (2019). Molecular Mechanism of Polycation-Induced Pore Formation in Biomembranes. ACS Biomater. Sci. Eng..

[835] Bennett W.F.D., Hong C.K., Wang Y., Tieleman D.P. (2016). Antimicrobial Peptide Simulations and the Influence of Force Field on the Free Energy for Pore Formation in Lipid Bilayers. J. Chem. Theory Comput..

[836] Yesylevskyy S., Marrink S., Mark A.E. (2009). Alternative Mechanisms for the Interaction of the Cell-Penetrating Peptides Penetratin and the TAT Peptide with Lipid Bilayers. Biophysj.

[837] Simon R.J., Kania R.S., Zuckermann R.N., Huebner V.D., Jewell D.A., Banville S., Ng S., Wang L., Rosenberg S., Marlowe C.K. (1992). Peptoids: A modular approach to drug discovery. Proc. Natl. Acad. Sci. USA.

[838] Jin H., Jiao F., Daily M.D., Chen Y., Yan F., Ding Y.H., Zhang X., Robertson E.J., Baer M.D., Chen C.-L. (2016). Highly stable and self-repairing membrane-mimetic 2D nanomaterials assembled from lipid-like peptoids. Nat. Commun..

[839] Zhao M., Sampath J., Alamdari S., Shen G., Chen C.L., Mundy C.J., Pfaendtner J., Ferguson A.L. (2020). MARTINI-Compatible Coarse-Grained Model for the Mesoscale Simulation of Peptoids. J. Phys. Chem. B.

[840] Landry M.R., Rangel J.L., Dao V.P., Mackenzie M.A., Gutierrez F.L., Dowell K.M., Calkins A.L., Fuller A.A., Stokes G.Y. (2019). Length and Charge of Water-Soluble Peptoids Impact Binding to Phospholipid Membranes. J. Phys. Chem. B.

[841] Andreev K., Martynowycz M.W., Ivankin A., Huang M.L., Kuzmenko I., Meron M., Lin B., Kirshenbaum K., Gidalevitz D. (2016). Cyclization Improves Membrane Permeation by Antimicrobial Peptoids. Langmuir.

[842] Diamond G., Molchanova N., Herlan C., Fortkort J.A., Lin J.S., Figgins E., Bopp N., Ryan L.K., Chung D., Adcock R.S. (2021). Potent antiviral activity against HSV-1 and SARS-CoV-2 by antimicrobial peptoids. Pharmaceuticals.

[843] Koivuniemi A., Fallarero A., Bunker A. (2019). Insight into the antimicrobial mechanism of action of β2,2-amino acid derivatives from molecular dynamics simulation: Dancing the can-can at the membrane surface. Biochim. Biophys. Acta-Biomembr..

[844] Sessa L., Concilio S., Walde P., Robinson T., Dittrich P.S., Porta A., Panunzi B., Caruso U., Piotto S. (2020). Study of the interaction of a novel semi-synthetic peptide with model lipid membranes. Membranes.

[845] Nourbakhsh S., Yu L., Ha B.Y. (2021). Modeling the Protective Role of Bacterial Lipopolysaccharides against Membrane-Rupturing Peptides. J. Phys. Chem. B.

[846] Mitra M., Asad M., Kumar S., Yadav K., Chaudhary S., Bhavesh N.S., Khalid S., Thukral L., Bajaj A. (2019). Distinct intramolecular hydrogen bonding dictates antimicrobial action of membrane-targeting amphiphiles. J. Phys. Chem. Lett..

[847] Dalhoff A.A. (2018). Membrane Interactions of Antibacterial Agents. Trends Clin. Microbiol..

[848] Khondker A., Bider R.C., Passos-Gastaldo I., Wright G.D., Rheinstädter M.C. (2021). Membrane interactions of non-membrane targeting antibiotics: The case of aminoglycosides, macrolides, and fluoroquinolones. Biochim. Biophys. Acta-Biomembr..

[849] Ashrafuzzaman M., Khan Z., Alqarni A., Alanazi M., Alam M.S. (2021). Cell surface binding and lipid interactions behind chemotherapy-drug-induced ion pore formation in membranes. Membranes.

[850] Annaval T., Ramos-Martin F., Herrera-Leon C., Adelaide M., Antonietti V., Buchoux S., Sonnet P., Sarazin C., D’Amelio N. (2021). Antimicrobial Bombinin-like Peptide 3 Selectively Recognizes and Inserts into Bacterial Biomimetic Bilayers in Multiple Steps. J. Med. Chem..

[851] Petkov P., Lilkova E., Ilieva N., Litov L. (2019). Self-association of antimicrobial peptides: A molecular dynamics simulation study on bombinin. Int. J. Mol. Sci..

[852] Savini F., Loffredo M.R., Troiano C., Bobone S., Malanovic N., Eichmann T.O., Caprio L., Canale V.C., Park Y., Mangoni M.L. (2020). Binding of an antimicrobial peptide to bacterial cells: Interaction with different species, strains and cellular components. Biochim. Biophys. Acta-Biomembr..

[853] Huang H.W. (2020). DAPTOMYCIN, its membrane-active mechanism vs. that of other antimicrobial peptides. Biochim. Biophys. Acta-Biomembr..

[854] Mescola A., Ragazzini G., Alessandrini A. (2020). Daptomycin Strongly Affects the Phase Behavior of Model Lipid Bilayers. J. Phys. Chem. B.

[855] Liu B., Karttunen M. (2018). Lipopeptide daptomycin: Interactions with bacterial and phospholipid membranes, stability of membrane aggregates and micellation in solution. Biochim. Biophys. Acta-Biomembr..

[856] Ramos-Martín F., Herrera-León C., Antonietti V., Sonnet P., Sarazin C., D’amelio N. (2021). Antimicrobial peptide k11 selectively recognizes bacterial biomimetic membranes and acts by twisting their bilayers. Pharmaceuticals.

[857] Grishin S.Y., Domnin P.A., Kravchenko S.V., Azev V.N., Mustaeva L.G., Gorbunova E.Y., Kobyakova M.I., Surin A.K., Makarova M.A., Kurpe S.R. (2021). Is It Possible to Create Antimicrobial Peptides Based on the Amyloidogenic Sequence of Ribosomal S1 Protein of P. aeruginosa?. Int. J. Mol. Sci..

[858] Shi J., Chen C., Wang D., Tong Z., Wang Z., Liu Y. (2021). Amphipathic peptide antibiotics with potent activity against multidrug-resistant pathogens. Pharmaceutics.

[859] Aguiar L., Pinheiro M., Neves A.R., Vale N., Defaus S., Andreu D., Reis S., Gomes P. (2021). Insights into the membranolytic activity of antimalarial drug-cell penetrating peptide conjugates. Membranes.

[860] Ohgita T., Takechi-Haraya Y., Okada K., Matsui S., Takeuchi M., Saito C., Nishitsuji K., Uchimura K., Kawano R., Hasegawa K. (2020). Enhancement of direct membrane penetration of arginine-rich peptides by polyproline II helix structure. Biochim. Biophys. Acta-Biomembr..

[861] Strandberg E., Bentz D., Wadhwani P., Bürck J., Ulrich A.S. (2020). Terminal charges modulate the pore forming activity of cationic amphipathic helices. Biochim. Biophys. Acta-Biomembr..

[862] Wu E., Jenschke R.M., Hristova K., Wimley W.C. (2020). Rational Modulation of pH-Triggered Macromolecular Poration by Peptide Acylation and Dimerization. J. Phys. Chem. B.

[863] Bansal S., Su W.C., Budamagunta M., Xiao W., Ajena Y., Liu R., Voss J.C., Carney R.P., Parikh A.N., Lam K.S. (2020). Discovery and mechanistic characterization of a structurally-unique membrane active peptide. Biochim. Biophys. Acta-Biomembr..

[864] Lima B., Ricci M., Garro A., Juhász T., Szigyártó I.C., Papp Z.I., Feresin G., Garcia de la Torre J., Lopez Cascales J., Fülöp L. (2021). New short cationic antibacterial peptides. Synthesis, biological activity and mechanism of action. Biochim. Biophys. Acta-Biomembr..

[865] Das P., Sercu T., Wadhawan K., Padhi I., Gehrmann S., Cipcigan F., Chenthamarakshan V., Strobelt H., dos Santos C., Chen P.Y. (2021). Accelerated antimicrobial discovery via deep generative models and molecular dynamics simulations. Nat. Biomed. Eng..

[866] Chakraborty A., Kobzev E., Chan J., De Zoysa G.H., Sarojini V., Piggot T.J., Allison J.R. (2021). Molecular Dynamics Simulation of the Interaction of Two Linear Battacin Analogs with Model Gram-Positive and Gram-Negative Bacterial Cell Membranes. ACS Omega.

[867] Malik E., Phoenix D.A., Badiani K., Snape T.J., Harris F., Singh J., Morton L.H.G., Dennison S.R. (2020). Biophysical studies on the antimicrobial activity of linearized esculentin 2EM. Biochim. Biophys. Acta-Biomembr..

[868] Abel S., Marchi M. (2021). Deciphering the structure of the gramicidin A channel in the presence of AOT reverse micelles in pentane using molecular dynamics simulations. J. Phys. Chem. B.

[869] Nawae W., Hannongbua S., Ruengjitchatchawalya M. (2014). Defining the membrane disruption mechanism of kalata B1 via coarse-grained molecular dynamics simulations. Sci. Rep..

[870] Rashid M.M.O., Moghal M.M.R., Billah M.M., Hasan M., Yamazaki M. (2020). Effect of membrane potential on pore formation by the antimicrobial peptide magainin 2 in lipid bilayers. Biochim. Biophys. Acta-Biomembr..

[871] Amos S.B.T.A., Vermeer L.S., Ferguson P.M., Kozlowska J., Davy M., Bui T.T., Drake A.F., Lorenz C.D., Mason A.J. (2016). Antimicrobial Peptide Potency is Facilitated by Greater Conformational Flexibility when Binding to Gram-negative Bacterial Inner Membranes. Sci. Rep..

[872] Hong J., Lu X., Deng Z., Xiao S., Yuan B., Yang K. (2019). How Melittin Inserts into Cell Membrane: And Disturbance on the Membrane. Molecules.

[873] Irudayam S.J., Pobandt T., Berkowitz M.L. (2013). Free energy barrier for melittin reorientation from a membrane-bound state to a transmembrane state. J. Phys. Chem. B.

[874] Manna M., Mukhopadhyay C. (2009). Cause and effect of melittin-induced pore formation: A computational approach. Langmuir.

[875] Leveritt J.M., Pino-Angeles A., Lazaridis T. (2015). The structure of a melittin-stabilized pore. Biophys. J..

[876] Pal S., Chakraborty H., Chattopadhyay A. (2021). Lipid Headgroup Charge Controls Melittin Oligomerization in Membranes: Implications in Membrane Lysis. J. Phys. Chem. B.

[877] Koch D.C., Schmidt T.H., Sahl H.G., Kubitscheck U., Kandt C. (2014). Structural dynamics of the cell wall precursor lipid II in the presence and absence of the lantibiotic nisin. Biochim. Biophys. Acta-Biomembr..

[878] Jefferies D., Hsu P.C., Khalid S. (2017). Through the Lipopolysaccharide Glass: A Potent Antimicrobial Peptide Induces Phase Changes in Membranes. Biochemistry.

[879] Afanasyeva E.F., Syryamina V.N., De Zotti M., Formaggio F., Toniolo C., Dzuba S.A. (2019). Peptide antibiotic trichogin in model membranes: Self-association and capture of fatty acids. Biochim. Biophys. Acta-Biomembr..

[880] Arinaminpathy Y., Khurana E., Engelman D.M., Gerstein M.B. (2009). Computational analysis of membrane proteins: The largest class of drug targets. Drug Discov. Today.

[881] Yin H., Flynn A.D. (2016). Drugging Membrane Protein Interactions. Annu. Rev. Biomed. Eng..

[882] Alballa M., Butler G. (2020). Integrative approach for detecting membrane proteins. BMC Bioinform..

[883] Dobson L.L., Remenyi I., Tusnady G.E., Reményi I., Tusnády G.E. (2015). The human transmembrane proteome. Biol. Direct.

[884] Garrow A.G., Agnes A., Westhead D.R., Agnew A., Westhead D.R. (2005). TMB-Hunt: An amino acid composition based method to screen proteomes for beta-barrel transmembrane proteins. BMC Bioinform..

[885] Potterton A., Husseini F.S., Southey M.W.Y., Bodkin M.J., Heifetz A., Coveney P.V., Townsend-Nicholson A. (2019). Ensemble-Based Steered Molecular Dynamics Predicts Relative Residence Time of A 2A Receptor Binders. J. Chem. Theory Comput..

[886] Zhang X., Sun H., Wen X., Yuan H. (2019). A Selectivity Study of FFAR4/FFAR1 Agonists by Molecular Modeling. J. Chem. Inf. Model..

[887] Perez-Aguilar J.M., Kang S.G., Zhang L., Zhou R. (2019). Modeling and Structural Characterization of the Sweet Taste Receptor Heterodimer. ACS Chem. Neurosci..

[888] Bellucci L., Felline A., Fanelli F. (2020). Dynamics and structural communication in the ternary complex of fully phosphorylated V2 vasopressin receptor, vasopressin, and β-arrestin 1. Biochim. Biophys. Acta-Biomembr..

[889] Azhagiya Singam E.R., Tachachartvanich P., La Merrill M.A., Smith M.T., Durkin K.A. (2019). Structural Dynamics of Agonist and Antagonist Binding to the Androgen Receptor. J. Phys. Chem. B.

[890] An X., Bai Q., Bing Z., Liu H., Zhang Q., Liu H., Yao X. (2020). Revealing the Positive Binding Cooperativity Mechanism between the Orthosteric and the Allosteric Antagonists of CCR2 by Metadynamics and Gaussian Accelerated Molecular Dynamics Simulations. ACS Chem. Neurosci..

[891] Lei T., Hu Z., Ding R., Chen J., Li S., Zhang F., Pu X., Zhao N. (2020). Exploring the Activation Mechanism of a Metabotropic Glutamate Receptor Homodimer via Molecular Dynamics Simulation. ACS Chem. Neurosci..

[892] Yang J.F., Williams A.H., Penthala N.R., Prather P.L., Crooks P.A., Zhan C.G. (2020). Binding Modes and Selectivity of Cannabinoid 1 (CB1) and Cannabinoid 2 (CB2) Receptor Ligands. ACS Chem. Neurosci..

[893] Reis M.H., Antunes D., Santos L.H.S., Guimarães A.C.R., Caffarena E.R. (2020). Shared Binding Mode of Perrottetinene and Tetrahydrocannabinol Diastereomers inside the CB1 Receptor May Incentivize Novel Medicinal Drug Design: Findings from an in Silico Assay. ACS Chem. Neurosci..

[894] Ji B., Liu S., He X., Man V.H., Xie X.Q., Wang J. (2020). Prediction of the Binding Affinities and Selectivity for CB1 and CB2 Ligands Using Homology Modeling, Molecular Docking, Molecular Dynamics Simulations, and MM-PBSA Binding Free Energy Calculations. ACS Chem. Neurosci..

[895] Poudel H., Leitner D.M. (2021). Activation-Induced Reorganization of Energy Transport Networks in the β 2 Adrenergic Receptor. J. Phys. Chem. B.

[896] Shang Y., Yeatman H.R., Provasi D., Alt A., Christopoulos A., Canals M., Filizola M. (2016). Proposed Mode of Binding and Action of Positive Allosteric Modulators at Opioid Receptors. ACS Chem. Biol..

[897] Bortolato A., Deflorian F., Weiss D.R., Mason J.S. (2015). Decoding the Role of Water Dynamics in Ligand-Protein Unbinding: CRF1R as a Test Case. J. Chem. Inf. Model..

[898] Provasi D., Bortolato A., Filizola M. (2009). Exploring molecular mechanisms of ligand recognition by opioid receptors with metadynamics. Biochemistry.

[899] Kaszuba K., Róg T., Bryl K., Vattulainen I., Karttunen M. (2010). Molecular dynamics simulations reveal fundamental role of water as factor determining affinity of binding of β-blocker nebivolol to β-adrenergic receptor. J. Phys. Chem. B.

[900] Ribeiro J.M.L., Filizola M. (2019). Insights From Molecular Dynamics Simulations of a Number of G-Protein Coupled Receptor Targets for the Treatment of Pain and Opioid Use Disorders. Front. Mol. Neurosci..

[901] Yang D., Zhou Q., Labroska V., Qin S., Darbalaei S., Wu Y., Yuliantie E., Xie L., Tao H., Cheng J. (2021). G protein-coupled receptors: Structure- and function-based drug discovery. Signal Transduct. Target. Ther..

[902] Tautermann C.S., Seeliger D., Kriegl J.M. (2015). What can we learn from molecular dynamics simulations for GPCR drug design?. Comput. Struct. Biotechnol. J..

[903] Odoemelam C.S., Percival B., Wallis H., Chang M.W., Ahmad Z., Scholey D., Burton E., Williams I.H., Kamerlin C.L., Wilson P.B. (2020). G-Protein coupled receptors: Structure and function in drug discovery. RSC Adv..

[904] Denisov I.G., Sligar S.G. (2017). Nanodiscs in membrane biochemistry and biophysics. Chem. Rev..

[905] Denisov I.G., Sligar S.G. (2016). Nanodiscs for structural and functional studies of membrane proteins. Nat. Struct. Mol. Biol..

[906] Pollock N.L., Lee S.C., Patel J.H., Gulamhussein A.A., Rothnie A.J. (2018). Structure and function of membrane proteins encapsulated in a polymer-bound lipid bilayer. Biochim. Biophys. Acta-Biomembr..

[907] Loll P.J. (2014). Membrane proteins, detergents and crystals: What is the state of the art?. Acta Crystallogr. Sect. FStructural Biol. Commun..

[908] 9Stetsenko A., Guskov A. (2017). An Overview of the Top Ten Detergents Used for Membrane Protein Crystallization. Crystals.

[909] Caffrey M. (2015). A comprehensive review of the lipid cubic phase or in meso method for crystallizing membrane and soluble proteins and complexes. Acta Crystallogr. Sect. F Struct. Biol. Commun..

[910] Hameduh T., Haddad Y., Adam V., Heger Z. (2020). Homology modeling in the time of collective and artificial intelligence. Comput. Struct. Biotechnol. J..

[911] Haddad Y., Adam V., Heger Z. (2020). Ten quick tips for homology modeling of high-resolution protein 3D structures. PLOS Comput. Biol..

[912] Muhammed M.T., Aki-Yalcin E. (2019). Homology modeling in drug discovery: Overview, current applications, and future perspectives. Chem. Biol. Drug Des..

[913] Barozet A., Bianciotto M., Vaisset M., Siméon T., Minoux H., Cortés J. (2021). Protein loops with multiple meta-stable conformations: A challenge for sampling and scoring methods. Proteins Struct. Funct. Bioinform..

[914] Kaszuba K., Grzybek M., Orłowski A., Danne R., Róg T., Simons K., Coskun Ü., Vattulainen I. (2015). N-Glycosylation as determinant of epidermal growth factor receptor conformation in membranes. Proc. Natl. Acad. Sci. USA.

[915] Polley A., Orłowski A., Danne R., Gurtovenko A.A., Bernardino de La Serna J., Eggeling C., Davis S.J., Róg T., Vattulainen I. (2017). Glycosylation and Lipids Working in Concert Direct CD2 Ectodomain Orientation and Presentation. J. Phys. Chem. Lett..

[916] Mobarak E., Håversen L., Manna M., Rutberg M., Levin M., Perkins R., Róg T., Vattulainen I., Borén J. (2018). Glucosylceramide modifies the LPS-induced inflammatory response in macrophages and the orientation of the LPS/TLR4 complex in silico. Sci. Rep..

[917] Almén M.S., Nordström K.J.V., Fredriksson R., Schiöth H.B. (2009). Mapping the human membrane proteome: A majority of the human membrane proteins can be classified according to function and evolutionary origin. BMC Biol..

[918] Lefkowitz R.J., Kobilka B.K. (2012). The Nobel Prize in Chemistry 2012.

[919] Royal Swedish Academy of Sciences The Nobel Prize in Physiology or Medicine 1967 Ragnar Granit, Haldan Keffer Hartline and George Wald for Their Discoveries Concerning the Primary Physiological and Chemical Visual Processes in the Eye. https://www.nobelprize.org/prizes/medicine/1967/.

[920] Royal Swedish Academy of Sciences The Nobel Prize in Physiology or Medicine 1988 Sir James W. Black, Gertrude B. Elion and George H. Hitchings for Their Discoveries of Important Principles for Drug Treatment. https://www.nobelprize.org/prizes/medicine/1988/.

[921] Royal Swedish Academy of Sciences The Nobel Prize in Physiology or Medicine 1994 Alfred G. Gilman and Martin Rodbell for Their Discovery of G-proteins and the Role of These Proteins in Signal Transduction in Cells. https://www.nobelprize.org/prizes/medicine/1994/.

[922] Axel R., Buck L.B. (2004). The Nobel Prize in Physiology or Medicine 2004.

[923] Vauquelin G., Packeu A. (2009). Ligands, their receptors and… plasma membranes. Mol. Cell. Endocrinol..

[924] Vauquelin G. (2016). Cell membranes… and how long drugs may exert beneficial pharmacological activity in vivo. Br. J. Clin. Pharmacol..

[925] Vauquelin G. (2010). Rebinding: Or why drugs may act longer in vivo than expected from their in vitro target residence time. Expert Opin. Drug Discov..

[926] Lappano R., Maggiolini M. (2011). G protein-coupled receptors: Novel targets for drug discovery in cancer. Nat. Rev. Drug Discov..

[927] Vauquelin G. (2015). On the ‘micro’-pharmacodynamic and pharmacokinetic mechanisms that contribute to long-lasting drug action. Expert Opin. Drug Discov..

[928] Vauquelin G., Charlton S.J. (2010). Long-lasting target binding and rebinding as mechanisms to prolong in vivo drug action. Br. J. Pharmacol..

[929] Erbaş A., Olvera de la Cruz M., Marko J.F. (2019). Receptor-Ligand Rebinding Kinetics in Confinement. Biophys. J..

[930] Sykes D.A., Parry C., Reilly J., Wright P., Fairhurst R.A., Charlton S.J. (2014). Observed Drug-Receptor Association Rates Are Governed by Membrane Affinity: The Importance of Establishing “Micro-Pharmacokinetic/Pharmacodynamic Relationships” at the β2 -Adrenoceptors. Mol. Pharmacol..

[931] Gherbi K., Briddon S.J., Charlton S.J. (2018). Micro-pharmacokinetics: Quantifying local drug concentration at live cell membranes. Sci. Rep..

[932] Dickson C.J., Hornak V., Velez-Vega C., McKay D.J.J., Reilly J., Sandham D.A., Shaw D., Fairhurst R.A., Charlton S.J., Sykes D.A. (2016). Uncoupling the Structure-Activity Relationships of β2 Adrenergic Receptor Ligands from Membrane Binding. J. Med. Chem..

[933] Sanna M.G., Cahalan S.M., Han G.W., Schuerer S.C., Kuhn P., Stevens R.C., Desale H., Scott F.L., Clemons B., Griffith M.T. (2012). Crystal Structure of a Lipid G Protein-Coupled Receptor. Science.

[934] Hildebrand P.W., Scheerer P., Park J.H., Choe H.W., Piechnick R., Ernst O.P., Hofmann K.P., Heck M. (2009). A ligand channel through the G protein coupled receptor opsin. PLoS ONE.

[935] Strasser A., Wittmann H.J., Seifert R. (2017). Binding Kinetics and Pathways of Ligands to GPCRs. Trends Pharmacol. Sci..

[936] Szlenk C.T., Gc J.B., Natesan S. (2019). Does the lipid bilayer orchestrate access and binding of ligands to transmembrane orthosteric/allosteric sites of G protein-coupled receptors?. Mol. Pharmacol..

[937] Clark R.B., Allal C., Friedman J., Johnson M., Barber R. (1996). Stable activation and desensitization of β2-adrenergic receptor stimulation of adenylyl cyclase by salmeterol: Evidence for quasi-irreversible binding to an exosite. Mol. Pharmacol..

[938] Fronik P., Gaiser B.I., Sejer Pedersen D. (2017). Bitopic Ligands and Metastable Binding Sites: Opportunities for G Protein-Coupled Receptor (GPCR) Medicinal Chemistry. J. Med. Chem..

[939] Volpato D., Kauk M., Messerer R., Bermudez M., Wolber G., Bock A., Hoffmann C., Holzgrabe U. (2020). The Role of Orthosteric Building Blocks of Bitopic Ligands for Muscarinic M1 Receptors. ACS Omega.

[940] Feng Z., Hu G., Ma S., Xie X.Q. (2015). Computational Advances for the Development of Allosteric Modulators and Bitopic Ligands in G Protein-Coupled Receptors. AAPS J..

[941] Wang H., Reinecke B.A., Zhang Y. (2020). Computational insights into the molecular mechanisms of differentiated allosteric modulation at the mu opioid receptor by structurally similar bitopic modulators. J. Comput. Aided. Mol. Des..

[942] Stanley N., Pardo L., Fabritiis G. (2016). De The pathway of ligand entry from the membrane bilayer to a lipid G protein-coupled receptor. Sci. Rep..

[943] Karhu L., Magarkar A., Bunker A., Xhaard H. (2019). Determinants of Orexin Receptor Binding and Activation—A Molecular Dynamics Study. J. Phys. Chem. B.

[944] Bokoch M.P., Jo H., Valcourt J.R., Srinivasan Y., Pan A.C., Capponi S., Grabe M., Dror R.O., Shaw D.E., Degrado W.F. (2018). Entry from the Lipid Bilayer: A Possible Pathway for Inhibition of a Peptide G Protein-Coupled Receptor by a Lipophilic Small Molecule. Biochemistry.

[945] Feng Z., Liang T., Wang S., Chen M., Hou T., Zhao J., Chen H., Zhou Y., Xie X.Q. (2020). Binding Characterization of GPCRs-Modulator by Molecular Complex Characterizing System (MCCS). ACS Chem. Neurosci..

[946] Burger W.A.C., Gentry P.R., Berizzi A.E., Vuckovic Z., van der Westhuizen E.T., Thompson G., Yeasmin M., Lindsley C.W., Sexton P.M., Langmead C.J. (2021). Identification of a Novel Allosteric Site at the M 5 Muscarinic Acetylcholine Receptor. ACS Chem. Neurosci..

[947] Cao R., Giorgetti A., Bauer A., Neumaier B., Rossetti G., Carloni P. (2018). Role of extracellular loops and membrane lipids for ligand recognition in the neuronal adenosine receptor type 2A: An enhanced sampling simulation study. Molecules.

[948] Monk B.C., Tomasiak T.M., Keniya M.V., Huschmann F.U., Tyndall J.D.A., O’Connell J.D., Cannon R.D., McDonald J.G., Rodriguez A., Finer-Moore J.S. (2014). Architecture of a single membrane spanning cytochrome P450 suggests constraints that orient the catalytic domain relative to a bilayer. Proc. Natl. Acad. Sci. USA.

[949] Manikandan P., Nagini S. (2018). Cytochrome P450 Structure, Function and Clinical Significance: A Review. Curr. Drug Targets.

[950] Hasler J.A., Estabrook R., Murray M., Pikuleva I., Waterman M., Capdevila J., Holla V., Helvig C., Falck J.R., Farrell G. (1999). Human cytochromes P450. Mol. Aspects Med..

[951] Burkina V., Rasmussen M.K., Pilipenko N., Zamaratskaia G. (2016). Comparison of xenobiotic-metabolising human, porcine, rodent, and piscine cytochrome P450. Toxicology.

[952] Shalan H., Kato M., Cheruzel L. (2018). Keeping the spotlight on cytochrome P450. Biochim. Biophys. Acta-Proteins Proteomics.

[953] Zanger U.M., Schwab M. (2013). Cytochrome P450 enzymes in drug metabolism: Regulation of gene expression, enzyme activities, and impact of genetic variation. Pharmacol. Ther..

[954] Backman J.T., Filppula A.M., Niemi M., Neuvonen P.J. (2015). Role of Cytochrome P450 2C8 in Drug Metabolism and Interactions. Pharmacol. Rev..

[955] Šrejber M., Navrátilová V., Paloncýová M., Bazgier V., Berka K., Anzenbacher P., Otyepka M. (2018). Membrane-attached mammalian cytochromes P450: An overview of the membrane’s effects on structure, drug binding, and interactions with redox partners. J. Inorg. Biochem..

[956] Neves Cruz J., Santana De Oliveira M., Gomes Silva S., Pedro Da Silva Souza Filho A., Santiago Pereira D., Lima E Lima A.H., De Aguiar Andrade E.H. (2020). Insight into the Interaction Mechanism of Nicotine, NNK, and NNN with Cytochrome P450 2A13 Based on Molecular Dynamics Simulation. J. Chem. Inf. Model..

[957] Yousefpour A., Modarress H., Goharpey F., Amjad-Iranagh S. (2018). Interaction of drugs amlodipine and paroxetine with the metabolizing enzyme CYP2B4: A molecular dynamics simulation study. J. Mol. Model..

[958] Spinello A., Ritacco I., Magistrato A. (2019). The catalytic mechanism of steroidogenic cytochromes P450 from all-atom simulations: Entwinement with membrane environment, redox partners, and post-transcriptional regulation. Catalysts.

[959] Lonsdale R., Rouse S.L., Sansom M.S.P., Mulholland A.J. (2014). A Multiscale Approach to Modelling Drug Metabolism by Membrane-Bound Cytochrome P450 Enzymes. PLoS Comput. Biol..

[960] Cojocaru V., Balali-Mood K., Sansom M.S.P., Wade R.C. (2011). Structure and dynamics of the membrane-bound cytochrome P450 2C9. PLoS Comput. Biol..

[961] Cui Y.-L.L., Xue Q., Zheng Q.-C.C., Zhang J.-L.L., Kong C.-P.P., Fan J.-R.R., Zhang H.-X.X. (2015). Structural features and dynamic investigations of the membrane-bound cytochrome P450 17A1. Biochim. Biophys. Acta-Biomembr..

[962] Berka K., Paloncýová M., Anzenbacher P., Otyepka M. (2013). Behavior of human cytochromes P450 on lipid membranes. J. Phys. Chem. B.

[963] Baylon J.L., Lenov I.L., Sligar S.G., Tajkhorshid E. (2013). Characterizing the membrane-bound state of cytochrome P450 3A4: Structure, depth of insertion, and orientation. J. Am. Chem. Soc..

[964] Mustafa G., Nandekar P.P., Bruce N.J., Wade R.C. (2019). Differing membrane interactions of two highly similar drug-metabolizing cytochrome P450 isoforms: CYP 2C9 and CYP 2C19. Int. J. Mol. Sci..

[965] Mustafa G., Nandekar P.P., Camp T.J., Bruce N.J., Gregory M.C., Sligar S.G., Wade R.C. (2019). Influence of Transmembrane Helix Mutations on Cytochrome P450-Membrane Interactions and Function. Biophys. J..

[966] Navrátilová V., Paloncýová M., Kajšová M., Berka K., Otyepka M. (2015). Effect of cholesterol on the structure of membrane-attached cytochrome P450 3A4. J. Chem. Inf. Model..

[967] Navrátilová V., Paloncýová M., Berka K., Otyepka M. (2016). Effect of Lipid Charge on Membrane Immersion of Cytochrome P450 3A4. J. Phys. Chem. B.

[968] Mustafa G., Nandekar P.P., Mukherjee G., Bruce N.J., Wade R.C. (2020). The Effect of Force-Field Parameters on Cytochrome P450-Membrane Interactions: Structure and Dynamics. Sci. Rep..

[969] Cojocaru V., Winn P.J., Wade R.C. (2007). The ins and outs of cytochrome P450s. Biochim. Biophys. Acta-Gen. Subj..

[970] Li W., Shen J., Liu G., Tang Y., Hoshino T. (2011). Exploring coumarin egress channels in human cytochrome p450 2a6 by random acceleration and steered molecular dynamics simulations. Proteins Struct. Funct. Bioinform..

[971] Shen Z., Cheng F., Xu Y., Fu J., Xiao W., Shen J., Liu G., Li W., Tang Y. (2012). Investigation of indazole unbinding pathways in CYP2E1 by molecular dynamics simulations. PLoS ONE.

[972] Fishelovitch D., Shaik S., Wolfson H.J., Nussinov R. (2009). Theoretical characterization of substrate access/exit channels in the human cytochrome P450 3A4 enzyme: Involvement of phenylalanine residues in the gating mechanism. J. Phys. Chem. B.

[973] Yu X., Nandekar P., Mustafa G., Cojocaru V., Lepesheva G.I., Wade R.C. (2016). Ligand tunnels in T. brucei and human CYP51: Insights for parasite-specific drug design. Biochim. Biophys. Acta-Gen. Subj..

[974] Jeřábek P., Florián J., Martínek V. (2016). Lipid molecules can induce an opening of membrane-facing tunnels in cytochrome P450 1A2. Phys. Chem. Chem. Phys..

[975] Li J., Zhou Y., Tang Y., Li W., Tu Y. (2020). Dissecting the structural plasticity and dynamics of cytochrome P450 2B4 by molecular dynamics simulations. J. Chem. Inf. Model..

[976] Mouchlis V.D., Bucher D., McCammon J.A., Dennis E.A. (2015). Membranes serve as allosteric activators of phospholipase A 2, enabling it to extract, bind, and hydrolyze phospholipid substrates. Proc. Natl. Acad. Sci. USA.

[977] Mouchlis V.D., Chen Y., Andrew McCammon J., Dennis E.A. (2018). Membrane Allostery and Unique Hydrophobic Sites Promote Enzyme Substrate Specificity. J. Am. Chem. Soc..

[978] Costeira-Paulo J., Gault J., Popova G., Ladds M.J.G.W., van Leeuwen I.M.M., Sarr M., Olsson A., Lane D.P., Laín S., Marklund E.G. (2018). Lipids Shape the Electron Acceptor-Binding Site of the Peripheral Membrane Protein Dihydroorotate Dehydrogenase. Cell Chem. Biol..

[979] Goossens K., Neves R.P.P., Fernandes P.A., De Winter H. (2020). A computational and modeling study of the reaction mechanism of Staphylococcus aureus monoglycosyltransferase reveals new insights on the GT51 family of Enzymes. J. Chem. Inf. Model..

[980] Barnaba C., Sahoo B.R., Ravula T., Medina-Meza I.G., Im S.C., Anantharamaiah G.M., Waskell L., Ramamoorthy A. (2018). Cytochrome-P450-Induced Ordering of Microsomal Membranes Modulates Affinity for Drugs. Angew. Chemie-Int. Ed..

[981] Jeřábek P., Florián J., Martínek V. (2016). Membrane-Anchored Cytochrome P450 1A2–Cytochrome *b*_5_ Complex Features an X-Shaped Contact between Antiparallel Transmembrane Helices. Chem. Res. Toxicol..

[982] Sellner M., Fischer A., Don C.G., Smieško M. (2021). Conformational landscape of cytochrome P450 reductase interactions. Int. J. Mol. Sci..

[983] Cui Y.-L., Wu R.-L. (2017). Molecular dynamics investigations of membrane-bound CYP2C19 polymorphisms reveal distinct mechanisms for peripheral variants by long-range effects on the enzymatic activity. Mol. Biosyst..

[984] Navrátilová V., Paloncýová M., Berka K., Mise S., Haga Y., Matsumura C., Sakaki T., Inui H., Otyepka M. (2017). Molecular insights into the role of a distal F240A mutation that alters CYP1A1 activity towards persistent organic pollutants. Biochim. Biophys. Acta-Gen. Subj..

[985] Caudle K.E., Dunnenberger H.M., Freimuth R.R., Peterson J.F., Burlison J.D., Whirl-Carrillo M., Scott S.A., Rehm H.L., Williams M.S., Klein T.E. (2017). Standardizing Terms for Clinical Pharmacogenetic Test Results: Consensus Terms from the Clinical Pharmacogenetics Implementa- tion Consortium (CPIC). Genet. Med..

[986] Fischer A., Don C.G., Smieško M. (2018). Molecular Dynamics Simulations Reveal Structural Differences among Allelic Variants of Membrane-Anchored Cytochrome P450 2D6. J. Chem. Inf. Model..

[987] Bastos P., Gomes T., Ribeiro L. (2017). Catechol-O-Methyltransferase (COMT): An Update on Its Role in Cancer, Neurological and Cardiovascular Diseases. Rev. Physiol. Biochem. Pharmacol..

[988] Ramsay R.R., Albreht A. (2018). Kinetics, mechanism, and inhibition of monoamine oxidase. J. Neural Transm..

[989] Ramsay R.R. (2016). Molecular aspects of monoamine oxidase B. Prog. Neuro-Psychopharmacology Biol. Psychiatry.

[990] Fišar Z. (2016). Drugs related to monoamine oxidase activity. Prog. Neuro-Psychopharmacology Biol. Psychiatry.

[991] Kumar B., Dwivedi A.R., Sarkar B., Gupta S.K., Krishnamurthy S., Mantha A.K., Parkash J., Kumar V. (2019). 4,6-Diphenylpyrimidine Derivatives as Dual Inhibitors of Monoamine Oxidase and Acetylcholinesterase for the Treatment of Alzheimer’s Disease. ACS Chem. Neurosci..

[992] Mangiatordi G.F., Alberga D., Pisani L., Gadaleta D., Trisciuzzi D., Farina R., Carotti A., Lattanzi G., Catto M., Nicolotti O. (2017). A rational approach to elucidate human monoamine oxidase molecular selectivity. Eur. J. Pharm. Sci..

[993] Ahmad S., Zaib S., Jalil S., Shafiq M., Ahmad M., Sultan S., Iqbal M., Aslam S., Iqbal J. (2018). Synthesis, characterization, monoamine oxidase inhibition, molecular docking and dynamic simulations of novel 2,1-benzothiazine-2,2-dioxide derivatives. Bioorg. Chem..

[994] Larit F., Elokely K.M., Chaurasiya N.D., Benyahia S., Nael M.A., León F., Abu-Darwish M.S., Efferth T., Wang Y.H., Belouahem-Abed D. (2018). Inhibition of human monoamine oxidase A and B by flavonoids isolated from two Algerian medicinal plants. Phytomedicine.

[995] Prah A., Purg M., Stare J., Vianello R., Mavri J. (2020). How Monoamine Oxidase A Decomposes Serotonin: An Empirical Valence Bond Simulation of the Reactive Step. J. Phys. Chem. B.

[996] Abad E., Zenn R.K., Kästner J. (2013). Reaction Mechanism of Monoamine Oxidase from QM/MM Calculations. J. Phys. Chem. B.

[997] Maršavelski A., Vianello R. (2017). What a Difference a Methyl Group Makes: The Selectivity of Monoamine Oxidase B Towards Histamine and N-Methylhistamine. Chem.—A Eur. J..

[998] Vianello R., Domene C., Mavri J. (2016). The Use of Multiscale Molecular Simulations in Understanding a Relationship between the Structure and Function of Biological Systems of the Brain: The Application to Monoamine Oxidase Enzymes. Front. Neurosci..

[999] Apostolov R., Yonezawa Y., Standley D.M., Kikugawa G., Takano Y., Nakamura H. (2009). Membrane attachment facilitates ligand access to the active site in monoamine oxidase A. Biochemistry.

[1000] Allen W.J., Bevan D.R. (2011). Steered molecular dynamics simulations reveal important mechanisms in reversible monoamine oxidase B inhibition. Biochemistry.

[1001] Jones H.B.L., Crean R.M., Mullen A., Kendrick E.G., Bull S.D., Wells S.A., Carbery D.R., Macmillan F., Van Der Kamp M.W., Pudney C.R. (2019). Exposing the Interplay between Enzyme Turnover, Protein Dynamics, and the Membrane Environment in Monoamine Oxidase B. Biochemistry.

[1002] Karpov O.A., Fearnley G.W., Smith G.A., Kankanala J., McPherson M.J., Tomlinson D.C., Harrison M.A., Ponnambalam S. (2015). Receptor tyrosine kinase structure and function in health and disease. AIMS Biophys..

[1003] Karl K., Paul M.D., Pasquale E.B., Hristova K. (2020). Ligand bias in receptor tyrosine kinase signaling. J. Biol. Chem..

[1004] Castrén E., Monteggia L. (2021). Brain-Derived Neurotrophic Factor Signaling in Depression and Antidepressant Action. Biol. Psychiatry.

[1005] Cannarozzo C., Fred S.M., Girych M., Biojone C., Enkavi G., Róg T., Vattulainen I., Casarotto P.C., Castrén E. (2021). Cholesterol-recognition motifs in the transmembrane domain of the tyrosine kinase receptor family: The case of TRKB. Eur. J. Neurosci..

[1006] Tulodziecka K., Diaz-Rohrer B.B., Farley M.M., Chan R.B., Di Paolo G., Levental K.R., Waxham M.N., Levental I. (2016). Remodeling of the postsynaptic plasma membrane during neural development. Mol. Biol. Cell.

[1007] Rantamäki T. (2019). TrkB neurotrophin receptor at the core of antidepressant effects, but how?. Cell Tissue Res..

[1008] Lieto A., Rantamäki T., Vesa L., Yanpallewar S., Antila H., Lindholm J., Rios M., Tessarollo L., Castrén E. (2012). The responsiveness of trkb to bdnf and antidepressant drugs is differentially regulated during mouse development. PLoS ONE.

[1009] Conroy J.N., Jhaveri D.J., Coulson E.J. (2021). Fast-Trk(B)ing the mechanism of antidepressants. Neuron.

[1010] Slattery D.A., Cryan J.F. (2021). Membrane molecules for mood. Trends Neurosci..

[1011] Ateaque S., Barde Y.A. (2021). A new molecular target for antidepressants. Cell Res..

[1012] Kornhuber J., Gulbins E. (2021). New Molecular Targets for Antidepressant Drugs. Pharmaceuticals.

[1013] Pankiewicz P., Szybiński M., Kisielewska K., Gołębiowski F., Krzemiński P., Rutkowska-Włodarczyk I., Moszczyński-Pętkowski R., Gurba-Bryśkiewicz L., Delis M., Mulewski K. (2021). Do small molecules activate the TrkB receptor in the same manner as BDNF? Limitations of published TrkB low molecular agonists and screening for novel TrkB orthosteric agonists. Pharmaceuticals.

[1014] Dahlström M., Madjid N., Nordvall G., Halldin M.M., Vazquez-Juarez E., Lindskog M., Sandin J., Winblad B., Eriksdotter M., Forsell P. (2021). Identification of Novel Positive Allosteric Modulators of Neurotrophin Receptors for the Treatment of Cognitive Dysfunction. Cells.

[1015] Boes D.M., Godoy-Hernandez A., McMillan D.G.G. (2021). Peripheral Membrane Proteins: Promising Therapeutic Targets across Domains of Life. Membranes.

[1016] Sudhahar C.G., Haney R.M., Xue Y., Stahelin R.V. (2008). Cellular Membranes and Lipid-Binding Domains as Attractive Targets for Drug Development. Curr Drug Targets.

[1017] Meuillet E.J., Mahadevan D., Vankayalapati H., Berggren M., Williams R., Coon A., Kozikowski A.P., Powis G. (2003). Specific inhibition of the akt1 pleckstrin homology domain by D-3-deoxy-phosphatidyl-myo-inositol analogues. Mol. Cancer Ther..

[1018] Indarte M., Puentes R., Maruggi M., Ihle N.T., Grandjean G., Scott M., Ahmed Z., Meuillet E.J., Zang S., Lemos R. (2019). An inhibitor of the pleckstrin homology domain of CNK1 selectively blocks the growth of mutant KRAS cells and tumors. Cancer Res..

[1019] Bertuzzi S., Gimeno A., Núñez-Franco R., Bernardo-Seisdedos G., Delgado S., Jiménez-Osés G., Millet O., Jiménez-Barbero J., Ardá A. (2020). Unravelling the Time Scale of Conformational Plasticity and Allostery in Glycan Recognition by Human Galectin-1. Chem.—A Eur. J..

[1020] Parasuraman P., Murugan V., Selvin J.F.A., Gromiha M.M., Fukui K., Veluraja K. (2015). Theoretical investigation on the glycan-binding specificity of Agrocybe cylindracea galectin using molecular modeling and molecular dynamics simulation studies. J. Mol. Recognit..

[1021] Jayakody R.S., Wijewardhane P., Herath C., Perera S. (2018). Bergenin: A computationally proven promising scaffold for novel galectin-3 inhibitors. J. Mol. Model..

[1022] Miller M.C., Cai C., Wichapong K., Bhaduri S., Pohl N.L.B., Linhardt R.J., Gabius H.J., Mayo K.H. (2020). Structural insight into the binding of human galectins to corneal keratan sulfate, its desulfated form and related saccharides. Sci. Rep..

[1023] Newton A.C., Brognard J. (2017). Reversing the Paradigm: Protein Kinase C as a Tumor Suppressor. Trends Pharmacol. Sci..

[1024] Newton A.C. (2018). Protein kinase C: Perfectly balanced. Crit. Rev. Biochem. Mol. Biol..

[1025] 1Talman V., Pascale A., Jäntti M., Amadio M., Tuominen R.K. (2016). Protein Kinase C Activation as a Potential Therapeutic Strategy in Alzheimer’s Disease: Is there a Role for Embryonic Lethal Abnormal Vision-like Proteins?. Basic Clin. Pharmacol. Toxicol..

[1026] Katti S., Igumenova T.I. (2021). Structural insights into C1-ligand interactions: Filling the gaps by in silico methods. Adv. Biol. Regul..

[1027] Alwarawrah M., Wereszczynski J. (2017). Investigation of the effect of bilayer composition on PKCα-C2 domain docking using molecular dynamics simulations. J. Phys. Chem. B.

[1028] Li J., Ziemba B.P., Falke J.J., Voth G.A. (2014). Interactions of Protein Kinase C-α C1A and C1B Domains with Membranes: A Combined Computational and Experimental Study. J. Am. Chem. Soc..

[1029] Lai C.L., Landgraf K.E., Voth G.A., Falke J.J. (2010). Membrane docking geometry and target lipid stoichiometry of membrane-bound PKCα C2 domain: A combined molecular dynamics and experimental study. J. Mol. Biol..

[1030] Geragotelis A.D., Freites J.A., Tobias D.J. (2021). Anomalous Diffusion of Peripheral Membrane Signaling Proteins from All-Atom Molecular Dynamics Simulations. J. Phys. Chem. B.

[1031] Boije af Gennäs G., Talman V., Aitio O., Ekokoski E., Finel M., Tuominen R.K., Yli-Kauhaluoma J. (2009). Design, Synthesis, and Biological Activity of Isophthalic Acid Derivatives Targeted to the C1 Domain of Protein Kinase C. J. Med. Chem..

[1032] Provenzani R., Tarvainen I., Brandoli G., Lempinen A., Artes S., Turku A., Jäntti M.H., Talman V., Yli-Kauhaluoma J., Tuominen R.K. (2018). Scaffold hopping from (5- hydroxymethyl) isophthalates to multisubstituted pyrimidines diminishes binding affinity to the C1 domain of protein kinase C. PLoS ONE.

[1033] Talman V., Tuominen R.K., Boije af Gennäs G., Yli- Kauhaluoma J., Ekokoski E. (2011). C1 Domain-targeted isophthalate derivatives induce cell elongation and cell cycle arrest in HeLa cells. PLoS ONE.

[1034] Jäntti M.H., Talman V., Räsänen K., Tarvainen I., Koistinen H., Tuominen R.K. (2018). Anticancer activity of the protein kinase C modulator HMI-1a3 in 2D and 3D cell culture models of androgenresponsive and androgen-unresponsive prostate cancer. FEBS Open Bio.

[1035] Dickson E.J., Hille B. (2019). Understanding phosphoinositides: Rare, dynamic, and essential membrane phospholipids. Biochem. J..

[1036] Wu E.L., Qi Y., Song K.C., Klauda J.B., Im W. (2014). Preferred orientations of phosphoinositides in bilayers and their implications in protein recognition mechanisms. J. Phys. Chem. B.

[1037] Stahelin R.V., Scott J.L., Frick C.T. (2014). Cellular and molecular interactions of phosphoinositides and peripheral proteins. Chem. Phys. Lipids.

[1038] Moravcevic K., Oxley C.L.L., Lemmon M.A.A. (2012). Conditional peripheral membrane proteins: Facing up to limited specificity. Structure.

[1039] Hammond G.R.V., Balla T. (2015). Polyphosphoinositide binding domains: Key to inositol lipid biology. Biochim. Biophys. Acta-Mol. Cell Biol. Lipids.

[1040] Letunic I., Khedkar S., Bork P. (2021). SMART: Recent updates, new developments and status in 2020. Nucleic Acids Res..

[1041] Kuang G., Bulone V., Tu Y. (2016). Computational studies of the binding profile of phosphoinositide PtdIns (3,4,5) P3 with the pleckstrin homology domain of an oomycete cellulose synthase. Sci. Rep..

[1042] Rosen S.A.J., Gaffney P.R.J., Spiess B., Gould I.R. (2012). Understanding the relative affinity and specificity of the pleckstrin homology domain of protein kinase B for inositol phosphates. Phys. Chem. Chem. Phys..

[1043] Rai S., Mohanty P., Bhatnagar S. (2018). Modeling, dynamics and phosphoinositide binding of the pleckstrin homology domain of two novel PLCs: η1 and η2. J. Mol. Graph. Model..

[1044] Lumb C.N., He J., Xue Y., Stansfeld P.J., Stahelin R.V., Kutateladze T.G., Sansom M.S.P. (2011). Biophysical and computational studies of membrane penetration by the GRP1 pleckstrin homology domain. Structure.

[1045] Psachoulia E., Sansom M.S.P. (2008). Interactions of the pleckstrin homology domain with phosphatidylinositol phosphate and membranes: Characterization via molecular dynamics simulations. Biochemistry.

[1046] Buyan A., Kalli A.C., Sansom M.S.P. (2016). Multiscale Simulations Suggest a Mechanism for the Association of the Dok7 PH Domain with PIP-Containing Membranes. PLoS Comput. Biol..

[1047] Pant S., Tajkhorshid E. (2020). Microscopic Characterization of GRP1 PH Domain Interaction with Anionic Membranes. J. Comput. Chem..

[1048] Lai C.-L., Srivastava A., Pilling C., Chase A.R., Falke J.J., Voth G.A. (2013). Molecular Mechanism of Membrane Binding of the GRP1 PH Domain. J. Mol. Biol..

[1049] Yamamoto E., Kalli A.C.C., Yasuoka K., Sansom M.S.P.S.P. (2016). Interactions of Pleckstrin Homology Domains with Membranes: Adding Back the Bilayer via High-Throughput Molecular Dynamics. Structure.

[1050] Naughton F.B., Kalli A.C., Sansom M.S.P. (2016). Association of Peripheral Membrane Proteins with Membranes: Free Energy of Binding of GRP1 PH Domain with Phosphatidylinositol Phosphate-Containing Model Bilayers. J. Phys. Chem. Lett..

[1051] Yamamoto E., Domański J., Naughton F.B., Best R.B., Kalli A.C., Stansfeld P.J., Sansom M.S.P. (2020). Multiple lipid binding sites determine the affinity of PH domains for phosphoinositide-containing membranes. Sci. Adv..

[1052] Chan K.C., Lu L., Sun F., Fan J. (2017). Molecular Details of the PH Domain of ACAP1BAR-PH Protein Binding to PIP-Containing Membrane. J. Phys. Chem. B.

[1053] Chen L., Du-Cuny L., Moses S., Dumas S., Song Z., Rezaeian A.H., Lin H.K., Meuillet E.J., Zhang S. (2015). Novel Inhibitors Induce Large Conformational Changes of GAB1 Pleckstrin Homology Domain and Kill Breast Cancer Cells. PLoS Comput. Biol..

[1054] Ghoula M., Le Marec A., Magnan C., Le Stunff H., Taboureau O. (2020). Identification of the Interactions Interference Between the PH and START Domain of CERT by Limonoid and HPA Inhibitors. Front. Mol. Biosci..

[1055] Kumar A., Purohit R. (2013). Cancer Associated E17K Mutation Causes Rapid Conformational Drift in AKT1 Pleckstrin Homology (PH) Domain. PLoS ONE.

[1056] Chandra M., Chin Y.K.Y., Mas C., Feathers J.R., Paul B., Datta S., Chen K.E., Jia X., Yang Z., Norwood S.J. (2019). Classification of the human phox homology (PX) domains based on their phosphoinositide binding specificities. Nat. Commun..

[1057] Psachoulia E., Sansom M.S.P. (2009). PX- and FYVE-mediated interactions with membranes: Simulation studies. Biochemistry.

[1058] Han K., Pastor R.W., Fenollar–Ferrer C. (2020). PLD2–PI(4,5)P2 interactions in fluid phase membranes: Structural modeling and molecular dynamics simulations. PLoS ONE.

[1059] Kalli A.C., Campbell I.D., Sansom M.S.P. (2013). Conformational Changes in Talin on Binding to Anionic Phospholipid Membranes Facilitate Signaling by Integrin Transmembrane Helices. PLoS Comput. Biol..

[1060] Orłowski A., Kukkurainen S., Pöyry A., Rissanen S., Vattulainen I., Hytönen V.P., Róg T. (2015). PIP2 and Talin Join Forces to Activate Integrin. J. Phys. Chem. B.

[1061] Kukkurainen S., Azizi L., Zhang P., Jacquier M.-C., Baikoghli M., von Essen M., Tuukkanen A., Laitaoja M., Liu X., Rahikainen R. (2020). The F1 loop of the talin head domain acts as a gatekeeper in integrin activation and clustering. J. Cell Sci..

[1062] Zhou J., Aponte-Santamaría C., Sturm S., Bullerjahn J.T., Bronowska A., Gräter F. (2015). Mechanism of Focal Adhesion Kinase Mechanosensing. PLoS Comput. Biol..

[1063] Herzog F.A., Braun L., Schoen I., Vogel V. (2017). Structural Insights How PIP2 Imposes Preferred Binding Orientations of FAK at Lipid Membranes. J. Phys. Chem. B.

[1064] Elliott P.R., Goult B.T., Kopp P.M., Bate N., Grossmann J.G., Roberts G.C.K., Critchley D.R., Barsukov I.L. (2010). The structure of the talin head reveals a novel extended conformation of the FERM domain. Structure.

[1065] Busse R.A.A., Scacioc A., Krick R., Pérez-Lara Á., Thumm M., Kühnel K. (2015). Characterization of PROPPIN-phosphoinositide binding and role of loop 6CD in PROPPIN-membrane binding. Biophys. J..

[1066] Krick R., Busse R.A., Scacioc A., Stephan M., Janshoff A., Thumm M., Kühnel K. (2012). Structural and functional characterization of the two phosphoinositide binding sites of PROPPINs, a β-propeller protein family. Proc. Natl. Acad. Sci. USA.

[1067] Lai C.-L., Jao C.C., Lyman E., Gallop J.L., Peter B.J., McMahon H.T., Langen R., Voth G.A. (2012). Membrane Binding and Self-Association of the Epsin N-Terminal Homology Domain. J. Mol. Biol..

[1068] Thallmair V., Schultz L., Marrink S.J., Oliver D., Thallmair S. (2020). A second PI (4, 5) P2 binding site determines PI (4, 5) P2 sensitivity of the tubby domain. bioRxiv.

[1069] Muller M.P., Wang Y., Morrissey J.H., Tajkhorshid E. (2017). Lipid specificity of the membrane binding domain of coagulation factor X. J. Thromb. Haemost..

[1070] Sandvig K., Bergan J., Kavaliauskiene S., Skotland T. (2014). Lipid requirements for entry of protein toxins into cells. Prog. Lipid Res..

[1071] Basu I., Mukhopadhyay C. (2014). Insights into Binding of Cholera Toxin to GM1 Containing Membrane. Langmuir.

[1072] Gangopadhyay A., Chakraborty H.J., Datta A. (2019). Employing virtual screening and molecular dynamics simulations for identifying hits against the active cholera toxin. Toxicon.

[1073] 1Li F., Shrivastava I.H., Hanlon P., Dagda R.K., Gasanoff E.S. (2020). Molecular mechanism by which cobra venom cardiotoxins interact with the outer mitochondrial membrane. Toxins.

[1074] Martín-Acebes M.A., de Oya N.J., Saiz J.C. (2019). Lipid metabolism as a source of druggable targets for antiviral discovery against zika and other flaviviruses. Pharmaceuticals.

[1075] Nitenberg M., Makshakova O., Rocha J., Perez S., Maréchal E., Block M.A., Girard-Egrot A., Breton C. (2020). Mechanism of activation of plant monogalactosyldiacylglycerol synthase 1 (MGD1) by phosphatidylglycerol. Glycobiology.

[1076] Koss H., Bunney T.D., Esposito D., Martins M., Katan M., Driscoll P.C. (2018). Dynamic Allostery in PLCγ1 and Its Modulation by a Cancer Mutation Revealed by MD Simulation and NMR. Biophys. J..

[1077] Robertson R.M., Yao J., Gajewski S., Kumar G., Martin E.W., Rock C.O., White S.W. (2017). A two-helix motif positions the active site of lysophosphatidic acid acyltransferase for catalysis within the membrane bilayer Rosanna. Nat. Struct. Mol. Biol..

[1078] Cozza G., Rossetto M., Bosello-Travain V., Maiorino M., Roveri A., Toppo S., Zaccarin M., Zennaro L., Ursini F. (2017). Glutathione peroxidase 4-catalyzed reduction of lipid hydroperoxides in membranes: The polar head of membrane phospholipids binds the enzyme and addresses the fatty acid hydroperoxide group toward the redox center. Free Radic. Biol. Med..

[1079] Grabon A., Orłowski A., Tripathi A., Vuorio J., Javanainen M., Róg T., Lönnfors M., McDermott M.I.M.I., Siebert G., Somerharju P. (2017). Dynamics and energetics of the mammalian phosphatidylinositol transfer protein phospholipid exchange cycle. J. Biol. Chem..

[1080] Lu J., Chan C., Yu L., Fan J., Sun F., Zhai Y. (2020). Molecular mechanism of mitochondrial phosphatidate transfer by Ups1. Commun. Biol..

